# The differential impact of scientific quality, bibliometric factors, and social media activity on the influence of systematic reviews and meta-analyses about psoriasis

**DOI:** 10.1371/journal.pone.0191124

**Published:** 2018-01-29

**Authors:** Juan Ruano, Macarena Aguilar-Luque, Francisco Gómez-Garcia, Patricia Alcalde Mellado, Jesus Gay-Mimbrera, Pedro J. Carmona-Fernandez, Beatriz Maestre-López, Juan Luís Sanz-Cabanillas, José Luís Hernández Romero, Marcelino González-Padilla, Antonio Vélez García-Nieto, Beatriz Isla-Tejera

**Affiliations:** 1 Department of Dermatology, Reina Sofía University Hospital, Menéndez Pidal Ave, 14004 Córdoba, Spain; 2 Instituto Maimonides de Investigación Biomédica de Córdoba (IMIBIC)/Reina Sofía University Hospital/University of Córdoba, Menendez Pidal Ave, 14004 Cordoba, Spain; 3 School of Medicine, University of Cordoba, Menéndez Pidal Ave, 14004 Córdoba, Spain; 4 Department of Pharmacy, Reina Sofía University Hospital, Menéndez Pidal Ave, 14004 Córdoba, Spain; Dalian University of Technology, CHINA

## Abstract

Researchers are increasingly using on line social networks to promote their work. Some authors have suggested that measuring social media activity can predict the impact of a primary study (i.e., whether or not an article will be highly cited). However, the influence of variables such as scientific quality, research disclosures, and journal characteristics on systematic reviews and meta-analyses has not yet been assessed. The present study aims to describe the effect of complex interactions between bibliometric factors and social media activity on the impact of systematic reviews and meta-analyses about psoriasis (PROSPERO 2016: CRD42016053181). Methodological quality was assessed using the Assessing the Methodological Quality of Systematic Reviews (AMSTAR) tool. Altmetrics, which consider Twitter, Facebook, and Google+ mention counts as well as Mendeley and SCOPUS readers, and corresponding article citation counts from Google Scholar were obtained for each article. Metadata and journal-related bibliometric indices were also obtained. One-hundred and sixty-four reviews with available altmetrics information were included in the final multifactorial analysis, which showed that social media and impact factor have less effect than Mendeley and SCOPUS readers on the number of cites that appear in Google Scholar. Although a journal’s impact factor predicted the number of tweets (OR, 1.202; 95% CI, 1.087–1.049), the years of publication and the number of Mendeley readers predicted the number of citations in Google Scholar (OR, 1.033; 95% CI, 1.018–1.329). Finally, methodological quality was related neither with bibliometric influence nor social media activity for systematic reviews. In conclusion, there seems to be a lack of connectivity between scientific quality, social media activity, and article usage, thus predicting scientific success based on these variables may be inappropriate in the particular case of systematic reviews.

## Introduction

The dissemination of research results is necessary for scientific progress because critical assessment of published research promotes new hypotheses that lead to future experiments. Traditionally, authors have used scientific meetings and journals to distribute their results. However, the emergence of the Internet and social networking platforms such as Twitter and Facebook has allowed such reports to be accessible almost immediately to a wider audience, by connecting one sender with a vast number of readers [1].

It is desirable to read articles with the highest scientific standards, and least amount of bias, but it is beyond the capacity of almost researchers to review all such studies [2]. Many studies have been carried out with the aim to analyze whether social media activity generated by the scientific community about a new publication anticipates the number of cites an article will receive in the future, as a surrogate marker of its scientific quality [3–10]. However, there are several factors involved in the dissemination process that may be in conflict. First, publishers of scientific journals and the pharmaceutical industry are interested in obtaining a large number of readers and cites per article, so that they can increase their product’s impact factor [11] or the visibility of studies carried out with their products, which in many cases present conflicts of interests with theses [12, 13]. Secondly, scientific quality in biomedicine does not depend on statistically positive results, but on high standards of methodological quality, lack of bias, transparency of the scientific method, reproducibility of research results, and clinical usability, some features that may not be neatly summarized merely by bibliometric impact indices [14]. Nevertheless, scientific quality is more related to methodological rigor than to the impact of research. In this sense, there are validated tools to evaluate the methodological quality of research but they are not available for all types of scientific research.

To date, articles that have analyzed social media as a predictor of future cites have only evaluated factors such as author’s scientific production or journal’s bibliometric characteristics, without considering article’s scientific quality or research disclosures [15]. Moreover, these articles have focused on primary studies but not on systematic reviews (SRs). SRs are considered the standard for the synthesis of the evidence that often are used as a starting point for the development of clinical practice guidelines, establishing the recommendations of diagnostic, prognostic, and/or therapeutic interventions [16]. Assessing the Methodological Quality of Systematic Reviews (AMSTAR) has proven to be a valid and reliable tool to assess the methodological quality of SRs and is the most universally accepted [17].

Psoriasis is a highly prevalent immune-mediated chronic skin disease whose moderate-severe forms are associated with significant comorbidity, impaired quality of life, and increased medical costs [18, 19]. In recent years, highly effective treatments have been developed but with potentially serious adverse effects and a high cost. Making decisions based on the best evidence improves health outcomes and allows the sustainability of health systems. Regardless of the importance of the conclusions of SRs, no studies have explored the influence of methodological quality on the use of social media to disseminate their results.

For all these reasons, our aim was to evaluate the influence of scientific quality and research disclosures on the bibliometrics and altmetrics of systematic reviews of psoriasis.

## Materials and methods

### Protocol and eligibility criteria

To begin, we established an *a priori* protocol and published it in the PROSPERO International Prospective Register of Systematic Reviews (PROSPERO 2016: CRD42016053181). In this protocol, we included SRs or MAs published in scientific journals related to skin psoriasis. Abstracts of congresses, case reports, surveys, narrative reviews, narrative reports (i.e., reports that have a particular focus on understanding a concept), clinical practice guidelines, consensus documents, MAs performed without a systematic literature search, and reviews titled as literature reviews or integrative reviews were not included. Further, as a result of a time limitation to complete the project, the documents retrieved were restricted to English-language reviews. No limitation was placed on the year of publication or study population.

### Search methods

SRs and MAs published up to July 4, 2016 were identified in MEDLINE, EMBASE, and the Cochrane Database through a comprehensive systematic Boolean search using MeSH terms (“psoriasis”/exp or psoriasis) and (“meta analysis” or “systematic review”). We identified additional eligible studies by searching the reference lists of the included SRs, MAs, and health technology assessment (HTA) reports. We contacted study authors when necessary to identify additional information that may not have been captured.

### Methods for identification and selection

Authors independently performed tasks related to study filtering and selection (FG-G, MG-P, PJG-F, and BI-T) and data extraction (FG-G, JG-M, MG-P, MA-L, PJG-F, and BI-T). Screening was performed in two stages. In the first stage, abstracts downloaded from literature searches were screened, and any study that clearly failed to meet eligibility criteria was rejected. In the second stage, full papers were retrieved for the remaining candidate study and reviewed to identify all SRs and MAs that met the eligibility criteria. In uncertain or controversial cases, all discrepancies identified during the first stage and throughout the review were resolved via discussion; for select cases, this process involved a different investigator (JR).

### Assessment of methodological quality

Data were analyzed from August 30 to September 15, 2016. A 10-study pilot evaluation was performed prior to evaluation of the selected articles to standardize usage and eliminate inconsistencies. Two investigators (FG-G, MA-L) independently assessed the methodological quality of each SR using data abstraction forms and 11 criteria from the Assessing the Methodological Quality of Systematic Reviews (AMSTAR) tool [17]; quality assessment discrepancies were discussed with a third author (JR) until an agreement was reached. We identified and discussed differences in quality between reviews and used the review quality assessment to interpret the results synthesized in this overview. The 11 AMSTAR criteria were rated as “yes” (criteria were met), “no” (criteria were not met), “cannot answer” (unclear information), or “not applicable” (criteria could not be evaluated because of the design of background studies in the reviews) (Table C in S1 File). For all items except number 4, ratings of “yes” received a score of 1, whereas “no”, “cannot answer” and “not applicable” received a score of 0. For item 4, a rating of “no” (i.e., the review did not exclude unpublished or grey literature) was considered adequate. The highest possible AMSTAR score was 11, and scores were used to classify review quality as follows: 0–4 = low quality, 5–8 = moderate quality, and 9–11 = high quality. Total AMSTAR scores were summarized descriptively as medians and interquartile ranges or as percentages of achievement per item. Inter-rater agreement was tested using Cohens’s *kappa* (for squared) using the R-language *irr* package (R Project for Statistical Computing, Vienna, Austria). Kappa values ranged from -1.0 to 1.0, with -1.0 indicating perfect disagreement below chance, 0.0 indicating agreement equal to chance, and 1.0 indicating perfect agreement above chance. Generally, a kappa value of ≥0.70 indicates adequate inter-rater agreement; in this study, a value of ≥0.65 was chosen to indicate sufficient agreement.

### Bibliometrics and altmetrics data extraction

We independently obtained metadata from articles and journals related to studies that fulfilled the inclusion criteria (title, year of publication, journal’s name, source of funding, number of authors with conflict of interest, and impact factor from InCites^™^ Journal Citation Reports^®^ (Clarivate Analytics, Philadelphia, PA, USA). Altmetrics comprise quantitative data from social media sites, science blogs, many mainstream media outlets, and reference managers in terms of mentions of academic papers; altmetrics are complementary to traditional, citation-based metrics. Altmetrics uses 3 parameters: source document, identifier, and received mentions. The identifiers help to recognize the different versions of an investigation. The Altmetrics are calculated automatically by weighted count according to the received attention based on: number of mentions, source, and authors. Depending on where they have been mentioned or who has done so, they get a score or another. Finally, a global score is calculated taking into account all of the above. In our study, altmetric data scraping was performed using the *rAltmetric* R package [20]. For the same data (July 12, 2017), Twitter, Facebook, and Google+ mention counts, Mendeley and SCOPUS readers, and corresponding article citation counts from Google scholar were obtained using the Digital Object Identifier (DOI) of the included reviews.

### Data analysis

A range of approaches were used to capture the results of the included reviews. Article and journal metadata were collected using standardized data extraction templates implemented in *AppSheet* (https://www.appsheet.com/), a custom mobile app based on Google forms. Missing data were imputed using bootstrapping and the Expectation-Maximization algorithm implemented in the *Amelia II* R package. Multivariate exploratory data analysis was performed to characterize the reviews using the Multiple Factor Analysis (MFA) method implemented with the *FactorMineR* R package. Individual reviews and variable sub-analyses were performed. MFA is used to analyze a data set in which individuals are described by several sets of variables (i.e., quantitative and/or qualitative) structured into groups. This method allows comparison of group variables and highlighting of a typology of the groups or simultaneously comparison of the typologies of reviews seen by each group of variables one by one. Following this approach, two sets of variables were considered close to each other if reviews were similar for the first as well as the second set of variables. Clustering dendrogram comparisons were performed using the *dendextend* R package. To improve the layout of the trees an optimal rotation was found using the untangle function with the “step2side” method.

### Differences between protocol and overview

Our planned search strategy, as recorded in PROSPERO, was compared with the final reported review methods. We did not add, omit, or change the outcomes after our protocol was published. Consideration was restricted to English-language reviews because of time limitations placed on project completion. Sub-analysis of the influence of altmetrics data was not included in the original protocol.

### Reproducibility of results

Several R language packages were used to produce graphs and perform the statistical analyses. Our analyses can be fully reproduced using raw data source file and R scripts stored at our GitHub hosting repository (https://github.com/info4cure/altmetricsAndSRsOnPsoriasis).

### Ethics

No ethical approval was required.

## Results

### Search results

**Fig 1:**
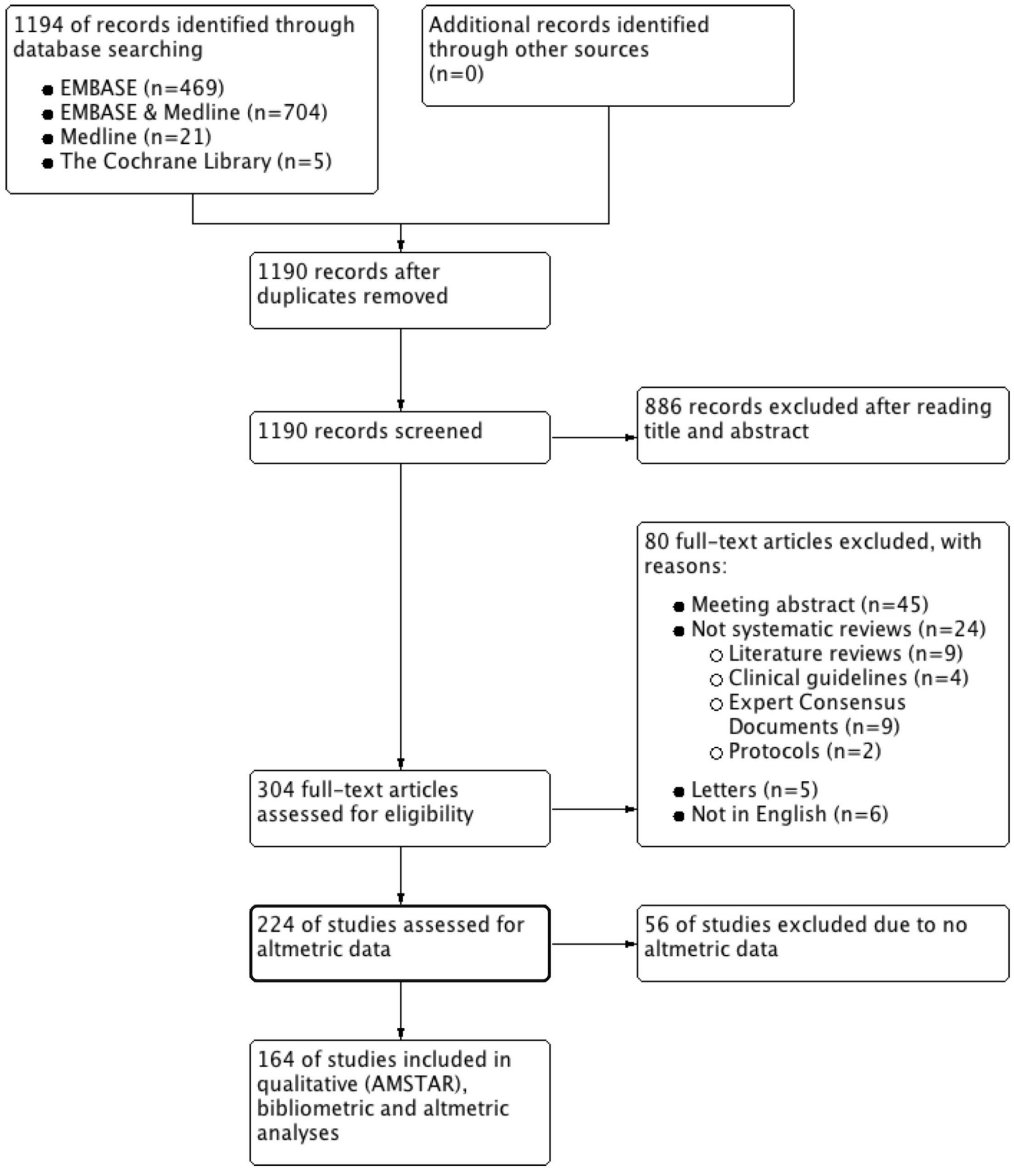
PRISMA flow diagram of article selection process.

The database search yielded 1195 potentially relevant titles (699 EMBASE & MEDLINE; 474 EMBASE; 22 MEDLINE; 4 Cochrane Database). After excluding duplicates and screening abstracts, 304 studies remained eligible for full-text review. From 92 peer-reviewed journals, 220 reviews were subjected to quality assessment (see Fig 1 for the PRISMA flowchart and Tables A and B in S1 File for included and excluded studies, respectively). Finally, 164 reviews with available altmetrics information were selected to perform all data analyses.

### Characteristics of reviews

From 2011 to 2016, 164 reviews were published by 926 authors from 580 different institutions and 10 countries in 72 peer-reviewed journals (30.5% in dermatology journals), with a median impact factor of 3.057 (range 0.5-30.03). The median number of authors and institutions per review was five (range, 2-20) and one (range 1-3), respectively. Most reviews were funded by an academic institution (27.4%) or pharmaceutical company (32.9%), and 65.5% were authored by at least one researcher with a conflict of interest. Reviews received at least one comment on a social network (Facebook: median 4, range 1-116; Twitter: median 2, range 1-89; Google +: median 3, range 1-101); the median number of readers on Mendeley or SCOPUS was 19 (range, 0-327); and the median of cites on Google Scholar was 14 (range, 0-426). The AMSTAR statement was used to assess methodology quality after substantial inter-rater agreement was achieved [*kappa* = 0.75, 95% CI, 0.69-0.82]. The median AMSTAR score of the 164 reviews, including both SRs and MAs, was 6 (1–11); 22.2%, 53.3%, and 24.5% of reviews were classified as high, moderate, or low methodological quality, respectively.

### Chronological changes of bibliometric and altmetric features

**Fig 2:**
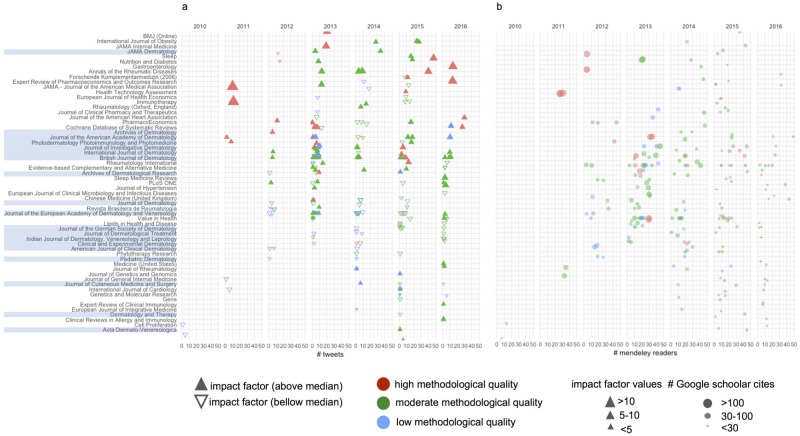
Bibliometric and altmetric features of systematic reviews and meta-analysis on psoriasis. Journals were sorted based on median Twitter mention counts considering all published articles in the same journal. Reviews are represented by colored figures based on AMSTAR levels. (a) Articles are displayed based on journal, year of publication, and Twitter mention counts. Triangles that represent reviews are colored based on their methodological quality (AMSTAR level). Up- and down- triangles represent reviews published in journals whose impact factor is above or bellow the median of journal in our dataset, respectively. Triangle size is proportional to the value of journal’s impact factor. (b) Articles are represented by circles and displayed based on journal, year of publication, and number of readers on Mendeley. Circle size is proportional to Google scholar cites.

Fig 2 depicts a panel of two plots showing the chronology of reviews by journal and year of publication with some additional bibliometric and altmetric features. As reviews are displayed from top to bottom in descending order based on the number of tweets, we can highlight several features: 1) most Dermatology journals are near the middle of the figure; 2) reviews with the greatest number of tweets were published in non-dermatology journals; and 3) there seems to be a gradient from top to bottom related to methodological quality and impact factor (Fig 2a), and from top to bottom and from left to right concerning the number of cites in Google Scholar (Fig 2b).

### Multifactorial analysis

**Fig 3:**
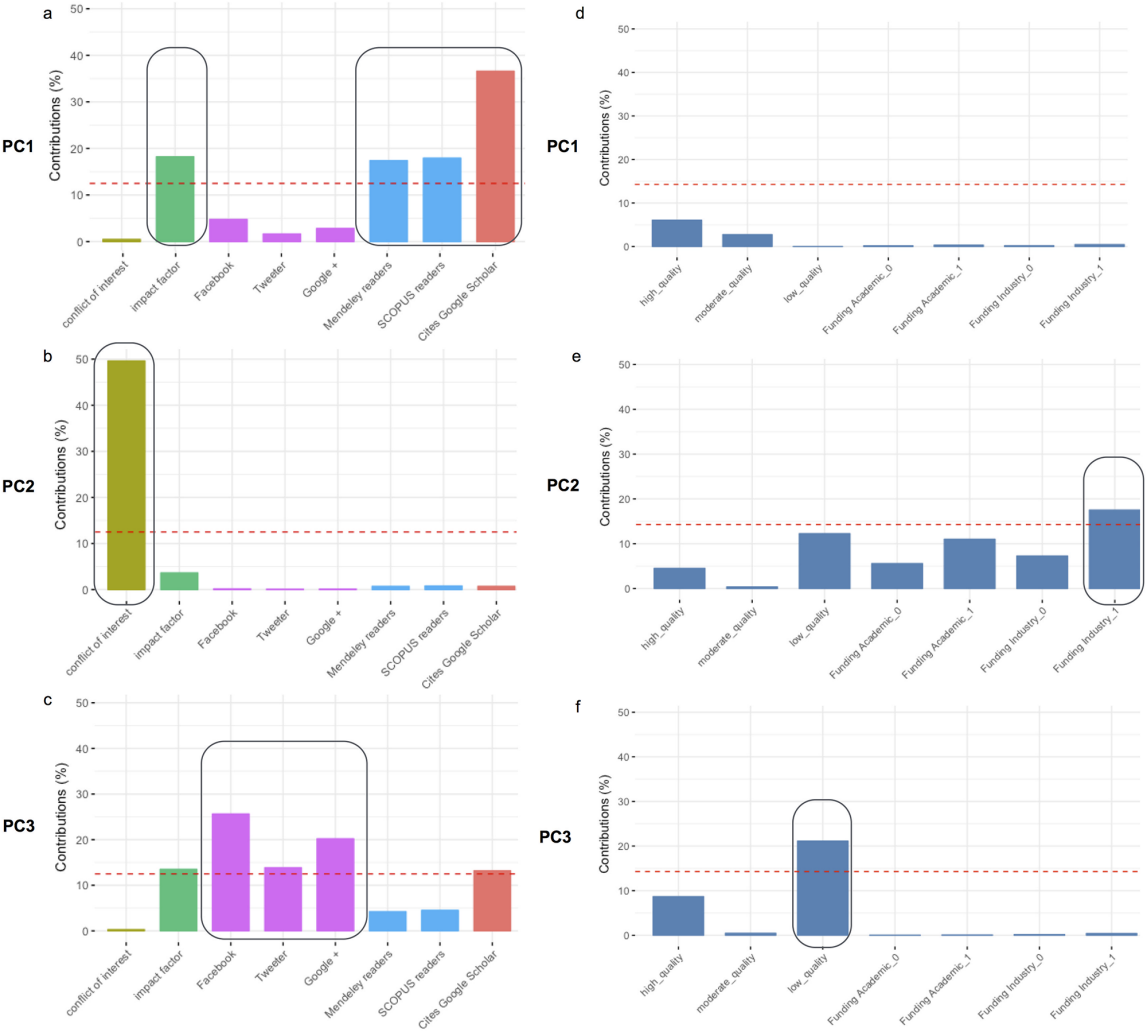
Contribution of quantitative and qualitative variables to principal components. This panel of six plots display the percentage of contribution to components PC1 (a-d), PC2 (b-e), and PC3 (c-f) by quantitative (a-c) and qualitative (d-f) variables.

Multifactorial analysis was used to convert the vectors of values for quantitative and qualitative variables per article into a set of linearly uncorrelated variables called principal components (PCs). This transformation is defined in such a way that the first principal component (PC1) has the largest possible variance, and each succeeding component in turn has the highest variance possible under the constraint that it is orthogonal to the preceding components. In this regards, PC1, PC2, and PC3 explained 32.2%, 25.7%, and 16.2% of the variability in our dataset, respectively (Fig A in S1 File). After the PCA, we have plotted the percentage of contribution of every factor (quantitative factors on the left, a-c; qualitative factors on the right, d-f) to the three most important components. PC1 is an orthogonal combination of all factors that in our case it explains 32.2% of the review variability. Fig 3a, shows that most of this variability is due to three variables: Google Scholars, Mendeley and SCOPUS readers, and impact factor. Thus, PC1 is dominated by bibliometric and usage group of variables. Considering Fig 3b, review variability due to PC2 is almost exclusively explained by conflict of interest and funding industry variables. Finally, media variables (Facebook, Twitter, and Google+ mentions) and low methodological quality reviews contribute to most of the variability of reviews explained by PC3, a total of only 15% (Fig 3c).

**Fig 4:**
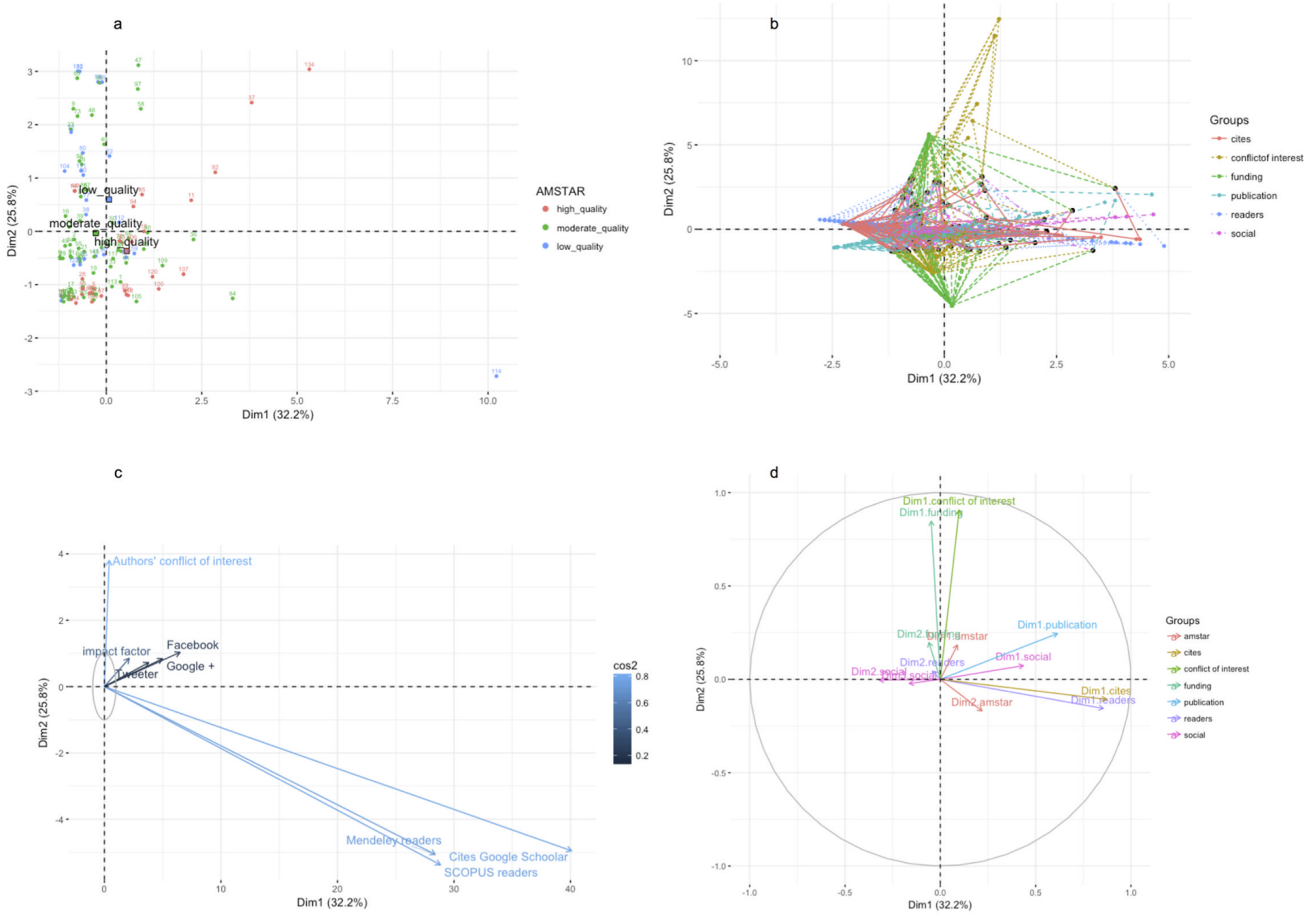
Multiple factor analyses (MFA). This panel display four plots PC1—PC2 scores of reviews (a), variables (c), and group of variables (b-d). Fig 3a: reviews are colored based on methodological quality (red: high; green: moderate; blue: low); Fig 3b and 3d: projections of reviews to variable position and coordinates of the partial axes by variable were colored by group; Fig 3c: A gradient scale of blues represent values of squared cosines associated with PC1-PC2 variable projections.

Fig 4 represents a panel of figures, where reviews (Fig 4a and 4b) or variables (Fig 4c and 4d) are mapped into a PC1 vs PC2 coordinate system. We have selected the first two components of PCA (both explain 58.2% of review variation) to study their influence on every single review or variable. In Fig 4a, the position of most low or moderate methodological quality reviews are determined by PC2 to which they mainly contribute conflict of interest and funding variables (Fig 4c and 4d). Most of the reviews of high methodological quality are represented in two groups: a) one group placed in the 1st quadrant is influenced equally by PC1 and PC2, thus reviews being dominated by conflict of interest, bibliometric, and social media variables, but all them having a low contribution to the PC1 vs PC2 explained variability (Fig 4c and 4d); b) a second group, mainly located in the 4th quadrant, is influenced equally by PC1 and PC2, and therefore dominated negatively by the number of author’s with conflict of interest and positively influenced by the number of readers and the number of cites (Fig 4b and 4c). This is very interesting because it demonstrates that some high methodological quality reviews may be driven by strategies of social diffusion and disclosure factors, whereas some other reviews would be directed by the usability that other researchers without conflict of interest make of them.

### Regression analysis

Regression analysis showed that social media activity and a journal’s impact factor had less influence than number of Mendeley and Scopus readers on the number of Google Scholar cites (Table 1). Although a journal’s impact factor predicted the number of tweets associated with an article [OR, 1.202; 95%CI, 1.087-1.049], the years of publication and the number of Mendeley readers better predicted the number of cites in Google Scholar [OR, 1.033; 95%CI, 1.018-1.329]. Methodological quality was related neither with bibliometric influence nor social media activity.

**Table 1:** Univariate and multivariate regression models.

**Model: # Google scholar cites**		**Estimate (SE)**	**t value**	**p value**	**OR [CI95%]**
Methodological quality	*High*	-	-	-	
	*Moderate*	-0.237 (0.299)	-0.793	ns	
	*Low*	-0.417 (0.396)	-1.052	ns	
Funding sources	*Academic*	-0.542 (0.291)	-1.863	ns	
	*Pharma*	-0.289 (0.357)	-0.808	ns	
*Author*′*s* *conflict* *of* *interest*		0.009 (0.038)	0.253	ns	
*Impact* *factor*		0.029 (0.059)	0.500	ns	
*Year* *of* *publication*	2011	-	-	-	
	2012	0.545 (0.944)	0.577	ns	
	2013	0.629 (0.873)	0.720	ns	
	2014	-0.383 (0.893)	-0.429	ns	
	2015	-2.156 (0.903)	-2.388	*<0.016*	0.115 [0.019; 0.679]
	2016	-3.645 (0.930)	-3.917	*<0.001*	0.026 [0.004; 0.161]
*Number* *of* *tweets*		0.015 (0.020)	0.754	ns	
*Mendeley*′*s* *readers*		0.033 (0.007)	4.263	*<0.001*	1.033 [1.018; 1.049]
**Model: # Tweets**		**Estimate**	**SE**	**t value**	**OR [CI95%]**
Methodological quality	*High*	-	-	-	
	*Moderate*	0.627 (0.548)	1.142	ns	
	*Low*	0.321 (0.629)	0.510	ns	
Funding sources	*Academic*	0.750 (0.557)	1.346	ns	
	*Pharma*	-0.271 (0.687)	-0.394	ns	
*Author*′*s* *conflict* *of* *interest*		0.221 (0.120)	1.833	ns	
*Impact* *factor*		0.184 (0.051)	3.584	*<0.001*	1.202 [1.087; 1.329]
*Year* *of* *publication*	2011	-	-	-	
	2012	1.316 (0.863)	0.271	ns	
	2013	1.053 (0.807)	0.406	ns	
	2014	1.139 (0.811)	-2.661	ns	
	2015	1.360 (0.804)	-2.436	ns	
	2016	0.690 (0.823)	-4.26	ns	

…

### Clustering analysis

In order to discover clusters of reviews, articles were ranked based on a K-means clustering algorithm using the three most informative components of MFA (PC1, PC2, PC3), quality of representation (cos2) and contribution per review Fig 4.

**Fig 5:**
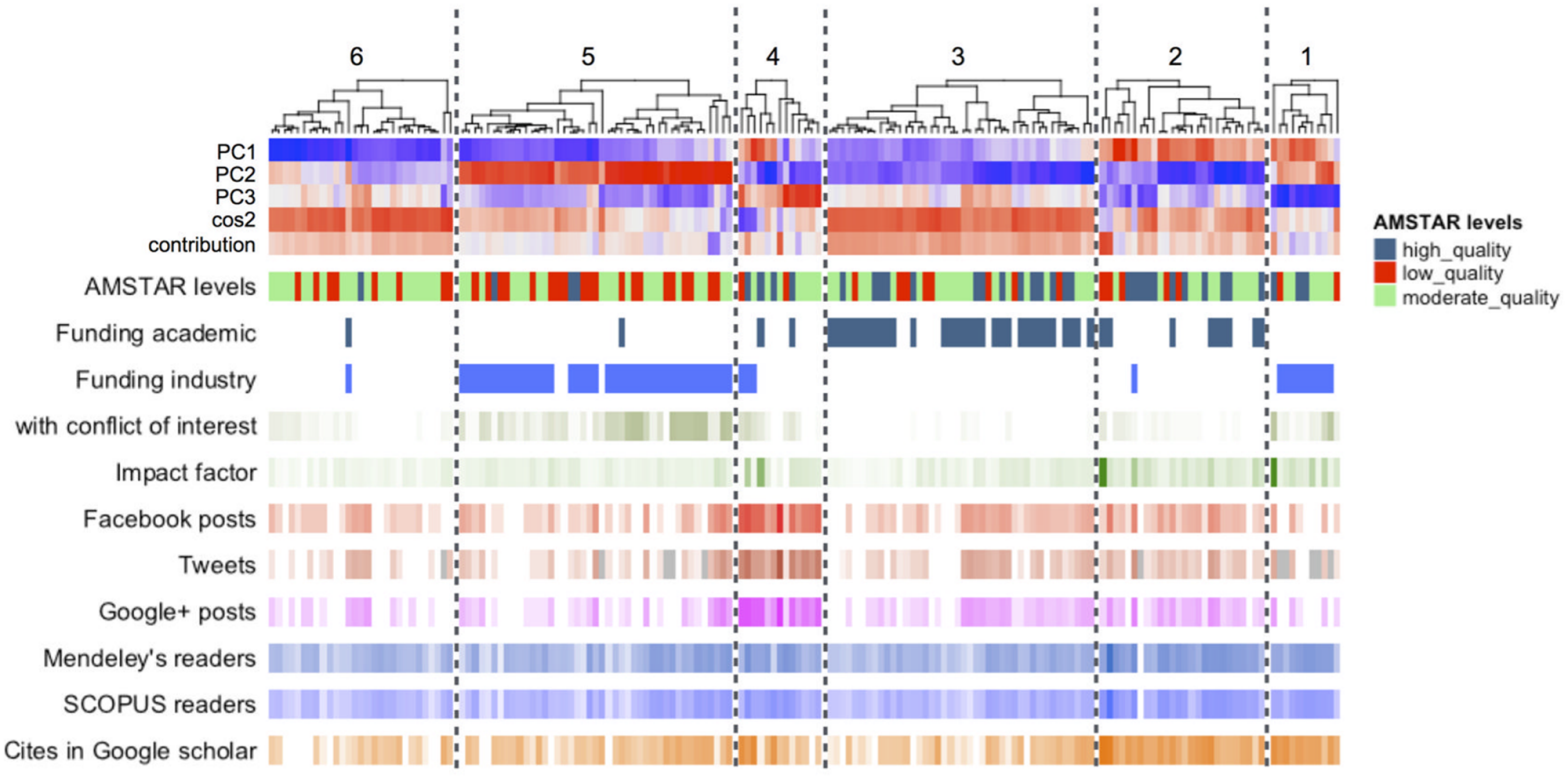
Scale reduction of SRs by multifactorial analysis (MFA). Clustering heat map of all include reviews based on PC1, PC2, and PC3 projections, and the quality and contribution of these values per review. Six clusters (1-6) were indentified. Article- and journal-related bibliometric and altmetric metadata are also displayed as individual heat maps.

Fig 5 shows a complex heatmap where reviews (on the top) are reordered using a clustering dendrogram algorithm based on the info of every review derived from the PCA: PC1, PC2, and PC3 individual values, and cos2 and contribution. The rest of the heatmaps (article- and journal-related bibliometric and altmetric metadata) are also displayed as individual heat maps sorted based on the established order by first review clustering. These extra heatmaps show pattern distribution of single values for every variable, thus helping us to describe by all these features every cluster of reviews. Two major groups are worth highlighting: the group of reviews financed mainly by the pharmaceutical industry (*cluster #1* and *cluster #5*), and the group of reviews financed mainly by the academy (*cluster #2* and *cluster #3*). In both groups, the clusters differed in terms of impact factor of the journal in which the article was published and the number of cites received (i.e., a greater number of cites was received by *cluster #5* vs *cluster #1* and in the *cluster #2* vs *cluster #3*). Interestingly, the activity observed in social networks was somewhat greater in the group of reviews financed by the academy compared with that financed by the pharmaceutical industry; there were no differences between clusters in the same group, however. *Cluster #6* includes reviews that did not identify a source of funding. The profile of *cluster #6* is very similar to *cluster #3*, but *cluster #3* one contains reviews that have received academic funding and whose average impact index is greater. In *cluster #4* we found reviews that showed more social media activity, but did not observe a differentiating pattern related to methodological quality, source of funding, impact factor, number of readers, or number of cites. However, these reviews did not stand out either in terms of number or by their contribution to the multifactorial analysis. Finally, although the majority of high-quality revisions were found in *clusters #2, #3* and *4*, those of moderate or low quality were represented in all other groups.

### Influence of variable groups on the structure of reviews dataset

**Fig 6:**
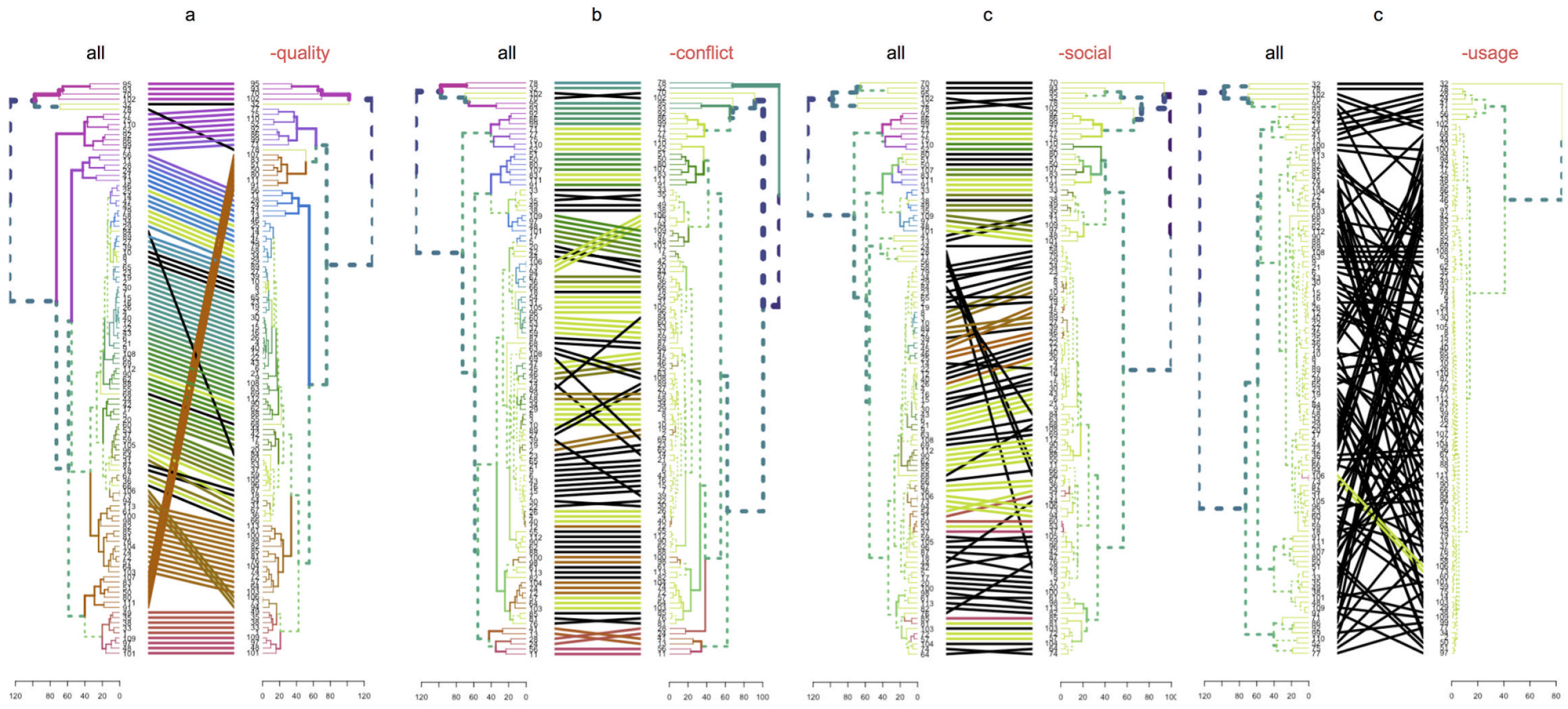
Influence of variable groups on multifactorial-based by clustering dendrogram comparisons. This panel of four plots compare dendrograms with the same set of labels, one facing the other, and having their labels connected by lines. In every comparison, all-variables clustering dendrogram was compared with a modified version of this dendrogram obtained after subtracting one of these group of variables at a time (a, ‘quality’: AMSTAR levels; b, ‘conflict’: source of funding, number of authors with conflict of interest; c, ‘social’: Twitter, Facebook, and Google+ mention counts; d, ‘usage’: Mendeley and SCOPUS readers and citation counts from Google Scholar). Unique nodes are highlighted with dashed lines. Connecting lines are colored to highlight two sub-trees which are present in both dendrograms. Black lines connect nodes not included in the same sub-tree. Same color of trees branches show two common sub-trees.

Variables were structured into groups for clustering dendrogram comparisons in term of “quality” (AMSTAR levels), “conflict” (source of funding, number of authors with conflict of interest), “social” (Twitter, Facebook, and Google+ mention counts), and “usage” (Mendeley and SCOPUS readers and citation counts from Google scholar). Fig 6a–6d is a panel of plots showing the influence of every group of variables in the architecture of reviews obtained by clustering dendrogram. Clustering dendrogram built with all the group of variables (reference dendrogram: “all”) is compared with a new dendrogram obtained as a result of clustering the same reviews but without considering one of the group of variables at a time. As reviews are linked at both sides of the plot by a line, the importance of the perturbation introduced by subtracting the variable is proportional to the amount of changes (visualized as many black crossing lines from left to right). In this panel, we can see that the group of variables related with “usage” (Mendeley and Scopus readers and Google cites) is the most important to explain the complex relationship among reviews. On the other hand, methodological quality of reviews has the least influence on the multidimensional architecture of our reviews, while conflict and social factors showed intermediate structural influence.

## Discussion

This is the first study to evaluate the influence of scientific quality and research disclosures on the bibliometrics and altmetrics of systematic reviews of psoriasis. Our primary finding was that the number of citations is not predicted directly by the quality of research, even if there is a significant amount of social media activity associated with an article. Rather, the number of cites that a review receives is linked to the number of readers and the year of its publication. The other major finding was that the number of tweets related to a review were predicted by the journal’s impact factor, thus potentially much of the social media activity around an article is promoted by the authors or the journal itself. Our study did not find an obvious linkage between on-line attention to a review and its academic usage.

Altmetrics are built upon interactions gathered from academic users’ social media networks, but metrics associated with journals and pharmaceutical companies are also considered. Messages flowing in these networks may be generated by users who do not always share the same objectives.

We found that factors explaining most of the variability between reviews in the multifactorial analysis were those related with academic usage and a journal’s impact factor. Therefore, journal-derived citation metrics seem to be related to the number of readers and cites that an article achieves. There are two possible explanations for this phenomenon. First, probably not all researchers in the psoriasis field are part of the publication–citations pipeline. Secondly, even if researchers are active in the publication ecosystem, they must filter all social media messages and focus on publications related to their field and topic of research, and after that download and read the full-text version of an article.

On the other hand, the main contributions to the methodological variability of reviews was due to conflict of interest and funding by the pharmaceutical industry. Interestingly, methodological quality, as a proxy of scientific quality, was the least influential factor in the multifactorial structure of our dataset. Indeed, we did not find quality of research to be a predictor of either social media activity or the number of cites received by an article. This suggests that diffusion activity and citation behavior may be driven by other motivators.

In this sense, Warren HR *et al.* found that factors such as high impact journals, open access journals, or the field of research (i.e., diet, human mortality, exercise or cancer) were the predictors of top 100 most cited articles published in 2015 [21]. On the other hand, it has been described that the desire to convince the peer reviewers and to base the author’s point of view are the main motivations for citing [22] as well as less honorable reasons like to improve a colleague’s bibliometrics, to satisfy a potential referee, or to give the impression that there is a great interest in the topic of research by including many irrelevant citations [23].

Journal- and author-derived complex citation-based metrics such as impact factor and H-index have been criticized by the DoRA declaration (http://www.ascb.org/dora/) and the Leiden manifesto [24], both of which promote transparency around scientific metadata to generate scores that can be easily and independently verified. One of the most successful alternatives to citation-based metrics has been altmetrics, or alternative metrics, which combine online sources such as social networks and public policy documents to provide a fuller understanding of the academic impact of publications [25].

Altmetric data aim to measure the real-time influence of an academic article from the moment it is published. Researchers may then filter scientific literature to put their attention only on articles that are generating interest. Some authors have suggested that altmetrics can be used as another measure of research impact, and can sometimes predict whether an article will be become highly cited [4, 7, 11, 26–28]. However, altmetric data must be carefully interpreted, as the role of social media activity to predict citation rates is still controversial. Some authors found a weak [3, 5, 10, 29] or no relationship between the number of tweets and the number of citations an article received [30]. Peoples and colleagues suggest that altmetrics and traditional metrics are closely related, but not identical [7]. For example, they found a strong positive relationship between Twitter activity and number of citations, but this activity was not driven by journal impact factor. A review of the 2015 altmetric top 100 articles did not demonstrate a clear link between social media activity and number of citations [31].

### Strengths and limitations

One strength of our work was the use of a large sample size of publications following an *a* *priori* protocol involving a systematic search, filtering, data extraction, and analysis. The data and scripts used in this paper have been made available on-line to facilitate reproduction of our results. In addition, we have used AMSTAR, the most universally used tool, for the evaluation of the methodological quality of the articles.

The present study, however, also had several limitations that placed constraints on its generalizability. First, our study focused on psoriasis, and specifically on SRs and MAs of this topic. We do not know whether our results would be widely applicable to other classes of study or topics of research. Therefore, the next step would involve of generating of stronger overall evidence to support our findings regarding the influence of the author-paper affiliation network structure on variations in scientific productivity and quality in other areas of research. Second, there was no altmetric data for a large number (approximately 25%) of SRs about psoriasis. Third, papers need time to accumulate citations, thus it is well understood that considering the years 2015 and 2016 would be a negative predictor of the number of cites. Fourth, since the quality and consistency of the measurements vary between different providers of altmetrics, our results must be considered in the context of the tool used in this regard [32]. Fifth, it is should be mentioned that the OR for the number of Mendeley readers better predicted the number of cites in Google Scholar only indicates a small difference, and this result, although with an 95%CI of 1.018-10.049, should be interpreted with caution. Sixth, AMSTAR tool was designed so that all the items have the same value. Many investigators consider this system imprecise and suggest major weight to items more important. However, in the literature both the sum or the percentage of items have been used as a measure of the quality of the report. In our case, using total score for map reducing allowed us to simplify tagging reviews to perform more sophisticated analyses. Finally, among the limitations of the work are those related to AMSTAR as tool for the methodological quality evaluation, including the inclusion of related aspects in the notification of systematic reviews.

### Conclusions and future research

In summary, the social diffusion and the number of citations is not related to the methodological quality of SRs on psoriasis. Given that the greater number of readers is related to the number of citations and that the impact factor leads to greater diffusion, it would be desirable for the journal editors to incorporate the quality assessment tools of SRs to filter reviews of lower methodological rigor, regardless of the direction or significance of results and conflicts of interest of the article. It would also help to dissociate the concepts of bibliometric impact with scientific quality of research. Because the nature of this work limits its extrapolation to other areas of scientific research, future studies that replicate our results would be desirable.

## Supporting information

S1 File List of included studies (Table A), list of non included studies (Table B), AMSTAR checklist (Table C), PROSPERO register file (Table D), scree plot of multifactorial analysis (Fig A), group representation (Fig B). (DOC)
