# Supplementary material for: The differential impact of scientific quality, bibliometric factors, and social media activity on the influence of systematic reviews and meta-analyses about psoriasis
Source: PLoS One. 2018 Jan 29;13(1):e0191124. doi: 10.1371/journal.pone.0191124 (PMC5788350; doi:10.1371/journal.pone.0191124)
Supplement: S1 File — (DOC) [file pone.0191124.s001.doc]

**SUPPLEMENTARY MATERIAL**

**Title:** The differential impact of scientific quality, bibliometric factors, and social media activity on the influence of systematic reviews and meta-analyses about psoriasis

**Authors**: J. Ruano; M. Aguilar-Luque; F. Gómez-García; P. Alcalde-Mellado; J. Gay-Mimbrera; P. J. Carmona-Fernández; B. Maestre-Lopez; J.L. Sanz-Cabanillas; J.L. Hernández-Romero; M. González-Padilla; A. Vélez García-Nieto; B. Isla-Tejera.

**Content:**

**1. Supplementary Tables.**

**Table A.** List of included studies.

**Table B.** List of non included studies.

**Table C.** PROSPERO register file.

**Table D.** AMSTAR checklist.

**2. Supplementary Figures.**

**Figure A.** Graph showing the *scree test* results.

**Figure B.** PC1-PC2 projections of variable groups.

**1. Supplementary Tables.**

**Table A.** List of included studies.

**2017**

1. Ali FM, Cueva AC, Vyas J, Atwan AA, Salek MS, Finlay AY, Piguet V. A systematic review of the use of quality-of-life instruments in randomized controlled trials for psoriasis. Br J Dermatol 2017;176:577-93. doi: 10.1111/bjd.14788.
2. Brown G, Wang E, Leon A, Huynh M, Wehner M, Matro R, Linos E, Liao W, Haemel A. Tumor necrosis factor-α inhibitor-induced psoriasis: Systematic review of clinical features, histopathological findings, and management experience. J Am Acad Dermatol 2017;76:334-341. doi: 10.1016/j.jaad.2016.08.012.
3. de Carvalho AV, Duquia RP, Horta BL, Bonamigo RR. Efficacy of Immunobiologic and Small Molecule Inhibitor Drugs for Psoriasis: A Systematic Review and Meta-Analysis of Randomized Clinical Trials. Drugs R D 2017;17:29-51.
4. Gómez-García F, Epstein D, Isla-Tejera B, Lorente A, Vélez García-Nieto A, Ruano J. Short-term efficacy and safety of new biological agents targeting the interleukin-23-T helper 17 pathway for moderate-to-severe plaque psoriasis: a systematic review and network meta-analysis. Br J Dermatol 2017;176:594-603. doi: 10.1111/bjd.14814.
5. Puig L, Thom H, Mollon P, Tian H, Ramakrishna GS. Clear or almost clear skin improves the quality of life in patients with moderate-to-severe psoriasis: a systematic review and meta-analysis. J Eur Acad Dermatol Venereol 2017;31:213-220. doi: 10.1111/jdv.14007.

**2016**

1. Belinchón I, Rivera R, Blanch C, Comellas M, Lizán L. Adherence, satisfaction and preferences for treatment in patients with psoriasis in the European Union: a systematic review of the literature. Patient Prefer Adherence 2016 Nov;10:2357-2367. ECollection 2016.
2. Chen HQ, Li X, Tang R. Effects of Narrow Band Ultraviolet B on Serum Levels of Vascular Endothelial Growth Factor and Interleukin-8 in Patients with Psoriasis Am J Ther 2016;23:e655-62. doi: 10.1097/MJT.0000000000000330.
3. de Carvalho AV, Duquia RP, Horta BL, Bonamigo RR. Efficacy of Immunobiologic and Small Molecule Inhibitor Drugs for Psoriasis: A Systematic Review and Meta-Analysis of Randomized Clinical Trials. Drugs R D 2017;17:29-51. doi: 10.1007/s40268-016-0152-x.
4. De Vecchis R, Baldi C, Palmisani L. Protective effects of methotrexate against ischemic cardiovascular disorders in patients treated for rheumatoid arthritis or psoriasis: novel therapeutic insights coming from a meta-analysis of the literature data. Anatol J Cardiol 2016;16:2-9. doi: 10.5152/akd.2015.6136.
5. Desai RJ, Thaler KJ, Mahlknecht P, Gartlehner G, McDonagh MS, Mesgarpour B, Mazinanian A, Glechner A, Gopalakrishnan C, Hansen RA. Comparative Risk of Harm Associated With the Use of Targeted Immunomodulators: A Systematic Review. Arthritis Care Res (Hoboken) 2016;68:1078-88. doi: 10.1002/acr.22815.
6. Gutknecht M, Schaarschmidt ML, Herrlein O, Augustin M. A systematic review on methods used to evaluate patient preferences in psoriasis treatments. J Eur Acad Dermatol Venereol 2016;30:1454-64. doi: 10.1111/jdv.13749.
7. Jacobs I, Petersel D, Isakov L, Lula S, Lea Sewell K. Biosimilars for the Treatment of Chronic Inflammatory Diseases: A Systematic Review of Published Evidence. BioDrugs 2016;30:525-70.
8. Obradors M, Blanch C, Comellas M, Figueras M, Lizan L. Health-related quality of life in patients with psoriasis: a systematic review of the European literature. Qual Life Res 2016;25:2739-54.
9. Pickett K, Frampton G, Loveman E. Education to improve quality of life of people with chronic inflammatory skin conditions: a systematic review of the evidence. British Journal of Dermatology 2016;174:1228-41.
10. Ryoo JY, Yang HJ, Ji E, Yoo BK. Meta-analysis of the Efficacy and Safety of Secukinumab for the Treatment of Plaque Psoriasis. Ann Pharmacother 2016;50:341-51. doi: 10.1177/1060028015626545.
11. Schlager JG, Rosumeck S, Werner RN, Jacobs A, Schmitt J, Schlager C, et al. Topical treatments for scalp psoriasisCochrane Database of Systematic Reviews 2016:2:CD009687.
12. Shelton E, Laharie D, Scott FI, Mamtani R, Lewis JD, Colombel JF, Ananthakrishnan AN. Cancer Recurrence Following Immune-Suppressive Therapies in Patients With Immune-Mediated Diseases: A Systematic Review and Meta-analysis. Gastroenterology 2016;151:97-109.e4. doi: 10.1053/j.gastro.2016.03.037.
13. Svendsen MT, Jeyabalan J, Andersen KE, Andersen F, Johannessen H. Worldwide utilization of topical remedies in treatment of psoriasis: a systematic review. J Dermatolog Treat 2016;13:1-10.
14. Teixeira A, Teixeira M, Almeida V, Torres T, Sousa Lobo JM, Almeida IF. Methodologies for medication adherence evaluation: Focus on psoriasis topical treatment. Journal of Dermatological Science 2016;82:63-8.
15. Vaughn AR, Branum A, Sivamani RK. Effects of Turmeric (Curcuma longa) on Skin Health: A Systematic Review of the Clinical Evidence. Phytother Res 2016;30:1243-64. doi: 10.1002/ptr.5640.
16. Wang J, Zhan Q, Zhang L. A systematic review on the efficacy and safety of Infliximab in patients with psoriasis. Hum Vaccin Immunother 2016;12:431-37.
17. West J, Ogston S, Foerster J. Safety and efficacy of methotrexate in psoriasis: A meta-analysis of published trials. PLoS ONE 2016;11:5: e0153740.
18. Yamauchi PS, Bissonnette R, Teixeira HD, Valdecantos WC. Systematic review of efficacy of anti-tumor necrosis factor (TNF) therapy in patients with psoriasis previously treated with a different anti-TNF agent. J Am Acad Dermatol 2016;75:612-618.e6. doi: 10.1016/j.jaad.2016.02.1221.
19. Yan R, Jiang S, Wu Y, Gao XH, Chen HD. Topical calcipotriol/betamethasone dipropionate for psoriasis vulgaris: A systematic review.Indian Journal of Dermatology, Venereology and Leprology 2016;82:135-44.
20. Yang ZS, Lin NN, Li L, Li Y. The Effect of TNF Inhibitors on Cardiovascular Events in Psoriasis and Psoriatic Arthritis: an Updated Meta-Analysis Clinical Reviews in Allergy and Immunology 2016;(1-8).
21. Yiu ZZ, Exton LS, Jabbar-Lopez Z, Mohd Mustapa MF, Samarasekera EJ, Burden AD, Murphy R, Owen CM, Parslew R, Venning V, Ashcroft DM, Griffiths CE, Smith CH, Warren RB. Risk of Serious Infections in Patients with Psoriasis on Biologic Therapies: A Systematic Review and Meta-Analysis. J Invest Dermatol 2016;136:1584-91. doi: 10.1016/j.jid.2016.03.035.
22. Zhang CS, Yang L, Zhang AL, May BH, Yu JJ, Guo X, et al. Is Oral Chinese Herbal Medicine Beneficial for Psoriasis Vulgaris? A Meta-Analysis of Comparisons with Acitretin Journal of Alternative and Complementary Medicine 2016;22:174-88.

**2015**

1. Almutawa F, Thalib L, Hekman D, Sun Q, Hamzavi I, Lim HW. Efficacy of localized phototherapy and photodynamic therapy for psoriasis: a systematic review and meta-analysis. Photodermatol Photoimmunol Photomed 2015;31:5-14. doi: 10.1111/phpp.12092.
2. Atwan A, Ingram JR, Abbott R, Kelson MJ, Pickles T, Bauer A, Piguet V. Oral fumaric acid esters for psoriasis: abridged Cochrane systematic review including GRADE assessments. Br J Dermatol 2016;175:873-881. doi: 10.1111/bjd.14676.
3. Chen Y, Qian T, Zhang D, Yan H, Hao F. Clinical efficacy and safety of anti-IL-17 agents for the treatment of patients with psoriasis. Immunotherapy 2015;7:1023-37. doi: 10.2217/imt.15.50.
4. Conway R, Low C, Coughlan RJ, O'Donnell MJ, Carey JJ. Methotrexate use and risk of lung disease in psoriasis, psoriatic arthritis, and inflammatory bowel disease: systematic literature review and meta-analysis of randomised controlled trials. BMJ 2015;13:h1269. doi: 10.1136/bmj.h1269.
5. Coyle M, Deng J, Zhang AL, Yu J, Guo X, Xue CC, et al. Acupuncture therapies for psoriasis vulgaris: a systematic review of randomized controlled trials. Forsch Komplementmed 2015;22:102-9. doi: 10.1159/000381225.
6. Dannepond C, Maruani A, Machet L, Ternant D, Paintaud G, Samimi M. Serum infliximab concentrations in psoriatic patients treated with infliximab: a systematic review. Acta Derm Venereol 2015;95:401-6. doi: 10.2340/00015555-1980.
7. Depont F, Berenbaum F, Filippi J, Le Maitre M, Nataf H, Paul C, Peyrin-Biroulet L, Thibout E. Interventions to Improve Adherence in Patients with Immune-Mediated Inflammatory Disorders: A Systematic Review. PLoS One 2015;10:e0145076. doi: 10.1371/journal.pone.0145076. ECollection 2015.
8. Fleming P, Roubille C, Richer V, Starnino T, McCourt C, McFarlane A, et al. Effect of biologics on depressive symptoms in patients with psoriasis: a systematic review. J Eur Acad Dermatol Venereol 2015;29:1063-70. doi: 10.1111/jdv.12909.
9. Jacobi A, Mayer A, Augustin M. Keratolytics and emollients and their role in the therapy of psoriasis: a systematic review. Dermatol Ther (Heidelb) 2015;5(1):1-18.
10. Jacobs A, Rosumeck S, Nast A. Systematic review on the maintenance of response during systemic antipsoriatic therapy. Br J Dermatol 2015;173:910-21. doi: 10.1111/bjd.14077.
11. Miroddi M, Navarra M, Calapai F, Mancari F, Giofrè SV, Gangemi S, Calapai G. Review of Clinical Pharmacology of Aloe vera L. in the Treatment of Psoriasis. Phytother Res 2015;29:648-55. doi: 10.1002/ptr.5316
12. Nast A, Jacobs A, Rosumeck S, Werner RN. Efficacy and Safety of Systemic Long-Term Treatments for Moderate-to-Severe Psoriasis: A Systematic Review and Meta-Analysis. J Invest Dermatol 2015;135:2641-8. doi: 10.1038/jid.2015.206.
13. Nast A, Rosumeck S, Seidenschnur K. Biosimilars: a systematic review of published and ongoing clinical trials of antipsoriatics in chronic inflammatory diseases. J Dtsch Dermatol Ges 2015;13:294-300. doi: 10.1111/ddg.12621.
14. Pickett K, Loveman E, Kalita N, Frampton GK, Jones J. Educational interventions to improve quality of life in people with chronic inflammatory skin diseases: systematic reviews of clinical effectiveness and cost-effectiveness. Health Technol Assess 2015;19:1-176, v-vi. doi: 10.3310/hta19860.
15. Rachakonda TD, Dhillon JS, Florek AG, Armstrong AW. Effect of tonsillectomy on psoriasis: a systematic review. J Am Acad Dermatol 2015;72:261-75.
16. Roubille C, Richer V, Starnino T, McCourt C, McFarlane A, Fleming P, et al. The effects of tumour necrosis factor inhibitors, methotrexate, non-steroidal anti-inflammatory drugs and corticosteroids on cardiovascular events in rheumatoid arthritis, psoriasis and psoriatic arthritis: a systematic review and meta-analysis. Ann Rheum Dis 2015;74:480-9. doi: 10.1136/annrheumdis-2014-206624.
17. Sanclemente G, Murphy R, Contreras J, García H, Bonfill Cosp X. Anti-TNF agents for paediatric psoriasis. Cochrane Database Syst Rev. 2015:CD010017. doi: 10.1002/14651858.CD010017.pub2.
18. Signorovitch JE, Betts KA, Yan YS, LeReun C, Sundaram M, Wu EQ, et al. Comparative efficacy of biological treatments for moderate-to-severe psoriasis: a network meta-analysis adjusting for cross-trial differences in reference arm response. Br J Dermatol 2015;172:504-12. doi: 10.1111/bjd.13437.
19. Upala S, Sanguankeo A. Effect of lifestyle weight loss intervention on disease severity in patients with psoriasis: a systematic review and meta-analysis. Int J Obes (Lond). 2015;39:1197-202. doi: 10.1038/ijo.2015.64.
20. Vangeli E, Bakhshi S, Baker A, Fisher A, Bucknor D, Mrowietz U, Östör AJ, Peyrin-Biroulet L, Lacerda AP, Weinman J. A Systematic Review of Factors Associated with Non-Adherence to Treatment for Immune-Mediated Inflammatory Diseases. Adv Ther 2015;32:983-1028. doi: 10.1007/s12325-015-0256-7.
21. Yang L, Zhang CS, May B, Yu J, Guo X, Zhang AL, Xue CC, Lu C. Efficacy of combining oral Chinese herbal medicine and NB-UVB in treating psoriasis vulgaris: a systematic review and meta-analysis. Chin Med 2015;10:27. doi: 10.1186/s13020-015-0060-y. eCollection 2015.

**2014**

1. Augustin M, Mrowietz U, Bonnekoh B, Rosenbach T, Thaçi D, Reusch M, et al. Topical long-term therapy of psoriasis with vitamin D₃ analogues, corticosteroids and their two compound formulations: position paper on evidence and use in daily practice. J Dtsch Dermatol Ges 2014;12:667-82. doi: 10.1111/ddg.12396.
2. Boehncke WH, Alvarez Martinez D, Solomon JA, Gottlieb AB. Safety and efficacy of therapies for skin symptoms of psoriasis in patients with psoriatic arthritis: a systematic review. J Rheumatol 2014;41:2301-5. doi: 10.3899/jrheum.140880
3. Busard C, Zweegers J, Limpens J, Langendam M, Spuls PI. Combined use of systemic agents for psoriasis: a systematic review. JAMA Dermatol 2014;150:1213-20. Doi: 10.1001/jamadermatol.2014.1111.
4. Deng S, May BH, Zhang AL, Lu C, Xue CC. Topical herbal formulae in the management of psoriasis: systematic review with meta-analysis of clinical studies and investigation of the pharmacological actions of the main herbs. Phytother Res 2014;28:480-97. doi: 10.1002/ptr.5028.
5. Gupta AK, Daigle D, Lyons DC. Network Meta-analysis of Treatments for Chronic Plaque Psoriasis in Canada. J Cutan Med Surg 2014;18:371-8.
6. Hsu L, Snodgrass BT, Armstrong AW. Antidrug antibodies in psoriasis: a systematic review. Br J Dermatol 2014;170:261-73. doi: 10.1111/bjd.12654.
7. Larsen MH, Hagen KB, Krogstad AL, Aas E, Wahl AK. Limited evidence of the effects of patient education and self-management interventions in psoriasis patients: a systematic review. Patient Educ Couns 2014;94:158-69. doi: 10.1016/j.pec.2013.10.005.
8. Mattei PL, Corey KC, Kimball AB. Psoriasis Area Severity Index (PASI) and the Dermatology Life Quality Index (DLQI): the correlation between disease severity and psychological burden in patients treated with biological therapies. J Eur Acad Dermatol Venereol 2014;28:333-7. doi: 10.1111/jdv.12106.
9. Maybury CM, Jabbar-Lopez ZK, Wong T, Dhillon AP, Barker JN, Smith CH. Methotrexate and liver fibrosis in people with psoriasis: a systematic review of observational studies. Br J Dermatol 2014;171:17-29. doi: 10.1111/bjd.12941.
10. Meng Y, Dongmei L, Yanbin P, Jinju F, Meile T, Binzhu L, et al. Systematic review and meta-analysis of ustekinumab for moderate to severe psoriasis. Clin Exp Dermatol 2014;39:696-707. doi: 10.1111/ced.12390
11. Posso-De Los Rios CJ, Pope E, Lara-Corrales I. A systematic review of systemic medications for pustular psoriasis in pediatrics. Pediatr Dermatol 2014;31:430-9. doi: 10.1111/pde.12351.
12. Puig L, López A, Vilarrasa E, García I. Efficacy of biologics in the treatment of moderate-to-severe plaque psoriasis: a systematic review and meta-analysis of randomized controlled trials with different time points. J Eur Acad Dermatol Venereol 2014;28:1633-53. doi: 10.1111/jdv.12238.
13. Sandoval LF, Pierce A, Feldman SR. Systemic therapies for psoriasis: an evidence-based update. Am J Clin Dermatol 2014;15:165-80. doi: 10.1007/s40257-014-0064-x.
14. Schmitt J, Rosumeck S, Thomaschewski G, Sporbeck B, Haufe E, Nast A. Efficacy and safety of systemic treatments for moderate-to-severe psoriasis: meta-analysis of randomized controlled trials.Br J Dermatol 2014;170:274-303. doi: 10.1111/bjd.12663.
15. Sevrain M, Richard MA, Barnetche T, Rouzaud M, Villani AP, Paul C, et al. Treatment for palmoplantar pustular psoriasis: systematic literature review, evidence-based recommendations and expert opinion. J Eur Acad Dermatol Venereol 2014;28 Suppl 5:13-6. doi: 10.1111/jdv.12561.
16. Zhang CS, Yu JJ, Parker S, Zhang AL, May B, Lu C, et al. Oral Chinese herbal medicine combined with pharmacotherapy for psoriasis vulgaris: a systematic review. Int J Dermatol 2014;53:1305-18. doi: 10.1111/ijd.12607.
17. Zhou D, Chen W, Li X, et al. Evidence-based practice guideline of Chinese herbal medicine for psoriasis vulgaris (Bai Bi) Eur J Integr Med 2014;6:135-46.

**2013**

1. Almutawa F, Alnomair N, Wang Y, Hamzavi I, Lim HW. Systematic review of UV-based therapy for psoriasis. Am J Clin Dermatol 2013;14:87-109. doi: 10.1007/s40257-013-0015-y.
2. Chen X, Yang M, Cheng Y, Liu GJ, Zhang M. Narrow-band ultraviolet B phototherapy versus broad-band ultraviolet B or psoralen-ultraviolet A photochemotherapy for psoriasis. Cochrane Database Syst Rev 2013:CD009481. doi: 10.1002/14651858.CD009481.pub2.
3. Correr CJ, Rotta I, Teles T de S, Godoy RR, Riveros BS, Garcia MM, et al. Efficacy and safety of biologics in the treatment of moderate to severe psoriasis: a comprehensive meta-analysis of randomized controlled trials. Cad Saude Publica 2013;29 Suppl 1:S17-31.
4. de Vries AC, Bogaards NA, Hooft L, Velema M, Pasch M, Lebwohl M, Spuls PI. Interventions for nail psoriasis. Cochrane Database Syst Rev 2013;31:CD007633. doi: 10.1002/14651858.CD007633.pub2.
5. Deng S, May BH, Zhang AL, Lu C, Xue CC. Plant extracts for the topical management of psoriasis: a systematic review and meta-analysis. Br J Dermatol 2013;169:769-82. doi: 10.1111/bjd.12557.
6. Deng S, May BH, Zhang AL, Lu C, Xue CC. Plant extracts for the topical management of psoriasis: a systematic review and meta-analysis. Br J Dermatol 2013;169:769-82. doi: 10.1111/bjd.12557.
7. Deng S, May BH, Zhang AL, Lu C, Xue CC. Topical herbal medicine combined with pharmacotherapy for psoriasis: a systematic review and meta-analysis. Arch Dermatol Res 2013 Apr;305(3):179-89. doi: 10.1007/s00403-013-1316-y.
8. Erceg A, de Jong EM, van de Kerkhof PC, Seyger MM. The efficacy of pulsed dye laser treatment for inflammatory skin diseases: a systematic review. J Am Acad Dermatol 2013;69:609-615.e8. doi: 10.1016/j.jaad.2013.03.029.
9. Galván-Banqueri M, Marín Gil R, Santos Ramos B, Bautista Paloma FJ. Biological treatments for moderate-to-severe psoriasis: indirect comparison. J Clin Pharm Ther 2013;38:121-30. doi: 10.1111/jcpt.12044.
10. Garcês S, Demengeot J, Benito-Garcia E. The immunogenicity of anti-TNF therapy in immune-mediated inflammatory diseases: a systematic review of the literature with a meta-analysis. Ann Rheum Dis 2013;72:1947-55. doi: 10.1136/annrheumdis-2012-202220.
11. Hendriks AG, Keijsers RR, de Jong EM, Seyger MM, van de Kerkhof PC. Combinations of classical time-honoured topicals in plaque psoriasis: a systematic review. J Eur Acad Dermatol Venereol 2013;27:399-410. doi: 10.1111/j.1468-3083.2012.04640.x.
12. Hendriks AG, Keijsers RR, de Jong EM, Seyger MM, van de Kerkhof PC. Efficacy and safety of combinations of first-line topical treatments in chronic plaque psoriasis: a systematic literature review. J Eur Acad Dermatol Venereol 2013;27:931-51. doi: 10.1111/jdv.12058.
13. Maneiro JR, Salgado E, Gomez-Reino JJ. Immunogenicity of monoclonal antibodies against tumor necrosis factor used in chronic immune-mediated Inflammatory conditions: systematic review and meta-analysis. JAMA Intern Med 2013;12;173:1416-28. doi: 10.1001/jamainternmed.2013.7430.
14. Mason A, Mason J, Cork M, Hancock H, Dooley G. Topical treatments for chronic plaque psoriasis: an abridged Cochrane systematic review. J Am Acad Dermatol 2013;69:799-807. doi: 10.1016/j.jaad.2013.06.027.
15. Mason AR, Mason J, Cork M, Dooley G, Hancock H. Topical treatments for chronic plaque psoriasis. Cochrane Database Syst Rev 2013; 28:CD005028. doi: 10.1002/14651858.CD005028.pub3.
16. Mason AR, Mason JM, Cork MJ, Hancock H, Dooley G. Topical treatments for chronic plaque psoriasis of the scalp: a systematic review. Br J Dermatol 2013;169:519-27. doi: 10.1111/bjd.12393.
17. Mustafa AA, Al-Hoqail IA. Biologic systemic therapy for moderate-to-severe psoriasis: A review. Journal of Taibah University Medical Sciences 2013;8:142-50.
18. Samarasekera EJ, Sawyer L, Wonderling D, Tucker R, Smith CH. Topical therapies for the treatment of plaque psoriasis: systematic review and network meta-analyses. Br J Dermatol 2013 May;168(5):954-67. doi: 10.1111/bjd.12276.
19. Strohal R, Chimenti S, Vena GA, Girolomoni G. Etanercept provides an effective, safe and flexible short- and long-term treatment regimen for moderate-to-severe psoriasis: a systematic review of current evidence. J Dermatolog Treat 2013;24:199-208. doi: 10.3109/09546634.2012.713462.
20. Thorlund K, Druyts E, Aviña-Zubieta JA, Mills EJ. Anti-tumor necrosis factor (TNF) drugs for the treatment of psoriatic arthritis: an indirect comparison meta-analysis. Biologics 2012;6:417-27. doi: 10.2147/BTT.S37606.
21. Thorneloe RJ, Bundy C, Griffiths CE, Ashcroft DM, Cordingley L. Adherence to medication in patients with psoriasis: a systematic literature review. Br J Dermatol 2013;168:20-31. doi: 10.1111/bjd.12039.
22. Yu JJ, Zhang CS, Zhang AL, May B, Xue CC, Lu C. Add-on effect of chinese herbal medicine bath to phototherapy for psoriasis vulgaris: a systematic review. Evid Based Complement Alternat Med 2013:673078. doi: 10.1155/2013/673078

**2012**

1. Archier E, Devaux S, Castela E, Gallini A, Aubin F, Le Maître M, et al. Carcinogenic risks of psoralen UV-A therapy and narrowband UV-B therapy in chronic plaque psoriasis: a systematic literature review. J Eur Acad Dermatol Venereol 2012;26 Suppl 3:22-31. doi: 10.1111/j.1468-3083.2012.04520.x.
2. Archier E, Devaux S, Castela E, Gallini A, Aubin F, Le Maître M, et al. Efficacy of psoralen UV-A therapy vs. narrowband UV-B therapy in chronic plaque psoriasis: a systematic literature review. J Eur Acad Dermatol Venereol 2012;26 Suppl 3:11-21. doi: 10.1111/j.1468-3083.2012.04519.x.
3. Archier E, Devaux S, Castela E, Gallini A, Aubin F, Le Maître M, et al. Ocular damage in patients with psoriasis treated by psoralen UV-A therapy or narrow band UVB therapy: a systematic literature review. J Eur Acad Dermatol Venereol 2012;26 Suppl 3:32-5. doi: 10.1111/j.1468-3083.2012.04521.x.
4. Bailey EE, Ference EH, Alikhan A, Hession MT, Armstrong AW. Combination treatments for psoriasis: a systematic review and meta-analysis. Arch Dermatol 2012;148:511-22. doi: 10.1001/archdermatol.2011.1916.
5. Brezinski EA, Armstrong AW. Off-label biologic regimens in psoriasis: a systematic review of efficacy and safety of dose escalation, reduction, and interrupted biologic therapy. PLoS One. 2012;7(4):e33486. doi: 10.1371/journal.pone.0033486. 1.
6. Castela E, Archier E, Devaux S, Gallini A, Aractingi S, Cribier B, et al. Topical corticosteroids in plaque psoriasis: a systematic review of efficacy and treatment modalities. J Eur Acad Dermatol Venereol 2012;26 Suppl 3:36-46. doi: 10.1111/j.1468-3083.2012.04522.x.
7. Castela E, Archier E, Devaux S, Gallini A, Aractingi S, Cribier B, et al. Topical corticosteroids in plaque psoriasis: a systematic review of risk of adrenal axis suppression and skin atrophy. J Eur Acad Dermatol Venereol 2012;26 Suppl 3:47-51. doi: 10.1111/j.1468-3083.2012.04523.x.
8. Devaux S, Castela A, Archier E, Gallini A, Joly P, Misery L, Aractingi S, Aubin F, Bachelez H, Cribier B, Jullien D, Le Maître M, Richard MA, Ortonne JP, Paul C. Adherence to topical treatment in psoriasis: a systematic literature review. J Eur Acad Dermatol Venereol 2012;26 Suppl 3:61-7. doi: 10.1111/j.1468-3083.2012.04525.x. Review.
9. Devaux S, Castela A, Archier E, Gallini A, Joly P, Misery L, et al. Topical vitamin D analogues alone or in association with topical steroids for psoriasis: a systematic review. J Eur Acad Dermatol Venereol 2012;Suppl 3:52-60. doi: 10.1111/j.1468-3083.2012.04524.x.
10. Kim IH, West CE, Kwatra SG, Feldman SR, O'Neill JL. Comparative efficacy of biologics in psoriasis: a review. Am J Clin Dermatol 2012;13:365-74. doi: 10.2165/11633110-000000000-00000.
11. Lamel SA, Myer KA, Younes N, Zhou JA, Maibach H, Maibach HI. Placebo response in relation to clinical trial design: a systematic review and meta-analysis of randomized controlled trials for determining biologic efficacy in psoriasis treatment. Arch Dermatol Res 2012;304:707-17. doi: 10.1007/s00403-012-1266-9.
12. Li N, Li YQ, Li HY, Guo W, Bai YP. Efficacy of externally applied Chinese herbal drugs in treating psoriasis: a systematic review. Chin J Integr Med 2012;18(3):222-9. doi: 10.1007/s11655-012-1004-3.
13. Lin VW, Ringold S, Devine EB. Comparison of Ustekinumab With Other Biological Agents for the Treatment of Moderate to Severe Plaque Psoriasis: A Bayesian Network Meta-analysis. Arch Dermatol 2012;148:1403-10. doi: 10.1001/2013.jamadermatol.238.
14. Lucka TC, Pathirana D, Sammain A, Bachmann F, Rosumeck S, Erdmann R, et al. Efficacy of systemic therapies for moderate-to-severe psoriasis: a systematic review and meta-analysis of long-term treatment. J Eur Acad Dermatol Venereol 2012;26:1331-44. doi: 10.1111/j.1468-3083.2012.04492.x.
15. Malhotra A, Shafiq N, Rajagopalan S, Dogra S, Malhotra S. Thiazolidinediones for plaque psoriasis: a systematic review and meta-analysis. Evid Based Med. 2012;17:171-6. doi: 10.1136/ebmed-2011-100388.
16. Paul C, Gallini A, Archier E, Castela E, Devaux S, Aractingi S, et al. Evidence-based recommendations on topical treatment and phototherapy of psoriasis: systematic review and expert opinion of a panel of dermatologists. J Eur Acad Dermatol Venereol 2012;26 Suppl 3:1-10. doi: 10.1111/j.1468-3083.2012.04518.x.
17. Reich K, Burden AD, Eaton JN, Hawkins NS. Efficacy of biologics in the treatment of moderate to severe psoriasis: a network meta-analysis of randomized controlled trials. Br J Dermatol 2012;166(1):179-88. doi: 10.1111/j.1365-2133.2011.10583.x.
18. Umar N, Yamamoto S, Loerbroks A, Terris D. Elicitation and use of patients' preferences in the treatment of psoriasis: a systematic review. Acta Derm Venereol 2012;92:341-6. doi: 10.2340/00015555-1304.

**2011**

1. Atwan A, Ingram JR, Abbott R, Kelson MJ, Pickles T, Bauer A, Piguet V. Oral fumaric acid esters for psoriasis: abridged Cochrane systematic review including GRADE assessments. Br J Dermatol 2016;175:873-81. doi: 10.1111/bjd.14676.
2. Bottomley JM, Taylor RS, Ryttov J. The effectiveness of two-compound formulation calcipotriol and betamethasone dipropionate gel in the treatment of moderately severe scalp psoriasis: a systematic review of direct and indirect evidence. Curr Med Res Opin 2011;27:251-68. doi: 10.1185/03007995.2010.541022.
3. Dommasch ED, Abuabara K, Shin DB, Nguyen J, Troxel AB, Gelfand JM. The risk of infection and malignancy with tumor necrosis factor antagonists in adults with psoriatic disease: a systematic review and meta-analysis of randomized controlled trials. J Am Acad Dermatol 2011;64:1035-50. doi: 10.1016/j.jaad.2010.09.734.
4. Langham S, Langham J, Goertz HP, Ratcliffe M. Large-scale, prospective, observational studies in patients with psoriasis and psoriatic arthritis: A systematic and critical review. BMC Med Res Methodol 2011;11:32. doi: 10.1186/1471-2288-11-32.
5. Montaudié H, Sbidian E, Paul C, Maza A, Gallini A, Aractingi S, et al. Methotrexate in psoriasis: a systematic review of treatment modalities, incidence, risk factors and monitoring of liver toxicity. J Eur Acad Dermatol Venereol 2011;25 Suppl 2:12-8. doi: 10.1111/j.1468-3083.2011.03991.x.
6. Ryan C, Leonardi CL, Krueger JG, Kimball AB, Strober BE, Gordon KB, et al. Association between biologic therapies for chronic plaque psoriasis and cardiovascular events: a meta-analysis of randomized controlled trials. JAMA 2011;306:864-71. doi: 10.1001/jama.2011.1211.
7. Maza A, Montaudié H, Sbidian E, Gallini A, Aractingi S, Aubin F, et al. Oral cyclosporin in psoriasis: a systematic review on treatment modalities, risk of kidney toxicity and evidence for use in non-plaque psoriasis. J Eur Acad Dermatol Venereol 2011;25 Suppl 2:19-27. doi: 10.1111/j.1468-3083.2011.03992.x.
8. Sbidian E, Maza A, Montaudié H, Gallini A, Aractingi S, Aubin F, et al. Efficacy and safety of oral retinoids in different psoriasis subtypes: a systematic literature review. J Eur Acad Dermatol Venereol 2011;25 Suppl 2:28-33. doi: 10.1111/j.1468-3083.2011.03993.x.
9. Tan JY, Li S, Yang K, Ma B, Chen W, Zha C, Zhang J. Ustekinumab, a human interleukin-12/23 monoclonal antibody, in patients with psoriasis: a meta-analysis. J Dermatolog Treat 2011;22:323-36. Doi: 10.3109/09546634.2010.487890.

**2010**

1. de Jager ME, de Jong EM, van de Kerkhof PC, Seyger MM. Efficacy and safety of treatments for childhood psoriasis: a systematic literature review. J Am Acad Dermatol 2010;62:1013-30. doi: 10.1016/j.jaad.2009.06.048.

**2009**

1. Bansback N, Sizto S, Sun H, Feldman S, Willian MK, Anis A. Efficacy of systemic treatments for moderate to severe plaque psoriasis: systematic review and meta-analysis. Dermatology 2009;219:209-18. doi: 10.1159/000233234.
2. Feldman SR, Yentzer BA. Topical clobetasol propionate in the treatment of psoriasis: a review of newer formulations. Am J Clin Dermatol 2009;10:397-406. doi: 10.2165/11311020-000000000-00000.
3. Naldi L, Rzany B. Psoriasis (chronic plaque). BMJ Clin Evid 2009. pii: 1706.
4. Smith N, Weymann A, Tausk FA, Gelfand JM. Complementary and alternative medicine for psoriasis: a qualitative review of the clinical trial literature. J Am Acad Dermatol 2009;61(5):841-56. doi: 10.1016/j.jaad.2009.04.029.
5. Prey S, Paul C. Effect of folic or folinic acid supplementation on methotrexate-associated safety and efficacy in inflammatory disease: a systematic review. Br J Dermatol 2009;160:622-8. doi: 10.1111/j.1365-2133.2008.08876.x.

**2008**

1. Brimhall AK, King LN, Licciardone JC, Jacobe H, Menter A. Safety and efficacy of alefacept, efalizumab, etanercept and infliximab in treating moderate to severe plaque psoriasis: a meta-analysis of randomized controlled trials. Br J Dermatol 2008;159:274-85. doi: 10.1111/j.1365-2133.2008.08673.x.
2. Schmitt J, Zhang Z, Wozel G, Meurer M, Kirch W. Efficacy and tolerability of biologic and nonbiologic systemic treatments for moderate-to-severe psoriasis: meta-analysis of randomized controlled trials. Br J Dermatol 2008;159:513-26. doi: 10.1111/j.1365-2133.2008.08732.x.

**2006**

1. Boehncke WH, Prinz J, Gottlieb AB. Biologic therapies for psoriasis. A systematic review. J Rheumatol 2006;33:1447-51.
2. Cassell S, Kavanaugh AF. Therapies for psoriatic nail disease. A systematic review. J Rheumatol 2006;33:1452-6.
3. Marsland AM, Chalmers RJ, Hollis S, Leonardi-Bee J, Griffiths CE. Interventions for chronic palmoplantar pustulosis. Cochrane Database Syst Rev 2006:CD001433.
4. Strober BE, Siu K, Menon K. Conventional systemic agents for psoriasis. A systematic review. J Rheumatol 2006;33:1442-6.
5. Woolacott N, Hawkins N, Mason A, Kainth A, Khadjesari Z, Vergel YB, Misso K, Light K, Chalmers R, Sculpher M, Riemsma R. Etanercept and efalizumab for the treatment of psoriasis: a systematic review. Health Technol Assess. 2006;10:1-233, i-iv.

**2003**

1. Bruner CR, Feldman SR, Ventrapragada M, Fleischer AB Jr. A systematic review of adverse effects associated with topical treatments for psoriasis. Dermatol Online J 2003;9:2.

**2002**

1. Heydendael VM, Spuls PI, Ten Berge IJ, Opmeer BC, Bos JD, de Rie MA. Cyclosporin trough levels: is monitoring necessary during short-term treatment in psoriasis? A systematic review and clinical data on trough levels. Br J Dermatol 2002;147:122-9.
2. Mason J, Mason AR, Cork MJ. Topical preparations for the treatment of psoriasis: a systematic review. Br J Dermatol 2002;146:351-64.

**2001**

1. Chalmers RJ, O'Sullivan T, Owen CM, Griffiths CE. A systematic review of treatments for guttate psoriasis. Br J Dermatol 2001;145:891-4.
2. Owen CM, Chalmers RJ, O'Sullivan T, Griffiths CE. A systematic review of antistreptococcal interventions for guttate and chronic plaque psoriasis. Br J Dermatol 2001;145:886-90.

**2000**

1. Ashcroft DM, Li Wan Po A, Williams HC, Griffiths CE. Combination regimens of topical calcipotriene in chronic plaque psoriasis: systematic review of efficacy and tolerability. Arch Dermatol 2000;136:1536-43.
2. Ashcroft DM, Po AL, Williams HC, Griffiths CE. Systematic review of comparative efficacy and tolerability of calcipotriol in treating chronic plaque psoriasis. BMJ 2000;320:963-7.
3. Chalmers RJ, O'Sullivan T, Owen CM, Griffiths CE. Interventions for guttate psoriasis. Cochrane Database Syst Rev 2000;CD001213.
4. Griffiths CE, Clark CM, Chalmers RJ, Li Wan Po A, Williams HC. A systematic review of treatments for severe psoriasis. Health Technol Assess 2000;4:1-125.
5. Owen CM, Chalmers RJ, O'Sullivan T, Griffiths CE. Antistreptococcal interventions for guttate and chronic plaque psoriasis. Cochrane Database Syst Rev 2000:CD001976.

**1999**

1. Pasker-de Jong PC, Wielink G, van der Valk PG, van der Wilt GJ. Treatment with UV-B for psoriasis and nonmelanoma skin cancer: a systematic review of the literature. Arch Dermatol 1999;135:834-40.

**1997**

1. Spuls PI, Witkamp L, Bossuyt PM, Bos JD. A systematic review of five systemic treatments for severe psoriasis. Br J Dermatol 1997;137:943-9.

**Table B.** List of non included studies.

**2016**

1. Ahadieh S, Ito K, Wolk R, Valdez H, Tallman A, Krishnaswami S, et al. Model-based meta-analysis (MBMA) for evaluating time course of clinical response across psoriasis treatments. Clin. Pharmacol. Ther. . S. Ahadieh, Pfizer, Inc., Groton, United States; 2016;99:S52.

2. Ali F, Cueva A, Atwan A, Vyas J, Piguet V, Finlay A, et al. Can systematic reviews help with choosing a suitable health-related quality of life measure in interventional studies of psoriasis? Int. J. Clin. Pharm. . S. Salek, Department of Pharmacy, University of Hertfordshire, Hatfield, United Kingdom; 2016;38:565.

3. Armstrong A, Betts K, Li J, Sundaram M, Wu E, Signorovitch J. Numbers needed to treat and costs per responder for novel treatments of moderate to severe psoriasis. J. Am. Acad. Dermatol. . A. Armstrong, Keck School of Medicine, University of Southern California, Los Angeles, United States; 2016;74:AB261.

4. Atzmony L, Reiter O, Hodak E, Gdalevich M, Mimouni D. Treatments for Cutaneous Lichen Planus: A Systematic Review and Meta-Analysis. Am. J. Clin. Dermatol. . D. Mimouni, Department of Dermatology, Rabin Medical Center, Beilinson Hospital, Petach Tikva, Israel; 2016;17:11–22.

5. Bahrani E, Nunneley CE, Hsu S, Kass JS. Cutaneous Adverse Effects of Neurologic Medications. CNS Drugs . J.S. Kass, Department of Neurology, Baylor College of Medicine, Houston, United States; 2016;30:245–67.

6. Brambilla L, Brena M, Tourlaki A. Textiles in dermatology: Our experience and literature review. G. Ital. di Dermatologia e Venereol. . L. Brambilla, Operative Unit of Dermatology, IRCCS Ca’ Granda Foundation, Ospedale Maggiore Policlinico di Milano, Milan, Italy; 2016;151:266–74.

7. Bull L, Rayment M. HIV-indicator-condition-driven HIV testing: Clinically effective but still rarely implemented. Clin. Med. J. R. Coll. Physicians London . M. Rayment, Department of HIV/GU Medicine, Chelsea and Westminster Hospital NHS Foundation Trust, London, United Kingdom; 2016;16:175–9.

8. Burden-Teh E, Thomas KS, Ratib S, Grindlay D, Adaji E, Murphy R. The epidemiology of childhood psoriasis: A scoping review. Br. J. Dermatol. . E. Burden-Teh, Centre of Evidence Based Dermatology University of Nottingham Nottingham U.K.; 2016;

9. Carrascosa JM, De La Cueva P, Ara M, Puig L, Bordas X, Carretero G, et al. Methotrexate in Moderate to Severe Psoriasis: Review of the Literature and Expert Recommendations. Actas Dermosifiliogr. . J.M. Carrascosa, Servicio de Dermatología, Hospital Universitari Germans Trias i Pujol, Badalona, Barcelona, Spain; 2016;107:194–206.

10. Carstens E. Many parallels between itch and pain research. Eur. J. Pain (United Kingdom) . E. Carstens, Neurobiology, Physiology and Behavior, University of California, Davis, United States; 2016;20:5–7.

11. Checchio T, Ito K, Mandema J, Wolk R, Valdez H, Tan H, et al. A model based meta-analysis (MBMA) for comparative efficacy of psoriasis treatments: Dose-response model for psoriasis area and severity index (PASI) responses. Clin. Pharmacol. Ther. . T. Checchio, Pfizer, Inc., Groton, United States; 2016;99:S44–5.

12. Chen H-Q, Li X, Tang R. Effects of narrow band Ultraviolet B on serum levels of vascular endothelial growth factor and interleukin-8 in patients with psoriasis. Am. J. Ther. . H.-Q. Chen, Department of Dermatology, Affiliated Hospital of Qingdao University, Qingdao, China; 2016;23:e655–62.

13. Chen S-W, Zhong X-S, Jiang L-N, Zheng X-Y, Xiong Y-Q, Ma S-J, et al. Maternal autoimmune diseases and the risk of autism spectrum disorders in offspring: A systematic review and meta-analysis. Behav. Brain Res. . Q. Chen, Department of Epidemiology, School of Public Health and Tropical Medicine, Southern Medical University, Guangzhou, China; 2016;296:61–9.

14. Chen Y, Sun J, Yang Y, Huang Y, Liu G. Malignancy risk of anti-tumor necrosis factor alpha blockers: an overview of systematic reviews and meta-analyses. Clin. Rheumatol. . G. Liu, Department of Rheumatology and Immunology, West China Hospital, Sichuan University, Chengdu, China; 2016;35:1–18.

15. Chiricozzi A, Suárez-Fariñas M, Fuentes-Duculan J, Cueto I, Li K, Tian S, et al. Increased expression of interleukin-17 pathway genes in nonlesional skin of moderate-to-severe psoriasis vulgaris. Br. J. Dermatol. . A. Chiricozzi, Laboratory of Investigative Dermatology, Rockefeller University, New York City, United States; 2016;174:136–45.

16. Christakos S, Dhawan P, Verstuyf A, Verlinden L, Carmeliet G. Vitamin D: Metabolism, molecular mechanism of action, and pleiotropic effects. Physiol. Rev. . S. Christakos, Dept. of Microbiology,Biochemistry,and Molecular Genetics,Rutgers, The State University of New Jersey, New Jersey Medical School, Newark, United States; 2016;96:365–408.

17. Cobo-Ibáñez T, Villaverde V, Seoane-Mato D, Muñoz-Fernández S, Guerra M, del Campo PD, et al. Multidisciplinary dermatology–rheumatology management for patients with moderate-to-severe psoriasis and psoriatic arthritis: a systematic review. Rheumatol. Int. . T. Cobo-Ibáñez, Rheumatology Department, Hospital Universitario Infanta Sofía, San Sebastián de Los Reyes, Spain; 2016;36:221–9.

18. DiMarco G, Hill D, Feldman SR. Review of patient registries in dermatology. J. Am. Acad. Dermatol. . S.R. Feldman, Department of Dermatology, Wake Forest School of Medicine, Medical Center Blvd, Winston-Salem, NC 27157-1071; 2016;

19. Dowlatshahi EA, Wakkee M. Rising interest in the field of paediatric psoriasis. Br. J. Dermatol. . 2016;174:1180–1.

20. Elyoussfi S, Thomas BJ, Ciurtin C. Tailored treatment options for patients with psoriatic arthritis and psoriasis: review of established and new biologic and small molecule therapies. Rheumatol. Int. . C. Ciurtin, Department of Rheumatology, University College London Hospital, London, United Kingdom; 2016;36:603–12.

21. Fang N, Jiang M, Fan Y. Association between Psoriasis and Subclinical Atherosclerosis. Med. (United States) . Y. Fan, Institute of Molecular Biology and Translational Medicine, Affiliated People’s Hospital, Jiangsu University, ZhenJiang, China; 2016;95.

22. Farahnik B, Beroukhim K, Abrouk M, Nakamura M, Zhu TH, Singh R, et al. Brodalumab for the Treatment of Psoriasis: A Review of Phase III Trials. Dermatol. Ther. (Heidelb). . B. Farahnik, University of Vermont College of Medicine, Burlington, United States; 2016;6:111–24.

23. Farahnik B, Beroukhim K, Zhu TH, Abrouk M, Nakamura M, Singh R, et al. Ixekizumab for the Treatment of Psoriasis: A Review of Phase III Trials. Dermatol. Ther. (Heidelb). . B. Farahnik, University of Vermont College of Medicine, Burlington, United States; 2016;6:25–37.

24. Ferreira BIRC, Da Costa Abreu JLP, Dos Reis JPG, Da Costa Figueiredo AM. Psoriasis and associated psychiatric disorders: A systematic review on etiopathogenesis and clinical correlation. J. Clin. Aesthet. Dermatol. 2016;9:36–43.

25. Generali E, Scirè CA, Cantarini L, Selmi C. Sex differences in the treatment of Psoriatic Arthritis: A systematic literature review. Isr. Med. Assoc. J. . C. Selmi, Division of Rheumatology and Clinical Immunology, Humanitas Research Hospital, Rozzano, Italy; 2016;18:203–8.

26. Generali E, Scirè CA, Favalli EG, Selmi C. Biomarkers in psoriatic arthritis: a systematic literature review. Expert Rev. Clin. Immunol. . C. Selmi, Division of Rheumatology and Clinical Immunology, Humanitas Clinical and Research Center, Rozzano, Milan, Italy; 2016;12:651–60.

27. Gómez-García F, Epstein D, Ruano J. Network metaanalysis of new biologic agents targeting IL-23/Th17 pathway for moderate to severe plaque psoriasis. J. Am. Acad. Dermatol. . F. Gómez-García, Department of Dermatology, IMIBIC/Reina Sofía University Hospital, University of Cordoba, Córdoba, Spain; 2016;74:AB261.

28. Gossec L, Smolen JS, Ramiro S, De Wit M, Cutolo M, Dougados M, et al. European League Against Rheumatism (EULAR) recommendations for the management of psoriatic arthritis with pharmacological therapies: 2015 update. Ann. Rheum. Dis. . L. Gossec, Hôpital Pitié-Salpétrière, Service de Rhumatologie, Paris, France; 2016;75:499–510.

29. Gupta MA, Simpson FC, Gupta AK. Psoriasis and sleep disorders: A systematic review. Sleep Med. Rev. . M.A. Gupta, London, Canada; 2016;29:63–75.

30. Hanley T, Handford M, Lavery D, Yiu ZZN. Assessment and monitoring of biologic drug adverse events in patients with psoriasis. Psoriasis Targets Ther. . Z.Z.N. Yiu, Dermatology Centre, Salford Royal NHS Foundation Trust, Manchester, United Kingdom; 2016;6:41–54.

31. Haraoui B. Limitations in the full reporting of systematic literature review. J. Rheumatol. . B. Haraoui, Institut de Rhumatologie de Montréal, Division of Rheumatology, Montreal, Canada; 2016;43:994.

32. Harden JL, Lewis SM, Lish SR, Suárez-Fariñas M, Gareau D, Lentini T, et al. The tryptophan metabolism enzyme L-kynureninase is a novel inflammatory factor in psoriasis and other inflammatory diseases. J. Allergy Clin. Immunol. . J.L. Harden, Laboratory for Investigative Dermatology, Rockefeller University, New York, United States; 2016;137:1830–40.

33. Hinde S, Wade R, Palmer S, Woolacott N, Spackman E. Apremilast for the Treatment of Moderate to Severe Plaque Psoriasis: A Critique of the Evidence. Pharmacoeconomics . S. Hinde, Centre for Health Economics (CHE), University of York, York, United Kingdom; 2016;34:587–96.

34. Hoogedoorn L, Gerritsen MJP, Wolberink EAW, Peppelman M, van de Kerkhof PCM, van Erp PEJ. A four-phase strategy for the implementation of reflectance confocal microscopy in dermatology. J. Eur. Acad. Dermatology Venereol. . L. Hoogedoorn, Department of Dermatology Radboud University Medical Center Nijmegen The Netherlands; 2016;

35. Islam PS, Chang C, Selmi C, Generali E, Huntley A, Teuber SS, et al. Medical Complications of Tattoos: A Comprehensive Review. Clin. Rev. Allergy Immunol. . M.E. Gershwin, Division of Rheumatology, Allergy and Clinical Immunology, University of California at Davis School of Medicine, Davis, United States; 2016;1–14.

36. Izzo AA, Hoon-Kim S, Radhakrishnan R, Williamson EM. A Critical Approach to Evaluating Clinical Efficacy, Adverse Events and Drug Interactions of Herbal Remedies. Phyther. Res. . A.A. Izzo, Department of Pharmacy, University of Naples Federico II, Naples, Italy; 2016;30:691–700.

37. Jackson C, Maibach H. Ethnic and socioeconomic disparities in dermatology. J. Dermatolog. Treat. . C. Jackson, Department of Dermatology, University of California, San Francisco Medical Center, San Francisco, United States; 2016;27:290–1.

38. Kalra S, Gupta Y. Endocrine and metabolic effects of glucagon like peptide 1 receptor agonists (GLP1RA). J. Pak. Med. Assoc. . S. Kalra, Department of Endocrinology, Bharti Hospital, Karnal, Pakistan; 2016;66:357–9.

39. Karczewski J, Dobrowolska A, Rychlewska-Hańczewska A, Adamski Z. New insights into the role of T cells in pathogenesis of psoriasis and psoriatic arthritis. Autoimmunity . J. Karczewski, Department of Environmental Medicine; 2016;1–16.

40. Korman AM, Hill D, Alikhan A, Feldman SR. Impact and management of depression in psoriasis patients. Expert Opin. Pharmacother. . 2016;17:147–52.

41. Landriscina A, Friedman AJ. Integrating lifestyle-focused approaches into psoriasis care: Improving patient outcomes? Psoriasis Targets Ther. . A.J. Friedman, Department of Dermatology, George Washington School of Medicine and Health Sciences, Washington, United Kingdom; 2016;6:1–5.

42. Li X, Miao X, Wang H, Wang Y, Li F, Yang Q, et al. Association of serum uric acid levels in psoriasis: A systematic review and meta-analysis. Med. (United States) . B. Li, Department of Dermatology, Yueyang Hospital of Integrated Traditional Chinese and Western Medicine, Shanghai University of Traditional Chinese Medicine, Shanghai, China; 2016;95.

43. Li X, Xiao Q, Li F, Xu R, Fan B, Wu M, et al. Immune Signatures in Patients with Psoriasis Vulgaris of Blood-Heat Syndrome: A Systematic Review and Meta-Analysis. Evidence-based Complement. Altern. Med. . 2016;2016:1–11.

44. Ling TC, Clayton TH, Crawley J, Exton LS, Goulden V, Ibbotson S, et al. British Association of Dermatologists and British Photodermatology Group guidelines for the safe and effective use of psoralen-ultraviolet A therapy 2015. Br. J. Dermatol. . T.C. Ling, Dermatology Centre, Faculty of Medical and Human Sciences, Salford Royal NHS Foundation Trust, Salford, Manchester, United Kingdom; 2016;174:24–55.

45. Longden E, Read J. Assessing and reporting the adverse effects of antipsychotic medication: A systematic review of clinical studies, and prospective, retrospective, and cross-sectional research. Clin. Neuropharmacol. . E. Longden, Psychosis Research Unit, Greater Manchester West Mental Health NHS Foundation Trust, Harrop House, Prestwich, Manchester, United Kingdom; 2016;39:29–39.

46. Lv Z, Fan J, Zhang X, Huang Q, Han J, Wu F, et al. Integrative genomic analysis of interleukin-36RN and its prognostic value in cancer. Mol. Med. Rep. . Y. Jin, Department of Respiratory and Critical Care Medicine, Key Laboratory of Pulmonary Diseases of the Ministry of Health, Union Hospital, Tongji Medical College, Huazhong University of Science and Technology, Wuhan, Hubei, China; 2016;13:1404–12.

47. Ma K, Zhang H, Baloch Z. Pathogenetic and therapeutic applications of tumor necrosis factor-α (TNF-α) in major depressive disorder: A systematic review. Int. J. Mol. Sci. . K. Ma, Department of Physiology, Medical College of Qingdao University, Qingdao, China; 2016;17.

48. Mantovani A, Gisondi P, Lonardo A, Targher G. Relationship between non-alcoholic fatty liver disease and psoriasis: A novel Hepato-Dermal axis? Int. J. Mol. Sci. . G. Targher, Section of Endocrinology, Diabetes and Metabolism, Department of Medicine, University and Azienda Ospedaliera, Universitaria Integrata of Verona, Verona, Italy; 2016;17.

49. Maranda EL, Nguyen AH, Lim VM, Hafeez F, Jimenez JJ. Laser and light therapies for the treatment of nail psoriasis. J. Eur. Acad. Dermatology Venereol. . E.L. Maranda, Department of Dermatology and Cutaneous Surgery University of Miami Miller School of Medicine Miami, FL USA; 2016;

50. Mughal F, Barker J, Cawston H, Damera V, Bewley A, Morris J, et al. Cost-effectiveness of apremilast in moderate to severe psoriasis in the UK. J. Am. Acad. Dermatol. . F. Mughal, Celgene Ltd, United Kingdom; 2016;74:AB243.

51. Mysore V, Shashikumar BM. Targeted phototherapy. Indian J. Dermatol. Venereol. Leprol. . V. Mysore, Department of Dermatology, Venkat Charmalaya - Centre for Advanced Dermatology, Bengaluru, Karnataka, India; 2016;82:1–6.

52. Nyssen OP, Taylor SJC, Wong G, Steed E, Bourke L, Lord J, et al. Does therapeutic writing help people with long-term conditions? Systematic review, realist synthesis and economic considerations. Health Technol. Assess. (Rockv). . C. Meads, RAND Europe, Cambridge, United Kingdom; 2016;20:1–367.

53. Olsen JR, Gallacher J, Finlay AY, Piguet V, Francis NA. Quality of life impact of childhood skin conditions measured using the Children’s Dermatology Life Quality Index (CDLQI): A meta-analysis. Br. J. Dermatol. . J.R. Olsen, Institute of Health and Wellbeing, College of Medical Veterinary and Life Sciences, University of Glasgow, Glasgow, United Kingdom; 2016;174:853–61.

54. Orbai A-M, Ogdie A. Patient-Reported Outcomes in Psoriatic Arthritis. Rheum. Dis. Clin. North Am. . A.-M. Orbai, Division of Rheumatology, Johns Hopkins University, Baltimore, United States; 2016;42:265–83.

55. Park R, Lee WJ, Ji JD. Association between the three functional miR-146a single-nucleotide polymorphisms, rs2910164, rs57095329, and rs2431697, and autoimmune disease susceptibility: A meta-analysis. Autoimmunity . J.D. Ji, Division of Rheumatology, College of Medicine, Korea University, Seoul, Korea; 2016;1–8.

56. Petto H, Wilhelm S, Mallbris L, Dutronc Y, Leonardi C, Warren R. A statistical tool to convert published PASI 75, PASI 90 and PASI 100 response rates into absolute PASI values. J. Am. Acad. Dermatol. . H. Petto, Eli Lilly and Co, Indianapolis, United States; 2016;74:AB234.

57. Pickett K, Frampton G, Loveman E. Education to improve quality of life of people with chronic inflammatory skin conditions: a systematic review of the evidence. Br. J. Dermatol. 2016;174:1228–41.

58. Puig L, López-Ferrer A, Vilarrasa E, García I, Fernández-Del Olmo R. Model for assessing the efficiency of biologic drugs in the treatment of moderate to severe psoriasis for one year in clinical practice in Spain. Actas Dermosifiliogr. . L. Puig, Servicio de Dermatología, Hospital de la Santa Creu i Sant Pau, Universitat Autònoma de Barcelona, Barcelona, Spain; 2016;107:34–43.

59. Radtke MA, Spehr C, Reich K, Rustenbach SJ, Feuerhahn J, Augustin M. Treatment Satisfaction in Psoriasis: Development and Use of the PsoSat Patient Questionnaire in a Cross-Sectional Study. Dermatology . M.A. Radtke, aInstitute for Health Services Research in Dermatology and Nursing (IVDP), University Medical Center Hamburg-Eppendorf (UKE), and bDermatologikum Hamburg dermatologic practice, Hamburg, Germany; 2016;

60. Ramiro S, Smolen JS, Landewé R, Van Der Heijde D, Dougados M, Emery P, et al. Pharmacological treatment of psoriatic arthritis: A systematic literature review for the 2015 update of the EULAR recommendations for the management of psoriatic arthritis. Ann. Rheum. Dis. . S. Ramiro, Department of Rheumatology, Leiden University Medical Center, Leiden, Netherlands; 2016;75:490–8.

61. Richer V, Roubille C, Fleming P, Starnino T, McCourt C, McFarlane A, et al. Psoriasis and smoking: A systematic literature review and meta-analysis with qualitative analysis of effect of smoking on psoriasis severity. J. Cutan. Med. Surg. . V. Richer, Department of Medicine, Dermatology Service, St.-Luc Hospital, Montreal, Canada; 2016;20:221–7.

62. Romero-Jimenez RM, Escudero-Vilaplana V, Baniandres Rodriguez O, Garcia-Gonzalez X, Sanjurjo Saez M. Efficiency of biological therapies in patients with moderate to severe psoriasis: Impact of a pharmacotherapeutic protocol. J. Dermatolog. Treat. . R.M. Romero-Jimenez, Department of Pharmacy, Hospital General Universitario Gregorio Marañón, Madrid, Spain; 2016;27:198–202.

63. Rosenblat JD, McIntyre RS. Bipolar Disorder and Inflammation. Psychiatr. Clin. North Am. . R.S. McIntyre, Mood Disorder Psychopharmacology Unit, University Health Network, University of Toronto, Toronto, Canada; 2016;39:125–37.

64. Rothstein B, Gottlieb A. Secukinumab for treating plaque psoriasis. Expert Opin. Biol. Ther. . A. Gottlieb, Department of Dermatology, Tufts Medical Center, Tufts University, Boston, United States; 2016;16:119–28.

65. Ryoo JY, Yang H-J, Ji E, Yoo BK. Meta-analysis of the Efficacy and Safety of Secukinumab for the Treatment of Plaque Psoriasis. Ann. Pharmacother. . B.K. Yoo, College of Pharmacy, Gachon University, Incheon, South Korea; 2016;50:341–51.

66. Salavastru CM. Approach to the Evaluation and Management of Drug Eruptions. Curr. Dermatol. Rep. . C.M. Salavastru, Pediatric Dermatology Department, Colentina Clinical Hospital, Carol Davila University of Medicine and Pharmacy, Bucharest, Romania; 2016;5:49–57.

67. Schlager JG, Rosumeck S, Werner RN, Jacobs A, Schmitt J, Schlager C, et al. Topical treatments for scalp psoriasis. Cochrane Database Syst. Rev. . A. Nast, Charité - Universitätsmedizin Berlin, Division of Evidence Based Medicine, Department of Dermatology, Venerology and Allergology, Berlin, Germany; 2016;2016.

68. Shelton E, Laharie D, Scott FI, Mamtani R, Lewis JD, Colombel JF, et al. Cancer Recurrence Following Immune-Suppressive Therapies in Patients With Immune-Mediated Diseases: A Systematic Review and Meta-analysis. Gastroenterology . Elsevier, Inc; 2016;151:97–109.e4.

69. Svendsen MT, Andersen F, Hansen J, Johannessen H, Andersen KE. Medical adherence to topical corticosteroid preparations prescribed for psoriasis: A systematic review. J. Dermatolog. Treat. . M.T. Svendsen, Department of Dermatology and Allergy Centre, Odense University Hospital, Odense C, Denmark; 2016;1–8.

70. Taylor WJ, St.clair EW. Editorial: Shifting the Goal Posts: Treatment Recommendations for Ankylosing Spondylitis and the Newly Defined Condition of Nonradiographic Axial Spondyloarthritis. Arthritis Rheumatol. . W.J. Taylor, Department of Medicine, University of Otago Wellington, Wellington, New Zealand; 2016;68:265–9.

71. Teixeira A, Teixeira M, Almeida V, Torres T, Sousa Lobo JM, Almeida IF. Methodologies for medication adherence evaluation: Focus on psoriasis topical treatment. J. Dermatol. Sci. . I.F. Almeida, Faculdade de Farmácia da Universidade do Porto, Porto, Portugal; 2016;82:63–8.

72. Tencer T, Clancy Z, Zhang F. Cost per responder of apremilast and etanercept in patients with moderate to severe plaque psoriasis using results from liberate. J. Am. Acad. Dermatol. . T. Tencer, Celgene Corporation, Summit, United States; 2016;74:AB242.

73. Totté JEE, van der Feltz WT, Bode LGM, van Belkum A, van Zuuren EJ, Pasmans SGMA. A systematic review and meta-analysis on Staphylococcus aureus carriage in psoriasis, acne and rosacea. Eur. J. Clin. Microbiol. Infect. Dis. . J.E.E. Totté, Department of Dermatology, Erasmus MC University Medical Center, Rotterdam, Netherlands; 2016;35:1069–77.

74. Ungprasert P, Srivali N, Kittanamongkolchai W. Risk of Parkinson’s disease among patients with psoriasis: A systematic review and meta-analysis. Indian J. Dermatol. . P. Ungprasert, Department of Internal Medicine, Division of Rheumatology, Mayo Clinic, Rochester, United States; 2016;61:152–6.

75. Vaughn AR, Branum A, Sivamani RK. Effects of Turmeric (Curcuma longa) on Skin Health: A Systematic Review of the Clinical Evidence. Phyther. Res. . R.K. Sivamani, Department of Dermatology University of California 3301 C Street Suite 1400 Sacramento CA 95816 USA; 2016;

76. Vsn M, Mothe RK, Hyderboini RK, Likhar N, Ganji K, Singuru S, et al. Systematic review and meta-analysis of briakinumab, a fully human interleukin 12/23 monoclonal antibody , for the treatment of moderate to severe chronic plaque psoriasis. Value Heal. . M. Vsn, MarksMan Healthcare Solutions LLP, Health Economics and Outcomes Research (HEOR) and RWE (Real World Evidence), Navi Mumbai, India; 2016;19:A123.

77. Walia HK, Mehra R. Overview of common sleep disorders and intersection with dermatologic conditions. Int. J. Mol. Sci. . H.K. Walia, Center for Sleep Disorders Cleveland Clinic 11203, Cleveland, United States; 2016;17.

78. Wang J, Zhan Q, Zhang L. A systematic review on the efficacy and safety of Infliximab in patients with psoriasis. Hum. Vaccines Immunother. . L. Zhang, Tianjin Academy of Traditional Chinese Medicine Affiliated Hospital, Tianjin, China; 2016;12:431–7.

79. Wang X, Guo Z, Zhu Z, Bao Y, Yang B. Epicardial fat tissue in patients with psoriasis:a systematic review and meta-analysis. Lipids Health Dis. . Z. Guo, Department of Dermatovenereology, West China Hospital, Sichuan University, Chengdu, China; 2016;15.

80. West J, Ogston S, Foerster J. Safety and efficacy of methotrexate in psoriasis: A meta-analysis of published trials. PLoS One . 2016;11.

81. Wiseman SJ, Ralston SH, Wardlaw JM. Cerebrovascular disease in rheumatic diseases a systematic review and meta-analysis. Stroke . J.M. Wardlaw, Centre for Clinical Brain Sciences, University of Edinburgh, Chancellor’s Building, Royal Infirmary of Edinburgh, Edinburgh, United Kingdom; 2016;47:943–50.

82. Wong ITY, Shojania K, Dutz J, Tsao NW. Clinical and economic review of secukinumab for moderate-to-severe plaque psoriasis. Expert Rev. Pharmacoeconomics Outcomes Res. . N.W. Tsao, Faculty of Pharmaceutical Sciences, University of British Columbia, 2405 Wesbrook Mall, Vancouver, Canada; 2016;16:153–66.

83. Wu D, Shi D, Yang L, Zhu X. Association between methylenetetrahydrofolate reductase C677T polymorphism and psoriasis: A meta-analysis. J. Dermatol. . X. Zhu, Department of Dermatology, Nanfang Hospital, Southern Medical University, Guangzhou, Guangdong, China; 2016;43:162–9.

84. Xiao J-P, Wang X-R, Zhang S, Wang H-Y, Ye L, Pan H-F, et al. Association between rs6887695 and 3′-untranslated region polymorphisms within the interleukin-12B gene and susceptibility to autoimmune diseases in Asian and European population: A meta-analysis. Autoimmunity . D.-G. Wang, Department of Nephrology, The Second Affiliated Hospital of Anhui Medical University, Hefei, Anhui, China; 2016;49:277–84.

85. Yamauchi PS, Bissonnette R, Teixeira HD, Valdecantos WC. Systematic review of efficacy of anti-tumor necrosis factor (TNF) therapy in patients with psoriasis previously treated with a different anti-TNF agent. J. Am. Acad. Dermatol. . P.S. Yamauchi, Dermatology Institute and Skin Care Center, Clinical Science Institute, 2001 Santa Monica Blvd, Suite 1160W, Santa Monica, CA 90404; 2016;

86. Yan R, Jiang S, Wu Y, Gao X-H, Chen H-D. Topical calcipotriol/betamethasone dipropionate for psoriasis vulgaris: A systematic review. Indian J. Dermatol. Venereol. Leprol. . Y. Wu, Department of Dermatology, No. 1 Hospital of China Medical University, Shenyang, China; 2016;82:135–44.

87. Yang Z-S, Lin N-N, Li L, Li Y. The Effect of TNF Inhibitors on Cardiovascular Events in Psoriasis and Psoriatic Arthritis: an Updated Meta-Analysis. Clin. Rev. Allergy Immunol. . Z.-S. Yang, Department of Dermatology, The First Hospital of Qinhuangdao, Qinhuangdao, China; 2016;1–8.

88. Zhang CS, May B, Yan Y, Yu JJ, Yao D, Chang S, et al. Terms referring to psoriasis vulgaris in the classical Chinese medicine literature: a systematic analysis. Complement. Ther. Med. . Elsevier Ltd; 2016;25:55–60.

89. Zhang CS, Yang L, Zhang AL, May BH, Yu JJ, Guo X, et al. Is Oral Chinese Herbal Medicine Beneficial for Psoriasis Vulgaris? A Meta-Analysis of Comparisons with Acitretin. J. Altern. Complement. Med. . 2016;22:174–88.

90. Zhao Z, Xie F, Zhang X, Yang J, Wang R, Yang R, et al. Update on the association between interleukin-12 p40 gene polymorphism and risk of psoriasis: A meta-analysis. Dermatologica Sin. . H. Li, Department of Dermatology, Chinese People’s Liberation Army General Hospital, Number 28, Fuxing Road, Beijing 100853, China; 2016;

91. Zhu TH, Nakamura M, Abrouk M, Farahnik B, Koo J, Bhutani T. Demyelinating disorders secondary to TNF-inhibitor therapy for the treatment of psoriasis: A review. J. Dermatolog. Treat. . T.H. Zhu, University of Southern California Keck School of Medicine, Los Angeles, CA, USA; 2016;1–8.

92. Letter from the Editor. Hum. Vaccines Immunother. . 2016;12:255.

**2015**

93. Abbott R, Whear R, Nikolaou V, Bethel A, Coon JT, Stein K, et al. Tumour necrosis factor-α inhibitor therapy in chronic physical illness: A systematic review and meta-analysis of the effect on depression and anxiety. J. Psychosom. Res. . R. Abbott, PenCLAHRC, University of Exeter Medical School, Exeter, United Kingdom; 2015;79:175–84.

94. Ahn CS, Dothard EH, Garner ML, Feldman SR, Huang WW. To test or not to test? An updated evidence-based assessment of the value of screening and monitoring tests when using systemic biologic agents to treat psoriasis and psoriatic arthritis Presented in poster form at the 73rd Annual Meeting of the American Academy of Dermatology, San Francisco, CA, March 20-24, 2015. J. Am. Acad. Dermatol. . W.W. Huang, Department of Dermatology, Center for Dermatology Research, Wake Forest School of Medicine, Winston-Salem, United States; 2015;73:420–8.e1.

95. Alberdi T, Baldwin B. Development of cutaneous T-cell lymphoma during adalimumab monotherapy: A case report. J. Am. Acad. Dermatol. . T. Alberdi, University of South Florida Morsani, College of Medicine, Tampa, United States; 2015;72:AB157.

96. Ali FM, Cueva A, Vyas J, Piguet V, Salek S, Finlay AY. Quality of life measurement in therapeutic trials of psoriasis: A systematic review. J. Invest. Dermatol. . F.M. Ali, Cardiff University, Cardiff, United Kingdom; 2015;135:S37.

97. Almutawa F, Thalib L, Hekman D, Sun Q, Hamzavi I, Lim HW. Efficacy of localized phototherapy and photodynamic therapy for psoriasis: A systematic review and meta-analysis. Photodermatol. Photoimmunol. Photomed. . F. Almutawa, Department of Medicine, Faculty of Medicine, Kuwait University, Al-jabriya, Kuwait; 2015;31:5–14.

98. Alunno A, Carubbi F, Cafaro G, Pucci G, Battista F, Bartoloni E, et al. Targeting the IL-23/IL-17 axis for the treatment of psoriasis and psoriatic arthritis. Expert Opin. Biol. Ther. . G. Schillaci, Terni University Hospital, Unit of Internal Medicine, Terni, Italy; 2015;15:1727–37.

99. Anglada-Martinez H, Riu-Viladoms G, Martin-Conde M, Rovira-Illamola M, Sotoca-Momblona JM, Codina-Jane C. Does mHealth increase adherence to medication? Results of a systematic review. Int. J. Clin. Pract. . H. Anglada-Martinez, Pharmacy Service, Hospital Clinic Catalonia, Barcelona, Catalonia, Spain; 2015;69:9–32.

100. Armstrong AW, Florek AG. Response to: Is tonsillectomy a therapeutic option for plaque-type psoriasis? J. Am. Acad. Dermatol. . A.W. Armstrong, Department of Dermatology, University of Colorado Denver, Anschutz Medical Campus, Anschutz Medical Campus, Aurora, United States; 2015;73:e155.

101. Arora SK, Dewan P, Gupta P. Microbiome: Paediatricians’ perspective. Indian J. Med. Res. . S.K. Arora, Department of Pediatrics, Postgraduate Institute of Medical Education and Research and Dr Ram Manohar Lohia Hospital, New Delhi, India; 2015;142:515–24.

102. Atwan AA, Ingram JR, Abbott R, Kelson MJ, Pickles T, Bauer A, et al. Fumaric acid esters for psoriasis: Cochrane systematic review. Br. J. Dermatol. . A.A. Atwan, Cardiff University, Cardiff, United Kingdom; 2015;173:64.

103. Atzeni F, Masala IF, Salaffi F, Di Franco M, Casale R, Sarzi-Puttini P. Pain in systemic inflammatory rheumatic diseases. Best Pract. Res. Clin. Rheumatol. . F. Atzeni, Rheumatology Unit, L. Sacco University Hospital, Milan, Italy; 2015;29:42–52.

104. Azimi E, Lerner EA, Elmariah SB. Altered manifestations of skin disease at sites affected by neurological deficit. Br. J. Dermatol. . S.B. Elmariah, Department of Dermatology, Harvard Medical School, Massachusetts General Hospital, Boston, United States; 2015;172:988–93.

105. Balak DMW. Fumaric acid esters in the management of psoriasis. Psoriasis Targets Ther. . D.M.W. Balak, Department of Dermatology, Erasmus Medical Center, Rotterdam, Netherlands; 2015;5:9–23.

106. Bandino JP, Hang A, Norton SA. The Infectious and Noninfectious Dermatological Consequences of Flooding: A Field Manual for the Responding Provider. Am. J. Clin. Dermatol. . J.P. Bandino, Yorktown, United States; 2015;16:399–424.

107. Barbieri M, Capri S, Oskar B. Cost-utility analysis of apremilast for the treatment of moderate to severe plaque psoriasis in the Italian setting. Value Heal. . M. Barbieri, Centre for Health Economics, University of York, York, United Kingdom; 2015;18:A419–20.

108. Baurecht H, Hotze M, Brand S, Büning C, Cormican P, Corvin A, et al. Genome-wide comparative analysis of atopic dermatitis and psoriasis gives insight into opposing genetic mechanisms. Am. J. Hum. Genet. . S. Weidinger, Department of Dermatology, Allergology and Venereology, University Hospital Schleswig-Holstein, Campus Kiel, Kiel, Germany; 2015;96:104–20.

109. Blauvelt A, Armstrong AW, Krueger GG. Essential truths for the care and management of moderate-to-severe psoriasis. J. Drugs Dermatology . A. Blauvelt, Oregon Medical Research Center, Portland, United States; 2015;14:805–12.

110. Boehncke W-H, Anliker MD, Conrad C, Dudler J, Hasler F, Hasler P, et al. The dermatologists’ role in managing psoriatic arthritis: Results of a swiss delphi exercise intended to improve collaboration with rheumatologists. Dermatology . 2015;230:75–81.

111. Bowes J, Loehr S, Budu-Aggrey A, Uebe S, Bruce IN, Feletar M, et al. PTPN22 is associated with susceptibility to psoriatic arthritis but not psoriasis: Evidence for a further PsA-specific risk locus. Ann. Rheum. Dis. . A. Barton, Arthritis Research UK Centre for Genetics and Genomics, The University of Manchester, Manchester M13 9PT, UK; 2015;

112. Brezinski EA, Dhillon JS, Armstrong AW. Economic burden of psoriasis in the United States a systematic review. JAMA Dermatology . A.W. Armstrong, Department of Dermatology, University of Colorado Denver, Aurora, United States; 2015;151:651–8.

113. Broersen LHA, Pereira AM, Jørgensen JOL, Dekkers OM. Adrenal insufficiency in corticosteroids use: Systematic review and meta-analysis. J. Clin. Endocrinol. Metab. . O.M. Dekkers, Department of Clinical Epidemiology, Leiden University Medical Centre, Leiden, Netherlands; 2015;100:2171–80.

114. Burden-Teh E, Lam ML, Taibjee SM, Taylor A, Webster S, Dolman S, et al. How are we using systemic drugs to treat psoriasis in children? An insight into current clinical U.K. practice. Br. J. Dermatol. . 2015;173:614–8.

115. Candia R, Ruiz A, Torres-Robles R, Chávez-Tapia N, Méndez-Sánchez N, Arrese M. Risk of non-alcoholic fatty liver disease in patients with psoriasis: A systematic review and meta-analysis. J. Eur. Acad. Dermatology Venereol. 2015;29:656–62.

116. Capogrosso Sansone A, Mantarro S, Tuccori M, Ruggiero E, Montagnani S, Convertino I, et al. Safety Profile of Certolizumab Pegol in Patients with Immune-Mediated Inflammatory Diseases: A Systematic Review and Meta-Analysis. Drug Saf. . M. Tuccori, Tuscan Regional Centre for Pharmacovigilance, Unit of Adverse Drug Reaction Monitoring, University Hospital of Pisa, Pisa, Italy; 2015;38:869–88.

117. Capri S, Barbieri M, Oskar B. Cost-utility analysis of apremilast for the treatment of psoriatic arthritis in the Italian setting. Value Heal. . S. Capri, School of Economics and Management Cattaneo, LIUC University, Castellanza, Italy; 2015;18:A646.

118. Carrascosa JM, Belinchón I, De-La-Cueva P, Izu R, Luelmo J, Ruiz-Villaverde R. Expert recommendations on treating psoriasis in special circumstances. Actas Dermosifiliogr. . J.M. Carrascosa, Servei de Dermatologia, Hospital Universitari Germans Trias i Pujol, Badalona, Barcelona, Spain; 2015;106:292–309.

119. Carrascosa JM, Vanaclocha F, Caloto T, Echave M, Oyagüez I, Tencer T. Cost-utility analysis of apremilast for the treatment of moderate to severe psoriasis in Spain. Value Heal. . J.M. Carrascosa, Department of Dermatology, Germans Trias I Pujol University Hospital, Barcelona, Spain; 2015;18:A420.

120. Căruntu C, Negrei C, Ghiţă MA, Căruntu A, Bădărău AI, Buraga I, et al. Capsaicin, a hot topic in skin pharmacology and physiology. Farmacia . C. Negrei, Department of Toxicology, “Carol Davila” University of Medicine and Pharmacy, Bucharest, Romania; 2015;63:487–91.

121. Chandra A, Ray A, Senapati S, Chatterjee R. Genetic and epigenetic basis of psoriasis pathogenesis. Mol. Immunol. . R. Chatterjee, Human Genetics Unit, Indian Statistical Institute, Kolkata, India; 2015;64:313–23.

122. Chen H, Zhang T, Gong B, Cao X. Association between VEGF -634G/C polymorphism and susceptibility to autoimmune diseases: A meta-analysis. Gene . X. Cao, Department of Life Science, Beifang University of Nationalities, Yinchuan, China; 2015;558:181–6.

123. Chen W, Xu H, Wang X, Gu J, Xiong H, Shi Y. The tumor necrosis factor receptor superfamily member 1B polymorphisms predict response to anti-TNF therapy in patients with autoimmune disease: A meta-analysis. Int. Immunopharmacol. . Y. Shi, Department of Dermatology, Shanghai Tenth People’s Hospital, Tongji University School of Medicine, Shanghai, China; 2015;28:146–53.

124. Chen Y, Qian T, Zhang D, Yan H, Hao F. Clinical efficacy and safety of anti-IL-17 agents for the treatment of patients with psoriasis. Immunotherapy . F. Hao, Department of Dermatology, Southwest Hospital, Third Military Medical University, Chongqing, China; 2015;7:1023–37.

125. Choi YM, Adelzadeh L, Wu JJ. Photodynamic therapy for psoriasis. J. Dermatolog. Treat. . J.J. Wu, Department of Dermatology, Kaiser Permanente Medical Center, Los Angeles, United States; 2015;26:202–7.

126. Christensen R, Singh JA, Wells GA, Tugwell PS. Do “evidence-based recommendations” need to reveal the evidence? Minimal criteria supporting an “evidence claim.” J. Rheumatol. . R. Christensen, Copenhagen University Hospital at Frederiksberg, Musculoskeletal Statistics Unit, Parker Institute, Copenhagen F, Denmark; 2015;42:1737–9.

127. Conway R, Low C, Coughlan RJ, O’Donnell MJ, Carey JJ. Risk of liver injury among methotrexate users: A meta-analysis of randomised controlled trials. Semin. Arthritis Rheum. . R. Conway, Department of Rheumatology, Galway University Hospitals, Galway, Ireland; 2015;45:156–62.

128. Conway R, Low C, Coughlan RJ, O’Donnell MJ, Carey JJ. Methotrexate use and risk of lung disease in psoriasis, psoriatic arthritis, and inflammatory bowel disease: Systematic literature review and meta-analysis of randomised controlled trials. BMJ . R. Conway, Department of Rheumatology, Galway University Hospitals, Galway, Ireland; 2015;350.

129. Correia B, Torres T. Obesity: A key component of psoriasis. Acta Biomed. . T. Torres, Serviço de Dermatologia Centro Hospitalar do Porto, Porto, Portugal; 2015;86:121–9.

130. Coyle M, Deng J, Zhang AL, Yu J, Guo X, Xue CC, et al. Acupuncture therapies for psoriasis vulgaris: a systematic review of randomized controlled trials. Forsch. Komplementmed. . 2015;22:102–9.

131. Dannepond C, Maruani A, Machet L, Ternant D, Paintaud G, Samimi M. Serum infliximab concentrations in psoriatic patients treated with infliximab: A systematic review. Acta Derm. Venereol. . M. Samimi, Department of Dermatology, Laboratory of Molecular Virology and Immunology, ISP 1282, INRAUniversity François Rabelais, Hospital of Tours, Tours, France; 2015;95:401–6.

132. Dawwas MF, Aithal GP. The quest for an evidence-based approach to surveillance for methotrexate-related hepatotoxicity: Promise and perils. Br. J. Dermatol. . 2015;172:1684–5.

133. Depont F, Berenbaum F, Filippi J, Le Maitre M, Nataf H, Paul C, et al. Interventions to improve adherence in patients with immune-mediated inflammatory disorders: A systematic Review. PLoS One . 2015;10.

134. Duarte G V, Porto-Silva L, De Paim De Oliveira MF. Epidemiology and treatment of psoriasis: A Brazilian perspective. Psoriasis Targets Ther. . G.V. Duarte, Salvador, Brazil; 2015;5:55–64.

135. Dulai PS, Mosli M, Khanna R, Levesque BG, Sandborn WJ, Feagan BG. Vedolizumab for the treatment of moderately to severely active ulcerative colitis. Pharmacotherapy . B.G. Feagan, Robarts Clinical Trials, Robarts Research Institute, Western University, London, Canada; 2015;35:412–23.

136. Eder L, Gladman DD. Atherosclerosis in psoriatic disease: latest evidence and clinical implications. Ther. Adv. Musculoskelet. Dis. . D.D. Gladman, Centre for Prognosis Studies in The Rheumatic Diseases, Toronto Western Hospital, Toronto, Canada; 2015;7:187–95.

137. Edwards SK, Bates CM, Lewis F, Sethi G, Grover D. 2014 UK national guideline on the management of vulval conditions. Int. J. STD AIDS . S.K. Edwards, Cambridgeshire Community Services NHS Trust, West Suffolk Hospital, Bury St Edmunds, United Kingdom; 2015;26:611–24.

138. Fathi R, Armstrong AW. The Role of Biologic Therapies in Dermatology. Med. Clin. North Am. . R. Fathi, Department of Dermatology, University of Colorado Denver, Aurora, United States; 2015;99:1183–94.

139. Feldman SR, Tencer T, Clancy Z, Zhang F. Cost per responder of apremilast versus etanercept, adalimumab, and ustekinumab in patients with moderate to severe psoriasis. J. Am. Acad. Dermatol. . S.R. Feldman, Wake Forest School of Medicine, Winston-Salem, United States; 2015;72:AB229.

140. Feuerstein JD, Cullen G, Cheifetz AS. Immune-mediated reactions to anti-tumor necrosis factors in inflammatory bowel disease. Inflamm. Bowel Dis. . A.S. Cheifetz, Boston, United States; 2015;21:1176–86.

141. Fleming P, Kraft J, Gulliver WP, Lynde C. The relationship of obesity with the severity of psoriasis: A systematic review. J. Cutan. Med. Surg. . P. Fleming, Division of Dermatology, University of Toronto, Sunnybrook Health Sciences Centre (M1-722), Toronto, Canada; 2015;19:450–6.

142. Fleming P, Roubille C, Richer V, Starnino T, McCourt C, McFarlane A, et al. Effect of biologics on depressive symptoms in patients with psoriasis: A systematic review. J. Eur. Acad. Dermatology Venereol. . P. Fleming, Division of Dermatology, University of Toronto, Toronto, Canada; 2015;29:1063–70.

143. Florek A, Rachakonda T, Dhillon J, Armstrong A. A systematic review of tonsillectomy as a treatment for guttate or plaque psoriasis. J. Am. Acad. Dermatol. . A. Florek, University of Colorado Denver, Anschutz Medical Campus, Aurora, United States; 2015;72:AB223.

144. Foster S, Zhu B, Al Sawah S. Cost per additional responder associated with biologic use in psoriasis. Value Heal. . S. Foster, Eli Lilly and Company, Indianapolis, United States; 2015;18:A297.

145. Friedman B, English JC, Ferris LK. Indoor Tanning, Skin Cancer and the Young Female Patient: A Review of the Literature. J. Pediatr. Adolesc. Gynecol. . J.C. English, Department of Dermatology, University of Pittsburgh, Pittsburgh, United States; 2015;28:275–83.

146. Gieler U, Gieler T, Schut C, Niemeier V, Peters EM, Kupfer J. Quality of Life and Comorbidities in Urticaria: What Is Known? Curr. Dermatol. Rep. . U. Gieler, Department of Dermatology, University of Giessen, Giessen, Germany; 2015;4:77–82.

147. Gilbert KE, Manalo IF, Wu JJ. Efficacy and safety of etanercept and adalimumab with and without a loading dose for psoriasis: A systematic review. J. Am. Acad. Dermatol. . J.J. Wu, Department of Dermatology, Kaiser Permanente Los Angeles Medical Center, Los Angeles, United States; 2015;73:329–31.

148. Goetghebeur MM, Wagner M, Nikodem M, Zyla A, Micaleff A, Amzal B. Combining MCDA with advanced statistics to tackle challenges of data and judgment uncertainty: Case study of safety assessments. Value Heal. . M.M. Goetghebeur, LASER Analytica, Montreal, Canada; 2015;18:A12–3.

149. Goff K, Boyers LN, Karimkhani C, Lott JP, Dellavalle RP. Disease Burden Measures: a Review. Curr. Dermatol. Rep. . R.P. Dellavalle, Department of Veteran Affairs Medical Center, Denver, United States; 2015;4:30–5.

150. Gupta AK, Daigle D, Foley KA. The prevalence of culture-confirmed toenail onychomycosis in at-risk patient populations. J. Eur. Acad. Dermatology Venereol. . A.K. Gupta, University of Toronto, Toronto, Canada; 2015;29:1039–44.

151. Gupta M, Mahajan VK, Mehta KS, Chauhan PS, Rawat R. Peroxisome proliferator-activated receptors (PPARs) and PPAR agonists: the “future” in dermatology therapeutics? Arch. Dermatol. Res. . V.K. Mahajan, Department of Dermatology, Venereology and Leprosy, Dr. R. P. Govt. Medical College, Tanda, Himachal Pradesh, India; 2015;307:767–80.

152. Gupta P, Mandema JW, Ahadieh S, Wolk R, Valdez H, Mallbris L, et al. A model-based metaanalysis for dose-response comparison of psoriasis treatments. J. Am. Acad. Dermatol. . P. Gupta, Pfizer Inc, Groton, United States; 2015;72:AB221.

153. Haddad A, Johnson SR, Somaily M, Fazelzad R, Kron AT, Chau C, et al. Psoriatic arthritis mutilans: Clinical and radiographic criteria. A systematic review. J. Rheumatol. . V. Chandran, University of Toronto Psoriatic Arthritis Clinic, Toronto Western Hospital, Toronto, Canada; 2015;42:1432–8.

154. Hamilton MP, Ntais D, Griffiths CEM, Davies LM. Psoriasis treatment and management - A systematic review of full economic evaluations. Br. J. Dermatol. . M.P. Hamilton, Centre for Health Economics, University of Manchester, Jean McFarlane Building, Manchester, United Kingdom; 2015;172:574–83.

155. Henry AL, Kyle SD, Bhandari S, Chisholm A, Griffiths CE, Bundy C. Measurement, classification and evaluation of sleep disturbance in psoriasis: A systematic review. Sleep . A.L. Henry, Dermatology Centre, Manchester Academic Health Science Centre, University of Manchester, Manchester, United Kingdom; 2015;38:A310.

156. Higgins S, Wesley NO. Topical Retinoids and Cosmeceuticals: Where Is the Scientific Evidence to Recommend Products to Patients? Curr. Dermatol. Rep. . N.O. Wesley, Skin Care and Laser Physicians of Beverly Hills, Los Angeles, United States; 2015;4:56–62.

157. Hoffman MB, Farhangian M, Feldman SR. Psoriasis during pregnancy: Characteristics and important management recommendations. Expert Rev. Clin. Immunol. . M. Farhangian, Department of Dermatology, Center for Dermatology Research, Wake Forest School of Medicine, Winston-Salem, United States; 2015;11:709–20.

158. Honardoost MA, Naghavian R, Ahmadinejad F, Hosseini A, Ghaedi K. Integrative computational mRNA-miRNA interaction analyses of the autoimmune-deregulated miRNAs and well-known Th17 differentiation regulators: An attempt to discover new potential miRNAs involved in Th17 differentiation. Gene . K. Ghaedi, Division of Cellular and Molecular Biology, Department of Biology, Faculty of Sciences, University of Isfahan, Isfahan, Iran; 2015;572:153–62.

159. Hoogedoorn L, Peppelman M, Van De Kerkhof PCM, Van Erp PEJ, Gerritsen MJP. The value of in vivo reflectance confocal microscopy in the diagnosis and monitoring of inflammatory and infectious skin diseases: A systematic review. Br. J. Dermatol. . L. Hoogedoorn, Department of Dermatology, Radboud University Medical Center, HB Nijmegen, Netherlands; 2015;172:1222–48.

160. Huang H, Shen E, Tang S, Tan X, Guo X, Wang Q, et al. Increased serum resistin levels correlate with psoriasis: A meta-analysis. Lipids Health Dis. . H. Huang, Dermatological Department, First People’s Hospital of YueYang, YueYang, China; 2015;14.

161. Huang R-Y, Li L, Wang M-J, Chen X-M, Huang Q-C, Lu C-J. An Exploration of the Role of MicroRNAs in Psoriasis. Medicine (Baltimore). . 2015 [cited 2016 Nov 28];94:e2030.

162. Iskandarani G, Ehsan Khan E, Lau-Walker MO. Risk factors associated with atrial fibrillation in adults diagnosed under the age of 60-a systmatic review. Eur. Hear. J. Acute Cardiovasc. Care . G. Iskandarani, King’s College London, Florence Nightingale Faculty of Nursing and Midwifery, London, United Kingdom; 2015;4:78.

163. Jacobi A, Mayer A, Augustin M. Keratolytics and Emollients and Their Role in the Therapy of Psoriasis: a Systematic Review. Dermatol. Ther. (Heidelb). . A. Jacobi, Institute for Health Services Research in Dermatology and Nursing (IVDP), German Center for Dermatological Research (CeDeF), University Medical Center Hamburg-Eppendorf, Hamburg, Germany; 2015;5.

164. Jacobs A, Rosumeck S. Systematic review and meta-analysis of ustekinumab for moderate to severe psoriasis: Comment. Clin. Exp. Dermatol. . 2015;40:810–1.

165. Jacobs A, Rosumeck S, Nast A. Systematic review on the maintenance of response during systemic antipsoriatic therapy. Br. J. Dermatol. 2015;173:910–21.

166. Jadon DR, Nightingale AL, McHugh NJ, Lindsay MA, Korendowych E, Sengupta R. Serum soluble bone turnover biomarkers in psoriatic arthritis and psoriatic spondyloarthropathy. J. Rheumatol. . D.R. Jadon, Department of Rheumatology, Royal National Hospital for Rheumatic Diseases, Upper-Borough-Walls-Bath, United Kingdom; 2015;42:21–30.

167. Juncadella AC, Alame AM, Sands LR, Deshpande AR. Perianal crohn’s disease: A review. Postgrad. Med. . A.C. Juncadella, Department of Medicine, Massachusetts General Hospital, Boston, United States; 2015;127:266–72.

168. Kim J, Nadella P, Kim DJ, Brodmerkel C, Correa Da Rosa J, Krueger JG, et al. Histological stratification of thick and thin plaque psoriasis explores molecular phenotypes with clinical implications. PLoS One . 2015;10.

169. Kingsley GH, Scott DL. Assessing the effectiveness of synthetic and biologic disease-modifying antirheumatic drugs in psoriatic arthritis - A systematic review. Psoriasis Targets Ther. . G.H. Kingsley, Rheumatology Unit, Kings College London, Weston Education Centre, London, United Kingdom; 2015;5:71–81.

170. Kitchen H, Cordingley L, Young H, Griffiths CEM, Bundy C. Patient-reported outcome measures in psoriasis: The good, the bad and the missing! Br. J. Dermatol. . H. Kitchen, Dermatology Research Centre, University of Manchester, Manchester Academic Health Science Centre, Manchester, United Kingdom; 2015;172:1210–21.

171. Kölliker Frers RA, Bisoendial RJ, Montoya SF, Kerzkerg E, Castilla R, Tak PP, et al. Psoriasis and cardiovascular risk: Immune-mediated crosstalk between metabolic, vascular and autoimmune inflammation. IJC Metab. Endocr. . F. Capani, Rheumatology Department, J. M. Ramos Mejia Hospital, CABA, Buenos Aires, Argentina; 2015;6:43–54.

172. Kötter I. The challenging treatment of Behcets disease. Expert Opin. Orphan Drugs . I. Kötter, Asklepios Clinic Altona, The Department of Internal Medicine 4 (Rheumatology, Immunology, Nephrology), Hamburg, Germany; 2015;3:1101–10.

173. Krasuska M, Millings A, Lavda A, Thompson A. Psychosocial well-being and quality of life in rosacea: A systematic literature review. Br. J. Dermatol. . M. Krasuska, University of Sheffield, Sheffield, United Kingdom; 2015;173:206.

174. Kroon FP, van der Burg LR, Ramiro S, Landewé RB, Buchbinder R, Falzon L, et al. Non-steroidal anti-inflammatory drugs (NSAIDs) for axial spondyloarthritis (ankylosing spondylitis and non-radiographic axial spondyloarthritis). Cochrane database Syst. Rev. . 2015;7:CD010952.

175. Lee A, Gregory V, Gu Q, Becker DL, Barbeau M. Cost-effectiveness of secukinumab compared to current treatments for the treatment of moderate to severe plaque psoriasis in Canada. Value Heal. . A. Lee, Optum, Burlington, Canada; 2015;18:A182.

176. Lee HM, Liapakis A, Lim JK. Diagnosis, management, and prevention of hepatitis B reactivation. Curr. Hepat. Rep. . J.K. Lim, Yale Liver Center, Section of Digestive Diseases, Yale University School of Medicine, New Haven, United States; 2015;14:184–94.

177. Lee M-G, Bae S-C, Lee YH. Association between FOXP3 polymorphisms and susceptibility to autoimmune diseases: A meta-analysis. Autoimmunity . Y.H. Lee, Division of Rheumatology, Department of Internal Medicine, Korea University, College of Medicine, Korea University Anam Hospital, College of Medicine, Seongbuk-gu, Seoul, South Korea; 2015;48:445–52.

178. Lee RA, Eisen DB. Treatment of hidradenitis suppurativa with biologic medications. J. Am. Acad. Dermatol. . R.A. Lee, Dermatology Clinic, University of California San Diego, San Diego, United States; 2015;73:S82–8.

179. Lee YH, Song GG. Vascular endothelial growth factor gene polymorphisms and psoriasis susceptibility: A meta-analysis. Genet. Mol. Res. . Y.H. Lee, Division of Rheumatology, Department of Internal Medicine, Korea University College of Medicine, Seoul, South Korea; 2015;14:14396–405.

180. Li X, Kong L, Li F, Chen C, Xu R, Wang H, et al. Association between psoriasis and chronic obstructive pulmonary disease: A systematic review and meta-analysis. PLoS One . 2015;10.

181. Liang J, Zhang S, Luo Q, Li W, Tian X, Zhang F, et al. Lack of association between cytotoxic T-lymphocyte antigen-4+49A/G polymorphism and psoriasis and vitiligo: A meta-analysis of case-control studies. Gene . X. Zhang, Department of Dermatology, Guangzhou Institute of Dermatology, Guangzhou, China; 2015;568:196–202.

182. Lotti T, Hercogova J, Fabrizi G. Advances in the treatment options for vitiligo: Activated low-dose cytokines-based therapy. Expert Opin. Pharmacother. . T. Lotti, University of Rome G. Marconi, Rome, Italy; 2015;16:2485–96.

183. Mahajan BB, Kaur S. Interferons. Indian J. Dermatol. Venereol. Leprol. . S. Kaur, Department of Dermatology, Venereology and Leprology, Guru Gobind Singh Medical College and Hospital, Faridkot, Punjab, India; 2015;81:51–5.

184. Manalo IF, Gilbert KE, Wu JJ. Subcutaneous methotrexate for symptomatic control of severe recalcitrant psoriasis: Safety, efficacy, and patient acceptability. Psoriasis Targets Ther. . 2015;5:65–70.

185. Manriquez J, Andino-Navarrete R, Cataldo-Cerda K, Harz-Fresno I. Bibliometric characteristics of systematic reviews in dermatology: A cross-sectional study through Web of Science and Scopus. Dermatologica Sin. . J. Manriquez, Department of Dermatology, Pontificia Universidad Catolica de Chile, Santiago, Chile; 2015;33:154–6.

186. Marrie RA, Cohen J, Stuve O, Trojano M, Sørensen PS, Reingold S, et al. A systematic review of the incidence and prevalence of comorbidity in multiple sclerosis: Overview. Mult. Scler. J. . R.A. Marrie, Department of Internal Medicine, University of Manitoba, Health Sciences Center, Winnipeg, Canada; 2015;21:263–81.

187. Marrie RA, Reider N, Cohen J, Stuve O, Sorensen PS, Cutter G, et al. A systematic review of the incidence and prevalence of autoimmune disease in multiple sclerosis. Mult. Scler. J. . R.A. Marrie, Departments of Internal Medicine and Community Health Sciences, University of Manitoba, Health Sciences Center, Winnipeg, Canada; 2015;21:282–93.

188. May B, Deng S. Selecting herbs for psoriasis based on clinical, experimental and in-silico evidence. Integr. Med. Res. . B. May, RMIT University, Australia; 2015;4:129.

189. Meroni PL, Valentini G, Ayala F, Cattaneo A, Valesini G. New strategies to address the pharmacodynamics and pharmacokinetics of tumor necrosis factor (TNF) inhibitors: A systematic analysis. Autoimmun. Rev. . P.L. Meroni, Istituto G Pini, Piazza C. Ferrari, Milan, Italy; 2015;14:812–29.

190. Messori A, Trippoli S, Fadda V, Maratea D, Marinai C. Subcutaneous Biological Treatments for Moderate to Severe Psoriasis: Interpreting Safety Data by Network Meta-Analysis. Drugs - Real World Outcomes . A. Messori, HTA Unit, ESTAV Centro, Area Vasta Centro Toscana, Regional Health System, Prato, Italy; 2015;2:23–7.

191. Miroddi M, Navarra M, Calapai F, Mancari F, Giofrè S V, Gangemi S, et al. Review of Clinical Pharmacology of Aloe vera L. in the Treatment of Psoriasis. Phyther. Res. . G. Calapai, Department of Clinical and Experimental Medicine University of Messina Messina Italy; 2015;

192. Molina-Leyva A, Jiménez-Moleõn JJ, Naranjo-Sintes R, Ruiz-Carrascosa JC. Sexual dysfunction in psoriasis: A systematic review. J. Eur. Acad. Dermatology Venereol. 2015;29:649–55.

193. Mosca S, Gargiulo P, Balato N, Di Costanzo L, Parente A, Paolillo S, et al. Ischemic cardiovascular involvement in psoriasis: A systematic review. Int. J. Cardiol. . P. Perrone-Filardi, Department of Advanced Biomedical Sciences, Federico II University, Naples, Italy; 2015;178:191–9.

194. Mughal F, Cawston H, Cure S, Morris J, Tencer T, Zhang F. Cost-effectiveness of apremilast in psoriatic arthritis in Scotland. Value Heal. . F. Mughal, Celgene Ltd, Uxbridge, United Kingdom; 2015;18:A644.

195. Mughal F, Cawston H, Kinahan D, Morris J, Tencer T, Zhang F. Cost-effectiveness of apremilast in moderate to severe psoriasis in Scotland. Value Heal. . F. Mughal, Celgene Ltd, Uxbridge, United Kingdom; 2015;18:A420.

196. Murrell DF, Marinovic B, Caux F, Prost C, Ahmed R, Wozniak K, et al. Definitions and outcome measures for mucous membrane pemphigoid: Recommendations of an international panel of experts. J. Am. Acad. Dermatol. . D.F. Murrell, Department of Dermatology, St George Hospital, University of New South Wales, Sydney, Australia; 2015;72:168–74.

197. Murru A, Popovic D, Pacchiarotti I, Hidalgo D, León-Caballero J, Vieta E. Management of Adverse Effects of Mood Stabilizers. Curr. Psychiatry Rep. . E. Vieta, Bipolar Disorder Unit, Hospital Clínic, Barcelona, Spain; 2015;17.

198. Nair RP, Tsoi LC, Ghosh M, Stuart PE, Kabra M, Tejasvi T, et al. Genome wide association study of psoriasis in India. J. Invest. Dermatol. . R.P. Nair, Dermatology, University of Michigan, Ann Arbor, United States; 2015;135:S75.

199. Nast A, Jacobs A, Rosumeck S, Werner RN. Methods Report: European S3-Guidelines on the systemic treatment of psoriasis vulgaris - Update 2015 - EDF in cooperation with EADV and IPC. J. Eur. Acad. Dermatology Venereol. . A. Nast, Division of Evidence Based Medicine, Klinik für Dermatologie, Allergologie und Venerologie, Charité - Universitätsmedizin Berlin, Berlin, Germany; 2015;29:e1–22.

200. Nast A, Rosumeck S, Seidenschnur K. Biosimilars: A systematic review of published and ongoing clinical trials of antipsoriatics in chronic inflammatory diseases. JDDG - J. Ger. Soc. Dermatology . A. Nast, Division of Evidence Based Medicine (DEBM), Department of Dermatology, Charité - Universitätsmedizin Berlin, Berlin, Germany; 2015;13:294–301.

201. Nast A, Jacobs A, Rosumeck S, Werner RN. Efficacy and Safety of Systemic Long-Term Treatments for Moderate-to-Severe Psoriasis: A Systematic Review and Meta-Analysis. J. Invest. Dermatol. . Elsevier Masson SAS; 2015;135:2641–8.

202. Nguyen CM, Liao W. Genomic imprinting in psoriasis and atopic dermatitis: A review. J. Dermatol. Sci. . C.M. Nguyen, University of California, Irvine, School of Medicine, Irvine, United States; 2015;80:89–93.

203. Nititham J, Taylor KE, Gupta R, Ahn R, Lee KM, Chen H, et al. Meta-analysis of the TNIP1 region in psoriasis identifies two independent association signals. J. . Dermatol. . J. Nititham, UCSF, San Francisco, United States; 2015;135:S76.

204. Nititham J, Taylor KE, Gupta R, Chen H, Ahn R, Liu J, et al. Meta-analysis of the TNFAIP3 region in psoriasis reveals a risk haplotype that is distinct from other autoimmune diseases. Genes Immun. . W. Liao, Department of Dermatology, University of California San Francisco, San Francisco, United States; 2015;16:120–6.

205. Ogdie A, Weiss P. The Epidemiology of Psoriatic Arthritis. Rheum. Dis. Clin. North Am. . A. Ogdie, Division of Rheumatology, Center for Clinical Epidemiology and Biostatistics, Perelman School of Medicine, University of Pennsylvania, Philadelphia, United States; 2015;41:545–68.

206. Onuora S. Genetics: Meta-analysis reveals novel overlap in genetic aetiologies of paediatric autoimmune disorders. Nat. Rev. Rheumatol. . S. Onuora; 2015;11:561.

207. Palmer JB, Kanter S, Druyts E, Tsang Y, Herrera V. Clinical factors that modify treatment effects of biologics in psoriatic arthritis: A systematic review and meta-regression. Ann. Rheum. Dis. . J.B. Palmer, Novartis Pharmaceuticals, East Hanover, United States; 2015;74:1169–70.

208. Palmer JB, Kanter S, Druyts E, Tsang Y, Herrera V. Clinical factors that modify the effects of biologics on quality of life: A case study in psoriatic arthritis. Ann. Rheum. Dis. . J.B. Palmer, Novartis Pharmaceuticals, East Hanover, United States; 2015;74:1170.

209. Paternoster L, Standl M, Waage J, Baurecht H, Hotze M, Strachan DP, et al. Multi-ancestry genome-wide association study of 21,000 cases and 95,000 controls identifies new risk loci for atopic dermatitis. Nat. Genet. . L. Paternoster, Medical Research Council (MRC), Integrative Epidemiology Unit, University of Bristol, Bristol, United Kingdom; 2015;47:1449–56.

210. Pereira R, Lago P, Faria R, Torres T. Safety of Anti-TNF Therapies in Immune-Mediated Inflammatory Diseases: Focus on Infections and Malignancy. Drug Dev. Res. . T. Torres, Serviço de Dermatologia, Centro Hospitalar do Porto, Edifício das Consultas Externas, Ex-CICAP, Porto, Portugal; 2015;76:419–27.

211. Pickett K, Loveman E, Kalita N, Frampton GK, Jones J. Educational interventions to improve quality of life in people with chronic inflammatory skin diseases: Systematic reviews of clinical effectiveness and cost-effectiveness. Health Technol. Assess. (Rockv). . 2015;19:1–95.

212. Poddubnyy D, Van Tubergen A, Landewé R, Sieper J, Van Der Heijde D. Development of an ASAS-endorsed recommendation for the early referral of patients with a suspicion of axial spondyloarthritis. Ann. Rheum. Dis. . D. Poddubnyy, Rheumatology, Med. Department I, Charité Universitätsmedizin Berlin, Berlin, Germany; 2015;74:1483–7.

213. Prieto-Pérez R, Solano-Lõpez G, Cabaleiro T, Román M, Ochoa D, Talegõn M, et al. New immune system genetic polymorphisms associated with moderate-to-severe plaque psoriasis: A case-control study. Br. J. Dermatol. . F. Abad-Santos, Clinical Pharmacology Service, Hospital Universitario de la Princesa, Instituto de Investigaciõn Sanitaria Princesa (IP), Madrid, Spain; 2015;172:1432–5.

214. Puig L. On clinical thresholds, clinical equivalents and indirect comparisons of biological treatments for moderate-to-severe psoriasis. J. Clin. Pharm. Ther. . L. Puig, Department of Dermatology, Hospital de la Sant Antoni, Barcelona, Spain; 2015;40:131–4.

215. Puig L. PASI90 response: The new standard in therapeutic efficacy for psoriasis. J. Eur. Acad. Dermatology Venereol. . L. Puig, Department of Dermatology, Hospital de la Santa Creu i Sant Pau, Universitat Autònoma de Barcelona, Catalonia, Barcelona, Spain; 2015;29:645–8.

216. Qi JH, Qi JH, Shi N, Chen YJ, Nie G. Association between MTHFR 677C/T polymorphism and psoriasis risk: A meta-analysis. Genet. Mol. Res. . G. Nie, Department of Dermatology, Huangshi Central Hospital, Affiliated Hospital of Hubei Polytechnic University, Huangshi, China; 2015;14:3869–76.

217. Rachakonda TD, Dhillon JS, Florek AG, Armstrong AW. Effect of tonsillectomy on psoriasis: A systematic review. J. Am. Acad. Dermatol. . A.W. Armstrong, Department of Dermatology, University of Colorado at Denver, Anschutz Medical Campus, Aurora, United States; 2015;72:261–75.

218. Radack KP, Farhangian ME, Anderson KL, Feldman SR. A Review of the Use of Tanning Beds as a Dermatological Treatment. Dermatol. Ther. (Heidelb). . M.E. Farhangian, Department of Dermatology, Center for Dermatology Research, Wake Forest School of Medicine, Winston-Salem, United States; 2015;5:37–51.

219. Rogalski C. Calcipotriol/betamethasone for the treatment of psoriasis: Efficacy, safety, and patient acceptability. Psoriasis Targets Ther. . C. Rogalski, Medical Business Development, Business Management, edia.con gGmbH, Leipzig, Germany; 2015;5:97–107.

220. Roubille C, Richer V, Starnino T, McCourt C, McFarlane A, Fleming P, et al. The effects of tumour necrosis factor inhibitors, methotrexate, non-steroidal anti-inflammatory drugs and corticosteroids on cardiovascular events in rheumatoid arthritis, psoriasis and psoriatic arthritis: a systematic review and meta-analysis. Ann. Rheum. Dis. 2015;74:480–9.

221. Sandoval LF, Williams B, Feldman SR. Clinical potential of brodalumab in the management of psoriasis: The evidence to date. Psoriasis Targets Ther. . L.F. Sandoval, Department of Dermatology, Center for Dermatology Research, Wake Forest School of Medicine, Wilmington, United States; 2015;5:35–41.

222. Sawyer LM, Wonderling D, Jackson K, Murphy R, Samarasekera EJ, Smith CH. Biological Therapies for the Treatment of Severe Psoriasis in Patients with Previous Exposure to Biological Therapy: A Cost-Effectiveness Analysis. Pharmacoeconomics . L.M. Sawyer, Symmetron Limited, Hertfordshire, United Kingdom; 2015;33:163–77.

223. Shen C, Gao J, Yin X, Sheng Y, Sun L, Cui Y, et al. Association of the late cornified envelope-3 genes with psoriasis and psoriatic arthritis: A systematic review. J. Genet. Genomics . X. Zhang, Institute and Department of Dermatology, The First Affiliated Hospital, Anhui Medical University, Hefei, China; 2015;42:49–56.

224. Shenoy C, Shenoy MM, Rao GK. Dyslipidemia in dermatological disorders. N. Am. J. Med. Sci. . M.M. Shenoy, Departments of Dermatology, Yenepoya Medical College, Yenepoya University, Deralakatte, Mangalore, India; 2015;7:421–8.

225. Signorovitch JE, Betts KA, Yan YS, Lereun C, Sundaram M, Wu EQ, et al. Comparative efficacy of biological treatments for moderate-to-severe psoriasis: A network meta-analysis adjusting for cross-trial differences in reference arm response. Br. J. Dermatol. . J.E. Signorovitch, Analysis Group, Inc., Boston, United States; 2015;172:504–12.

226. Simpson RC. Cardiovascular disease risk factors in patients with hidradenitis suppurativa. Br. J. Dermatol. . R.C. Simpson, Centre of Evidence Based Dermatology, King’s Meadow Campus, University of Nottingham, Nottingham, United Kingdom; 2015;173:1118–9.

227. Siu S, Haraoui B, Bissonnette R, Bessette L, Roubille C, Richer V, et al. Meta-analysis of tumor necrosis factor inhibitors and glucocorticoids on bone density in rheumatoid arthritis and ankylosing spondylitis trials. Arthritis Care Res. . J.E. Pope, St. Joseph’s Hospital, London, Canada; 2015;67:754–64.

228. Smith MD. Review: Methotrexate does not increase risk for lung disease in psoriasis, psoriatic arthritis, or IBD. Ann. Intern. Med. . M.D. Smith, Repatriation General Hospital, Adelaide, Australia; 2015;163:JC7.

229. Sokolova A, Lee A, D Smith S. The Safety and Efficacy of Narrow Band Ultraviolet B Treatment in Dermatology: A Review. Am. J. Clin. Dermatol. . A. Lee, Department of Dermatology, Royal North Shore Hospital, St Leonards, Australia; 2015;16:501–31.

230. Song GG, Bae S-C, Kim J-H, Lee YH. The angiotensin-converting enzyme insertion/deletion polymorphism and susceptibility to rheumatoid arthritis, vitiligo and psoriasis: A meta-analysis. JRAAS - J. Renin-Angiotensin-Aldosterone Syst. . Y.H. Lee, Division of Rheumatology, Department of Internal Medicine, Korea University College of Medicine, Seongbuk-gu, Seoul, South Korea; 2015;16:195–202.

231. Song GG, Seo YH, Kim J-H, Choi SJ, Ji JD, Lee YH. Association between TNF-α (-308 A/G,-238 A/G,-857 C/T polymorphisms and responsiveness to TNF-α blockers in spondyloarthropathy, psoriasis and Crohn’s disease: A meta-analysis. Pharmacogenomics . Y.H. Lee, Division of Rheumatology, Korea University, College of Medicine, Seoul, South Korea; 2015;16:1427–37.

232. Sørensen JA, Clemmensen KK, Nixon RL, Diepgen TL, Agner T. Tobacco smoking and hand eczema - Is there an association? Contact Dermatitis . T. Agner, Department of Dermatology D40, Bispebjerg Hospital, Copenhagen NV, Denmark; 2015;73:326–35.

233. Sorenson E, Koo J. Evidence-based adverse effects of biologic agents in the treatment of moderate-to-severe psoriasis: Providing clarity to an opaque topic. J. Dermatolog. Treat. . E. Sorenson, San Francisco, United States; 2015;26:493–501.

234. Spinicci M, Mencarini J, Goletti D, Mantella A, Malva ND, Bartoloni A, et al. Discordance between the QuantiFERON-TB Gold In-Tube and Tuberculin Skin Test: Need for a further step? Eur. Respir. J. . F. Bartalesi, SOD Malattie Infettive e Tropicali, Azienda Ospedaliero-Universitaria Careggi, Florence, Italy; 2015;46:1506–9.

235. Sritheran D, Leung YY. Making the next steps in psoriatic arthritis management: current status and future directions. Ther. Adv. Musculoskelet. Dis. . Y.Y. Leung, Department of Rheumatology and Immunology, Singapore General Hospital, he Academia, , Singapore; 2015;7:173–86.

236. Steven S, Münzel T, Daiber A. Exploiting the pleiotropic antioxidant effects of established drugs in cardiovascular disease. Int. J. Mol. Sci. . A. Daiber, 2nd Medical Clinic, University Medical Center of the Johannes Gutenberg-University, Mainz, Germany; 2015;16:18185–223.

237. Stolwijk C, Van Tubergen A, Castillo-Ortiz JD, Boonen A. Prevalence of extra-articular manifestations in patients with ankylosing spondylitis: A systematic review and meta-analysis. Ann. Rheum. Dis. . C. Stolwijk, Department of Medicine, Division of Rheumatology, Maastricht University Medical Center, Maastricht, Netherlands; 2015;74:65–73.

238. Stuart PE, Nair RP, Tsoi LC, Tejasvi T, Das S, Kang HM, et al. Genome-wide Association Analysis of Psoriatic Arthritis and Cutaneous Psoriasis Reveals Differences in Their Genetic Architecture. Am. J. Hum. Genet. . J.T. Elder, Department of Dermatology, University of Michigan Medical School, Ann Arbor, United States; 2015;97:816–36.

239. Swindell WR, Sarkar MK, Stuart PE, Voorhees JJ, Elder JT, Johnston A, et al. Psoriasis drug development and GWAS interpretation through in silico analysis of transcription factor binding sites. Clin. Transl. Med. . W.R. Swindell, University of Michigan School of Medicine, Department of Dermatology, Ann Arbor, United States; 2015;4.

240. Tada Y. What is the risk of inadvertent exposure to topical retinoids during first trimester pregnancy? Br. J. Dermatol. . Y. Tada, Department of Dermatology, Teikyo University School of Medicine, Itabashi-Ku, Tokyo, Japan; 2015;173:1117–8.

241. Tajaddini MH, Keikha M, Razzazzadeh A, Kelishadi R. A systematic review on the association of serum selenium and metabolic syndrome. J. Res. Med. Sci. . M. Keikha, Department of Pediatrics, Child Growth and Development Research Center, Research Institute for Primordial Prevention of Non-communicable Disease, Isfahan University of Medical Sciences, Isfahan, Iran; 2015;20:782–9.

242. Tencer T, Clancy Z, Damera V, Zhang F, Cure S, Feldman S. Economic evaluation of apremilast in the treatment of moderate to severe psoriasis in the United States. J. Am. Acad. Dermatol. . T. Tencer, Celgene Corporation, Warren, United States; 2015;72:AB97.

243. Torales-Cardeña A, Martínez-Torres I, Rodríguez-Martínez S, Gómez-Chávez F, Cancino-Díaz JC, Vázquez-Sánchez EA, et al. Cross talk between proliferative, angiogenic, and cellular mechanisms orchestred by HIF-1 in Psoriasis. Mediators Inflamm. . M.E. Cancino-Díaz, Immunology Department, National School of Biological Sciences, National Polytechnic Institute, Mexico City, Mexico; 2015;2015.

244. Tsoi LC, Elder JT, Abecasis GR. Graphical algorithm for integration of genetic and biological data: proof of principle using psoriasis as a model. Bioinformatics . 2015;31:1243–9.

245. Tsoi LC, Spain SL, Ellinghaus E, Stuart PE, Capon F, Knight J, et al. Enhanced meta-analysis and replication studies identify five new psoriasis susceptibility loci. Nat. Commun. . G.R. Abecasis, Department of Biostatistics, Center for Statistical Genetics, University of Michigan, Ann Arbor, United States; 2015;6.

246. Tzanetakos C, Vassilopoulos D, Kourlaba G, Christou P, Maniadakis N. Cost-utility analysis of certolizumab pegol for the treatment of active psoriatic arthritis in Greece. Value Heal. . C. Tzanetakos, National School of Public Health, Athens, Greece; 2015;18:A646–7.

247. Ungprasert P, Cheungpasitporn W, Thongprayoon C, Sanguankeo A, Srivali N. Association between chronic obstructive pulmonary disease and psoriasis: A systematic review and meta-analysis. Am. J. Respir. Crit. Care Med. . P. Ungprasert, Mayo Clinic, Rochester, United States; 2015;191.

248. Ungprasert P, Srivali N, Thongprayoon C. Association between psoriasis and chronic obstructive pulmonary disease: A systematic review and meta-analysis. J. Dermatolog. Treat. . P. Ungprasert, Division of Rheumatology, Department of Internal Medicine, Mayo Clinic, Rochester, MN, USA; 2015;

249. Ungprasert P, Srivali N, Upala S, Sanguankeo A, Thongprayoon C, Cheungpasitporn W. Risk of venous thromboembolism in patients with ankylosing spondylitis: A systematic review and meta-analysis. Am. J. Respir. Crit. Care Med. . P. Ungprasert, Mayo Clinic, Rochester, United States; 2015;191.

250. Upala S, Sanguankeo A. Effect of lifestyle weight loss intervention on disease severity in patients with psoriasis: A systematic review and meta-analysis. Int. J. Obes. . A. Sanguankeo, Department of Internal Medicine, Bassett Medical Center, Columbia University, College of Physicians and Surgeons, Cooperstown, United States; 2015;39:1197–202.

251. Väätäinen S, Soini EJ, Valgardsson VS, Mälkönen T. Cost-effectiveness of ustekinumab in the treatment of psoriasis in Finland. Value Heal. . S. Väätäinen, ESIOR Oy, Kuopio, Finland; 2015;18:A670.

252. Van Bezooijen JS, Prens EP, Pradeepti MS, Atiqi R, Schreurs MWJ, Koch BCP, et al. Combining biologics with methotrexate in psoriasis: A systematic review. Br. J. Dermatol. . 2015;172:1676–80.

253. Van Geel MJ, Mul K, De Jager MEA, Van De Kerkhof PCM, De Jong EMGJ, Seyger MMB. Systemic treatments in paediatric psoriasis: A systematic evidence-based update. J. Eur. Acad. Dermatology Venereol. . M.J. Van Geel, Department of Dermatology, Radboud University Medical Center, Nijmegen, Netherlands; 2015;29:425–37.

254. Van Laarhoven AIM, Van Der Sman-Mauriks IM, Donders ART, Pronk MC, Van De Kerkhof PCM, Evers AWM. Placebo effects on itch: A meta-analysis of clinical trials of patients with dermatological conditions. J. Invest. Dermatol. . A.I.M. Van Laarhoven, Unit Health, Medical and Neuropsychology, Faculty of Social and Behavioural Sciences, Leiden University, Leiden, Netherlands; 2015;135:1234–43.

255. Vanderpuye-Orgle J, Zhao Y, Lu J, Shrestha A, Sexton A, Seabury S, et al. Evaluating the economic burden of psoriasis in the United States. J. Am. Acad. Dermatol. . M. Lebwohl, Mount Sinai Hospital, New York, United States; 2015;72:961–7.e5.

256. Vangeli E, Bakhshi S, Baker A, Fisher A, Bucknor D, Mrowietz U, et al. A Systematic Review of Factors Associated with Non-Adherence to Treatment for Immune-Mediated Inflammatory Diseases. Adv. Ther. . J. Weinman, Institute of Pharmaceutical Science, King’s College London, London, United Kingdom; 2015;32:983–1028.

257. Vena GA, Cassano N, Bellia G, Colombo D. Psoriasis in pregnancy: Challenges and solutions. Psoriasis Targets Ther. . G.A. Vena, Dermatology and Venereology Private Practice, Barletta, Italy; 2015;5:83–95.

258. Villani AP, Rouzaud M, Sevrain M, Barnetche T, Paul C, Richard M-A, et al. Prevalence of undiagnosed psoriatic arthritis among psoriasis patients: Systematic review and meta-analysis. J. Am. Acad. Dermatol. . A.P. Villani, Dermatology Department, Hôpital Edouard Herriot, Lyon, France; 2015;73:242–8.

259. Wang L, Yang H, Li N, Wang W, Bai Y. Acupuncture for psoriasis: Protocol for a systematic review. BMJ Open . Y. Bai, Department of Dermatology, China-Japan Friendship Hospital, Beijing, China; 2015;5.

260. Weisman MH. Psoriatic Arthritis. Rheum. Dis. Clin. North Am. . M.H. Weisman, Division of Rheumatology, Cedars-Sinai Medical Center, David Geffen School of Medicine at UCLA, Los Angeles, United States; 2015;41:xi.

261. Winthrop KL, Novosad SA, Baddley JW, Calabrese L, Chiller T, Polgreen P, et al. Opportunistic infections and biologic therapies in immune-mediated inflammatory diseases: Consensus recommendations for infection reporting during clinical trials and postmarketing surveillance. Ann. Rheum. Dis. . K.L. Winthrop, Division of Infectious Diseases, Oregon Health and Science University, Portland, United States; 2015;74:2107–16.

262. Wong D, Cook R, Lee K-A, Gladman D, Eder L, Chandran V. Reliability of radiographic assessment of psoriatic arthritis mutilans. J. Rheumatol. . D. Wong, University of Toronto, Toronto, Canada; 2015;42:1287.

263. Wong SH, Gao Q, Wu JC, Lee N, Chan FKL, Sung JJY, et al. Effect of immunosuppressive therapy on interferon gamma release assay (IGRA) in immune-mediated inflammatory diseases: A meta-analysis of 17 cohort studie. Gastroenterology . S.H. Wong; 2015;148:S433.

264. Wu D, Guo Y-Y, Xu N-N, Zhao S, Hou L-X, Jiao T, et al. Efficacy of anti-tumor necrosis factor therapy for extra-articular manifestations in patients with ankylosing spondylitis: A meta-analysis. BMC Musculoskelet. Disord. . N. Zhang, Second Departments of Rheumatology, Shengjing Hospital of China Medical University, Tiexi District, Shenyang Liaoning, China; 2015;16.

265. Wu JJ, Choi YM. Time to consider psoriasis an autoimmune disorder? J. Drugs Dermatology . J.J. Wu, Department of Dermatology, Kaiser Permanente Medical Center, Los Angeles, United States; 2015;14:112.

266. Wu S, Ding Y, Wu F, Li R, Xie G, Hou J, et al. Family history of autoimmune diseases is associated with an increased risk of autism in children: A systematic review and meta-analysis. Neurosci. Biobehav. Rev. . P. Mao, Research and Technology Service Center, Beijing, China; 2015;55:322–32.

267. Xiong H-Z, Gu J-Y, He Z-G, Chen W-J, Zhang X, Wang J-Y, et al. Efficacy and safety of secukinumab in the treatment of moderate to severe plaque psoriasis: A meta-analysis of randomized controlled trials. Int. J. Clin. Exp. Med. . Y. Shi, Department of Dermatology, Shanghai Tenth People’s Hospital, Tongji University School of Medicine, Shanghai, China; 2015;8:3156–72.

268. Yamamoto T. Optimal management of dactylitis in patients with psoriatic arthritis. Open Access Rheumatol. Res. Rev. . T. Yamamoto, Department of Dermatology, Fukushima Medical University, Fukushima, Japan; 2015;7:55–62.

269. Yamamoto T. Is tonsillectomy a therapeutic option for plaque-type psoriasis? J. Am. Acad. Dermatol. . T. Yamamoto, Department of Dermatology, Fukushima Medical University, Fukushima, Japan; 2015;73:e153.

270. Yang L, Zhang CS, May B, Yu J, Guo X, Zhang AL, et al. Efficacy of combining oral Chinese herbal medicine and NB-UVB in treating psoriasis vulgaris: A systematic review and meta-analysis. Chinese Med. (United Kingdom) . C.C. Xue, China-Australia International Research Centre for Chinese Medicine, School of Health Sciences, RMIT Health Innovations Research Institute, RMIT University, Bundoora, PO Box 71, Melbourne, VIC, 3083 Australia; 2015;

271. Yang Y, Brazier J, Longworth L. EQ-5D in skin conditions: an assessment of validity and responsiveness. Eur. J. Heal. Econ. . Y. Yang, Nuffield Department of Primary Care Health Sciences, University of Oxford, Oxford, United Kingdom; 2015;16:927–39.

272. Yao R, Du YY, Zhang YZ, Chen QH, Zhao LS, Li L. Association between G-217A polymorphism in the AGT gene and essential hypertension: A meta-analysis. Genet. Mol. Res. . Y.Z. Zhang, Department of Cardiology, The First Affiliated Hospital of Zhengzhou University, Zhengzhou, China; 2015;14:5527–34.

273. Yin X, Low HQ, Wang L, Li Y, Ellinghaus E, Han J, et al. Genome-wide meta-analysis identifies multiple novel associations and ethnic heterogeneity of psoriasis susceptibility. Nat. Commun. . A. Franke, Institute of Clinical Molecular Biology, Christian-Albrechts-University of Kiel, Kiel, Germany; 2015;6.

274. Yin X, Low H, Seielstad M, Liao W, Stahle M, Franke A, et al. Trans-ethnic genome-wide meta-analysis identifies multiple novel associations and reveals ethnic heterogeneity of psoriasis susceptibility. J. Invest. Dermatol. . X. Yin, Institute of Dermatology, Anhui Medical University, Hefei, China; 2015;135:S74.

275. Yiu ZZN, Warren RB, Mrowietz U, Griffiths CEM. Safety of conventional systemic therapies for psoriasis on reproductive potential and outcomes. J. Dermatolog. Treat. . Z.Z.N. Yiu, Dermatology Centre, Salford Royal NHS Foundation Trust, University of Manchester, Manchester Academic Health Science Centre, Manchester, United Kingdom; 2015;26:329–34.

276. Zhao CY, Murrell DF. Outcome measures for autoimmune blistering diseases. J. Dermatol. . D.F. Murrell, Department of Dermatology, St George Hospital, Sydney, Australia; 2015;42:31–6.

277. Drugs for psoriatic arthritis. Med. Lett. Drugs Ther. . 2015;57:88–92.

278. The international psoriasis council exome chip project: Exome arrays reveal known and novel coding variant associations. J. Invest. Dermatol. . 2015;135:S57.

**2014**

279. Ahlehoff O, Gislason G, Hansen PR. Cardiovascular aspects of psoriasis: An updated review. Int. J. Dermatol. . O. Ahlehoff, Department of Cardiology, Copenhagen University Hospital Roskilde, Gentofte, DK-2900, Denmark; 2014;53:e337–e337.

280. Albert U, De Cori D, Blengino G, Bogetto F, Maina G. Lithium treatment and potential long-term side effects: A systematic review of the literature. Riv. Psichiatr. . 2014;49:12–21.

281. Alexander W. American academy of dermatology and American college of cardiology. P T . W. Alexander, New York City, United States; 2014;39:370–4.

282. Almaghlouth I, Thava A, Haroon N, Inman RD. Differing patterns of axial spondyloarthritis in females and males. Arthritis Rheumatol. . I. Almaghlouth, Toronto Western Hospital, Toronto, Canada; 2014;66:S263–4.

283. Anić B, Padjen I, Mayer M, Bosnić D, Cerovec M. Clinical features of the SAPHO syndrome and their role in choosing the therapeutic approach: Report of four patients and review of the literature. Acta Dermatovenerologica Croat. . I. Padjen, Department of Internal Medicine, Division of Clinical Immunology and Rheumatology, University of Zagreb School of Medicine, University Hospital Centre Zagreb, Zagreb, Croatia; 2014;22:180–8.

284. Armstrong AW, Brezinski EA, Follansbee MR, Armstrong EJ. Effects of biologic agents and other disease-modifying antirheumatic drugs on cardiovascular outcomes in psoriasis and psoriatic arthritis: A Systematic review. Curr. Pharm. Des. . A. W. Armstrong, University of California Davis, Department of Dermatology, Sacramento, CA 95816, United States; 2014;20:500–12.

285. Armstrong AW, Harskamp CT, Dhillon JS, Armstrong EJ. Psoriasis and smoking: A systematic review and meta-analysis. Br. J. Dermatol. . A.W. Armstrong, Department of Dermatology, University of California, Davis, Sacramento, CA 95816, United States; 2014;170:304–14.

286. Armstrong AW, Tuong W, Love TJ, Carneiro S, Grynszpan R, Lee SS, et al. Treatments for nail psoriasis: A systematic review by the GRAPPA nail psoriasis work group. J. Rheumatol. . A.W. Armstrong, Psoriasis Program, Department of Dermatology, University of Colorado Denver, Aurora, United States; 2014;41:2306–14.

287. Armuzzi A, Lionetti P, Blandizzi C, Caporali R, Chimenti S, Cimino L, et al. Anti-TNF Agents as Therapeutic Choice in Immune-Mediated Inflammatory Diseases: Focus on Adalimumab. Int. J. Immunopathol. Pharmacol. . A. Armuzzi, IBD Unit, Complesso Integrato Columbus, Catholic University, Rome, Italy; 2014;27:11–32.

288. Augustin M, Mrowietz U, Bonnekoh B, Rosenbach T, Thaçi D, Reusch M, et al. Topical long-term therapy of psoriasis with vitamin D3 analogues, corticosteroids and their two compound formulations: Position paper on evidence and use in daily practice. JDDG - J. Ger. Soc. Dermatology . M. Augustin, CVderm - Centre of Excellence for Health Services Research in Dermatology, Institute for Health Services Research in Dermatology and Nursing, University Medical Center Hamburg-Eppendorf, 20246 Hamburg, Germany; 2014;12:667–82.

289. Azimi E, Lerner EA, Elmariah S. Altered manifestations of skin disease in patients with nerve damage. J. Invest. Dermatol. . E. Azimi, Dermatology, Massachusetts General Hospital, Boston, United States; 2014;134:S19.

290. Bang CN, Okin PM. Statin treatment, new-onset diabetes, and other adverse effects: A systematic review. Curr. Cardiol. Rep. . C.N. Bang, Department of Medicine, Division of Cardiology, Weill Cornell Medical College, New York, NY, United States; 2014;16.

291. Baurecht H, Hotze M, Elder JT, Weidinger S, Cordell HJ, Brown SJ. Genome-wide comparative analysis of atopic dermatitis and psoriasis gives insight into shared and opposing genetic risk mechanisms. J. Invest. Dermatol. . H. Baurecht, Dermatology, Allergology and Venerology, University Hospital Schleswig-Holstein, Kiel, Germany; 2014;134:S1.

292. Berki D, Burden D, Choon S-E, Allen M, Seyger M, Smith C, et al. The p.Asp176His CARD14 variant is associated with generalized pustular psoriasis presenting with concurrent psoriasis vulgaris. Br. J. Dermatol. . D. Berki, Division of Genetics and Molecular Medicine, King’s College London, London, United Kingdom; 2014;171:e147.

293. Beygi S, Lajevardi V, Abedini R. C-reactive protein in psoriasis: A review of the literature. J. Eur. Acad. Dermatology Venereol. . V. Lajevardi, Department of Dermatology, Razi Hospital, Tehran University of Medical Sciences, Tehran, Iran; 2014;28:700–11.

294. Bhatia BK, Millsop JW, Debbaneh M, Koo J, Linos E, Liao W. Diet and psoriasis, part II: Celiac disease and role of a gluten-free diet. J. Am. Acad. Dermatol. . W. Liao, Department of Dermatology, University of California, San Francisco, San Francisco, CA 94118, United States; 2014;71:350–8.

295. Bigby M. Understanding and evaluating systematic reviews and meta-analyses. Indian J. Dermatol. . M. Bigby, Department of Dermatology, Harvard Medical School, Beth Israel Deaconess Medical Center, Boston, MA 02215, United States; 2014;59:134–9.

296. Birnbaum J, Bingham CO. Non-length-dependent and length-dependent small-fiber neuropathies associated with tumor necrosis factor (TNF)-inhibitor therapy in patients with rheumatoid arthritis: Expanding the spectrum of neurological disease associated with TNF-inhibitors. Semin. Arthritis Rheum. . J. Birnbaum, Department of Neurology, Johns Hopkins University School of Medicine, Baltimore, MD 21224, United States; 2014;43:638–47.

297. Boehncke WH, Qureshi A, Merola JF, Thaçi D, Krueger GG, Walsh J, et al. Diagnosing and treating psoriatic arthritis: An update. Br. J. Dermatol. . A.B. Gottlieb, Tufts Medical Center, Boston MA 02111, United States; 2014;170:772–86.

298. Boehncke W-H, Alvarez Martinez D, Solomon J a, Gottlieb AB. Safety and efficacy of therapies for skin symptoms of psoriasis in patients with psoriatic arthritis: a systematic review. J. Rheumatol. . 2014;41:2301–5.

299. Bouwmeester W, Van Beurden-Tan C, Bennison C, Heeg B. The proportional odds model is more efficient than the multinomial logistic model for network meta-analyses of ordered outcomes. Value Heal. . W. Bouwmeester, Pharmerit International, Rotterdam, Netherlands; 2014;17:A566.

300. Brezinski EA, Follansbee MR, Armstrong EJ, Armstrong AW. Endothelial dysfunction and the effects of TNF inhibitors on the endothelium in psoriasis and psoriatic arthritis: A systematic review. Curr. Pharm. Des. . A. W. Armstrong, University of California Davis, Department of Dermatology, Sacramento, CA 95816, United States; 2014;20:513–28.

301. Bruner V, Atteno M, Spanò A, Scarpa R, Peluso R. Biological therapies for spondyloarthritis. Ther. Adv. Musculoskelet. Dis. . R. Peluso, Rheumatology Research Unit, Department of Clinical Medicine and Surgery, University Federico II, 80131 Naples, Italy; 2014;6:92–101.

302. Burudpakdee C, Khan ZM, Gala S, Nanavaty M, Kaura S. Impact of patient programs on adherence in inflammation and immunology: A global systematic review and meta-analysis of published evidence. Value Heal. . C. Burudpakdee, MKTXS, Raritan, United States; 2014;17:A534.

303. Busard C, Zweegers J, Limpens J, Langendam M, Spuls PI. Combined use of systemic agents for psoriasis: A systematic review. JAMA Dermatology . P.I. Spuls, Department of Dermatology, Academic Medical Center, University of Amsterdam, Amsterdam, Netherlands; 2014;150:1213–20.

304. Cantini F, Boccia S, Goletti D, Iannone F, Leoncini E, Panic N, et al. HBV reactivation in patients treated with antitumor necrosis factor-alpha (TNF- α) agents for rheumatic and dermatologic conditions: A systematic review and meta-analysis. Int. J. Rheumatol. . S. Boccia, Institute of Public Health, Department of Public Health, Università Cattolica Del Sacro Cuore, 00168 Rome, Italy; 2014;2014.

305. Capogrosso-Sansone A, Mantarro S, Blandizzi C, Montagnani S, Ruggiero E, Saporiti A, et al. Update of certolizumab pegol safety profile: A systematic review and meta-analysis. Drug Saf. . A. Capogrosso-Sansone, Department of Clinical and Experimental Medicine, University of Pisa, Pisa, Italy; 2014;37:844–5.

306. Capon F, Hussain S, Berki D, Choon S-E, Burden AD, Allen M, et al. IL36RN mutations define a severe, early-onset subtype of generalized pustular psoriasis. Br. J. Dermatol. . F. Capon, King’s College London, London, United Kingdom; 2014;171:e113–4.

307. Cardona-Arias JA, Franco-Aguirre JQ. Effect of the psoriasis on the health-related quality of life: Meta-analyses 2003-2013. Rev. Argentina Dermatologia . J.A. Cardona-Arias, Oficina 103, Medellín, Colombia; 2014;95.

308. Cawson MR, Mitchell SA, Knight C, Wildey H, Spurden D, Bird A, et al. Systematic review, network meta-analysis and economic evaluation of biological therapy for the management of active psoriatic arthritis. BMC Musculoskelet. Disord. . S.A. Mitchell, Abacus International, 6 Talisman Business Centre, Bicester OX26 6HR, United Kingdom; 2014;15.

309. Ceglowska U, Wlodarczyk A, Slomka M. Clinical effectiveness of fumaric acid esters (Fumaderm) in psoriasis: A systematic review of literature. Value Heal. . U. Ceglowska, Agency for Health Technology Assessment In Poland (AHTAPol), Warsaw, Poland; 2014;17:A605.

310. Chen M-L, Huang J, Xie Z-F. Comment on Song et al.: Associations between the major histocompatibility complex class i chain-related gene A transmembrane (MICA-TM) polymorphism and susceptibility to psoriasis and psoriatic arthritis: A meta-analysis. Rheumatol. Int. . Z.-F. Xie, Department of Geriatrics and Gerontology, First Affiliated Hospital, Guangxi Medical University, Nanning, China; 2014;34:297.

311. Chen Y, Lyga J. Brain-skin connection: Stress, inflammation and skin aging. Inflamm. Allergy - Drug Targets . Y. Chen, Global R&D, Avon Products, Suffern, United States; 2014;13:177–90.

312. Chen Y, Xin T, Cheng ASK. Evaluating the effectiveness of psychological and/or educational interventions in psoriasis: A narrative review. J. Dermatol. . Y. Chen, Guangdong Provincial Dermatology Hospital, Guangzhou, Guangdong Province, China; 2014;41:775–8.

313. Chi C-C, Wang S-H. Cost-efficacy of biologic therapies for moderate to severe psoriasis from the perspective of the Taiwanese healthcare system. Pharmacoepidemiol. Drug Saf. . C.-C. Chi, Department of Dermatology, Chang Gung Memorial Hospital, Chiayi, Taiwan; 2014;23:5.

314. Chi C-C, Wang S-H. Efficacy and cost-efficacy of biologic therapies for moderate to severe psoriasis: A meta-analysis and cost-efficacy analysis using the intention-to-treat principle. Biomed Res. Int. . S.-H. Wang, Department of Dermatology, Far Eastern Memorial Hospital, Banciao, New Taipei 22060, Taiwan; 2014;2014.

315. Churton S, Brown L, Shin TM, Korman NJ. Does treatment of psoriasis reduce the risk of cardiovascular disease? Drugs . S. Churton, Department of Dermatology, Murdough Family Center for Psoriasis, University Hospitals Case Medical Center, Cleveland, OH 44106, United States; 2014;74:169–82.

316. Cinotti E, Fouilloux B, Perrot JL, Labeille B, Douchet C, Cambazard F. Confocal microscopy for healthy and pathological nail. J. Eur. Acad. Dermatology Venereol. . E. Cinotti, Dermatology Department, University Hospital of Saint-Etienne, Saint-Etienne, France; 2014;28:853–8.

317. Coates LC, Kavanaugh A, Ritchlin CT. Systematic review of treatments for psoriatic arthritis: 2014 update for the GRAPPA. J. Rheumatol. . L.C. Coates, Leeds Institute of Rheumatic and Musculoskeletal Medicine, University of Leeds, Chapel Allerton Hospital, Leeds, United Kingdom; 2014;41:2273–6. Available from:http://www.embase.com/search/results?subaction=viewrecord&from=export&id=L602477388

318. Coates LC, Ritchlin CT, Kavanaugh AF. GRAPPA treatment recommendations: An update from the GRAPPA 2013 annual meeting. J. Rheumatol. . L.C. Coates, UK National Institute for Health Research Clinical Lecturer, Leeds Institute of Rheumatic and Musculoskeletal Medicine, University of Leeds, Leeds, United Kingdom; 2014;41:1237–9.

319. Conway R, Low C, Coughlan R, O’Donnell MJ, Carey JJ. Methotrexate and lung disease-a meta-analysis of randomized controlled trials. Ir. J. Med. Sci. . R. Conway, Department of Rheumatology, Galway Univeristy Hospitals, Galway National University of Ireland, Galway, Ireland; 2014;183:S516–7.

320. Cresce ND, Davis SA, Huang WW, Feldman SR. The quality of life impact of acne and rosacea compared to other major medical conditions. J. Drugs Dermatology . S.R. Feldman, Center for Dermatology Research, Department of Dermatology, Wake Forest School of Medicine, Winston-Salem, NC, United States; 2014;13:692–7.

321. De Castro Maymone MB, Gan SD, Bigby M. Evaluating the strength of clinical recommendations in the medical literature: GRADE, SORT, and AGREE. J. Invest. Dermatol. . M.B. De Castro Maymone, Department of Dermatology, Boston University School of Medicine, Boston, United States; 2014;134:e25.

322. de Eusebio E, Armario-Hita JC, de Miquel VA. Treatment of Psoriasis: Focus on Clinic-based Management with Infliximab. Am. J. Clin. Dermatol. . V.A. de Miquel, Servicio de Dermatología, Hospital General Universitario de Valencia, Valencia, Spain; 2014;15:5–16.

323. De Vecchis R, Palmisani L, Pucciarelli A, Ariano C, Giasi A, Cioppa C, et al. Protective effects of methotrexate against ischemic cardiovascular disorders in patients treated for rheumatoid arthritis or psoriasis: Novel therapeutic insights coming from a meta-analysis of the literature data. G. Ital. Cardiol. . R. De Vecchis, Cardiology Unit, Presidio Sanitario Intermedio Elena d’Aosta, Napoli, Italy; 2014;15:e54.

324. De Wit M, Campbell W, FitzGerald O, Gladman DD, Helliwell PS, James J, et al. Patient participation in psoriasis and psoriatic arthritis outcome research: A report from the GRAPPA 2013 annual meeting. J. Rheumatol. . M. De Wit, Department of Medical Humanities, VU Medical Centre, 1081 BT Amsterdam, Netherlands; 2014;41:1206–11.

325. Deng S, May BH, Zhang AL, Lu C, Xue CCL. Topical herbal formulae in the management of psoriasis: Systematic review with meta-analysis of clinical studies and investigation of the pharmacological actions of the main herbs. Phyther. Res. . C. Lu, Guangdong Provincial Academy of Chinese Medical Sciences, Guangdong Provincial Hospital of Chinese Medicine, Guangzhou, China; 2014;28:480–97.

326. Dogra S, Yadav S. Acitretin in psoriasis: An evolving scenario. Int. J. Dermatol. . S. Dogra, Department of Dermatology, Venereology and Leprology, Postgraduate Institute of Medical Education and Research, Chandigarh, 160012, India; 2014;53:525–38.

327. Dommasch ED, Troxel AB, Gelfand JM. Counterpoint: A tale of two meta-analyses revisited. J. Am. Acad. Dermatol. . E.D. Dommasch, Department of Dermatology, Boston University, Boston, MA 02118, United States; 2014;70:381–3.

328. Dowlatshahi EA, Wakkee M, Arends LR, Nijsten T. The prevalence and odds of depressive symptoms and clinical depression in psoriasis patients: a systematic review and meta-analysis. J. Invest. Dermatol. . Elsevier Masson SAS; 2014;134:1542–51.

329. Dregan A, Charlton J, Chowienczyk P, Gulliford MC. Chronic inflammatory disorders and risk of type 2 diabetes mellitus, coronary heart disease, and stroke : A population-based cohort study. Circulation . A. Dregan, Department of Primary Care and Public Health Sciences, King’s College London, London, SE1 3QD, United Kingdom; 2014;130:837–44.

330. Farrell E, Whistance R, Lloyd A, Jones A, Elwyn G, Durand M-A, et al. Shared decision-making in plaque psoriasis: Development of an Option Grid to assist in decisions to commence oral therapy. Br. J. Dermatol. . E. Farrell, Cardiff University, School of Medicine, Cardiff, United Kingdom; 2014;171:7.

331. Feldman SR, Burudpakdee C, Gala S, Nanavaty M, Mallya UG. The economic burden of psoriasis: A systematic literature review. Expert Rev. Pharmacoeconomics Outcomes Res. . C. Burudpakdee, MKTXS, Raritan, United States; 2014;14:685–705.

332. Felquer MLA, Coates LC, Soriano ER, Ranza R, Espinozaa LR, Helliwell PS, et al. Drug therapies for peripheral joint disease in psoriatic arthritis: A systematic review. J. Rheumatol. . E.R. Soriano, Sección Reumatologia, Servicio de Clinica Médica, Hospital Italiano de Buenos Aires, Argentina; 2014;41:2277–85.

333. Flatz L, Conrad C. Role of T-cell-mediated inflammation in psoriasis: Pathogenesis and targeted therapy. Psoriasis Targets Ther. . C. Conrad, Department of Dermatology, University Hospital of Lausanne (CHUV), 1011 Lausanne, Switzerland; 2014;4:1–10.

334. Franco-Aguirre JQ, Cardona-Arias JA. Characterization of health-related quality of life studies in people with psoriasis: Systematic review 2003-2013. Rev. Colomb. Reumatol. 2014;21:35–41.

335. Frendl DM, Ware JE. Patient-reported functional health and well-being outcomes with drug therapy: A systematic review of randomized trials using the SF-36 health survey. Med. Care . D.M. Frendl, Department of Quantitative Health Sciences, University of Massachusetts Medical School, Worcester, MA 01605, United States; 2014;52:439–45.

336. Frez MLF, Asawanonda P, Gunasekara C, Koh C, Loo S, Oon HH, et al. Recommendations for a patient-centered approach to the assessment and treatment of scalp psoriasis: A consensus statement from the Asia Scalp Psoriasis Study Group. J. Dermatolog. Treat. . M.L.F. Frez, Dermatology Clinic, Philippine General Hospital, Outpatient Building, Manila 1000, Philippines; 2014;25:38–45.

337. Gillespie T. Research evidence for reducing cardiovascular risk with biologic therapies in patients who have psoriasis. J. Dermatol. Nurses. Assoc. . T. Gillespie, University of Kansas Medical Center, Hays, KS 67601, United States; 2014;6:142–7.

338. Goel N, Charnce K. The biosimilar landscape: A systematic review of its current status. Arthritis Rheumatol. . N. Goel, Quintiles, Durham, United States; 2014;66:S662.

339. Gorouhi F, Maibach H. Evidence or experience: That is the question. Am. J. Clin. Dermatol. . F. Gorouhi, Department of Dermatology, University of California, Davis, Sacramento, CA 95816-3367, United States; 2014;15:147–8.

340. Gremese E, Tolusso B, Gigante MR, Ferraccioli G. Obesity as a risk and severity factor in rheumatic diseases (autoimmune chronic inflammatory diseases). Front. Immunol. . G. Ferraccioli, Division of Rheumatology, Institute of Rheumatology and Affine Sciences, Catholic University of the Sacred Heart, Rome, Italy; 2014;5.

341. Gulliver W, Gulliver S, Randell S. Serum ferritin levels as an indicator of anaemia in patients with moderate-to-severe psoriasis compared with the general public. Br. J. Dermatol. . W. Gulliver, Faculty of Medicine, Memorial University of Newfoundland, St. John’s, Canada; 2014;171:e161.

342. Gupta AK, Daigle D, Lyons DCA. Network meta-analysis of treatments for chronic plaque psoriasis in Canada. J. Cutan. Med. Surg. . A.K. Gupta, London, Canada; 2014;18:371–8.

343. Hamilton M, Ntais D, Griffiths C, Davies L. Value for money in treating psoriasis: A systematic review of full economic evaluations of psoriasis therapies. Br. J. Dermatol. . M. Hamilton, Centre for Health Economics, University of Manchester, Manchester, United Kingdom; 2014;171:e158.

344. Hashkes PJ, Becker ML, Cabral DA, Laxer RM, Paller AS, Rabinovich CE, et al. Methotrexate: New uses for an old drug. J. Pediatr. . P.J. Hashkes, Pediatric Rheumatology Unit, Shaare Zedek Medical Center, Jerusalem 9103102, Israel; 2014;164:231–6.

345. Hay RJ, Johns NE, Williams HC, Bolliger IW, Dellavalle RP, Margolis DJ, et al. The global burden of skin disease in 2010: An analysis of the prevalence and impact of skin conditions. J. Invest. Dermatol. . R.J. Hay, International Foundation for Dermatology, London, United Kingdom; 2014;134:1527–34.

346. Heyes C, Tait C, Toholka R, Gebauer K. Non-infectious skin disease in Indigenous Australians. Australas. J. Dermatol. . C. Heyes, Skin and Cancer Foundation, Carlton, VIC 3053, Australia; 2014;55:176–84.

347. Hotze M, Baurecht H, Rodriguez E, Elder JT, Franke A, Novak N, et al. Genome-wide comparative analysis of atopic eczema and psoriasis gives insight into disease mechanisms. Exp. Dermatol. . M. Hotze, Department of Dermatology, Allergology, and Venerology, University Hospital Schleswig-Holstein, Kiel, Germany; 2014;23:e21.

348. Hsieh J, Kadavath S, Efthimiou P. Can traumatic injury trigger psoriatic arthritis? A review of the literature. Clin. Rheumatol. . P. Efthimiou, Division of Rheumatology, Lincoln Medical and Mental Health Center, Bronx, NY 10451, United States; 2014;33:601–8.

349. Hsu L, Snodgrass BT, Armstrong AW. Antidrug antibodies in psoriasis: A systematic review. Br. J. Dermatol. . A.W. Armstrong, Department of Dermatology, University of California, Davis, Sacramento, CA 95816, United States; 2014;170:261–73.

350. Hu C, Wasfi Y, Zhuang Y, Zhou H. Information contributed by meta-analysis in exposure-response modeling: Application to phase 2 dose selection of guselkumab in patients with moderate-to-severe psoriasis. J. Pharmacokinet. Pharmacodyn. . C. Hu, Model Based Drug Development, Janssen Research and Development, LLC, Spring House, PA 19477, United States; 2014;41:239–50.

351. Hunn BHM, Martin WG, Simpson Jr. S, Mclean CA. Idiopathic granulomatous hypophysitis: A systematic review of 82 cases in the literature. Pituitary . B.H.M. Hunn, School of Medicine, University of Tasmania, Hobart, TAS 7000, Australia; 2014;17:357–65.

352. Iyer A, Elsone L, Appleton R, Jacob A. A review of the current literature and a guide to the early diagnosis of autoimmune disorders associated with neuromyelitis optica. Autoimmunity . A. Jacob, Walton Centre for Neurology and Neurosurgery, Liverpool L9 7LJ, United Kingdom; 2014;47:154–61.

353. Jadali Z, Eslami MB. T cell immune responses in psoriasis. Iran. J. Allergy, Asthma Immunol. . Z. Jadali, Department of Immunology, School of Public Health, Tehran University of Medical Sciences, Tehran, Iran; 2014;13:220–30.

354. Jani M, Massey J, Wedderburn L, Vencovský J, Danko K, Lundberg I, et al. Genetic risk factors in idiopathic inflammatory myopathies are shared with other autoimmune disorders in european populations. Ann. Rheum. Dis. . M. Jani, Arthritis Research UK Centre for Epidemiology, University of Manchester, Manchester, United Kingdom; 2014;73.

355. Jensen JD, Delcambre MR, Nguyen G, Sami N. Biologic Therapy with or Without Topical Treatment in Psoriasis: What Does the Current Evidence Say? Am. J. Clin. Dermatol. . N. Sami, Department of Dermatology, University of Alabama at Birmingham, South Birmingham, United States; 2014;15:379–85.

356. Karczewski J, Poniedziałek B, Rzymski P, Adamski Z. Factors affecting response to biologic treatment in psoriasis. Dermatol. Ther. . B. Poniedziałek, Poznan University of Medical Sciences, Poznan, Poland; 2014;27:323–30.

357. Karimkhani C, Boyers LN, Dunnick CA, Dellavalle R. Representation of the top three most disabling skin diseases in the cochrane database of systematic reviews. J. Invest. Dermatol. . C. Karimkhani, Columbia University, College of Physicians and Surgeons, New York, United States; 2014;134:S50.

358. Karimkhani C, Boyers LN, Prescott L, Welch V, Delamere FM, Nasser M, et al. Global burden of skin disease as reflected in Cochrane Database of Systematic Reviews. JAMA Dermatology . R.P. Dellavalle, Dermatology Service, Department of Veterans Affairs Medical Center, Denver, United States; 2014;150:945–51.

359. Katta R, Desai SP. Diet and dermatology: The role of dietary intervention in skin disease. J. Clin. Aesthet. Dermatol. . R. Katta, Baylor College of Medicine, Department of Dermatology, Houston, TX 77030, United States; 2014;7:46–51.

360. Kitchen H, Cordingley L, Young H, Griffiths C, Bundy C. Identifying patient-reported outcome measures for the clinical management of psoriasis. Br. J. Dermatol. . H. Kitchen, Centre for Dermatology Research, University of Manchester, Manchester Academic Health Science Centre, Manchester, United Kingdom; 2014;171:e129.

361. Klaassen KMG, Dulak MG, Van De Kerkhof PCM, Pasch MC. The prevalence of onychomycosis in psoriatic patients: A systematic review. J. Eur. Acad. Dermatology Venereol. . K.M.G. Klaassen, Department of Dermatology, Radboud University, Nijmegen Medical Centre, Nijmegen, Netherlands; 2014;28:533–41.

362. Kragballe K, Van De Kerkhof PC, Gordon KB. Unmet needs in the treatment of psoriasis. Eur. J. Dermatology . K. Kragballe, Dept of Dermatology, Århus University Hospital, Århus, Denmark; 2014;24:523–32.

363. Krishnareddy S, Swaminath A. When combination therapy isn’t working: Emerging therapies for the management of inflammatory bowel disease. World J. Gastroenterol. . A. Swaminath, Mount Sinai School of Medicine, Columbia University Medical Center, New York, NY 10029, United States; 2014;20:1139–46.

364. Lallas A, Giacomel J, Argenziano G, García-García B, González-Fernández D, Zalaudek I, et al. Dermoscopy in general dermatology: Practical tips for the clinician. Br. J. Dermatol. . A. Lallas, Skin Cancer Unit, Arcispedale Santa Maria Nuova IRCCS, Reggio Emilia, Italy; 2014;170:514–26.

365. Langley RG, Signorovitch J, Wang K, Betts KA, Sundaram M, Mulani P, et al. Number needed to treat and cost per responder for biologic therapies for the treatment of moderate to severe psoriasis. J. Am. Acad. Dermatol. . R.G. Langley, Dalhousie University, Halifax, Canada; 2014;70:AB2.

366. Lapadula G, Marchesoni A, Armuzzi A, Blandizzi C, Caporali R, Chimenti S, et al. Adalimumab in the Treatment of Immune-Mediated Diseases. Int. J. Immunopathol. Pharmacol. . A. Marchesoni, G. Pini Orthopedic Institute, Milano, Italy; 2014;27:33–48.

367. Larsen MH, Hagen KB, Krogstad A-L, Aas E, Wahl AK. Limited evidence of the effects of patient education and self-management interventions in psoriasis patients: A systematic review. Patient Educ. Couns. . M.H. Larsen, Department of Health Sciences, Institute of Health and Society, University of Oslo, Norway; 2014;94:158–69.

368. Lee J, Son C, Lee JA, Choi J, Lee MS. Do herbal medicines negatively affect liver tests? A systematic review of Korean clinical studies on safety. Eur. J. Integr. Med. . J.A. Lee, Medical Research Division, Korea Institute of Oriental Medicine, Daejeon, South Korea; 2014;6:441–50.

369. Liang Y, Pan H-F, Ye D-Q. Therapeutic potential of STAT4 in autoimmunity. Expert Opin. Ther. Targets . D.-Q. Ye, Anhui Medical University, School of Public Health, Department of Epidemiology and Biostatistics, Anhui, China; 2014;18:945–60.

370. Ling TC, Clayton TH, Crawley J, Exton LS, Goulden V, Ibbotson S, et al. British Association of Dermatologists and British Photodermatology Group guidelines for the safe and effective use of psoralen combined with ultraviolet A therapy 2014. Br. J. Dermatol. . T.C. Ling, Salford Royal NHS Foundation Trust, Salford, Manchester, United Kingdom; 2014;171:59.

371. Lis K, Kuzawińska O, Bałkowiec-Iskra E. Tumor necrosis factor inhibitors - State of knowledge. Arch. Med. Sci. . E. Bałkowiec-Iskra, Department of Experimental and Clinical Pharmacology, Medical University of Warsaw, Warsaw, Poland; 2014;10:1175–85.

372. Liu Y, Gong J-P, Li W-F. Therapeutic effect and safety of ustekinumab for plaque psoriasis: A meta-analysis. Chinese Med. Sci. J. . W.-F. Li, Department of Hepatibiliary Surgery, Second Affiliated Hospital, Chongqing Medical University, Chongqing, China; 2014;29:131–8.

373. Longworth L, Yang Y, Young T, Mulhern B, Hernández Alava M, Mukuria C, et al. Use of generic and condition-specific measures of health-related quality of life in NICE decision-making: A systematic review, statistical modelling and survey. Health Technol. Assess. (Rockv). . L. Longworth, Health Economics Research Group, Brunel University, Uxbridge, Middlesex, United Kingdom; 2014;18:1–224.

374. Lønnberg AS, Zachariae C, Skov L. Targeting of interleukin-17 in the treatment of psoriasis. Clin. Cosmet. Investig. Dermatol. . A.S. Lønnberg, Department of Dermato-Allergology, Gentofte Hospital, DK-2900 Hellerup, Denmark; 2014;7:251–9.

375. Lorenzetti R, Zullo A, Ridola L, Diamanti AP, Laganà B, Gatta L, et al. Higher risk of tuberculosis reactivation when anti-TNF is combined with immunosuppressive agents: A systematic review of randomized controlled trials. Ann. Med. . R. Lorenzetti, Ospedale Nuovo Regina Margherita, Roma, Italy; 2014;46:547–54.

376. Low C, Conway R, Coughlan RJ, O’Donnell MJ, Carey JJ. Methotrexate use is not associated with an increased risk of lung disease: A meta-analysis of randomised controlled trials. Ann. Rheum. Dis. . C. Low, Rheumatology, St. James Hospital, Dublin, Ireland; 2014;73.

377. Lubrano E, Spadaro A. Pharmacoeconomic burden in the treatment of psoriatic arthritis: From systematic reviews to real clinical practice studies. BMC Musculoskelet. Disord. . A. Spadaro, Dipartimento di Medicina Interna e Specialità Mediche - UOC di Reumatologia, Sapienza - Università di Roma, Azienda Policlinico Umberto i, 00161 Rome, Italy; 2014;15.

378. Lynch M, Kirby B, Warren RB. Treating moderate to severe psoriasis - Best use of biologics. Expert Rev. Clin. Immunol. . M. Lynch, Dermatology Department, St Vincent’s University Hospital, Dubline 4, Ireland; 2014;10:269–79.

379. Makara-Studzinśka M, Partyka I, Ziemecki P, Ziemecka A. The occurrence of emotional problems in somatic diseases based on psychodermatology. Arch. Psychiatry Psychother. . I. Partyka, Neuropsychiatric Hospital in Lublin, Poland; 2014;16:23–8.

380. Marí A, Morla A, Melero M, Schiavone R, Rodríguez J. Diffuse sclerosing osteomyelitis (DSO) of the mandible in SAPHO syndrome: A novel approach with anti-TNF therapy. Systematic review. J. Cranio-Maxillofacial Surg. . A. Marí, Department of Oral and Maxillofacial Surgery, University Hospital Bellvitge (HUB), Hospitalet de Llobregat, Barcelona, Spain; 2014;42:1990–6.

381. Marotte H, Cimaz R. Etanercept - TNF receptor and IgG1 Fc fusion protein: Is it different from other TNF blockers? Expert Opin. Biol. Ther. . H. Marotte, Hôpital Nord, CHU de Saint-Etienne, Department of Rheumatology, Saint-Etienne, France; 2014;14:569–72.

382. Mattei PL, Corey KC, Kimball AB. Psoriasis Area Severity Index (PASI) and the Dermatology Life Quality Index (DLQI): The correlation between disease severity and psychological burden in patients treated with biological therapies. J. Eur. Acad. Dermatology Venereol. . A.B. Kimball, Massachusetts General Hospital, Harvard Medical School, Boston, MA, United States; 2014;28:333–7.

383. Mauskopf J, Samuel M, McBride D, Mallya UG, Feldman SR. Treatment sequencing after failure of the first biologic in cost-effectiveness models of psoriasis: A systematic review of published models and clinical practice guidelines. Pharmacoeconomics . J. Mauskopf, RTI Health Solutions, Research Triangle Park, NC 27709, United States; 2014;32:395–409.

384. Maybury CM, Jabbar-Lopez ZK, Wong T, Dhillon AP, Barker JN, Smith CH. Methotrexate and liver fibrosis in people with psoriasis: A systematic review of observational studies. Br. J. Dermatol. 2014;171:17–29.

385. Maybury CM, Samarasekera E, Douiri A, Barker JN, Smith CH. Diagnostic accuracy of noninvasive markers of liver fibrosis in patients with psoriasis taking methotrexate: A systematic review and meta-analysis. Br. J. Dermatol. 2014;170:1237–47.

386. McFarlane A, Roubille C, Richer V, Starnino T, McCourt C, Fleming P, et al. Cardiovascular outcomes in patients with rheumatoid arthritis, psoriasis and psoriatic arthritis: A systematic review and meta-analyses. Ann. Rheum. Dis. . A. McFarlane, Division of Rheumatology, Department of Medicine, University of Alberta, Edmonton, Canada; 2014;73.

387. Meng Y, Dongmei L, Yanbin P, Jinju F, Meile T, Binzhu L, et al. Systematic review and meta-analysis of ustekinumab for moderate to severe psoriasis. Clin. Exp. Dermatol. 2014;39:696–707.

388. Messori A, Fadda V, Maratea D, Trippoli S, Gatto R, De Rosa M, et al. Biological drugs for the treatment of moderate-to-severe psoriasis by subcutaneous route: Determining statistical equivalence according to evidence-based methods. Clin. Drug Investig. . A. Messori, HTA Unit, Area Vasta Centro Toscana Regional Health System, 50100 Florence, Italy; 2014;34:593–8.

389. Mimoso C, Lee D-D, Zavadil J, Tomic-Canic M, Blumenberg M. Analysis and meta-analysis of transcriptional profiling in human epidermis . Methods Mol. Biol. 2014. p. 61–97.

390. Mounach A, El Maghraoui A. Efficacy and safety of adalimumab in ankylosing spondylitis. Open Access Rheumatol. Res. Rev. . A. El Maghraoui, Rheumatology Department, Military Hospital Mohammed V, Rabat, Morocco; 2014;6:83–90.

391. Murdaca G, Gulli R, Spanò F, Lantieri F, Burlando M, Parodi A, et al. TNF-α gene polymorphisms: Association with disease susceptibility and response to anti-TNF-α treatment in psoriatic arthritis. J. Invest. Dermatol. . G. Murdaca, Clinical Immunology Unit, Department of Internal Medicine, University of Genoa, Genoa, Italy; 2014;134:2503–9.

392. Nair R, Stuart P, Tsoi L, Ellinghaus E, Walsh J, Chandran V, et al. Genome-wide association analysis of psoriatic arthritis. Br. J. Dermatol. . R. Nair, University of Michigan, Ann Arbor, United States; 2014;171:e111.

393. Nanau RM, Cohen LB, Neuman MG. Risk of infections of biological therapies with accent on inflammatory bowel disease. J. Pharm. Pharm. Sci. . M.G. Neuman, Department of Pharmacology and Toxicology, In Vitro Drug Safety and Biotechnology, Banting Institute, Toronto, Canada; 2014;17:485–528.

394. Nanau RM, Neuman MG. Safety of anti-tumor necrosis factor therapies in arthritis patients. J. Pharm. Pharm. Sci. . M. G. Neuman, Department of Pharmacology and Toxicology, In VitroDrug Safety and Biotechnology, Banting Institute, Toronto, ON, M5G 1L7, Canada; 2014;17:324–61.

395. Nie G, Wan G-X, Qiu W, Xu Y-S. A meta-analysis on asociation of VEGF-460 T>C polymorphism with susceptibility to psoriasis. J. Clin. Dermatology . 2014;43:594–7.

396. Obradors M, Figueras M, Paz S, Comellas M, Lizán L. Factors conditioning health related quality of life in patients with psoriasis in europe: A systematic review of the literature. Value Heal. . M. Obradors, Novartis Farmaceutica S. A., Barcelona, Spain; 2014;17:A612.

397. Obradors M, Figueras M, Paz S, Comellas M, Lizán L. Costs of psoriasis in Europe. A systematic review of the literature. Value Heal. . M. Obradors, Novartis Farmaceutica S. A., Barcelona, Spain; 2014;17:A606.

398. Ogdie A, Schwartzman S, Eder L, Maharaj AB, Zisman D, Raychaudhuri SP, et al. Comprehensive treatment of psoriatic arthritis: Managing comorbidities and extraarticular manifestations. J. Rheumatol. . E. Husni, Cleveland Clinic, Rheumatologie and Immunologic Disease, Cleveland, United States; 2014;41:2315–22.

399. Orbai A-M, Weitz J, Siegel EL, Siebert S, Savage LJ, Aydin SZ, et al. Systematic review of treatment effectiveness and outcome measures for enthesitis in psoriatic arthritis. J. Rheumatol. . A.-M. Orbai, Johns Hopkins Arthritis Center, Baltimore, United States; 2014;41:2290–4.

400. Panchal S, Flint J, Van De Venne M, Piper M, Hurrell A, Cunningham J, et al. A systematic analysis of the safety of prescribing of anti-rheumatic, immunosuppressive and biologic drugs in men trying to conceive. Ann. Rheum. Dis. . S. Panchal, Leicester Hospitals, Leicester, United Kingdom; 2014;73.

401. Parkins G, Wylie G. Guideline vs. practice in procollagen-3-aminopeptide monitoring. Br. J. Dermatol. . G. Parkins, Alan Lyell Centre for Dermatology, Department of Dermatology, Southern General Hospital, Glasgow, United Kingdom; 2014;171:1599–600.

402. Posso-De Los Rios CJ, Pope E, Lara-Corrales I. A systematic review of systemic medications for pustular psoriasis in pediatrics. Pediatr. Dermatol. . I. Lara-Corrales, Hospital for Sick Children, Toronto, ON M5G 1X8, Canada; 2014;31:430–9.

403. Puig L. Biologic therapies for moderate to severe psoriasis are not interchangeable. Actas Dermosifiliogr. . L. Puig, Servicio de Dermatología, Hospital de la Santa Creu i Sant Pau, Universitat Autònoma de Barcelona, Barcelona, Spain; 2014;105:483–6.

404. Puig L, Lõpez A, Vilarrasa E, García I. Efficacy of biologics in the treatment of moderate-to-severe plaque psoriasis: A systematic review and meta-analysis of randomized controlled trials with different time points. J. Eur. Acad. Dermatology Venereol. . L. Puig, Department of Dermatology, Hospital de la Santa Creu i Sant Pau, Universitat Autònoma de Barcelona, Barcelona, Spain; 2014;28:1633–53.

405. Purohit VS, Lamba M, Gupta P. Pharmacometrics in dermatology . AAPS Adv. Pharm. Sci. Ser. V.S. Purohit, Department of Clinical Pharmacology, Global Innovative Pharma Business, Pfizer, Groton, United States; 2014. p. 499–516.

406. Qi M, Huang X, Zhou L, Zhang J. Four polymorphisms of VEGF (+405C>G,-460T>C,-2578C>A, and-1154G>A) in susceptibility to psoriasis: A meta-analysis. DNA Cell Biol. . J. Zhang, Department of Dermatology, Xiangya Hospital, Central South University, Changsha Hunan 410008, China; 2014;33:234–44.

407. Queiro R, Rodríguez S, Acasuso B, Morante I, Cabezas I, Coto P, et al. An onset of psoriasis after 40 years and a low education level may predict the development of diabetes mellitus in psoriatic arthritis. Ann. Rheum. Dis. . R. Queiro, Rheumatology, Oviedo, Spain; 2014;73.

408. Richard M-A, Barnetche T, Rouzaud M, Sevrain M, Villani AP, Aractingi S, et al. Evidence-based recommendations on the role of dermatologists in the diagnosis and management of psoriatic arthritis: Systematic review and expert opinion. J. Eur. Acad. Dermatology Venereol. . M.-A. Richard, Aix-Marseille University, Dermatology Department, Timone Hospital, Marseille, France; 2014;28:3–12.

409. Richard M-A, Paul C. Risk factors, diagnosis and management of psoriatic arthritis: Systematic literature reviews and expert opinion of a panel of dermatologists. J. Eur. Acad. Dermatology Venereol. . M.-A. Richard, Aix-Marseille University, Dermatology Department, Timone Hospital, Marseille, France; 2014;28:1–2.

410. Rodríguez-Cerdeira C, Molares-Vila A, Sánchez-Blanco E, Sánchez-Blanco B. Study on certain biomarkers of inflammation in psoriasis through “OMICS” platforms. Open Biochem. J. . C. Rodríguez-Cerdeira, Department of Dermatology, CHUVI, University of Vigo, Vigo, Spain; 2014;8:21–34.

411. Roper D, Al-nuaimi Y, Oza H, Goodfellow M, Spurgeon S. A systems biological model of chronic plaque psoriasis captures dynamics of known treatments. Br. J. Dermatol. . D. Roper, University of Exeter, Devon, United Kingdom; 2014;171:e146.

412. Roques CF. Balneotherapy actual medical benefit. Data of evidence for the last twenty years. Ann. Phys. Rehabil. Med. . C.F. Roques, AFRETH Scientific Committee President, Paris, France; 2014;57:e159.

413. Rose S, Toloza S, Bautista-Molano W, Helliwell PS. Comprehensive treatment of dactylitis in psoriatic arthritis. J. Rheumatol. . P.S. Helliwell, Institute of Molecular Medicine, Section of Musculoskeletal Disease, University of Leeds, Leeds, United Kingdom; 2014;41:2295–300.

414. Roubille C, Richer V, Starnino T, McCourt C, McFarlane A, Fleming P, et al. Evidence-based recommendations for the management of comorbidities in rheumatoid arthritis, psoriasis and psoriatic arthritis: A systematic literature search and expert opinion. Ann. Rheum. Dis. . C. Roubille, University of Montreal Hospital Research Center (CRCHUM), Notre Dame Hospital, Montreal, Canada; 2014;73.

415. Roubille C, Richer V, Starnino T, McCourt C, McFarlane A, Fleming P, et al. The effects of TNF inhibitors, methotrexate, NSAIDS and corticosteroids on cardiovascular events in rheumatoid arthritis, psoriasis and psoriatic arthritis: A systematic review and meta-analysis. Ann. Rheum. Dis. . C. Roubille, University of Montreal Hospital, Research Center (CRCHUM), Notre Dame Hospital, Montreal, Canada; 2014;73.

416. Rouzaud M, Sevrain M, Villani AP, Barnetche T, Paul C, Richard M-A, et al. Is there a psoriasis skin phenotype associated with psoriatic arthritis? Systematic literature review. J. Eur. Acad. Dermatology Venereol. . M. Beylot-Barry, Dermatology Department, CHU Bordeaux, University Bordeaux, Bordeaux, France; 2014;28:17–26.

417. Sandoval LF, Pierce A, Feldman SR. Systemic therapies for psoriasis: An evidence-based update. Am. J. Clin. Dermatol. . S.R. Feldman, Department of Dermatology, Center for Dermatology Research, Wake Forest School of Medicine, Winston-Salem, NC 27157-1071, United States; 2014;15:165–80.

418. Schmitt J, Rosumeck S, Thomaschewski G, Sporbeck B, Haufe E, Nast A. Efficacy and safety of systemic treatments for moderate-to-severe psoriasis: Meta-analysis of randomized controlled trials. Br. J. Dermatol. . J. Schmitt, Centre for Evidence-Based Healthcare, University Hospital Carl Gustav Carus, Technische Universität Dresden, D-01307 Dresden, Germany; 2014;170:274–303.

419. Selmi C. Unique topics and issues in rheumatology and clinical immunology. Clin. Rev. Allergy Immunol. . C. Selmi, Division of Rheumatology and Clinical Immunology, Humanitas Research Hospital, 20089 Rozzano, Milan, Italy; 2014;47:1–5.

420. Semble AL, Davis SA, Feldman SR. Safety and tolerability of tumor necrosis factor-α inhibitors in psoriasis: A narrative review. Am. J. Clin. Dermatol. . S.A. Davis, Center for Dermatology Research, Department of Dermatology, Wake Forest School of Medicine, Winston-Salem, NC 27157-1071, United States; 2014;15:37–43.

421. Sevrain M, Richard M-A, Barnetche T, Rouzaud M, Villani AP, Paul C, et al. Treatment for palmoplantar pustular psoriasis: Systematic literature review, evidence-based recommendations and expert opinion. J. Eur. Acad. Dermatology Venereol. . M. Sevrain, Dermatology Department, Morvan University Hospital, Brest, France; 2014;28:13–6.

422. Shaharyar S, Warraich H, McEvoy JW, Oni E, Ali SS, Karim A, et al. Subclinical cardiovascular disease in plaque psoriasis: Association or causal linkα. Atherosclerosis . K. Nasir, Center for Prevention and Wellness Research, Baptist Health Medical Group, Miami Beach, FL 33139, United States; 2014;232:72–8.

423. Simpson M. Identification of known and novel coding variant associations utilising exome arrays: The international psoriasis council exome chip project. Br. J. Dermatol. . M. Simpson, King’s College London, London, United Kingdom; 2014;171:e112–3.

424. Siu S, Haraoui B, Keeling S, Dutz J, Bessette L, Pope J. The effects of tumor necrosis factor inhibitors and corticosteroids on bone mineral density in patients with rheumatoid arthritis and ankylosing spondylitis: A meta-analysis of randomized controlled trials. J. Rheumatol. . S. Siu, Western University, London, Canada; 2014;41:1491.

425. Siu S, Haraoui B, Roubille C, Richer V, Starnino T, McCourt C, et al. Effect of disease modifying drugs on bone mineral density in patients with rheumatoid arthritis, psoriatic arthritis, psoriasis, and ankylosing spondylitis: A meta-analysis. Ann. Rheum. Dis. . S. Siu, Division of Rheumatology, Department of Medicine, University of Western Ontario, London, Canada; 2014;73. [L71551231](http://www.embase.com/search/results?subaction=viewrecord&from=export&id=L71551231)

426. Smiechowski B, Chen M, Vieira MC. Comparability of trial populations in network meta-analyses assessing biologic treatments in moderate to severe plaque psoriasis. Value Heal. . B. Smiechowski, Mapi, Boston, United States; 2014;17:A224.

427. Smiechowski B, Cope S. Assessing the relationship between treatment effect and baseline risk in network meta-analsysis of moderate to severe chronic plaque psoriasis trials. Value Heal. . B. Smiechowski, Mapi, Boston, United States; 2014;17:A544–5.

428. Song GG, Kim J-H, Lee YH. Associations between the major histocompatibility complex class i chain-related gene A transmembrane (MICA-TM) polymorphism and susceptibility to psoriasis and psoriatic arthritis: A meta-analysis. Rheumatol. Int. . Y.H. Lee, Department of Internal Medicine, Korea University Anam Hospital, Korea University College of Medicine, Seongbuk-gu, Seoul 136-705, South Korea; 2014;34:117–23.

429. Souto A, Maneiro JR, Salgado E, Carmona L, Gomez-Reino JJ. Risk of tuberculosis in patients with chronic immune-mediated inflammatory diseases treated with biologics and tofacitinib: a systematic review and meta-analysis of randomized controlled trials and long-term extension studies. Rheumatology (Oxford). . 2014;53:1872–85.

430. Sozzani S, Abbracchio MP, Annese V, Danese S, De Pità O, De Sarro G, et al. Chronic inflammatory diseases: Do immunological patterns drive the choice of biotechnology drugs? A critical review. Autoimmunity . S. Sozzani, Department of Molecular and Translational Medicine, University of Brescia, Brescia, Italy; 2014;47:287–306.

431. Strohal R, Kirby B, Puig L. Psoriasis beyond the skin: An expert group consensus on the management of psoriatic arthritis and common co-morbidities in patients with moderate-to-severe psoriasis. J. Eur. Acad. Dermatology Venereol. . R. Strohal, Department of Dermatology, Federal Academic Teaching Hospital, Feldkirch, Austria; 2014;28:1661–9.

432. Sun H, Xu B, Meng Q, Yang Q, Chang X. PSORS1C1/CDSN is associated with ankylosing spondylitis. Jt. Bone Spine . X. Chang, Medical Research Center of Shandong Provincial Qianfoshan Hospital, Shandong University, Jinan, Shandong, 250014, China; 2014;81:268–72.

433. Tangwongsiri D, Leartsakulpanitch J. Cost utility analysis of ustekinumab for the treatment of moderate to severe chronic plaque psoriasis in Thailand. Value Heal. . D. Tangwongsiri, Janssen-Cilag (Thailand) Limited, Bangkok, Thailand; 2014;17:A782–3.

434. Tausend W, Downing C, Tyring S. Systematic review of interleukin-12, interleukin-17, and interleukin-23 pathway inhibitors for the treatment of moderate-to-severe chronic plaque psoriasis: Ustekinumab, briakinumab, tildrakizumab, guselkumab, secukinumab, ixekizumab, and brodalumab. J. Cutan. Med. Surg. . C. Downing, Houston, TX 77004, United States; 2014;18:156–69.

435. Tencer T, Clancy Z, Cawston H, Cure S, Zhang F. Economic evaluation of sequencing strategies in the treatment of psoriatic arthritis in the united states. Arthritis Rheumatol. . T. Tencer, Celgene Corporation, Warren, United States; 2014;66:S705.

436. Terranova L, Mattozzi C, Richetta AG, Mantuano M, Cardosi L, Teruzzi C. Costs of therapy with biologics in the treatment of moderate to severe plaque psoriasis in the context of the Italian health-care system. G. Ital. di Dermatologia e Venereol. . C. Teruzzi, Temas a Quintiles Company Cassina Plaza, 20060 Cassina de’ Pecchi, Milan, Italy; 2014;149:131–43.

437. Tian G, Liang J-N, Wang Z-Y, Zhou D. Emerging role of leptin in rheumatoid arthritis. Clin. Exp. Immunol. . D. Zhou, Anhui Medical University, Hefei 230022, Anhui, China; 2014;177:557–70.

438. Tillett W, Adebajo A, Brooke M, Campbell W, Coates LC, FitzGerald O, et al. Patient involvement in outcome measures for Psoriatic arthritis. Curr. Rheumatol. Rep. . W. Tillett, Royal National Hospital for Rheumatic Diseases, Bath BA11RL, United Kingdom; 2014;16.

439. Toussirot É, Aubin F, Dumoulin G. Relationships between adipose tissue and psoriasis, with or without arthritis. Front. Immunol. . É. Toussirot, Clinical Investigation Center for Biotherapy INSERM CIC-1431, University Hospital of Besançon, Besançon 25000, France; 2014;5.

440. Tsoi L, Spain S, Ellinghaus E, Stuart P, Capon F, Tejasvi T, et al. Enhanced meta-analysis and replication studies identify five psoriasis susceptibility loci. Br. J. Dermatol. . L. Tsoi, University of Michigan, Ann Arbor, United States; 2014;171:e112.

441. Tzellos T, Kyrgidis A, Trigoni A, Zouboulis CC. Point: Major adverse cardiovascular events and anti-IL 12/23 agents. J. Am. Acad. Dermatol. . C.C. Zouboulis, Departments of Dermatology, Venereology Allergology and Immunology, Dessau Medical Center, 06847 Dessau, Germany; 2014;70:380–1.

442. Ungprasert P, Sanguankeo A, Upala S, Suksaranjit P. Psoriasis and risk of venous thromboembolism: a systematic review and meta-analysis. QJM . 2014;107:793–7.

443. Vaclavkova A, Chimenti S, Arenberger P, Holló P, Sator P-G, Burcklen M, et al. Oral ponesimod in patients with chronic plaque psoriasis: A randomised, double-blind, placebo-controlled phase 2 trial. Lancet . D. D’Ambrosio, Actelion Pharmaceuticals Ltd, Allschwil, Switzerland; 2014;384:2036–45.

444. Vary JC, O’Connor KM. Common dermatologic conditions. Med. Clin. North Am. . J.C. Vary, Division of Dermatology, Department of Internal Medicine, The University of Washington, Seattle, WA 98195, United States; 2014;98:445–85.

445. Vassallo C, Derlino F, Brazzelli V, D’Ospina RM, Borroni G. Acute generalized exanthematous pustulosis: Report of five cases and systematic review of clinical and histopathological findings. G. Ital. di Dermatologia e Venereol. . C. Vassallo, Department of Dermatology, University of Pavia, Policlinico San Matteo IRCCS Foundation, 27100 Pavia, Italy; 2014;149:281–90.

446. Villani AP, Rouzaud M, Sevrain M, Barnetche T, Paul C, Richard M-A, et al. Symptoms dermatologists should look for in daily practice to improve detection of psoriatic arthritis in psoriasis patients: An expert group consensus. J. Eur. Acad. Dermatology Venereol. . A.P. Villani, Dermatology Department, Lyon University, Edouard Herriot Hospital, Lyon, France; 2014;28:27–32.

447. Wang Y-D, Chen H, Liu H-Q, Hao M. Correlation between ovarian neoplasm and serum levels of osteopontin: a meta-analysis. Tumor Biol. . Y.-D. Wang, Department of Vascular Intervention, The Fourth Affiliated Hospital of Harbin Medical University, Harbin, China; 2014;35:11799–808.

448. Wat H, Dytoc M. Off-label uses of topical vitamin D in dermatology: A systematic review. J. Cutan. Med. Surg. . H. Wat, Faculty of Medicine and Dentistry, University of Alberta, 2J2.00 WC Mackenzie Health Sciences Centre, Edmonton, AB T6G 2R7, Canada; 2014;18:91–108.

449. Wootla B, Denic A, Rodriguez M. Polyclonal and monoclonal antibodies in clinic . Methods Mol. Biol. 2014. p. 79–110.

450. Xia J, Zhang W. A meta-Analysis revealed insights into the sources, conservation and impact of microRNA 5′-isoforms in four model species. Nucleic Acids Res. . W. Zhang, Institute for Systems Biology, Jianghan University, Wuhan, Hubei 430056, China; 2014;42:1427–41.

451. Xia T, Diao J, Huang H, Li J, Sun L, Li H, et al. Evaluation of the association between CD143 gene polymorphism and psoriasis. Cell Biochem. Biophys. . 2014;70:1617–23.

452. Yang Y, Zhang K, Zhou R. Meta-analysis of pre-miRNA polymorphisms association with susceptibility to autoimmune diseases. Immunol. Invest. . R. Zhou, Department of Obstetrics and Gynecology, West China Second University Hospital, Sichuan University, Chengdu 610041, China; 2014;43:13–27.

453. Zarur FP, d’Almeida LF V, Mafort MSP, de Gusmão PR, Avelleira JCR. Two cases of renal cell cancer during immunobiologic therapy for psoriasis. An. Bras. Dermatol. . F.P. Zarur, Rio de Janeiro, Brazil; 2014;89:1017–8.

454. Zhang CS, Yu JJ, Parker S, Zhang AL, May B, Lu C, et al. Oral Chinese herbal medicine combined with pharmacotherapy for psoriasis vulgaris: A systematic review. Int. J. Dermatol. . 2014;53:1305–18.

455. Zhang W, Islam N, Ma C, Anis AH. Systematic Review of Cost-Effectiveness Analyses of Treatments for Psoriasis. Pharmacoeconomics . A.H. Anis, Centre for Health Evaluation and Outcome Sciences, St. Paul’s Hospital, Vancouver, Canada; 2014;

456. Zhao YE, Hu L, Ma JX, Xiao SX, Zhao YL. Investigation of the association between psoriasis and human leucocyte antigens A by means of meta-analysis. J. Eur. Acad. Dermatology Venereol. . Y.E. Zhao, Department of Immunology and Pathogen Biology, Xi’An Jiaotong University College of Medicine, Shaanxi, Xi'an, China; 2014;28:355–69.

457. Zhou D, Chen W, Li X, Deng B, Xu W, Qu J, et al. Evidence-based practice guideline of Chinese herbal medicine for psoriasis vulgaris (Bai Bi). Eur. J. Integr. Med. . J. Liu, Centre for Evidence-Based Chinese Medicine, Beijing University of Chinese Medicine, Beijing, China; 2014;6:135–46.

458. Zschocke I, Mrowietz U, Lotzin A, Karakasili E, Reich K. Assessing adherence factors in patients under topical treatment: Development of the Topical Therapy Adherence Questionnaire (TTAQ). Arch. Dermatol. Res. . K. Reich, Dermatologikum Hamburg, 20354 Hamburg, Germany; 2014;306:287–97.

459. Australasian College of Dermatologists 47th Annual Scientific Meeting. Australas. J. Dermatol. . 2014;55.

**2013**

460. Ahn CS, Gustafson CJ, Sandoval LF, Davis SA, Feldman SR. Cost effectiveness of biologic therapies for plaque psoriasis. Am. J. Clin. Dermatol. . S.R. Feldman, Center for Dermatology Research, Department of Dermatology, Wake Forest University School of Medicine, Winston-Salem, NC 27157-1071, United States; 2013;14:315–26.

461. Ahn C, Gustafson C, Davis S, Feldman S. Cost-effectiveness of biologic therapies for plaque psoriasis: A systematic review. J. Am. Acad. Dermatol. . C. Ahn, Wake Forest School of Medicine, Winston-Salem, United States; 2013;68:AB2.

462. AlGhamdi K, Kumar A, Moussa N. The role of vitamin D in melanogenesis with an emphasis on vitiligo. Indian J. Dermatol. Venereol. Leprol. . K. AlGhamdi, Department of Dermatology, King Saud University, Riyadh 11322, Saudi Arabia; 2013;79:750–8.

463. Almutawa F, Alnomair N, Wang Y, Hamzavi I, Lim HW. Systematic review of UV-based therapy for psoriasis. Am. J. Clin. Dermatol. . H.W. Lim, Department of Dermatology, Henry Ford Medical Center-New Center One, Detroit MI 48202, United States; 2013;14:87–109.

464. Amerio P, Amoruso G, Bardazzi F, Campanati A, Cassano N, Conti A, et al. Detection and management of latent tuberculosis infections before biologic therapy for psoriasis. J. Dermatolog. Treat. . C. De Simone, Dept. Dermatology, Catholic University of the Sacred Heart, 00168 Rome, Italy; 2013;24:305–11.

465. Anjum N, Lee LH, Haworth A, Hughes B. Dermatology on-call service: Is it really necessary? Br. J. Dermatol. . N. Anjum, Portsmouth Hospitals NHS Trust, Portsmouth, United Kingdom; 2013;169:34–5.

466. Ariza JG, Nuñez C. A cost-effectiveness and budget impact analysis of different biologic treatments for psoriasis in Colombia. Value Heal. . J.G. Ariza, Janssen Cilag, Bogotá, Colombia; 2013;16:A696.

467. Armstrong AW, Harskamp CT, Armstrong EJ. The association between psoriasis and hypertension: A systematic review and meta-analysis of observational studies. J. Hypertens. . A.W. Armstrong, Department of Dermatology, University of California, Davis, Sacramento, CA 95816, United States; 2013;31:433–43.

468. Armstrong AW, Harskamp CT, Armstrong EJ. Psoriasis and the risk of diabetes mellitus: A systematic review and meta-analysis. JAMA Dermatology . A.W. Armstrong, Department of Dermatology, University of California, Davis, Sacramento, CA 95816, United States; 2013;149:84–91.

469. Armstrong AW, Harskamp CT, Armstrong EJ. Psoriasis and metabolic syndrome: A systematic review and meta-analysis of observational studies. J. Am. Acad. Dermatol. 2013. p. 654–62.

470. Armstrong EJ, Harskamp CT, Armstrong AW. Psoriasis and major adverse cardiovascular events: A systematic review and meta-analysis of observational studies. J. Am. Heart Assoc. . E.J. Armstrong, The Division of Cardiovascular Medicine, University of California, Davis, Minneapolis, MN 55454, United States; 2013;2.

471. Asseburg C, Valgardsson S, Soini EJ. Cost-effectiveness of sequences of biologic treatments for moderate-to-severe psoriasis in Finland. Value Heal. . C. Asseburg, ESiOR Oy, Kuopio, Finland; 2013;16:A507–8.

472. Asztalos ML, Heller MM, Lee ES, Koo J. The impact of emollients on phototherapy: A review. J. Am. Acad. Dermatol. . M.L. Asztalos, Philadelphia, PA 19103, United States; 2013;68:817–24.

473. Ayer J, Young HS. Pimecrolimus for psoriasis. Expert Opin. Pharmacother. . H.S. Young, University of Manchester, Salford Royal Hospital, Dermatology Research Centre, Manchester Academic Health Science Centre, Manchester, United Kingdom; 2013;14:767–74.

474. Bakewell CJ, Olivieri I, Aydin SZ, Dejaco C, Ikeda K, Gutierrez M, et al. Ultrasound and magnetic resonance imaging in the evaluation of psoriatic dactylitis: Status and perspectives. J. Rheumatol. . G.S. Kaeley, Division of Rheumatology, University of Florida, College of Medicine, Jacksonville, FL 32209-6561, United States; 2013;40:1951–7.

475. Barron AJ, Zaman N, Cole GD, Wensel R, Okonko DO, Francis DP. Systematic review of genuine versus spurious side-effects of beta-blockers in heart failure using placebo control: Recommendations for patient information. Int. J. Cardiol. . A.J. Barron, International Centre for Circulatory Health, Imperial College London, London W2 1LA, United Kingdom; 2013;168:3572–9.

476. Basharat P, Wilson A, Levstik M, Barra L. Transient elastography (fibroscan) for monitoring of liver fibrosis in methotrexate-treated patients with inflammatory disorders: A systematic review. J. Rheumatol. . P. Basharat, Western University, London, Canada; 2013;40:1009.

477. Bettoli V, Zauli S, Virgili A. Retinoids in the chemoprevention of non-melanoma skin cancers: Why, when and how. J. Dermatolog. Treat. . V. Bettoli, Department of Clinical and Experimental Medicine, Section of Dermatology, University of Ferrara, Arcispedale S. Anna, 44100 Ferrara, Italy; 2013;24:235–7.

478. Betts KA, Sundaram M, Mughal F, Yan SY, Signorovitch J, Wang K, et al. Cost-effectiveness of biologic therapies for the treatment of moderate to severe psoriasis in the United Kingdom. Value Heal. . K.A. Betts, Analysis Group, Inc., Boston, United States; 2013;16:A505–6.

479. Blok JL, Van Hattem S, Jonkman MF, Horváth B. Systemic therapy with immunosuppressive agents and retinoids in hidradenitis suppurativa: A systematic review. Br. J. Dermatol. . J.L. Blok, Department of Dermatology, University of Groningen, University Medical Center Groningen, 9700 RB Groningen, Netherlands; 2013;168:243–52.

480. Brazzelli V, Grasso V, Borroni G. Imatinib, dasatinib and nilotinib: A review of adverse cutaneous reactions with emphasis on our clinical experience. J. Eur. Acad. Dermatology Venereol. . V. Brazzelli, Department of Clinical-Surgical, Diagnostic and Paediatric Sciences, Institute of Dermatology, University of Pavia, Pavia, Italy; 2013;27:1471–80.

481. Brenaut E, Barnetche T, Misery L. Alcohol consumption: Is it really a risk factor for psoriasis? J. Dermatol. . L. Misery, Department of Dermatology, University Hospital of Brest, 29200 Brest, France; 2013;40:508.

482. Brenaut E, Horreau C, Pouplard C, Barnetche T, Paul C, Richard M-A, et al. Alcohol consumption and psoriasis: A systematic literature review. J. Eur. Acad. Dermatology Venereol. . E. Brenaut, Dermatology Department, Morvan University Hospital, Brest, France; 2013;27:30–5.

483. Brodszky V, Mo M, Gulacsi L, Baji P, Balogh O, Péntek M. Indirect comparison of the effect of biologics in patients with psoriasis; A meta-analysis of randomized, double blind clinical trials in bayesian framework. Value Heal. . V. Brodszky, Corvinus University of Budapest, Budapest, Hungary; 2013;16:A501–2.

484. Cárdenas-Roldán J, Rojas-Villarraga A, Anaya J-M. How do autoimmune diseases cluster in families? A systematic review and meta-analysis. BMC Med. . J.-M. Anaya, Center for Autoimmune Diseases Research (CREA), School of Medicine and Health Sciences, Universidad del Rosario, Bogota, Colombia; 2013;11.

485. Casanova Estruch B. Safety profile and practical considerations of monoclonal antibody treatment. Neurologia . B. Casanova Estruch, Unitat d’Esclerosi Múltiple, Hospital Universitari La Fe, Valencia, Spain; 2013;28:169–78.

486. Castiblanco J, Arcos-Burgos M, Anaya J-M. What is next after the genes for autoimmunity? BMC Med. . J.-M. Anaya, Center for Autoimmune Diseases Research (CREA), School of Medicine and Health Sciences, Universidad del Rosario, Bogota, Colombia; 2013;11.

487. Catena-Dell’Osso M, Rotella F, Dell’Osso A, Fagiolini A, Marazziti D. Inflammation, Serotonin and Major Depression. Curr. Drug Targets . M. Catena-Dell’Osso, Department of Clinical and Experimental Medicine, University of Pisavia Roma, 67 I-56100 Pisa, Italy; 2013;14:571–7.

488. Chen X, Yang M, Cheng Y, Liu GJ, Zhang M. Narrow-band ultraviolet B phototherapy versus broad-band ultraviolet B or psoralen-ultraviolet A photochemotherapy for psoriasis. Cochrane database Syst. Rev. . 2013;10:CD009481.

489. Correr CJ, Rotta I, Teles T. S, Godoy RR, Riveros BS, Garcia MM, et al. Efficacy and safety of biologics in the treatment of moderate to severe psoriasis: a comprehensive meta-analysis of randomized controlled trials. Cad. Saude Publica . 2013;29:S17–31.

490. Coto-Segura P, Eiris-Salvado N, González-Lara L, Queiro-Silva R, Martinez-Camblor P, Maldonado-Seral C, et al. Psoriasis, psoriatic arthritis and type 2 diabetes mellitus: A systematic review and meta-analysis. Br. J. Dermatol. 2013;169:783–93.

491. Daudén E, Castañeda S, Suárez C, García-Campayo J, Blasco AJ, Aguilar MD, et al. Clinical practice guideline for an integrated approach to comorbidity in patients with psoriasis. J. Eur. Acad. Dermatology Venereol. . A.J. Blasco, Advanced Techniques in Health Services Research (TAISS), Madrid, Spain; 2013;27:1387–404.

492. Dávila-Fajardo CL, Swen JJ, Cabeza Barrera J, Guchelaar H-J. Genetic risk factors for drug-induced liver injury in rheumatoid arthritis patients using low-dose methotrexate. Pharmacogenomics . H.-J. Guchelaar, Department of Clinical Pharmacy and Toxicology, Leiden University Medical Center, NL 2300 RC Leiden, Netherlands; 2013;14:63–73.

493. Denadai R, Teixeira F V, Steinwurz F, Romiti R, Saad-Hossne R. Induction or exacerbation of psoriatic lesions during anti-TNF-α therapy for inflammatory bowel disease: A systematic literature review based on 222 cases. J. Crohn’s Colitis . R. Denadai, 17516-707, Marília, SP, Brazil; 2013;7:517–24.

494. Deng S, May BH, Zhang AL, Lu C, Xue CCL. Topical herbal medicine combined with pharmacotherapy for psoriasis: A systematic review and meta-analysis. Arch. Dermatol. Res. . C. Lu, Guangdong Provincial Academy of Chinese Medical Sciences, Guangdong Provincial Hospital of Chinese Medicine, Guangzhou 510120, China; 2013;305:179–89.

495. Deng S, May BH, Zhang AL, Lu C, Xue CCL. Plant extracts for the topical management of psoriasis: A systematic review and meta-analysis. Br. J. Dermatol. 2013;169:769–82.

496. Dobson R, Giovannoni G. Autoimmune disease in people with multiple sclerosis and their relatives: A systematic review and meta-analysis. J. Neurol. . R. Dobson, Blizard Institute, Barts and London School of Medicine and Dentistry, Queen Mary University of London, London E1 2AT, United Kingdom; 2013;260:1272–85.

497. Dodds MG, Salinger DH, Mandema J, Gibbs JP, Gibbs MA. Clinical trial simulation to inform phase 2: Comparison of concentrated vs. distributed First-in-patient study designs in Psoriasis. CPT Pharmacometrics Syst. Pharmacol. . M.A. Gibbs, Department of Pharmacokinetics and Drug Metabolism, Amgen, Seattle, WA, United States; 2013;2.

498. Dols A, Sienaert P, Van Gerven H, Schouws S, Stevens A, Kupka R, et al. The prevalence and management of side effects of lithium and anticonvulsants as mood stabilizers in bipolar disorder from a clinical perspective: A review. Int. Clin. Psychopharmacol. . A. Dols, Department of Psychiatry, GGZ in Geest, VU University Medical Center, 1075 BH Amsterdam, Netherlands; 2013;28:287–96.

499. Dommasch ED, Troxel AB, Gelfand JM. Major cardiovascular events associated with anti-IL 12/23 agents: A tale of two meta-analyses. J. Am. Acad. Dermatol. . E.D. Dommasch, Boston, MA 02118, United States; 2013;68:863–5.

500. dos Santos FK, Oyafuso MH, Kiill CP, Daflon-Gremião MP, Chorilli M. Nanotechnology-based drug delivery systems for treatment of hyperproliferative skin diseases - a review. Curr. Nanosci. . M. Chorilli, Araraquara, SP CEP 14801-902, Brazil; 2013;9:159–67.

501. Dowlatshahi EA, Wakkee M, Arends L, Nijsten T. Varying prevalence of depression in psoriasis according to assessment method: A systematic review and meta-analysis. J. Invest. Dermatol. . E.A. Dowlatshahi, Dermatology, Erasmus Medical Centre, Rotterdam, Netherlands; 2013;133:S99.

502. Dowlatshahi EA, Van Der Voort EAM, Arends LR, Nijsten T. Markers of systemic inflammation in psoriasis: A systematic review and meta-analysis. Br. J. Dermatol. 2013;169:266–82.

503. Enamandram M, Kimball AB. Psoriasis epidemiology: The interplay of genes and the environment. J. Invest. Dermatol. . A.B. Kimball, Department of Dermatology, Massachusetts General Hospital, Harvard Medical School, Boston, MA 02114, United States; 2013;133:287–9.

504. Erceg A, De Jong EMJG, Van De Kerkhof PCM, Seyger MMB. The efficacy of pulsed dye laser treatment for inflammatory skin diseases: A systematic review. J. Am. Acad. Dermatol. . A. Erceg, Department of Dermatology, Amphia Hospital, 4818 CK Breda, Netherlands; 2013;69:609–15.e8.

505. Famenini S, Wu JJ. Infliximab-induced psoriasis in treatment of Crohn’s disease-associated ankylosing spondylitis: Case report and review of 142 cases. J. Drugs Dermatology . J.J. Wu, Department of Dermatology, Kaiser Permanente Los Angeles Medical Center, Los Angeles, CA, United States; 2013;12:939–43.

506. Feldman SR, Burudpakdee C, Gala S, Mallya U. Systematic literature review of economic burden of chronic plaque psoriasis. Value Heal. . S.R. Feldman, Wake Forest University, Winston-Salem, United States; 2013;16:A504.

507. Femia AN, Vleugels RA, Callen JP. Cutaneous dermatomyositis: An updated review of treatment options and internal associations. Am. J. Clin. Dermatol. . J.P. Callen, Division of Dermatology, University of Louisville, School of Medicine, Louisville, KY, United States; 2013;14:291–313.

508. Flatz L, Conrad C. Role of T-cell-mediated inflammation in psoriasis: Pathogenesis and targeted therapy. Psoriasis Targets Ther. . C. Conrad, Department of Dermatology, University Hospital of Lausanne (CHUV), 1011 Lausanne, Switzerland; 2013;3:1–10.

509. Flint J, Gayed M, Schreiber K, Arthanari S, Nisar M, Khamashta M, et al. A systematic analysis of the safety of prescribing of anti-rheumatic, immunosuppressive and biologic drugs in men trying to conceive. Rheumatol. (United Kingdom) . J. Flint, Rheumatology, University College London, London, United Kingdom; 2013;52:i149.

510. Foltz IN, Karow M, Wasserman SM. Evolution and emergence of therapeutic monoclonal antibodies what cardiologists need to know. Circulation . I.N. Foltz, Amgen British Columbia, Burnaby, BC, V5A 1V7, Canada; 2013;127:2222–30.

511. Foulkes AC, Jorgensen A, Pirmohamed M, Griffiths CE, Warren RB. Systematic review of pharmacogenomics in psoriasis. J. Invest. Dermatol. . A.C. Foulkes, Dermatology Centre, University of Manchester, Manchester, United Kingdom; 2013;133:S88.

512. Frendl DM, Strom M, Ware Jr. JE. Patient reported health outcomes from well-controlled trials of biologic therapies: A systematic review. Pharmacoepidemiol. Drug Saf. . D.M. Frendl, Quantitative Health Sciences, Division of Outcomes Measurement Science, University of Massachusetts Medical School, Worcester, United States; 2013;22:436–7.

513. Furst DE, Fleischman R, Kalden J, Kavanaugh A, Sieper J, Mease P, et al. Documentation of off-label use of biologics in Rheumatoid Arthritis. Ann. Rheum. Dis. . D.E. Furst, Los Angeles, CA 90025, United States; 2013;72:ii35–51.

514. Gaeta M, Castelvecchio S, Ricci C, Pigatto P, Pellissero G, Cappato R. Role of psoriasis as independent predictor of cardiovascular disease: A meta-regression analysis. Int. J. Cardiol. . R. Cappato, Arrhythmia and Electrophysiology Center, I.R.C.C.S. Policlinico San Donato, 20097 San Donato Milanese, Milan, Italy; 2013;168:2282–8.

515. Gajinov ZT, Matić MB, Duran VD, Vučković N, Prcić ST, Vujanović LM. Drug-related pityriasis rubra pilaris with acantholysis. Vojnosanit. Pregl. . Z. T. Gajinov, Dermatovenereological Clinic, Clinical Centre of Vojvodina, Novi Sad, Serbia; 2013;70:871–3.

516. Galván-Banqueri M, Marín Gil R, Santos Ramos B, Bautista Paloma FJ. Biological treatments for moderate-to-severe psoriasis: Indirect comparison. J. Clin. Pharm. Ther. 2013;38:121–30.

517. Gamble R, Gilchrest B, Dellavalle R. The Journal of Investigative Dermatology’s experience in its first six months on Facebook. J. Invest. Dermatol. . R. Gamble, Dermatology, University of Colorado, Aurora, United States; 2013;133:S100.

518. Gan EY, Chong W-S, Tey HL. Therapeutic strategies in psoriasis patients with psoriatic arthritis: Focus on new agents. BioDrugs . E.Y. Gan, National Skin Centre, Singapore 308205, Singapore; 2013;27:359–73.

519. Garcês S, Demengeot J, Benito-Garcia E. The immunogenicity of anti-TNF therapy in immune-mediated inflammatory diseases: A systematic review of the literature with a meta-analysis. Ann. Rheum. Dis. . S. Garcês, Instituto Gulbenkian de Ciência, Lymphocyte Physiology Group, P-2781-901, Oeiras, Portugal; 2013;72:1947–55.

520. Georgakopoulou EA, Andreadis D, Arvanitidis E, Loumou P. Biologic agents and oral diseases - An update on clinical applications. Acta Dermatovenerologica Croat. . E. A. Georgakopoulou, 1st Dermatology Clinic, A. Sygros Hospital, Medical School, National and Kapodistrian University of Athens, 14232 N Ionia Athens, Greece; 2013;21:24–34.

521. Gisondi P, Girolomoni G. Impact of TNF-α antagonists on the quality of life in selected skin diseases. G. Ital. di Dermatologia e Venereol. . P. Gisondi, Department of Medicine, Section of Dermatology and Venereology, University of Verona, I-37126 Verona, Italy; 2013;148:243–8.

522. Gokhale S, Jalapu A, Mallya U, Mpofu S. Assessment of cost-effectiveness models for biologics in the management of psoriatic arthritis. Value Heal. . S. Gokhale, Novartis Healthcare Pvt. Ltd., Hyderabad, India; 2013;16:A717.

523. Gomez-Reino JJ. What’s new: Psoriatic arthritis. Ann. Rheum. Dis. . J.J. Gomez-Reino, Rheumatology, Hospital Clinico Unversitario, Santiago, Chile; 2013;72.

524. Griffith JW, Luster AD. Targeting cells in motion: Migrating toward improved therapies. Eur. J. Immunol. . A.D. Luster, Center for Immunology and Inflammatory Diseases, Massachusetts General Hospital and Harvard Medical School, Boston, MA, United States; 2013;43:1430–5.

525. Gröber U, Spitz J, Reichrath J, Kisters K, Holick MF. Vitamin D: Update 2013 - From rickets prophylaxis to general preventive healthcare. Dermatoendocrinol. . U. Gröber, Academy for Micronutrient Medicine, Essen, Germany; 2013;5:331–47.

526. Gu W-J, Weng C-L, Zhao Y-T, Liu Q-H, Yin R-X. Psoriasis and risk of cardiovascular disease: A meta-analysis of cohort studies. Int. J. Cardiol. . R.-X. Yin, Department of Cardiology, First Affiliated Hospital, Guangxi Medical University, Nanning 530021, Guangxi, China; 2013;168:4992–6.

527. Gustafson CJ, Watkins C, Hix E, Feldman SR. Combination therapy in psoriasis: An evidence-based review. Am. J. Clin. Dermatol. . S.R. Feldman, Center for Dermatology Research, Department of Dermatology, Wake Forest University School of Medicine, Winston-Salem, NC 27157-1071, United States; 2013;14:9–25.

528. Gysin C. Indications of pediatric tonsillectomy. ORL . C. Gysin, Division of Pediatric Otolaryngology, University Children’s Hospital, Zurich, Switzerland; 2013;75:193–202.

529. Haddad A, Somaily M, Johnson SR, Fazelzad R, Kron AT, Chau C, et al. Psoriatic arthritis mutilans: Clinical and radiographic definitions. A systematic review. Arthritis Rheum. . A. Haddad, University of Toronto, Toronto Western Hospital, Toronto, Canada; 2013;65:S152.

530. Han Y, Liu T, Lu L. Apolipoprotein E Gene Polymorphism in Psoriasis: A Meta-analysis. Arch. Med. Res. . L. Lu, Department of Physiology, Kunming Medical University, Chenggong District, Kunming, Yunnan 650500, China; 2013;44:46–53.

531. Harsono D, Finelt N, Castano EB, Tonnesen MG, Clark RAF. Curcumin myths or wonders? A systematic analysis of in vitro studies. Wound Repair Regen. . D. Harsono, Department of Biomedical Engineering, Stony Brook University, Stony Brook, United States; 2013;21:335–6.

532. Hendriks AGM, Keijsers RRMC, De Jong EMGJ, Seyger MMB, Van De Kerkhof PCM. Combinations of classical time-honoured topicals in plaque psoriasis: A systematic review. J. Eur. Acad. Dermatology Venereol. . A.G.M. Hendriks, Department of Dermatology, Radboud University, Nijmegen Medical Centre, Nijmegen, Netherlands; 2013;27:399–410.

533. Hendriks AGM, Keijsers RRMC, De Jong EMGJ, Seyger MMB, Van De Kerkhof PCM. Efficacy and safety of combinations of first-line topical treatments in chronic plaque psoriasis: A systematic literature review. J. Eur. Acad. Dermatology Venereol. . A.G.M. Hendriks, Department of Dermatology, Radboud University, Nijmegen Medical Centre, Nijmegen, Netherlands; 2013;27:931–51.

534. Herrinton LJ, Harrold LR, Liu L, Raebel MA, Taharka A, Winthrop KL, et al. Association between anti-TNF-α therapy and interstitial lung disease. Pharmacoepidemiol. Drug Saf. . L.J. Herrinton, Division of Research, Kaiser Permanente, Oakland CA 94612, United States; 2013;22:394–402.

535. Hönigsmann H. Synergism between narrowband ultraviolet B phototherapy and etanercept for the treatment of plaque-type psoriasis. Br. J. Dermatol. . H. Hönigsmann, Department of Dermatology, Medical University of Vienna, A-1090 Vienna, Austria; 2013;169:4–5.

536. Horreau C, Pouplard C, Brenaut E, Barnetche T, Misery L, Cribier B, et al. Cardiovascular morbidity and mortality in psoriasis and psoriatic arthritis: A systematic literature review. J. Eur. Acad. Dermatology Venereol. . M.-A. Richard, Aix-Marseille University UMR 911, Dermatology Department, Timone Hospital, Marseille, France; 2013;27:12–29.

537. Hunt L, Emery P. Etanercept in the treatment of rheumatoid arthritis. Expert Opin. Biol. Ther. . P. Emery, NIHR Leeds Musculoskeletal Biomedical Research Unit, Leeds Teaching Hospitals NHS Trust, Leeds, United Kingdom; 2013;13:1441–50.

538. Huynh D, Kavanaugh A. Psoriatic arthritis: Current therapy and future directions. Expert Opin. Pharmacother. . A. Kavanaugh, University of California, Division of Rheumatology, Allergy and Immunology, San Diego, CA 92093-0943, United States; 2013;14:1755–64.

539. Ingham M, Ellis L, Bolge S. Number needed to treat, cost per responder and budget impact of TNF inhibitors in the treatment of psoriatic arthritis. Ann. Rheum. Dis. . M. Ingham, Janssen Scientific Affairs, LLC, Horsham, United States; 2013;71.

6540. Inkeles M, Modlin R, Pellegrini M. Meta-analysis of skin disease microarray datasets: Diagnosis, classification and insight into pathogenesis. J. Invest. Dermatol. . M. Inkeles, Bioinformatics, University of California, Los Angeles, United States; 2013;133:S141.

541. Ioannidis JPA, Karassa FB, Druyts E, Thorlund K, Mills EJ. Biologic agents in rheumatology: Unmet issues after 200 trials and $200 billion sales. Nat. Rev. Rheumatol. . J.P.A. Ioannidis, Stanford Prevention Research Centre, Department of Health Research and Policy, Stanford University School of Medicine, Stanford, CA 94305, United States; 2013;9:665–73.

542. Jamnitski A, Symmons D, Peters MJL, Sattar N, Mcilnnes I, Nurmohamed MT. Cardiovascular comorbidities in patients with psoriatic arthritis: A systematic review. Ann. Rheum. Dis. . M.T. Nurmohamed, Department of Rheumatology, Jan Van Breemen Research Institute/READE, 1056 AB Amsterdam, Netherlands; 2013;72:211–6.

543. Jani M, Chinoy H, Lamb JA, Wedderburn L, Vencovsky J, Danko K, et al. Investigation of idiopathic inflammatory myopathy for shared genetic risk factors with other autoimmune diseases: Results from the european myositis network. Lancet . M. Jani, Centre for Musculoskeletal Research, Institute of Inflammation and Repair, University of Manchester, Manchester, United Kingdom; 2013;381:S56.

544. Jia Y, Qin HJ, Zhang JX, Liu XL, Li LJ. Association of the tumour necrosis factor-α polymorphisms rs361525 and rs1800629 with susceptibility to psoriasis: A meta-analysis. Clin. Exp. Dermatol. . L.J. Li, First Affiliated Hospital, Henan University of Science and Technology, Luoyang 471003, China; 2013;38:836–44.

545. Kaffenberger BH, Wong HK, Jarjour W, Andritsos LA. Remission of psoriasis after allogeneic, but not autologous, hematopoietic stem-cell transplantation. J. Am. Acad. Dermatol. . B.H. Kaffenberger, Dermatology, Ohio State University, College of Medicine, Gahanna, OH 43216, United States; 2013;68:489–92.

546. Kannan S, Heller MM, Lee ES, Koo JY. The role of tumor necrosis factor-alpha and other cytokines in depression: What dermatologists should know. J. Dermatolog. Treat. . S. Kannan, Medical College of Georgia, School of Medicine, UCSF Psoriasis and Skin Treatment Center, San Francisco, CA 94118, United States; 2013;24:148–52.

547. Karim R, Sykakis E, Lightman S, Fraser-Bell S. Interventions for the treatment of uveitic macular edema: A systematic review and meta-analysis. Clin. Ophthalmol. . R. Karim, University of Sydney, Camperdown, NSW 2006, Australia; 2013;7:1109–44.

548. Kiliç E, Kiliç G, Akgül O, Akgöl G, Özgöçmen S. The reported adverse effects related to biological agents used for the treatment of rheumatic diseases in Turkey. Turkish J. Rheumatol. . E. Kiliç, Erciyes Üniversitesi, Fiziksel Tip ve Rehabilitasyon Anabilim Dali, Romatoloji Bilim Dali, 38039 Talas, Kayseri, Turkey; 2013;28:149–62.

549. Kirino Y, Bertsias G, Ishigatsubo Y, Mizuki N, Tugal-Tutkun I, Seyahi E, et al. Genome-wide association analysis identifies new susceptibility loci for Behçet’s disease and epistasis between HLA-B*51 and ERAP1. Nat. Genet. . D.L. Kastner, Inflammatory Disease Section, Medical Genetics Branch, National Human Genome Research Institute, Bethesda, MD, United States; 2013;45:202–7.

550. Krnjevic Pezic G, Tomic Babic L, Ceovic R, Kostovic K, Lazic Mosler E. Report of the prevalence of metabolic syndrome in Croatian psoriasis patients. J. Eur. Acad. Dermatology Venereol. . G. Krnjevic Pezic, Naftalan - Special Hospital for Skin and Rheumatic Diseases, Ivanic Grad, Croatia; 2013;27:33–4.

551. Kupetsky EA, Keller M. Psoriasis vulgaris: An evidence-based guide for primary care. J. Am. Board Fam. Med. . E.A. Kupetsky, Department of Dermatology and Cutaneous Biology, Thomas Jefferson University, Philadelphia, PA 19107, United States; 2013;26:787–801.

552. Ladizinski B, Heller MM, Bhutani T, Zitelli KB, Koo JYM. Progressive multifocal leukoencephalopathy and reversible progressive leukoencephalopathy syndrome in dermatologic therapy. J. Drugs Dermatology . B. Ladizinski, Duke University Medical Center, Department of Dermatology, Durham, NC, United States; 2013;12:e20–4.

553. Laftah Z, Arkir Z, Agius E, Desai N. Infliximab for hidradenitis suppurativa; should we be measuring antibody levels? Br. J. Dermatol. . Z. Laftah, St John’s Institute of Dermatology, Guy's and St Thomas' NHS Foundation Trust, London, United Kingdom; 2013;169:5–6.

554. Lang UE, Borgwardt S. Molecular mechanisms of depression: Perspectives on new treatment strategies. Cell. Physiol. Biochem. . University Hospital of Basel, Department of Psychiatry and Psychotherapy, Basel, Switzerland; 2013;31:761–77.

555. Lee YH, Song GG. Associations between interleukin-23R and interleukin-12B polymorphisms and psoriasis susceptibility: A meta-analysis. Immunol. Invest. . Y.H. Lee, Korea University College of Medicine, Department of Internal Medicine, Korea University Anam Hospital, Seongbuk-gu, Seoul 136-705, South Korea; 2013;42:726–36.

556. Lemos LLP, Reis CAL, Barbosa MM, Oliveira H, Almeida AM, Acurcio FA. Treating psoriatic arthritis with biological disease modifying antirheumatic drugs: Systematic review and meta-analysis to evaluate efficacy and safety. Value Heal. . L.L.P. Lemos, Universidade Federal de Minas Gerais, Belo Horizonte, Brazil; 2013;16:A715.

557. Liu JL, Zhang SQ, Zeng HM. ApaI, BsmI, FokI and TaqI polymorphisms in the vitamin D receptor (VDR) gene and the risk of psoriasis: A meta-analysis. J. Eur. Acad. Dermatology Venereol. . J.L. Liu, Department of Dermatology, Hainan Nongken General Hospital, Haikou, Hainan, China; 2013;27:739–46.

558. Liu T, Han Y, Lu L. Angiotensin-converting enzyme gene polymorphisms and the risk of psoriasis: A meta-analysis. Clin. Exp. Dermatol. . L. Lu, Department of Physiology, Kunming Medical University Yunnan, Chenggong District, Kunming, Yunnan, 650500, China; 2013;38:352–9.

559. Lopez A, Billioud V, Peyrin-Biroulet C, Peyrin-Biroulet L. Adherence to anti-TNF therapy in inflammatory bowel diseases: A systematic review. Inflamm. Bowel Dis. . L. Peyrin-Biroulet, Inserm U954, Department of Hepato-Gastroenterology, University Hospital of Nancy-Brabois, 54511 Vandoeuvre-lès-Nancy, France; 2013;19:1528–33.

560. Lu Y, Kane S, Chen H, Leon A, Levin E, Nguyen T, et al. The role of 39 psoriasis risk variants on age of psoriasis onset. ISRN Dermatol. . W. Liao, Department of Dermatology, University of California San Francisco, San Francisco, CA 94143-0808, United States; 2013;2013.

561. Ma C, Harskamp CT, Armstrong EJ, Armstrong AW. The association between psoriasis and dyslipidaemia: A systematic review. Br. J. Dermatol. . A.W. Armstrong, Department of Dermatology, University of California Davis, Sacramento, CA, United States; 2013;168:486–95.

562. Machet L, Samimi M, Delage M, Paintaud G, Maruani A. Systematic review of the efficacy and adverse events associated with infliximab treatment of hidradenitis suppurativa in patients with coexistent inflammatory diseases. J. Am. Acad. Dermatol. . L. Machet, Service de Dermatologie, CHU de Tours, F-37044 Tours cedex 01, France; 2013;69:649–50.

563. Makhija K, Karunakaran S. The role of inflammatory cytokines on the aetiopathogenesis of depression. Aust. N. Z. J. Psychiatry . K. Makhija, Towsnville Hospital, Dauglas, QLD 4814, Australia; 2013;47:828–39.

564. Makri OE, Georgalas I, Georgakopoulos CD. Drug-induced macular edema. Drugs . C.D. Georgakopoulos, Department of Ophthalmology, Medical School, University of Patras Rio, 26504 Patras, Greece; 2013;73:789–802.

565. Manczinger M, Kemény L. Novel factors in the pathogenesis of psoriasis and potential drug candidates are found with systems biology approach. PLoS One . Department of Dermatology and Allergology, University of Szeged, Szeged, Hungary; 2013;8.

566. Mandema J, Peterson M, Ahadieh S, Tan H, Krishnaswami S, Wolk R, et al. Evaluation of the impact of body weight on the efficacy of biologic therapies for the treatment of psoriasis: A dose-response meta-analysis. J. Eur. Acad. Dermatology Venereol. . P. Gupta, Pfizer Inc, Groton, United States; 2013;27:11–2.

567. Maneiro JR, Salgado E, Gomez-Reino JJ. Immunogenicity of monoclonal antibodies against tumor necrosis factor used in chronic immune-mediated Inflammatory conditions: systematic review and meta-analysis. JAMA Intern. Med. . 2013;173:1416–28.

568. Mason AR, Mason J, Cork M, Dooley G, Hancock H. Topical treatments for chronic plaque psoriasis. Cochrane database Syst. Rev. . A.R. Mason, Centre for Health Economics, The University of York, York, UK.; 2013;3:CD005028.

569. Mason A, Mason J, Cork M, Hancock H, Dooley G. Topical treatments for chronic plaque psoriasis: An abridged Cochrane Systematic Review. J. Am. Acad. Dermatol. . Elsevier Inc; 2013;69:799–807.

570. Mezentsev A V, Bruskin SA, Soboleva AG, Sobolev V V, Piruzian ES. Pharmacological control of receptor of ddvanced glycation end-products and its biological effects in psoriasis. Int. J. Biomed. Sci. . E. S. Piruzian, Moscow 119991, Russian Federation; 2013;9:112–22.

571. Miller IM, Skaaby T, Ellervik C, Jemec GBE. Quantifying cardiovascular disease risk factors in patients with psoriasis: A meta-analysis. Br. J. Dermatol. . I.M. Miller, Department of Dermatology, Roskilde Hospital, 4000 Roskilde, Denmark; 2013;169:1180–7.

572. Miller IM, Ellervik C, Yazdanyar S, Jemec GBE. Meta-analysis of psoriasis, cardiovascular disease, and associated risk factors. J. Am. Acad. Dermatol. 2013;69:1014–24.

573. Moon H-S, Mizara A, McBride SR. Psoriasis and psycho-dermatology. Dermatol. Ther. (Heidelb). . S. R. McBride, Department of Dermatology, Royal Free NHS Foundation Trust, London NW3 2QG, United Kingdom; 2013;3:117–30.

574. Mortazavi H, Aghazadeh N, Ghiasi M, Lajevardi V. A review of three systemic retinoids in dermatology: Acitretin, isotretinoin and bexarotene. Iran. J. Dermatology . H. Mortazavi, Razi Hospital, Tehran, Iran; 2013;16:144–58.

575. Mouthon L, Fermand J-P, Gottenberg J-E. Management of secondary immune deficiencies: What is the role of immunoglobulins? Curr. Opin. Allergy Clin. Immunol. . Department of Internal Medicine, Cochin Hospital, Université Paris Descartes, 75679 Paris Cedex 14, France; 2013;13:S56–67.

576. Mrowietz U, Domm S. Systemic steroids in the treatment of psoriasis: What is fact, what is fiction? J. Eur. Acad. Dermatology Venereol. . U. Mrowietz, Department of Dermatology, Campus Kiel, University Medical Center Schleswig-Holstein, Germany; 2013;27:1022–5.

577. Muftin Z, Thompson AR. A systematic review of self-help for disfigurement: Effectiveness, usability, and acceptability. Body Image . Z. Muftin; 2013;10:442–50.

578. Mulekar S V, Isedeh P. Surgical interventions for vitiligo: An evidence-based review. Br. J. Dermatol. . S.V. Mulekar, National Center for Vitiligo, Riyadh, 11372, Saudi Arabia; 2013;169:57–66.

579. Mustafa AA, Al-Hoqail IA. Biologic systemic therapy for moderate-to-severe psoriasis: A review. J. Taibah Univ. Med. Sci. . A.A. Mustafa, Deptartment of Basic Medical Sciences, King Saud Bin Abdulaziz University for Health Sciences, King Fahad Medical City, Riyadh 11525, P.O. Box 59046, Saudi Arabia; 2013;8:142–50.

580. Nair RP, Tsoi LC, Stuart PE, Chandran V, Tejasvi T, Ellinghaus E, et al. Meta-analysis of psoriasis and psoriatic arthritis identifies three new susceptibility loci. J. Invest. Dermatol. . R.P. Nair, Univ Michigan, Ann Arbor, United States; 2013;133:S136.

581. Nast A, Schmitt J. Physician global assessment (PGA) and psoriasis area and severity index (PASI): Why do both? A systematic analysis of randomized controlled trials of biologic agents for moderate to severe plaque psoriasis. J. Am. Acad. Dermatol. . A. Nast, Division of Evidence-Based Medicine (DEBM), Department of Dermatology Venerology and Allergy, Charité Universitätsmedizin, Berlin 10117, Germany; 2013;68:1040–1.

582. Nast A, Sporbeck B, Rosumeck S, Pathirana D, Jacobs A, Werner RN, et al. Which antipsoriatic drug has the fastest onset of action?-systematic review on the rapidity of the onset of action. J. Invest. Dermatol. . A. Nast, Division of Evidence Based Medicine (DEBM), Department of Dermatology, Charité-Universitätsmedizin Berlin, Berlin 10117, Germany; 2013;133:1963–70.

583. Ncube B, Ndhlala AR, Okem A, Van Staden J. Hypoxis (Hypoxidaceae) in African traditional medicine. J. Ethnopharmacol. . J. Van Staden, Research Centre for Plant Growth and Development, School of Life Sciences, University of KwaZulu-Natal Pietermaritzburg, Scottsvill 3209, South Africa; 2013;150:818–27.

584. Neef HC, Riebschleger MP, Adler J. Meta-analysis: Rapid infliximab infusions are safe. Aliment. Pharmacol. Ther. . J. Adler, Division of Pediatric Gastroenterology, Department of Pediatrics and Communicable Diseases, University of Michigan, Ann Arbor, MI 48109-5718, United States; 2013;38:365–76.

585. Odom D, Brogan A, Talbird SE, Schenkel B. A network meta-analysis of randomized, controlled trials of ustekinumab and adalimumab for moderate-to-severe psoriasis. Value Heal. . D. Odom, RTI Health Solutions, Research Triangle Park, United States; 2013;16:A112.

586. Pan M, Heinecke G, Bernardo S, Tsui C, Levitt J. Urea: A comprehensive review of the clinical literature. Dermatol. Online J. . J. Levitt, Department of Dermatology, New York, NY 10029, United States; 2013;19.

587. Parisi R, Symmons DPM, Griffiths CEM, Ashcroft DM. Global epidemiology of psoriasis: A systematic review of incidence and prevalence. J. Invest. Dermatol. . D.M. Ashcroft, School of Pharmacy and Pharmaceutical Sciences, University of Manchester, Stopford Building, Manchester M13 9PT, United Kingdom; 2013;133:377–85.

588. Parkins G, Burden AD. What is the optimal topical treatment for limited plaque psoriasis? Br. J. Dermatol. . Department of Dermatology, Western Infirmary, Glasgow G11 6NT, United Kingdom; 2013;168:925–6.

589. Paudyal V, Watson MC, Sach T, Porteous T, Bond CM, Wright DJ, et al. Are pharmacy-based minor ailment schemes a substitute for other service providers? A systematic review. Br. J. Gen. Pract. . M.C. Watson, Academic Primary Care, University of Aberdeen, Polwarth Building, Aberdeen, AB25 2ZD, United Kingdom; 2013;63:e472–81.

590. Petrof G, Almaani N, Archer CB, Griffiths WAD, Smith CH. A systematic review of the literature on the treatment of pityriasis rubra pilaris type 1 with TNF-antagonists. J. Eur. Acad. Dermatology Venereol. . G. Petrof, St John’s Institute of Dermatology, Guy's and St Thomas' NHS Foundation Trust, London, United Kingdom; 2013;27:e131–5.

591. Pietrzak A, Bartosińska J, Chodorowska G, Szepietowski JC, Paluszkiewicz P, Schwartz RA. Cardiovascular aspects of psoriasis: An updated review. Int. J. Dermatol. . J. Bartosińska, Department of Dermatology, Venereology and Pediatric Dermatology, Medical University of Lublin, 20-080 Lublin, Poland; 2013;52:153–62.

592. Poddubnyy D, Rudwaleit M. Adalimumab for the treatment of ankylosing spondylitis and nonradiographic axial spondyloarthritis - A five-year update. Expert Opin. Biol. Ther. . M. Rudwaleit, Endokrinologikum Berlin, Rheumatologie, Berlin, Germany; 2013;13:1599–611.

593. Pouplard C, Brenaut E, Horreau C, Barnetche T, Misery L, Richard M-A, et al. Risk of cancer in psoriasis: A systematic review and meta-analysis of epidemiological studies. J. Eur. Acad. Dermatology Venereol. . C. Paul, Dermatology Department, Paul Sabatier University, INSERM 1056, Toulouse, France; 2013;27:36–46.

594. Pride HB, Tollefson M, Silverman R. What’s new in pediatric dermatology?: Part II. Treatment. J. Am. Acad. Dermatol. . H.B. Pride, Department of Dermatology, Geisinger Medical Center, Danville, PA 17822-5206, United States; 2013;68:899.e1–899.e11.

595. Qiu Z-X, Zhang K, Qiu X-S, Zhou M, Li W-M. CD226 Gly307Ser association with multiple autoimmune diseases: A meta-analysis. Hum. Immunol. . W.-M. Li, Centers for Disease Control and Prevention, Changshou, Chongqing 401220, China; 2013;74:249–55.

596. Rashidi A, Fisher SI. Therapy-related acute promyelocytic leukemia: A systematic review. Med. Oncol. . A. Rashidi, Department of Internal Medicine, Eastern Virginia Medical School, Norfolk, VA 23507, United States; 2013;30.

597. Remmers EF, Kirino Y, Bertsias G, Ombrello MJ, Wood G, Ishigatsubo Y, et al. PW03-011 New Behc¸et’s loci and gene-gene interactions. Pediatr. Rheumatol. . E.F. Remmers, Inflammatory Dis Section/MGB, NHGRI, Bethesda, United States; 2013;11.

598. Richard M-A, Barnetche T, Horreau C, Brenaut E, Pouplard C, Aractingi S, et al. Psoriasis, cardiovascular events, cancer risk and alcohol use: Evidence-based recommendations based on systematic review and expert opinion. J. Eur. Acad. Dermatology Venereol. . M.-A. Richard, Aix-Marseille University, Dermatology Department, Timone Hospital, Marseille, France; 2013;27:2–11.

599. Richard M-A, Paul C. Cardiovascular morbidity, risk of cancer and alcohol abuse in psoriasis: Systematic literature reviews and expert opinion. J. Eur. Acad. Dermatology Venereol. . Aix-Marseille University UMR 911, Dermatology Department, Timone Hospital, Marseille, France; 2013;27:1.

600. Riveros BS, Rotta I, Garcia M, Souza TT, Godoy RR, Gonc¸alves P, et al. Multi-criteria benefit-risk assessment of biological agents in the treatment of moderate to severe psoriais: A stochastical approach. Value Heal. . B.S. Riveros, Universidade Federal do Paraná, Curitiba, Brazil; 2013;16:A727.

601. Rocha LK, Romitti R, Shinjo S, Neto ML, Carvalho J, Criado P. Cutaneous manifestations and comorbidities in 60 cases of Takayasu arteritis. J. Rheumatol. . L.K. Rocha, Hospital Das Clínicas, USP, Dermatology, 500 São Paulo, São Paulo 05409-000, Brazil; 2013;40:734–8.

602. Rosado-Buzzo A, Mould-Quevedo JF, Tang B, Gutierrez-Ardila M V, Vargas Zea N. Burden of disease associated with psoriasis in colombia. Value Heal. . A. Rosado-Buzzo, Links and Links S.A, de C.V., Mexico City, Mexico; 2013;16:A116.

603. Salinger DH, Mandema JW, Newmark RD, Gibbs MA. Model-based meta-analysis informs phase 3 head-to-head trial simulations of brodalumab and competitors in psoriasis. J. Pharmacokinet. Pharmacodyn. . D.H. Salinger, Amgen Inc., Seattle, United States; 2013;40:S45–6.

604. Samarasekera EJ, Sawyer L, Wonderling D, Tucker R, Smith CH. Topical therapies for the treatment of plaque psoriasis: Systematic review and network meta-analyses. Br. J. Dermatol. . C.H. Smith, Division of Medicine and Molecular Genetics, St John’s Institute of Dermatology Guy's Hospital, London SE1 9RT, United Kingdom; 2013;168:954–67.

605. Samarasekera EJ, Neilson JM, Warren RB, Parnham J, Smith CH. Incidence of cardiovascular disease in individuals with psoriasis: a systematic review and meta-analysis. J. Invest. Dermatol. . Elsevier Masson SAS; 2013;133:2340–6.

606. Sandmann FG, Franken MG, Steenhoek A, Koopmanschap MA. Do reassessments reduce the uncertainty of decision making? Reviewing reimbursement reports and economic evaluations of three expensive drugs over time. Health Policy (New. York). . F.G. Sandmann, Institute of Health Policy and Management (iBMG), Erasmus University Rotterdam, 3000 DR Rotterdam, Netherlands; 2013;112:285–96.

607. Saraceno R, Bavetta M, Zangrilli A, Chiricozzi A, Potenza C, Chimenti S, et al. Adalimumab in the treatment of plaque-type psoriasis and psoriatic arthritis. Expert Opin. Biol. Ther. . R. Saraceno, U.O.C. of Dermatology, University of Rome Tor Vergata, 00133, Rome, Italy; 2013;13:1325–34.

608. Savage LJ, McGonagle DG. The Role of Biological and Small Molecule Therapy in the Management of Psoriatic Arthritis. Biol. Ther. . L. J. Savage, Leeds Institute for Rheumatic and Musculoskeletal Medicine, University of Leeds, Chapel Allerton Hospital, Leeds, West Yorkshire, United Kingdom; 2013;3:61–81.

609. Sawyer L, Samarasekera EJ, Wonderling D, Smith CH. Topical therapies for the treatment of localized plaque psoriasis in primary care: A cost-effectiveness analysis. Br. J. Dermatol. . L. Sawyer, Symmetron Limited Kinetic Centre, Borehamwood, Hertfordshire WD6 4PJ, United Kingdom; 2013;168:1095–105.

610. Schiff M. Subcutaneous abatacept for the treatment of rheumatoid arthritis. Rheumatol. (United Kingdom) . M. Schiff, University of Colorado, School of Medicine, Greenwood Village, CO 80111, United States; 2013;52:986–97.

611. Segaert S, Røpke M. The biological rationale for use of vitamin D analogs in combination with corticosteroids for the topical treatment of plaque psoriasis. J. Drugs Dermatology . S. Segaert, University Hospital Leuven, Leuven, Belgium; 2013;12:e129–37.

612. Sikirica S. Accounting for partial responders in cost-effectiveness modeling of biologics for psoriasis. Value Heal. . S. Sikirica, Thomas Jefferson University, Philadelphia, United States; 2013;16:A27–8.

613. Sivamani RK, Goodarzi H, Garcia MS, Raychaudhuri SP, Wehrli LN, Ono Y, et al. Biologic therapies in the treatment of psoriasis: A comprehensive evidence-based basic science and clinical review and a practical guide to tuberculosis monitoring. Clin. Rev. Allergy Immunol. . E. Maverakis, Department of Dermatology, School of Medicine, University of California, Davis, Sacramento, CA 95816, United States; 2013;44:121–40.

614. Sokołowska-Wojdyło M, Ługowska-Umer H, MacIejewska-Radomska A. Oral retinoids and rexinoids in cutaneous T-cell lymphomas. Postep. Dermatologii i Alergol. . M. Sokołowska-Wojdyło, Department of Dermatology, Venereology and Allergology, Medical University of Gdansk, 80-210 Gdansk, Poland; 2013;30:19–29.

615. Song GG, Kim J-H, Lee YH. Association between the LCE3C-LCE3B deletion polymorphism and susceptibility to psoriasis: A meta-analysis of published studies. Genet. Test. Mol. Biomarkers . Y.H. Lee, Department of Internal Medicine, Korea University Anam Hospital, Korea University College of Medicine, Seongbuk-gu, Seoul 136-705, South Korea; 2013;17:572–7.

616. Stefanic M, Rucevic I, Barisic-Drusko V. Meta-analysis of vitamin D receptor polymorphisms and psoriasis risk. Int. J. Dermatol. . M. Stefanic, Clinical Institute of Nuclear Medicine and, Radiation Protection, Osijek University Hospital, 31 000 Osijek, Croatia; 2013;52:705–10.

617. Stolwijk C, Van Tubergen A, Castillo-Ortiz JD, Boonen A. Prevalence of extra-articular manifestations in patients with ankylosing spondylitis: A systematic review and meta-regression analysis. Ann. Rheum. Dis. . C. Stolwijk, Rheumatology, Maastricht University Medical Center, Maastricht, Netherlands; 2013;72.

618. Stolwijk C, van Tubergen A, Castillo-Ortiz JD, Boonen A. Prevalence of extra-articular manifestations in patients with ankylosing spondylitis: A systematic review and meta-analysis. Ann. Rheum. Dis. . C. Stolwijk, Department of Medicine, Division of Rheumatology, Maastricht University Medical Center; School for P,; 2013;

619. Strohal R, Chimenti S, Vena GA, Girolomoni G. Etanercept provides an effective, safe and flexible short- and long-term treatment regimen for moderate-to-severe psoriasis: a systematic review of current evidence. J. Dermatolog. Treat. . 2013;24:199–208.

620. Stuart ME, Shrite SA, Gandra SR. Systematic safety review of five biologic antirheumatic drugs. Ann. Rheum. Dis. . M.E. Stuart, University of Washington, School of Medicine, Seattle, United States; 2013;71.

621. Suarez-Farinas M, Belasco J, Sullivan T, Arbeit R, Krueger JG. Treatment of psoriasis patients with IMO-3100 shows improvement in gene expression patterns of meta-analysis derived-3 transcriptome and IL-17 pathway. Arthritis Rheum. . M. Suarez-Farinas, Rockefeller University, New York, United States; 2013;65:S495.

622. Suwandy F, Van Der Meijden WI. Smoking and the skin. Ned. Tijdschr. voor Dermatologie en Venereol. . W.I. Van Der Meijden, Afdeling Dermatologie, Havenziekenhuis, Rotterdam, Netherlands; 2013;23:666–71.

623. Tablazon ILD, Al-Dabagh A, Davis SA, Feldman SR. Risk of cardiovascular disorders in psoriasis patients: Current and future. Am. J. Clin. Dermatol. . S.A. Davis, Department of Dermatology, Center for Dermatology Research, Wake Forest School of Medicine, Winston-Salem, NC 27157-1071, United States; 2013;14:1–7.

624. Takeuchi M. A systematic review of biologics for the treatment of noninfectious uveitis. Immunotherapy . M. Takeuchi, Department of Ophthalmology, National Defense Medical College, Saitama, Japan; 2013;5:91–102.

625. Tempark T, Lueangarun S, Chatproedprai S, Wananukul S. Flood-related skin diseases: A literature review. Int. J. Dermatol. . T. Tempark, Department of Pediatrics, King Chulalongkorn Memorial Hospital, Bangkok 10330, Thailand; 2013;52:1168–76.

626. Thorneloe RJ, Bundy C, Griffiths CEM, Ashcroft DM, Cordingley L. Adherence to medication in patients with psoriasis: A systematic literature review. Br. J. Dermatol. . R.J. Thorneloe, Dermatology Research Centre, Institute of Inflammation and Repair, University of Manchester, Manchester, United Kingdom; 2013;168:20–31.

627. Tian S, Suárez-Fariñas M. Multi-TGDR: A regularization method for multi-class classification in microarray experiments. PLoS One . Division of Clinical Epidemiology, First Hospital of the Jilin University, Changchun, Jilin, China; 2013;8.

628. Tseng HW, Lin HS, Lam HC. Co-morbidities in psoriasis: A hospital-based case-control study. J. Eur. Acad. Dermatology Venereol. . H.C. Lam, Division of Endocrinology and Metabolism, Department of Internal Medicine, Kaohsiung Veterans General Hospital, Kaohsiung, Taiwan; 2013;27:1417–25.

629. Tzellos T, Kyrgidis A, Trigoni A, Zouboulis CC. Association of anti-IL-12/23 biologic agents ustekinumab and briakinumab with major adverse cardiovascular events. J. Eur. Acad. Dermatology Venereol. . C.C. Zouboulis, Division of Evidenced-Based Dermatology, Departments of Dermatology, Venereology, Allergology and Immunology, Dessau Medical Center, Dessau, Germany; 2013;27:1586–7.

630. Tzellos T, Kyrgidis A, Zouboulis CC. Re-evaluation of the risk for major adverse cardiovascular events in patients treated with anti-IL-12/23 biological agents for chronic plaque psoriasis: A meta-analysis of randomized controlled trials. J. Eur. Acad. Dermatology Venereol. 2013;27:622–7.

631. Valencia A, Hernandez A, Puig A. A cost-effectiveness model comparing sub-cutaneous biologic treatment for severe plaque psoriasis in Mexico. Value Heal. . A. Valencia, Janssen, Mexico City, Mexico; 2013;16:A726.

632. Van Den Bosch F. How to treat psoriatic arthritis? Ann. Rheum. Dis. . F. Van Den Bosch, Rheumatology, Ghent University Hospital, Gent, Belgium; 2013;71.

633. Vemer P, Rutten-Van Mölken MPMH. The road not taken: Transferability issues in multinational trials. Pharmacoeconomics . P. Vemer, Institute for Medical Technology Assessment (IMTA), Erasmus University Rotterdam, 3000 DR Rotterdam, Netherlands; 2013;31:863–76.

634. Wang X-Q, Zhang K, Xiong D. Toll-like receptor 4 polymorphisms and susceptibility to multiple autoimmune diseases: Evidence based on pooled analysis. Cent. J. Immunol. . K. Zhang, 563003 Zunyi, China; 2013;38:380–7.

635. Waters JP, Pober JS, Bradley JR. Tumour necrosis factor and cancer. J. Pathol. . J.R. Bradley, Addenbrooke’s Hospital, Cambridge CB2 0QQ, United Kingdom; 2013;230:241–8.

636. Weigle N, Mcbane S. Psoriasis. Am. Fam. Physician . N. Weigle, Duke University School of Medicine, Durham, NC 27710, United States; 2013;87:626–33.

637. Westerberg DP, Voyack MJ. Onychomycosis: Current trends in diagnosis and treatment. Am. Fam. Physician . D. P. Westerberg, FAAFP, Cooper University Hospital, Camden, NJ 08103, United States; 2013;88:762–70.

638. Wong HK. Novel biomarkers, dysregulated epigenetics, and therapy in cutaneous t-cell lymphoma. Discov. Med. . H.K. Wong, Department of Internal Medicine, The Ohio State University, Gahanna, Ohio, 43230, United States; 2013;16:71–8.

639. Wong JW, Nguyen T V, Koo JYM. Primary psychiatric conditions: Dermatitis artefacta, trichotillomania and neurotic excoriations. Indian J. Dermatol. . J.W. Wong, Department of Dermatology, University of California, UCSF Psoriasis and Skin Treatment Center, San Francisco, CA 94118, United States; 2013;58:44–8.

640. Yao Q. Nucleotide-binding oligomerization domain containing 2: Structure, function, and diseases. Semin. Arthritis Rheum. . Q. Yao, Department of Rheumatic, Immunologic Diseases/A50, Cleveland Clinic, Cleveland, OH 44195, United States; 2013;43:125–30.

641. Yu JJ, Zhang CS, Zhang AL, May B, Xue CC, Lu C. Add-on effect of chinese herbal medicine bath to phototherapy for psoriasis vulgaris: A systematic review. Evidence-based Complement. Altern. Med. . C. Lu, Department of Dermatology, Second Clinical College, Guangzhou University of Chinese Medicine, Guangzhou 510120, China; 2013;2013.

642. Zhang D-Y, Ren W-M, Shi C-R, Mao H. Effectiveness and safety of compound glycyrrhizin combined with acitretin for psoriasis: A systematic review. Chinese J. Evidence-Based Med. . W.-M. Ren, Department of Dermatovenereology, The First Clinical Hospital of Lanzhou University, Lanzhou 730000, China; 2013;13:112–20.

643. Zhao YE, Ma JX, Hu L, Xiao SX, Zhao YL. Meta-analysis of the association between psoriasis and human leucocyte antigen-B. Br. J. Dermatol. 2013;169:417–27. 644. Zhu JQ, Li J, Qu HD, Chen XG, Wang H. Tumor necrosis factor-alpha gene promoter region single nucleotide polymorphism change the susceptibility to psoriasis vulgaris and psoriatic arthritis: A meta-analysis. Int. J. Rheum. Dis. . J.Q. Zhu, Department of Rheumatology, Nanfang Hospital Southern Medical University, Guangzhou, China; 2013;16:39.

645. Zhu J, Qu H, Chen X, Wang H, Li J. Single Nucleotide Polymorphisms in the Tumor Necrosis Factor-Alpha Gene Promoter Region Alter the Risk of Psoriasis Vulgaris and Psoriatic Arthritis: A Meta-Analysis. PLoS One . J. Li, Department of Rheumatology, Nanfang Hospital, Southern Medical University, Guangzhou, Guangdong, China; 2013;8.

646. Zhu K-J, Shi G, Zhang C, Li M, Zhu C-Y, Fan Y-M. Adiponectin levels in patients with psoriasis: A meta-analysis. J. Dermatol. . K.-J. Zhu, Department of Dermatology, Affiliated Hospital of Guangdong Medical College, Xiashan District, Zhanjiang, Guangdong 524001, China; 2013;40:438–42.

647. Zhu K-J, Zhang C, Li M, Zhu C-Y, Shi G, Fan Y-M. Leptin levels in patients with psoriasis: A meta-analysis. Clin. Exp. Dermatol. . K.-J. Zhu, Department of Dermatology, Affiliated Hospital of Guangdong Medical College, Xiashan district, Zhanjiang, Guangdong 524001, China; 2013;38:478–83.

648. Zhu KJ, Zhu CY, Shi G, Fan YM. Meta-analysis of IL12B polymorphisms (rs3212227, rs6887695) with psoriasis and psoriatic arthritis. Rheumatol. Int. 2013;33:1785–90.

649. Zhuang L, Ma W, Cai D, Zhong H, Sun Q. Associations between tumor necrosis factor-α polymorphisms and risk of psoriasis: A meta-analysis. PLoS One . Q. Sun, Department of Dermatology, Qilu Hospital, Shandong University, Jinan, China; 2013;8.

650. The Cochrane Database of Systematic Reviews - Issue 10 2013. J. Evid. Based. Med. . 2013;6:305–6.

**2012**

651. Akkineni R, Albert DA. Is there an optimal treatment strategy for disease-modifying-antirheumatic-drug naïve patients with rheumatoid arthritis? Arthritis Rheum. . R. Akkineni, Dartmouth Hitchcock Medical Center, Lebanon, United States; 2012;64:S403.

652. Almalag HM, Mangoni AA, Crilly MA. Methotrexate and risk of cardiovascular disease. Am. J. Cardiol. . H.M. Almalag, Aberdeen, United Kingdom; 2012;109:1383–4.

653. Archier E, Devaux S, Castela E, Gallini A, Aubin F, Le Maître M, et al. Ocular damage in patients with psoriasis treated by Psoralen UV-A therapy or Narrow band UVB therapy: A systematic literature review. J. Eur. Acad. Dermatology Venereol. . E. Archier, Aix-Marseille Univ, Dermatology Department, Timone Hospital, Marseille, France; 2012;26:32–5.

654. Archier E, Devaux S, Castela E, Gallini A, Aubin F, Le Maître M, et al. Efficacy of Psoralen UV-A therapy vs. Narrowband UV-B therapy in chronic plaque psoriasis: A systematic literature review. J. Eur. Acad. Dermatology Venereol. . E. Archier, Dermatology Department, Aix-Marseille Univ, Timone Hospital, Marseille, France; 2012;26:11–21.

655. Archier E, Devaux S, Castela E, Gallini A, Aubin F, Le Maître M, et al. Carcinogenic risks of Psoralen UV-A therapy and Narrowband UV-B therapy in chronic plaque psoriasis: A systematic literature review. J. Eur. Acad. Dermatology Venereol. . E. Archier, Dermatology Department, Aix-Marseille University, Timone Hospital, Marseille, France; 2012;26:22–31.

656. Armesto S, Coto-Segura P, Osuna CG, Camblor PM, Santos-Juanes J. Psoriasis and hypertension: A case-control study. J. Eur. Acad. Dermatology Venereol. . J. Santos-Juanes, CAIBER, Oficina de Investigación Biosanitaria, Universidad de Oviedo, Spain; 2012;26:785–8.

657. Armstrong AW, Harskamp CT, Armstrong EJ. The association between psoriasis and obesity: a systematic review and meta-analysis of observational studies. Nutr. Diabetes . 2012;2:e54.

658. Atzeni F, Sarzi-Puttini P. Twelve years experience with etanercept in the treatment of rheumatoid arthritis: How it has changed clinical practice. Expert Rev. Clin. Immunol. . P. Sarzi-Puttini, Rheumatology Unit, L. Sacco University Hospital, Milan, Italy; 2012;8:213–22. L3644497336

659. Augustin M, Langenbruch AK, Gutknecht M, Radtke MA, Blome C. Quality of Life Measures for Dermatology: Definition, Evaluation, and Interpretation. Curr. Dermatol. Rep. . M. Augustin, University Medical Center Hamburg-Eppendorf, D - 20246 Hamburg, Germany; 2012;1:148–59.

660. Bae Y-SC, Van Voorhees AS, Hsu S, Korman NJ, Lebwohl MG, Young M, et al. Review of treatment options for psoriasis in pregnant or lactating women: From the Medical Board of the National Psoriasis Foundation. J. Am. Acad. Dermatol. . A.S. Van Voorhees, University of Pennsylvania, Department of Dermatology, Philadelphia, PA 19104, United States; 2012;67:459–77.

661. Bailey EE, Ference EH, Alikhan A, Hession MT, Armstrong AW. Combination treatments for psoriasis: A systematic review and meta-analysis. Arch. Dermatol. . A.W. Armstrong, Department of Dermatology, University of California, Davis, School of Medicine, Sacramento, CA 95816, United States; 2012;148:511–22.

662. Balak DM, Hengstman GJ, Çakmak A, Thio HB. Cutaneous adverse events associated with disease-modifying treatment in multiple sclerosis: A systematic review. Mult. Scler. J. . H.B. Thio, Department of Dermatology, Erasmus Medical Center, 3015 CA Rotterdam, Netherlands; 2012;18:1705–17.

663. Banks T, Gada S. A comprehensive review of current treatments for granulomatous cheilitis. Br. J. Dermatol. . T. Banks, Walter Reed National Military Medical Center, Bethesda, MD 20889-5600, United States; 2012;166:934–7.

664. Basra MKA, Chowdhury MMU, Smith E V, Freemantle N, Piguet V. A Review of the Use of the Dermatology Life Quality Index as a Criterion in Clinical Guidelines and Health Technology Assessments in Psoriasis and Chronic Hand Eczema. Dermatol. Clin. . M.K.A. Basra, Department of Dermatology and Wound Healing, Cardiff University School of Medicine, Cardiff, CF14 4XN, Wales, United Kingdom; 2012;30:237–44.

665. Baughman RP, Meyer KC, Nathanson I, Angel L, Bhorade SM, Chan KM, et al. Monitoring of nonsteroidal immunosuppressive drugs in patients with lung disease and lung transplant recipients: American College of Chest Physicians evidence-based clinical practice guidelines. Chest . R.P. Baughman, University of Cincinnati, Holmes Bldg., Cincinnati, OH 45267-0565, United States; 2012;142:e1S – e111S.

666. Bergboer JGM, Umićević-Mirkov M, Fransen J, den Heijer M, Franke B, van Riel PLCM, et al. A replication study of the association between rheumatoid arthritis and deletion of the Late Cornified Envelope genes LCE3B and LCE3C. PLoS One . M. J. H. Coenen, Department of Human Genetics, Radboud University Nijmegen Medical Centre, Nijmegen, Netherlands; 2012;7.

667. Bessissow T, Renard M, Hoffman I, Vermeire S, Rutgeerts P, Van Assche G. Review article: Non-malignant haematological complications of anti-tumour necrosis factor alpha therapy. Aliment. Pharmacol. Ther. . T. Bessissow, Department of Gastroenterology, Royal Victoria Hospital, McGill University Health Center, Montreal, QC H3A 1A1, Canada; 2012;36:312–23.

668. Betts K, Yan Y, Sundaram M, Hengst N, Wolff M, Bensimon AG. Cost-effectiveness of biologic therapies for the treatment of moderate to severe psoriasis in Germany. Value Heal. . K. Betts, Analysis Group, Inc., Boston, United States; 2012;15:A570.

669. Biberoglu K. Prevention of endometriosis: Is it possible? J. Endometr. . K. Biberoglu, Department of Obstetrics and Gynecology, Gazi University Medical School, Ankara, Turkey; 2012;4:129–30.

670. Bigby M. The use of anti-interleukin-12/23 agents and major adverse cardiovascular events. Arch. Dermatol. . M. Bigby, Department of Dermatology, Harvard Medical School, Beth Israel Deaconess Medical Center, Boston, MA 02215, United States; 2012;148:753–4.

671. Bogaczewicz A, Sobów T, Bogaczewicz J, Sysa-Jȩdrzejowska A, Woźniacka A. Meta-analysis of the usage of biologics for depressive symptoms in patients with psoriasis. Dermatologia Klin. . A. Bogaczewicz, Zakład Psychologii Lekarskiej Katedry Nauk Humanistycznych UM, 91-425 Łódź, Poland; 2012;14:53–7.

672. Brezinski EA, Armstrong AW. Off-label biologic regimens in psoriasis: A systematic review of efficacy and safety of dose escalation, reduction, and interrupted biologic therapy. PLoS One . A. W. Armstrong, Department of Dermatology, University of California Davis, Sacramento, CA, United States; 2012;7.

673. Calkin C, Alda M. Beyond the guidelines for bipolar disorder: Practical issues in long-term treatment with lithium. Can. J. Psychiatry . M. Alda, Department of Psychiatry, Dalhousie University, Halifax, NS B3H 2E2, Canada; 2012;57:437–45.

674. Capon F, Barker JNWN. The quest for psoriasis susceptibility genes in the postgenome-wide association studies era: Charting the road ahead. Br. J. Dermatol. . J.N.W.N. Barker, Division of Genetics and Molecular Medicine, King’s College London, London SE1 9RT, United Kingdom; 2012;166:1173–5.

675. Castela E, Archier E, Devaux S, Gallini A, Aractingi S, Cribier B, et al. Topical corticosteroids in plaque psoriasis: A systematic review of efficacy and treatment modalities. J. Eur. Acad. Dermatology Venereol. . E. Castela, Dermatology Department, Nice University, L’Archet II Hospital, Nice, France; 2012;26:36–46.

676. Castela E, Archier E, Devaux S, Gallini A, Aractingi S, Cribier B, et al. Topical corticosteroids in plaque psoriasis: A systematic review of risk of adrenal axis suppression and skin atrophy. J. Eur. Acad. Dermatology Venereol. . E. Castela, Dermatology Department, Nice University, L’Archet II Hospital, Nice, France; 2012;26:47–51.

677. Chen Y-F, Chang JS. PTPN22 C1858T and the risk of psoriasis: A meta-analysis. Mol. Biol. Rep. . J.S. Chang, National Institute of Cancer Research, National Health Research Institutes, Tainan 70456, Taiwan; 2012;39:7861–70.

678. Cheng J, Kuai D, Zhang L, Yang X, Qiu B. Psoriasis increased the risk of diabetes: A meta-analysis. Arch. Dermatol. Res. . J. Cheng, Department of Dermatology, Beijing 302 Hospital, Beijing, China; 2012;304:119–25.

679. Cho JH. Immunochip-based analysis of a large ibd case-control cohort identifies 50 novel loci, refining definitions of disease pathways. Gastroenterology . J.H. Cho; 2012;142:S149–50.

680. Cifuentes RA, Restrepo-Montoya D, Anaya J-M. The autoimmune tautology: An in silico approach. Autoimmune Dis. . R.A. Cifuentes, Center for Autoimmune Diseases Research (CREA), School of Medicine and Health Sciences, Universidad Del Rosario, Bogotá, Colombia; 2012;1.

681. Croxtall JD. Spotlight on Ustekinumab in moderate to severe plaque psoriasis. Am. J. Clin. Dermatol. . J.D. Croxtall, Adis, North Shore 0754, Auckland, New Zealand; 2012;13:135–7.

682. Dańczak-Pazdrowska A. Place of methotrexate in the treatment of psoriasis in the era of biologic agents. Postep. Dermatologii i Alergol. . A. Dańczak-Pazdrowska, Department of Dermatology, Poznan University of Medical Sciences, 60-355 Poznan, Poland; 2012;29:182–8.

683. Dao KH, Herbert M, Habal N, Cush JJ. Nonserious Infections. Should There Be Cause for Serious Concerns? Rheum. Dis. Clin. North Am. . K.H. Dao, Baylor Research Institute, Dallas, TX 75229, United States; 2012;38:707–25.

684. Daudén E, Castañeda S, Suárez C, García-Campayo J, Blasco AJ, Aguilar MD, et al. Integrated approach to comorbidity in patients with psoriasis. Actas Dermosifiliogr. . A.J. Blasco, Técnicas Avanzadas de Investigación en Servicios de Salud, Madrid, Spain; 2012;103:1–64.

685. De Mozzi P, Johnston GA, Alexandroff AB. Psoriasis: An evidence-based update. Report of the 9th Evidenced Based Update Meeting, 12 May 2011, Loughborough, U.K. Br. J. Dermatol. . A.B. Alexandroff, Department of Dermatology, Leicester Royal Infirmary, Leicester LE1 5WW, United Kingdom; 2012;166:252–60.

686. Denadai R, Teixeira F V, Saad-Hossne R. The onset of psoriasis during the treatment of inflammatory bowel diseases with infliximab: Should biological therapy be suspended? Arq. Gastroenterol. . R. Denadai, Botucatu, SP, 17516-707, Brazil; 2012;49:172–6.

687. Deng S-Q, Brian M, Tony Z, Lu C-J, Xue CC. Herbal medicines in the topical management of psoriasis: A systematic review of clinical evidence. J. Dermatol. . S.-Q. Deng, WHO Collaboration Centre for Traditional Medicine, School of Health Sciences, RMIT University, Bundoora, Australia; 2012;39:226–7.

688. Devaux S, Castela A, Archier E, Gallini A, Joly P, Misery L, et al. Adherence to topical treatment in psoriasis: A systematic literature review. J. Eur. Acad. Dermatology Venereol. . S. Devaux, Dermatology Department, Paul Sabatier University, Toulouse, France; 2012;26:61–7.

689. Devaux S, Castela A, Archier E, Gallini A, Joly P, Misery L, et al. Topical vitamin D analogues alone or in association with topical steroids for psoriasis: A systematic review. J. Eur. Acad. Dermatology Venereol. . S. Devaux, Dermatology Department, Paul Sabatier University, Toulouse, France; 2012;26:52–60.

690. Dowlatshahi EA, Van Der Voort EA, Arends L, Nijsten T. Markers of systemic inflammation in psoriasis: A systematic review and meta-analysis. J. Invest. Dermatol. . E.A. Dowlatshahi, Dermatology, Erasmus Medical Center, Rotterdam, Netherlands; 2012;132:S71.

691. Ellinghaus E, Stuart PE, Ellinghaus D, Nair RP, Debrus S, Raelson J V, et al. Genome-wide meta-analysis of psoriatic arthritis identifies susceptibility locus at REL. J. Invest. Dermatol. . E. Ellinghaus, Institute of Clinical Molecular Biology, Christian-Albrechts-University Kiel, D-24105 Kiel, Germany; 2012;132:1133–40.

692. Famenini S, Wu JJ. The safety of ustekinumab in psoriasis. J. Drugs Dermatology . J.J. Wu, Kaiser Permanente Los Angeles Medical Center, Department of Dermatology, Los Angeles, CA 90027, United States; 2012;11:907–10.

693. Farrell J, Mills E, Sheppard O, Thorlund K. Anti-tumour necrosis factor (TNF) drugs for the treatment of psoriatic arthritis (PSA). Value Heal. . J. Farrell, MSD, Hoddesdon, Hertfordshire, United Kingdom; 2012;15:A440.

694. Ferrándiz C, García A, Blasco AJ, Lázaro P. Cost-efficacy of adalimumab, etanercept, infliximab and ustekinumab for moderate-to-severe plaque psoriasis. J. Eur. Acad. Dermatology Venereol. . A.J. Blasco, Advanced Techniques in Health Services Research (TAISS), Madrid, Spain; 2012;26:768–77.

695. Fischer A, Schmid B, Ellinghaus D, Nothnagel M, Gaede KI, Schürmann M, et al. A novel sarcoidosis risk locus for europeans on chromosome 11q13.1. Am. J. Respir. Crit. Care Med. . S. Schreiber, Christian-Albrechts University, Institute of Clinical Molecular Biology, 24105 Kiel, Germany; 2012;186:877–85.

696. Galimova E, Akhmetova V, Latipov B, Kingo K, Rätsep R, Traks T, et al. Analysis of genetic variants of class II cytokine and their receptor genes in psoriasis patients of two ethnic groups from the Volga-Ural region of Russia. J. Dermatol. Sci. . E. Galimova, Institute of Biochemistry and Genetics, Ufa Scientific Center of Russian Academy of Sciences, Ufa, Russian Federation; 2012;68:9–18.

697. Garcia-Doval I, Rustenbach S, Norman Dam T, Cohen AD, Baker C, Spuls PI, et al. Heterogeneity of patients treated with biologics in Psonet countries. Br. J. Dermatol. . I. Garcia-Doval, Research Unit, Fundacion Academia Española de Dermatologia y Venereologia, Madrid, Spain; 2012;167:e18–9.

698. Gensicke H, Leppert D, Yaldizli O, Lindberg RLP, Mehling M, Kappos L, et al. Monoclonal antibodies and recombinant immunoglobulins for the treatment of multiple sclerosis. CNS Drugs . L. Kappos, Neurology and Clinical Neuroimmunology, Departments of Medicine and Biomedicine, University Hospital Basel, CH-4031 Basel, Switzerland; 2012;26:11–37.

699. Graeber KE, Olsen NJ. Th17 cell cytokine secretion profile in host defense and autoimmunity. Inflamm. Res. . N.J. Olsen, Department of Medicine, M.S. Hershey Medical Center, Pennsylvania State University, Hershey, PA 17033, United States; 2012;61:87–96.

700. Granados D, Depont F, Chevreul K. Effect of the anti-tumor necrosis factor adalimumab on work productivity in patients with chronic immune-mediated inflammatory diseases: Literature review. Value Heal. . D. Granados, Abbott Laboratories, Rungis, France; 2012;15:A511.

701. Guerini FR, Cagliani R, Forni D, Agliardi C, Caputo D, Cassinotti A, et al. A functional variant in ERAP1 predisposes to multiple sclerosis. PLoS One . F. R. Guerini, Don C. Gnocchi Foundation ONLUS, Milano, Italy; 2012;7.

702. Hayter SM, Cook MC. Updated assessment of the prevalence, spectrum and case definition of autoimmune disease. Autoimmun. Rev. . M.C. Cook, Department of Immunology, Canberra Hospital, Woden ACT, 2606, Australia; 2012;11:754–65.

703. Heo J, Sepah YJ, Yohannan J, Renner M, Akhtar A, Gregory A, et al. The role of biologic agents in the management of non-infectious uveitis. Expert Opin. Biol. Ther. . Q.D. Nguyen, Johns Hopkins University School of Medicine, Wilmer Eye Institute, Johns Hopkins Hospital, Baltimore, MD 21287, United States; 2012;12:995–1008.

704. Holick MF. Evidence-based D-bate on health benefits of vitamin D revisited. Dermatoendocrinol. . M.F. Holick, Department of Medicine, Vitamin D, Skin and Bone Research Laboratory, Boston University Medical Center, Boston, MA, United States; 2012;4:183–90.

705. Ingham M, Ellis L, Bolge S. Number needed to treat (NNT), cost per responder (CPR) and budget impact (BI) of tnf inhibitors (TNFI) in the treatment of psoriatic arthritis (PSA). Value Heal. . M. Ingham, Janssen Scientific Affairs, LLC, Horsham, United States; 2012;15:A35.

706. Jemec GBE, Ibler KS. Treatment of nail psoriasis with TNF-α or IL 12/23 inhibitors. J. Drugs Dermatology . G.B.E. Jemec, Department of Dermatology, Roskilde Hospital, University of Copenhagen, Denmark; 2012;11:939–42.

707. Jordan CT, Cao L, Roberson EDO, Duan S, Helms CA, Nair RP, et al. Rare and common variants in CARD14, encoding an epidermal regulator of NF-kappaB, in psoriasis. Am. J. Hum. Genet. . A.M. Bowcock, Department of Genetics, School of Medicine, Washington University, St. Louis, MO 63110, United States; 2012;90:796–808.

708. Jostins L, Ripke S, Weersma RK, Duerr RH, McGovern DP, Hui KY, et al. Host-microbe interactions have shaped the genetic architecture of inflammatory bowel disease. Nature . J.H. Cho, Department of Genetics, Yale School of Medicine, New Haven, CT 06520, United States; 2012;491:119–24.

709. Kamangar F, Neuhaus IM, Koo JYM. An evidence-based review of skin cancer rates on biologic therapies. J. Dermatolog. Treat. . F. Kamangar, University of California, Davis School of Medicine, San Francisco Psoriasis Skin and Treatment Center, San Francisco, CA 94118, United States; 2012;23:305–15.

710. Katz U, Shoenfeld Y, Zakin V, Sherer Y, Sukenik S. Scientific Evidence of the Therapeutic Effects of Dead Sea Treatments: A Systematic Review. Semin. Arthritis Rheum. . U. Katz, Maccabi Healthcare Services, Tel Aviv 64732, Israel; 2012;42:186–200.

711. Kaur A, Kumar S. Plants and plant products with potential antipsoriatic activity - A review. Pharm. Biol. . S. Kumar, Department of Pharmaceutical Sciences and Drug Research, Punjabi University, Patiala-147 002, Punjab, India; 2012;50:1573–91.

712. Kim IH, West CE, Kwatra SG, Feldman SR, O’Neill JL. Comparative efficacy of biologics in psoriasis: A review. Am. J. Clin. Dermatol. . C.E. West, Center for Dermatology Research, Department of Dermatology, Wake Forest School of Medicine, Winston-Salem, NC 27157-1071, United States; 2012;13:365–74.

713. Kurizky PS, da Mota LMH. Sexual dysfunction in patients with psoriasis and psoriatic arthritis - A systematic review. Rev. Bras. Reumatol. . L.M.H. da Mota, Centro Médico de Brasília, Asa Sul. CEP: 70390-904. Brasília, DF, Brazil; 2012;52:943–8.

714. Kwatra SG, Dabade TS, Gustafson CJ, Feldman SR. JAK inhibitors in psoriasis: A promising new treatment modality. J. Drugs Dermatology . C.J. Gustafson, Center for Dermatology Research, Department of Dermatology, Wake Forest University School of Medicine, Winston-Salem, NC, United States; 2012;11:913–8.

715. Kydd A, March L. Smoking and ankylosing spondylitis: A systematic review. J. Rheumatol. . A. Kydd, University of British Columbia, Roberts Creek, Canada; 2012;39:1710–1.

716. Lakatos PL. Anti-TNFs and severe infections in autoimmune diseases: The other side of the coin. Inflamm. Bowel Dis. . P.L. Lakatos, 1st Department of Medicine, Semmelweis University, Budapest, Hungary; 2012;18:2414–6.

717. Lamel SA, Myer KA, Younes N, Zhou JA, Maibach H, Maibach HI. Placebo response in relation to clinical trial design: A systematic review and meta-analysis of randomized controlled trials for determining biologic efficacy in psoriasis treatment. Arch. Dermatol. Res. 2012;304:707–17.

718. Lavda AC, Webb TL, Thompson AR. A meta-analysis of the effectiveness of psychological interventions for adults with skin conditions. Br. J. Dermatol. . A.R. Thompson, Department of Psychology, University of Sheffield, Sheffield, United Kingdom; 2012;167:970–9.

719. Lee YH, Choi SJ, Ji JD, Song GG. Associations between interleukin-10 polymorphisms and susceptibility to psoriasis: A meta-analysis. Inflamm. Res. . Y.H. Lee, Department of Internal Medicine, Korea University Anam Hospital, Korea University College of Medicine, Seongbuk-gu, Seoul 136-705, South Korea; 2012;61:657–63.

720. Lee YH, Choi SJ, Ji JD, Song GG. Vitamin D receptor ApaI, TaqI, BsmI, and FokI polymorphisms and psoriasis susceptibility: A meta-analysis. Mol. Biol. Rep. . Y.H. Lee, Department of Internal Medicine, Korea University Anam Hospital, Korea University College of Medicine, Seongbuk-gu, Seoul 136-705, South Korea; 2012;39:6471–8.

721. Li N, Li Y-Q, Li H-Y, Guo W, Bai Y-P. Efficacy of externally applied chinese herbal drugs in treating psoriasis: A systematic review. Chin. J. Integr. Med. . Y.-P. Bai, Department of Dermatology, China-Japan Friendship Hospital, Beijng 100029, China; 2012;18:222–9.

722. Li X, Ampleford E, Howard T, Torgerson D, Li H, Moore W, et al. Genome-wide association studies of asthma indicate opposite immunopathogenesis direction from autoimmune diseases. World Allergy Organ. J. . X. Li, Center for Genomics and Personalized Medicine Research, Wake Forest University, School of Medicine, Winston Salem, United States; 2012;5:S55–6.

723. Liang Y-L, Wu H, Shen X, Li P-Q, Yang X-Q, Liang L, et al. Association of STAT4 rs7574865 polymorphism with autoimmune diseases: A meta-analysis. Mol. Biol. Rep. . X.-D. Xie, Key Laboratory of Preclinical Study for New Drugs of Gansu Province, School of Basic Medical Sciences, Lanzhou University, Lanzhou City, Gansu Province 730000, China; 2012;39:8873–82.

724. Lin VW, Ringold S, Devine EB. Comparison of Ustekinumab With Other Biological Agents for the Treatment of Moderate to Severe Plaque Psoriasis: A Bayesian Network Meta-analysis. Arch. Dermatol. . 2012;148:1403–10.

725. Liu Y, Wu EQ, Bensimon AG, Fan C-PS, Bao Y, Ganguli A, et al. Cost per responder associated with biologic therapies for crohn’s disease, psoriasis, and rheumatoid arthritis. Adv. Ther. . Y. Liu, Division of Pharmacy Practice and Administration, University of Missouri, Kansas City School of Pharmacy, Kansas City, MO 64108, United States; 2012;29:620–34.

726. Lucka TC, Pathirana D, Sammain A. Erratum: Efficacy of systemic therapies for moderate-to-severe psoriasis: A systematic review and meta-analysis of long-term treatment (Journal of the European Academy of Dermatology and Venereology (2012) DOI: 10.1111/j.1468-3083. 2012.04492.x). J. Eur. Acad. Dermatology Venereol. . T.C. Lucka; 2012;26:930.

727. Lucka TC, Pathirana D, Sammain A, Bachmann F, Rosumeck S, Erdmann R, et al. Efficacy of systemic therapies for moderate-to-severe psoriasis: A systematic review and meta-analysis of long-term treatment. J. Eur. Acad. Dermatology Venereol. . A. Nast, Division of Evidence Based Medicine (DEBM), Charité- Universitätsmedizin, Berlin, Germany; 2012;26:1331–44.

728. Maes M, Berk M, Goehler L, Song C, Anderson G, Gałecki P, et al. Depression and sickness behavior are Janus-faced responses to shared inflammatory pathways. BMC Med. . M. Maes, Maes Clinics at TRIA, Piyavate Hospital, Bangkok 10310, Thailand; 2012;10.

729. Malhotra A, Shafiq N, Rajagopalan S, Dogra S, Malhotra S. Thiazolidinediones for plaque psoriasis: a systematic review and meta-analysis. Evid. Based. Med. . 2012;17:171–6.

730. Marcellusi A, Gitto L, Giannantoni P, Attinà G, Sundaram M, Mennini FS. Economic evaluation of biologic treatments for moderate to severe psoriasis in Italy. Value Heal. . A. Marcellusi, University of Rome, Rome, Italy; 2012;15:A570.

731. Marino A, Pagnini I, Savelli S, Moretti D, Simonini G, Cimaz R. Elbow monoarthritis: An atypical onset of juvenile idiopathic arthritis. Reumatismo . A. Marino; 2012;64:175–9.

732. May BH, Zhang AL, Zhou W, Lu C-J, Deng S, Xue CCL. Oral herbal medicines for psoriasis: A review of clinical studies. Chin. J. Integr. Med. . C.C.L. Xue, Health Innovations Research Institute, School of Health Sciences, RMIT University, Bundoora, VIC 3083, Australia; 2012;18:172–8.

733. Micha R, Imamura F, Wyler Von Ballmoos M, Solomon DH, Hernán MA, Ridker PM, et al. Authors’ reply. Am. J. Cardiol. . R. Micha, Boston, MA, United States; 2012;109:1823.

734. Mudigonda T, Dabade TS, Feldman SR. A review of targeted ultraviolet B phototherapy for psoriasis. J. Am. Acad. Dermatol. . T.S. Dabade, Department of Dermatology, Wake Forest University, School of Medicine, Winston-Salem, NC 27157-1071, United States; 2012;66:664–72.

735. Mudigonda T, Dabade TS, Feldman SR. A Review of protocols for 308 nm excimer laser phototherapy in psoriasis. J. Drugs Dermatology . S.R. Feldman, Dermatology, Pathology and Public Health Sciences, Wake Forest Baptist Medical Center, Winston-Salem, NC 27157-1071, United States; 2012;11:92–7.

736. Nast A, Boehncke W-H, Mrowietz U, Ockenfels H-M, Philipp S, Reich K, et al. S3 - Guidelines on the treatment of psoriasis vulgaris (English version). Update. JDDG - J. Ger. Soc. Dermatology . A. Nast, Division of Evidence Based Medicine (DEBM), Klinik für Dermatologie, Venerologie und Allergologie, Charité- Universitätsmedizin, 10117 Berlin, Germany; 2012;10:S1–95.

737. Nijsten T, Stern RS. How epidemiology has contributed to a better understanding of skin disease. J. Invest. Dermatol. . T. Nijsten, Department of Dermatology, Erasmus University Medical Center, 3000 CA Rotterdam, Netherlands; 2012;132:994–1002.

738. Nordgaard-Lassen I, Dahlerup JF, Belard E, Gerstoft J, Kjeldsen J, Kragballe K, et al. Guidelines for screening, prophylaxis and critical information prior to initiating anti-TNF-alpha treatment. Dan. Med. J. . I. Nordgaard-Lassen, Gastrounit, Medical Section, Hvidovre Hospital, 2650 Hvidovre, Denmark; 2012;59.

739. Olyaeemanesh A, Doaee S, Nejati M, Mobinizadeh M, Aboee P, Beyhaghi H. Effectiveness and safety of Etanercept in treatment of arthritis. HealthMED . M. Nejati, HTA office, Deputy of Curative Affairs, Ministry of Health and Medical Education (MOHME), Tehran, Taiwan; 2012;6:2709–16.

740. Page EK, Dar WA, Knechtle SJ. Biologics in organ transplantation. Transpl. Int. . S.J. Knechtle, 5105-WMB, Atlanta, GA 30322, United States; 2012;25:707–19. L51916678741. Paul C, Gallini A, Archier E, Castela E, Devaux S, Aractingi S, et al. Evidence-based recommendations on topical treatment and phototherapy of psoriasis: Systematic review and expert opinion of a panel of dermatologists. J. Eur. Acad. Dermatology Venereol. . C. Paul, Dermatology Department, Paul Sabatier University, Toulouse, France; 2012;26:1–10.

742. Paul C, Ortonne J-P. Topical treatment and phototherapy in psoriasis: Systematic review and expert opinion of a panel of dermatologists. J. Eur. Acad. Dermatology Venereol. . C. Paul, Department of Dermatology, Paul Sabatier University, Larrey Hospital, Toulouse, France; 2012;26:iii – iv.

743. Pereira FRA, Basra MKA, Finlay AY, Salek MS. The role of the EQ-5D in the economic evaluation of dermatological conditions and therapies. Dermatology . F.R.A. Pereira, Centre for Socioeconomic Research, Redwood Building, Cardiff University, Cardiff CF10 3XF, United Kingdom; 2012;225:45–53.

744. Perk J, De Backer G, Gohlke H, Graham I, Reiner Z, Verschuren WMM, et al. European Guidelines on cardiovascular disease prevention in clinical practice (version 2012): The Fifth Joint Task Force of the European Society of Cardiology and Other Societies on Cardiovascular Disease Prevention in Clinical Practice (constituted by representatives of nine societies and by invited experts). Eur. J. Prev. Cardiol. . J. Perk, School of Health and Caring Sciences, Linnaeus University, SE-391 82 Kalmar, Sweden; 2012;19:585–667.

745. Pham T, Bachelez H, Berthelot J-M, Blacher J, Claudepierre P, Constantin A, et al. Abatacept therapy and safety management. Jt. Bone Spine . T. Pham, Service de Rhumatologie, CHU Sainte-Marguerite, Marseille, France; 2012;79:3–84.

746. Posso-De Los Rios CJ, Pope E, Lara-Corrales I. Systemic interventions in pediatric pustular psoriasis: Systematic review. Pediatr. Dermatol. . C.J. Posso-De Los Rios, Hospital for Sick Children, United States; 2012;29:690.

747. Reich K, Burden AD, Eaton JN, Hawkins NS. Efficacy of biologics in the treatment of moderate to severe psoriasis: A network meta-analysis of randomized controlled trials. Br. J. Dermatol. . J.N. Eaton, Health Economics, Oxford Outcomes, Seacourt Tower, Oxford, OX2 0JJ, United Kingdom; 2012;166:179–88.

748. Remmers EF, Kirino Y, Bertsias G, Ishigatsubo Y, Kim Y, Ombrello MJ, et al. Genome-wide analysis reveals a recessive association of ERAP1 variants with behc¸et’s disease and epistasis between ERAP1 and HLA-B∗51. Arthritis Rheum. . E.F. Remmers, National Human Genome Research Institute, National Institutes of Health, Bethesda, United States; 2012;64:S428.

749. Robinson A, Kardos M, Kimball AB. Physician Global Assessment (PGA) and Psoriasis Area and Severity Index (PASI): Why do both? A systematic analysis of randomized controlled trials of biologic agents for moderate to severe plaque psoriasis. J. Am. Acad. Dermatol. . Elsevier Inc; 2012;66:369–75.

750. Sawchuk M, Spano F, Loo WJ, Guenther L. The coexistence of psoriasis and vitiligo: A review. J. Cutan. Med. Surg. . M. Sawchuk, Division of Dermatology, Department of Medicine, Schulich School of Medicine, University of Western Ontario, Canada; St. Joseph’s Health Care, Canada; Guenther Dermatology Research Centre, London, ON, Canada; 2012;16:300–5.

751. Selvarajah V, Montano-Loza AJ, Czaja AJ. Systematic review: Managing suboptimal treatment responses in autoimmune hepatitis with conventional and nonstandard drugs. Aliment. Pharmacol. Ther. . A.J. Montano-Loza, Zeidler Ledcor Centre, University of Alberta, Edmonton, AB T6G 2X8, Canada; 2012;36:691–707.

752. Silva F, Cisternas M, Specks U. TNF-α blocker therapy and solid malignancy risk in ANCA-associated vasculitis. Curr. Rheumatol. Rep. . F. Silva, Departamento de Inmunología Clínica y Reumatología, Escuela de Medicina, Facultad de Medicina, Pontificia Universidad Católica de Chile, Santiago, Chile; 2012;14:501–8.

753. Singh S, Mann BK. Clinical utility of clocortolone pivalate for the treatment of corticosteroid-responsive skin disorders: A systematic review. Clin. Cosmet. Investig. Dermatol. . S. Singh, Lanka, Varanasi-221005, India; 2012;5:61–8.

754. Sorin D, Pavlovsky L, David M. Psoriasis in Pregnancy. Curr. Dermatol. Rep. . M. David, Department of Dermatology, Rabin Medical Center, Petach Tiqva, 49100, Israel; 2012;1:209–13.

755. Spuls PHI. Critical appraisal of treatment guidelines for psoriasis. Br. J. Dermatol. . P.H.I. Spuls, Department of Dermatology, Academic Medical Centre, University of Amsterdam, Amsterdam, Netherlands; 2012;167:e5–6.

756. Stuart PE, Hüffmeier U, Nair RP, Palla R, Tejasvi T, Schalkwijk J, et al. Association of β-defensin copy number and psoriasis in three cohorts of European origin. J. Invest. Dermatol. . J.A.L. Armour, School of Biology, University of Nottingham, Queen’s Medical Centre, Nottingham NG7 2UH, United Kingdom; 2012;132:2407–13.

757. Tan X, Feldman SR, Chang J, Balkrishnan R. Topical drug delivery systems in dermatology: A review of patient adherence issues. Expert Opin. Drug Deliv. . X. Tan, University of Michigan, College of Pharmacy, Department of Clinical, Ann Arbor, MI, United States; 2012;9:1263–71.

758. Tang T, Bieber T, Williams H. Does “autoreactivity” play a role in atopic dermatitis? J. Invest. Dermatol. . T. Tang, Centre of Evidence-Based Dermatology, University of Nottingham, Nottingham, United Kingdom; 2012;132:S34.

759. Tejasvi T, Stuart PE, Chandran V, Voorhees JJ, Gladman DD, Rahman P, et al. TNFAIP3 gene polymorphisms are associated with response to TNF blockade in psoriasis. J. Invest. Dermatol. . R.P. Nair, Department of Dermatology, University of Michigan Medical School, Ann Arbor, MI 48109-5675, United States; 2012;132:593–600.

760. Thorlund K, Druyts E, Aviña-Zubieta JA, Mills EJ. Anti-tumor necrosis factor (TNF) drugs for the treatment of psoriatic arthritis: An indirect comparison meta-analysis. Biol. Targets Ther. . E. J. Mills, Faculty of Health Sciences, University of Ottawa, Ottawa K1N 6X1, Canada; 2012;6:417–27.

761. Thyssen JP, Johansen JD, Carlsen BC, Linneberg A, Meldgaard M, Szecsi PB, et al. The filaggrin null genotypes R501X and 2282del4 seem not to be associated with psoriasis: Results from general population study and meta-analysis. J. Eur. Acad. Dermatology Venereol. . J.P. Thyssen, National Allergy Research Centre, Department of Dermato-Allergology, Copenhagen University Hospital Gentofte, Denmark; 2012;26:782–4.

762. Tian S, Krueger JG, Jabbari A, Li K, Brodmerkel C, Lowes M, et al. Meta-analysis reveals “global” psoriasis transcriptome with links to cardiovascular, metabolic and other comorbidities. J. Invest. Dermatol. . S. Tian, Laboratory for Investigative Dermatology, Rockefeller University, New York, United States; 2012;132:S8.

763. Tian S, Krueger JG, Li K, Jabbari A, Brodmerkel C, Lowes MA, et al. Meta-Analysis Derived (MAD) Transcriptome of Psoriasis Defines the “Core” Pathogenesis of Disease. PLoS One . M. Suárez-Fariñas, Center for Clinical and Translational Science, The Rockefeller University, New York, NY, United States; 2012;7.

764. Tillett W, de-Vries C, McHugh NJ. Work disability in psoriatic arthritis: A systematic review. Rheumatology . W. Tillett, Department of Rheumatology, Royal National Hospital for Rheumatic Diseases, Bath BA11RL, United Kingdom; 2012;51:275–83.

765. Trembath RC, Abecasis G, Elder JT, Of Psoriasis O, Psoriasis Consortium T, Consortium A. The identification of fifteen novel psoriasis susceptibility loci highlights the skin’s role in innate immune defense. J. Invest. Dermatol. . R.C. Trembath, Queen Mary University of London, Barts and the London School of Medicine and Dentistry, London, United Kingdom; 2012;132:S95.

766. Tsoi LC, Spain SL, Knight J, Ellinghaus E, Stuart PE, Capon F, et al. Identification of 15 new psoriasis susceptibility loci highlights the role of innate immunity. Nat. Genet. . G.R. Abecasis, Wellcome Trust Sanger Institute, Hinxton, Cambridge, United Kingdom; 2012;44:1341–8.

767. Tzellos T, Kyrgidis A, Trigoni A, Zouboulis CC. Association of ustekinumab and briakinumab with major adverse cardiovascular events: An appraisal of meta-analyses and industry sponsored pooled analyses to date. Dermatoendocrinol. . C.C. Zouboulis, Division of Evidence Based Dermatology, Departments of Dermatology, Venereology, Allergology and Immunology, Dessau Medical Center, Dessau, Germany; 2012;4.

768. Umar N, Yamamoto S, Loerbroks A, Terris D. Elicitation and use of patients’ preferences in the treatment of psoriasis: A systematic review. Acta Derm. Venereol. . N. Umar, Mannheim Institute of Public Health, Social and Preventive Medicine, Universitätsmedizin Mannheim, Medical Faculty Mannheim, Heidelberg University, DE-68167 Mannheim, Germany; 2012;92:341–6.

769. Valencia-Mendoza A, Hernández-Garduño A, Puig A. Cost-effectiveness of ustekinumab in the management of moderate-to-severe plaque psoriasis in Mexico. Value Heal. . A. Valencia-Mendoza, Janssen de Mexico, Mexico, D.F., Mexico; 2012;15:A513.

770. Vena GA, Cassano N, Piaserico S, Conti A, Girolomoni G. Efficacy of etanercept for the treatment of psoriasis: An overview of the Italian clinical experience from the real-life setting and independent studies. Immunopharmacol. Immunotoxicol. . G.A. Vena, Department of Biomedical Sciences and Human Oncology, Unit of Dermatology and Venereology, University of Bari, Policlinico, 70124 Bari, Italy; 2012;34:901–6.

771. Voulgari P V, Kaltsonoudis E, Papagoras C, Drosos AA. Adalimumab in the treatment of rheumatoid arthritis. Expert Opin. Biol. Ther. . A.A. Drosos, University of Ioannina, Medical School, Rheumatology Clinic, Department of Internal Medicine, Ioannina, Greece; 2012;12:1679–86.

772. Wain L V, Artigas MS, Tobin MD. What can genetics tell us about the cause of fixed airflow obstruction? Clin. Exp. Allergy . M.D. Tobin, Departments of Health Sciences and Genetics, University of Leicester, Leicester LE1 7RH, United Kingdom; 2012;42:1176–82.

773. Wang X, Bansback N, Anis A, Joshi AD, Rao S, Wolff M, et al. Economic evaluation model of biologic therapies for moderate to severe psoriatic arthritis in Germany. Value Heal. . X. Wang, University of British Columbia, Vancouver, Canada; 2012;15:A446.

774. Williams HC, Dellavalle RP. The growth of clinical trials and systematic reviews in informing dermatological patient care. J. Invest. Dermatol. . H.C. Williams, Centre of Evidence-Based Dermatology, Nottingham University Hospitals NHS Trust, University Hospital, Nottingham NG7 2UH, United Kingdom; 2012;132:1008–17.

775. Wong PCH, Leung Y-Y, Li EK, Tam L-S. Measuring disease activity in psoriatic arthritis. Int. J. Rheumatol. . L.-S. Tam, Department of Medicine and Therapeutics, Prince of Wales Hospital, Chinese University of Hong Kong, Shatin, NT, Hong Kong; 2012;2012.

776. Wood A. Genome wide association studies (GWAS) for height variations: Where are we now? Horm. Res. Paediatr. . A. Wood, Genetics of Complex Traits, Peninsula College of Medicine and Dentistry, Exeter, United Kingdom; 2012;78:8.

777. Woods MS, Zimovetz E, Beard S, Balp MM. Systematic review of economic evaluations, utility estimates, resource utilisation, and costs in chronic idiopathic urticaria. Value Heal. . M.S. Woods, RTI Health Solutions, Manchester, United Kingdom; 2012;15:A562.

778. Wu Y, Chen J, Li Y-H, Ma G-H, Chen JZS, Gao X-H, et al. Treatment of psoriasis with interleukin-12/23 monoclonal antibody: A systematic review. Eur. J. Dermatology . H. D. Chen, Department of Dermatology, No.1 Hospital of China Medical University, Shenyang 110001, China; 2012;22:72–82.

779. Xu T, Zhang Y-H. Association of psoriasis with stroke and myocardial infarction: Meta-analysis of cohort studies. Br. J. Dermatol. . T. Xu, Department of Epidemiology, School of Public Health, Medical College of Soochow University, Suzhou, Jiangsu Province 215123, China; 2012;167:1345–50.

780. Yang H, Craig D, Epstein D, Bojke L, Light K, Bruce IN, et al. Golimumab for the treatment of psoriatic arthritis: A NICE single technology appraisal. Pharmacoeconomics . H. Yang, Centre for Reviews and Dissemination, University of York, York, YO10 5DD, United Kingdom; 2012;30:257–70.

781. Zheng J, Ibrahim S, Petersen F, Yu X. Meta-analysis reveals an association of PTPN22 C1858T with autoimmune diseases, which depends on the localization of the affected tissue. Genes Immun. . X. Yu, Laboratory of Autoimmunity, Medical College of Xiamen University, Xiamen University, Xiamen 361005, China; 2012;13:641–52.

782. Zhu K, Yin X, Tang X, Zhang F, Yang S, Zhang X. Meta-Analysis of NOD2/CARD15 polymorphisms with psoriasis and psoriatic arthritis. Rheumatol. Int. . S. Yang, Institute of Dermatology, Department of Dermatology, Anhui Medical University, 230032 Hefei, Anhui, China; 2012;32:1893–900.

783. Zhu K-J, Zhu C-Y, Fan Y-M. Alcohol consumption and psoriatic risk: A meta-analysis of case-control studies. J. Dermatol. . K.-J. Zhu, Department of Dermatology, Affiliated Hospital of Guangdong Medical College, Xiashan District, Zhanjiang, Guangdong 524001, China; 2012;39:770–3.

784. Zhu K-J, Zhu C-Y, Shi G, Fan Y-M. Association of IL23R polymorphisms with psoriasis and psoriatic arthritis: A meta-analysis. Inflamm. Res. . K.-J. Zhu, Department of Dermatology, Affiliated Hospital of Guangdong, Medical College, Zhanjiang, Guangdong 524001, China; 2012;61:1149–54.

785. Zöller B, Li X, Sundquist J, Sundquist K. Risk of pulmonary embolism in patients with autoimmune disorders: A nationwide follow-up study from Sweden. Lancet . B. Zöller, Center for Primary Health Care Research, Lund University/Region Skåne, Malmö University Hospital, Malmö S-205 02, Sweden; 2012;379:244–9.

**2011**

786. Abdullah L, Abbas O. Common nail changes and disorders in older people: Diagnosis and management. Can. Fam. Physician . O. Abbas, Department of Dermatology, American University of Beirut, Medical Centre, Riad El Solh/Beirut 1107 2020, Lebanon; 2011;57:173–81.

787. Actis GC, Rosina F, MacKay IR. Inflammatory bowel disease: Beyond the boundaries of the bowel. Expert Rev. Gastroenterol. Hepatol. . G. C. Actis, Department of Gastro-Hepatology, Ospedale Gradenigo, Torino 10153, Italy; 2011;5:401–10.

788. Afzali A, Wheat C, Lee S. The association of psoriasis with anti-tumor necrosis factor (anti-TNF)AQ1 therapy in inflammatory bowel disease: A single-center case series. Inflamm. Bowel Dis. . A. Afzali, University of Washington, Seattle, United States; 2011;17:S17.

789. Alandete JC. Effect of treatment switch on the cost-effectiveness of biologics in psoriasis in peru and colombia. Value Heal. . J.C. Alandete, Janssen-Cilag, Bogota, Colombia; 2011;14:A58.

790. Albrecht L, Bourcier M, Ashkenas J, Papp K. Topical psoriasis therapy in the age of biologics: Evidence-based treatment recommendations. J. Cutan. Med. Surg. . L. Albrecht, Surrey, BC V3R 6A7, Canada; 2011;15:309–21.

791. Amitay-Laish I, Stemmer SM, Lacouture ME. Adverse cutaneous reactions secondary to tyrosine kinase inhibitors including imatinib mesylate, nilotinib, and dasatinib. Dermatol. Ther. . I. Amitay-Laish, Department of Dermatology, Rabin Medical Center, Beilinson Hospital, Petah Tikva 4910, Israel; 2011;24:386–95.

792. Augustin M, Holland B, Dartsch D, Langenbruch A, Radtke MA. Adherence in the treatment of psoriasis: A systematic review. Dermatology . M.A. Radtke, Competenzzentrum Versorgungsforschung in der Dermatologie, Institut für Versorgungsforschung in der Dermatologie und bei Pflegeberufen, Universitätsklinikum Hamburg-Eppendorf, DE-20246 Hamburg, Germany; 2011;222:363–74.

793. Baker EL, Coleman CI, Reinhart KM, Phung OJ, Ashaye A, Kugelman L, et al. Safety of biologic treatments for moderate to severe plaque psoriasis: A systematic review, basic meta-analysis, and Bayesian mixed treatment comparison. Pharmacotherapy . E.L. Baker, Health Outcomes, Policy and Economics (HOPE) Collaborative Group, Hartford, United States; 2011;31:327e – 328e.

794. Bottomley JM, Taylor RS, Ryttov J. The effectiveness of two-compound formulation calcipotriol and betamethasone dipropionate gel in the treatment of moderately severe scalp psoriasis: A systematic review of direct and indirect evidence. Curr. Med. Res. Opin. . J. M. Bottomley, Amygdala Ltd., Letchworth Garden City, Hertfordshire, SG6 2AA, United Kingdom; 2011;27:251–68.

795. Brunasso AMG, Puntoni M, Gulia A, Massone C. Safety of anti-tumour necrosis factor agents in patients with chronic hepatitis C infection: A systematic review. Rheumatology . A.M.G. Brunasso, Department of Environmental Dermatology and Venereology, Medical University of Graz, A-8036 Graz, Austria; 2011;50:1700–11.

796. Byekova YA, Hughey LC, Elewski BE. Systemic drugs in patients with skin diseases. G. Ital. di Dermatologia e Venereol. . Y.A. Byekova, EFH 414, Birmingham, AL 35294-0009, United States; 2011;146:397–424.

797. Capon F. Genetics of psoriasis. Medizinische Genet. . F. Capon, Division of Genetics and Molecular Medicine, King’s College, London, United Kingdom; 2011;23:67.

798. Chiu H-Y, Tsai T-F. Topical use of systemic drugs in dermatology: A comprehensive review. J. Am. Acad. Dermatol. . T.-F. Tsai, Department of Dermatology, National Taiwan University Hospital, National Taiwan University College of Medicine, Taipei 100, Taiwan; 2011;65:1048.e1–1048.e22.

799. Cotsapas C, Voight BF, Rossin E, Lage K, Neale BM, Wallace C, et al. Pervasive sharing of genetic effects in autoimmune disease. PLoS Genet. . M. J. Daly, Center For Human Genetic Research, Massachusetts General Hospital, Boston, MA, United States; 2011;7.

800. Cottone M, Criscuoli V. Infliximab to treat Crohn’s disease: An update. Clin. Exp. Gastroenterol. . V. Criscuoli, 90146 Palermo, Italy; 2011;4:227–38.

801. Cullen G, Kroshinsky D, Cheifetz AS, Korzenik JR. Psoriasis associated with anti-tumour necrosis factor therapy in inflammatory bowel disease: A new series and a review of 120 cases from the literature. Aliment. Pharmacol. Ther. . G. Cullen, Division of Gastroenterology, Beth Israel Deaconess Medical Center, Boston, MA 02215, United States; 2011;34:1318–27.

802. David C V, Nguyen H, Goldenberg G. Imiquimod: A review of off-label clinical applications. J. Drugs Dermatology . G. Goldenberg, New York, NY 10029, United States; 2011;10:1300–6.

803. Docampo E, Giardina E, Riveira-Muñoz E, De Cid R, Escaramís G, Perricone C, et al. Deletion of LCE3C and LCE3B is a susceptibility factor for psoriatic arthritis: A study in Spanish and Italian populations and meta-analysis. Arthritis Rheum. . R. Rabionet, Genes and Disease Program, Center for Genomic Regulation, PRBB Building, Barcelona 08003, Spain; 2011;63:1860–5.

804. Dodds MG, Dong H, Tsuji W, Martin D, Gibbs JP, Salinger DH, et al. Clinical trial simulation to inform phase 2: Comparison of distributed versus concentrated phase 1 study designs. Clin. Pharmacol. Ther. . M.G. Dodds, Amgen, Inc., Seattle, United States; 2011;89:S84–5.

805. Domm S, Mrowietz U. Combination therapy in the treatment of psoriasis. JDDG - J. Ger. Soc. Dermatology . S. Domm, Psoriasis-Zentrum, Abt. Dermatologie, Venerologie und Allergologie, Campus Kiel, D-24105 Kiel, Germany; 2011;9:94–8.

806. Dommasch ED, Abuabara K, Shin DB, Nguyen J, Troxel AB, Gelfand JM. The risk of infection and malignancy with tumor necrosis factor antagonists in adults with psoriatic disease: A systematic review and meta-analysis of randomized controlled trials. J. Am. Acad. Dermatol. . J. M. Gelfand, Department of Biostatistics and Epidemiology, University of Pennsylvania, 1471 Penn Tower, Philadelphia, PA 19104, United States; 2011;64:1035–50.

807. Dommasch E, Troxel A, Shin D, Gelfand J, Abuabara K. The safety of tumor necrosis factor antagonists in patients with psoriatic disease: A systematic review and metaanalysis of randomized controlled trials. J. Am. Acad. Dermatol. . E. Dommasch, University of Pennsylvania, Department of Dermatology, Philadelphia, United States; 2011;64:AB8.

808. El Maghraoui A. Extra-articular manifestations of ankylosing spondylitis: Prevalence, characteristics and therapeutic implications. Eur. J. Intern. Med. . A. El Maghraoui, Rheumatology Department, Military Hospital Mohammed v, Rabat, Morocco; 2011;22:554–60.

809. Esparza Gordillo J, Schaarschmidt H, Weidinger S, Moffat M, Liang L, Lee-Kirsch M, et al. Overlap among inflammatory disorders identifies new genetic risk factors for atopic dermatitis. Allergy Eur. J. Allergy Clin. Immunol. . J. Esparza Gordillo, CharitéUniversity Medicine, Berlin, Germany; 2011;66:681.

810. Fan T, Bennett H, Smith N, Mari M, Shuvayu S. Mixed treatment comparison of infliximab with ustekinumab in patients with moderate to severe psoriasis. Br. J. Dermatol. . T. Fan, Merck and Co, Whitehouse Station, United States; 2011;165:e38–9.

811. Fan T, Bennett H, Smith N, Marin M, Sen S. Comparison of infliximab and ustekimumab for treatment of moderate to severe psoriasis: A mixed treatment meta-analysis. Value Heal. . T. Fan, Merck and Co., Inc., Whitehouse Station, United States; 2011;14:A410–1.

812. Foulkes AC, Grindlay DJC, Griffiths CEM, Warren RB. What’s new in psoriasis? An analysis of guidelines and systematic reviews published in 2009-2010. Clin. Exp. Dermatol. . A.C. Foulkes, Dermatology Centre, Salford Royal NHS Foundation Trust, University of Manchester, Manchester, M6 8HD, United Kingdom; 2011;36:585–8.

813. Freeman K. The two-compound formulation of calcipotriol and betamethasone dipropionate for treatment of moderately severe body and scalp psoriasis-an introduction. Curr. Med. Res. Opin. . K. Freeman, Bunny Hill Primary Care Centre, County Durham and Darlington NHS Foundation Trust, Sunderland Teaching Primary Care Trust, Sunderland, SR5 4BW, United Kingdom; 2011;27:197–203.

814. Fritz H, Kennedy D, Fergusson D, Fernandes R, Doucette S, Cooley K, et al. Vitamin A and retinoid derivatives for lung cancer: A systematic review and meta analysis. PLoS One . D. Seely, Department of Research and Epidemiology, The Canadian College of Naturopathic Medicine, Toronto, ON, Canada; 2011;6.

815. Georgiou I. Atlantis mystery and missing heritability. Gastric and Breast Cancer . I. Georgiou, Laboratory of Human Reproductive Genetics, Medical School, University of Ioannina, GR 451 10 Ioannina, Greece; 2011;10:139–45.

816. Gisondi P, Malara G, Ardigò M. The psoriatic patient profile for infliximab. Eur. Rev. Med. Pharmacol. Sci. . P. Gisondi, Department of Medicine, Section of Dermatology and Venereology, University of Verona, Verona, Italy; 2011;15:1445–51.

817. Gouldthorpe O, Catto-Smith AG, Alex G. Biologics in paediatric Crohn’s disease. Gastroenterol. Res. Pract. . A.G. Catto-Smith, Department of Gastroenterology and Clinical Nutrition, Royal Children’s Hospital, Parkville Melbourne, VIC 3052, Australia; 2011;

818. Huizinga T, Nigrovic P, Ruderman E, Schulze-Koops H. Association between biologic therapies for chronic plaque psoriasis and cardiovascular events: A meta-analysis of randomized controlled trials - Commentary. Int. J. Adv. Rheumatol. . T. Huizinga; 2011;9:141–2.

819. Jensen JD, Fujita M, Dellavalle RP. Validation of psoriasis clinical severity and outcome measures: Searching for a gold standard. Arch. Dermatol. . R. P. Dellavalle, Dermatology Service, Department of Veteran Affairs Medical Center, Denver, CO 80220, United States; 2011;147:95–8.

820. Juanola Roura X, Zarco Montejo P, Sanz Sanz J, Muñoz Fernández S, Mulero Mendoza J, Linares Ferrando LF, et al. Consensus Statement of the Spanish Society of Rheumatology on the management of biologic therapies in Spondyloarthritis except for Psoriatic Arthritis. Reumatol. Clin. . E. Loza Santamaría, Unidad de Investigación, Sociedad Española de Reumatología, Madrid, Spain; 2011;7:113–23.

821. Kriegel MA, Manson JE, Costenbader KH. Does Vitamin D Affect Risk of Developing Autoimmune Disease?: A Systematic Review. Semin. Arthritis Rheum. . K.H. Costenbader, Brigham and Women’s Hospital, Division of Rheumatology, Immunology and Allergy, PBB-B3, Boston, MA 02115, United States; 2011;40:512–31.e8.

822. Kurzeja M, Rudnicka L, Olszewska M. New interleukin-23 pathway inhibitors in dermatology: Ustekinumab, briakinumab, and secukinumab. Am. J. Clin. Dermatol. . L. Rudnicka, Department of Dermatology, CSK MSWiA, 02507 Warsaw, Poland; 2011;12:113–25.

823. Langham S, Langham J, Goertz HP, Ratcliffe M. Large-scale, prospective, observational studies in patients with psoriasis and psoriatic arthritis: A systematic and critical review. BMC Med. Res. Methodol. . S. Langham, PHMR consulting, London, UK.; 2011;11:32.

824. Li W, Han J, Hu F, Curhan G, Qureshi A. Psoriasis and risk of type 2 diabetes among women and men in the United States. J. Invest. Dermatol. . W. Li, Brigham and Women’s Hospital, Harvard Medical School, Boston, United States; 2011;131:S41.

825. Mao Z-F, Yang L-X, Mo X-A, Qin C, Lai Y-R, He N-Y, et al. Frequency of autoimmune diseases in myasthenia gravis: A systematic review. Int. J. Neurosci. . Z.-F. Mao, Department of Neurology, Third Affiliated Hospital, Guangxi Medical University, Nanning 530021, Guangxi, China; 2011;121:121–9.

826. Marquis P, Caron M, Emery M-P, Scott JA, Arnould B, Acquadro C. The role of health-related quality of life data in the drug approval processes in the us and Europe: A review of guidance documents and authorizations of medicinal products from 2006 to 2010. Pharmaceut. Med. . P. Marquis, Mapi Values USA, LLC, Boston, MA 02114, United States; 2011;25:147–60.

827. Maza A, Montaudié H, Sbidian E, Gallini A, Aractingi S, Aubin F, et al. Oral cyclosporin in psoriasis: A systematic review on treatment modalities, risk of kidney toxicity and evidence for use in non-plaque psoriasis. J. Eur. Acad. Dermatology Venereol. . A. Maza, Dermatology Department, Paul Sabatier University, Toulouse, France; 2011;25:19–27.

828. McGovern D, Cho J, Weersma R, Barrett J. Shared risk genes between inflammatory bowel disease (IBD) and otherimmune-mediated disorders: First results from the immunochipproject of the international ibd genetics consortium (IIBDGC). Inflamm. Bowel Dis. . D. McGovern, Cedars-Sinai Medical Center, Los Angeles, United States; 2011;17:S7–8.

829. Meeuwis KAP, de Hullu JA, Massuger LFAG, van de Kerkhof PCM, van Rossum MM. Genital psoriasis: A systematic literature review on this hidden skin disease. Acta Derm. Venereol. . K. A. P. Meeuwis, Departments of Dermatology, Radboud University Nijmegen Medical Centre, NL-6500 HB Nijmegen, Netherlands; 2011;91:5–11.

830. Meggitt SJ, Anstey A V, Mohd Mustapa MF, Reynolds NJ, Wakelin S. British Association of Dermatologists’ guidelines for the safe and effective prescribing of azathioprine 2011. Br. J. Dermatol. . S.J. Meggitt, Department of Dermatology, Royal Victoria Infirmary, Newcastle upon Tyne NE1 4LP, United Kingdom; 2011;165:711–34.

831. Menezes MD, McCarter R, Greene EA, Bauman NM. Status of propranolol for treatment of infantile hemangioma and description of a randomized clinical trial. Ann. Otol. Rhinol. Laryngol. . N.M. Bauman, Department of Otolaryngology-Head and Neck Surgery, Children’s National Medical Center, George Washington University School of Medicine, Washington, DC, United States; 2011;120:686–95.

832. Meyersburg D, Rödel RMW, Meyersburg AM, Schön MP, Mössner R. Nasal septum perforation under long-term therapy with low-dose methotrexate in a patient with psoriasis and psoriatic arthritis. JDDG - J. Ger. Soc. Dermatology . D. Meyersburg, Department of Dermatology, Georg-August-University, Göttingen, Germany; 2011;9:13.

833. Micha R, Imamura F, Wyler Von Ballmoos M, Solomon DH, Hernán MA, Ridker PM, et al. Systematic review and meta-analysis of methotrexate use and risk of cardiovascular disease. Am. J. Cardiol. . R. Micha, Department of Epidemiology, Harvard School of Public Health, Boston, MA, United States; 2011;108:1362–70.

834. Montaudié H, Sbidian E, Paul C, Maza A, Gallini A, Aractingi S, et al. Methotrexate in psoriasis: A systematic review of treatment modalities, incidence, risk factors and monitoring of liver toxicity. J. Eur. Acad. Dermatology Venereol. . H. Montaudié, Dermatology Department, Nice University, L’Archet II Hospital, Nice, France; 2011;25:12–8.

835. Muthalaly A, Ang DC, Hugenberg ST, Sampson R, Muthalaly A. Atypical persistent plaquelike skin rash of adult onset still’s disease. Case report and review of literature. Arthritis Rheum. . A. Muthalaly, Indiana University, School of Medicine, Indianapolis, United States; 2011;63.

836. Niazi AK, Niazi SK. Mindfulness-based stress reduction: A non-pharmacological approach for chronic illnesses. N. Am. J. Med. Sci. . A. Khan Niazi, Shifa College of Medicine/Shifa International Hospital, Islamabad, Pakistan; 2011;3:20–3.

837. Otuki MF, Reis RC, Cabrini D, Prudente AS, Horinouchi CDS, Correr CJ. Patient-reported outcomes in psoriasis research and practice. Br. J. Dermatol. . C.J. Correr, Pharmacy Department, Federal University of Parana, Curitiba Parana 80210-170, Brazil; 2011;165:1361–2.

838. Pareek A, Suthar M, Rathore GS, Bansal V. Feverfew (Tanacetum parthenium L.): A systematic review. Pharmacogn. Rev. . A. Pareek, Department of Pharmaceutical Science, L. M. College of Science and Technology (Pharmacy Wing), Jodhpur- 342 003, India; 2011;5:103–10.

839. Parisi R, Griffiths CEM, Ashcroft DM. Systematic review of the incidence and prevalence of psoriasis. Br. J. Dermatol. . R. Parisi, University of Manchester, School of Pharmacy and Pharmaceutical Sciences, Manchester, United Kingdom; 2011;165:e5.

840. Park H, del Rosso JQ. The emergence of mycophenolate mofetil in dermatology: From its roots in the world of organ transplantation to its versatile role in the dermatology treatment room. J. Clin. Aesthet. Dermatol. . H. Park, Valley Hospital Medical Center, Las Vegas, NV, United States; 2011;4.

841. Patel R V, Shelling ML, Prodanovich S, Federman DG, Kirsner RS. Psoriasis and vascular disease-risk factors and outcomes: A systematic review of the literature. J. Gen. Intern. Med. . R.S. Kirsner, Department of Dermatology and Cutaneous Surgery, University of Miami Miller, School of Medicine, Miami, FL 33136, United States; 2011;26:1036–49.

842. Paul C, Gallini A, Maza A, Montaudié H, Sbidian E, Aractingi S, et al. Evidence-based recommendations on conventional systemic treatments in psoriasis: Systematic review and expert opinion of a panel of dermatologists. J. Eur. Acad. Dermatology Venereol. . C. Paul, Dermatology Department, Paul Sabatier University, Toulouse, France; 2011;25:2–11.

843. Paul C, Ortonne J-P. Use of conventional systemic treatments in psoriasis: Systematic review and expert opinion of a panel of dermatologists. J. Eur. Acad. Dermatology Venereol. . C. Paul, Department of Dermatology, Paul Sabatier University, Larrey Hospital, Toulouse, France; 2011;25:1.

844. Pérez-Alvarez R, Díaz-Lagares C, García-Hernández F, Lopez-Roses L, Brito-Zerón P, Pérez-De-Lis M, et al. Hepatitis B virus (HBV) reactivation in patients receiving tumor necrosis factor (TNF)-targeted therapy: Analysis of 257 cases. Medicine (Baltimore). . M. Ramos-Casals, Servei de Malalties Autoimmunes, Hospital Clínic, 08036-Barcelona, Spain; 2011;90:359–71.

845. Perez-Alvarez R, Perez-de-Lis M, Diaz-Lagares C, Pego-Reigosa JM, Retamozo S, Bove A, et al. Interstitial Lung Disease Induced or Exacerbated by TNF-Targeted Therapies: Analysis of 122 Cases. Semin. Arthritis Rheum. . M. Ramos-Casals, Servei de Malalties Autoimmunes, Hospital Clínic, 08036 Barcelona, Spain; 2011;41:256–64.

846. Peyrin-Biroulet L, Loftus Jr. E V, Colombel J-F, Sandborn WJ. Long-term complications, extraintestinal manifestations, and mortality in adult Crohn’s disease in population-based cohorts. Inflamm. Bowel Dis. . E. V. Loftus Jr., Division of Gastroenterology and Hepatology, Mayo Clinic, Rochester, MN 55905, United States; 2011;17:471–8.

847. Ramos PS, Criswell LA, Moser KL, Comeau ME, Williams AH, Pajewski NM, et al. A comprehensive analysis of shared loci between systemic lupus erythematosus (SLE) and sixteen autoimmune diseases reveals limited genetic overlap. PLoS Genet. . P. S. Ramos, Department of Medicine, Medical University of South Carolina, Charleston, SC, United States; 2011;7.

848. Raval K, Lofland JH, Waters HC, Piech CT. Disease and treatment burden of psoriasis: Examining the impact of biologics. J. Drugs Dermatology . J.H. Lofland, Centocor Ortho Biotech, Inc., Horsham, PA 19044, United States; 2011;10:189–96.

849. Reino JG, Loza E, Andreu JL, Balsa A, Batlle E, Cañete JD, et al. Consensus statement of the spanish society of rheumatology on risk management of biologic therapy in rheumatic patients. Reumatol. Clin. . E. Loza, Unidad de Investigación, Sociedad Española de Reumatología, Madrid, Spain; 2011;7:284–98.

850. Riveira-Munoz E, He S-M, Escaramís G, Stuart PE, Hüffmeier U, Lee C, et al. Meta-analysis confirms the LCE3C-LCE3B deletion as a risk factor for psoriasis in several ethnic groups and finds interaction with HLA-Cw6. J. Invest. Dermatol. . X. Estivill, Genes and Disease Programme, Center for Genomic Regulation (CRG), Public Health and Epidemiology Network Biomedical Research Center (CIBERESP), Catalunya, Barcelona 08003, Spain; 2011;131:1105–9.

851. Rodgers M, Epstein D, Bojke L, Yang H, Craig D, Fonseca T, et al. Etanercept, infliximab and adalimumab for the treatment of psoriatic arthritis: A systematic review and economic evaluation. Health Technol. Assess. (Rockv). . M. Rodgers, Centre for Reviews and Dissemination, University of York, York, United Kingdom; 2011;15:134.

852. Ryan C, Daoud Y, Menter A. Biologic therapies for chronic plaque psoriasis and cardiovascular events: In reply. JAMA - J. Am. Med. Assoc. . C. Ryan, Department of Dermatology, Baylor Research Institute, Dallas, TX, United States; 2011;306:2095–6.

853. Ryan C, Leonardi CL, Krueger JG, Kimball AB, Strober BE, Gordon KB, et al. Association between biologic therapies for chronic plaque psoriasis and cardiovascular events: A meta-analysis of randomized controlled trials. JAMA - J. Am. Med. Assoc. . C. Ryan, Department of Dermatology, Baylor Research Institute, Dallas, TX 75246, United States; 2011;306:864–71.

854. Ryan C, Leonardi C, Krueger J, Kimball A, Strober B, Gordon K, et al. Antagonism of p40 cytokines in psoriasis patients may promote paradoxical cardiovascular risk. J. Invest. Dermatol. . C. Ryan, Baylor Research Institute, Dallas, United States; 2011;131:S83. Available from: <http://www.embase.com/search/results?subaction=viewrecord&from=export&id=L70645695>

855. Sbidian E, Maza A, Montaudié H, Gallini A, Aractingi S, Aubin F, et al. Efficacy and safety of oral retinoids in different psoriasis subtypes: A systematic literature review. J. Eur. Acad. Dermatology Venereol. . E. Sbidian, Dermatology Department, Saint-Louis Hospital, Paris, France; 2011;25:28–33.

856. Schram ME, Borgonjen RJ, Bik CMJM, Van Der Schroeff JG, Van Everdingen JJE, Spuls PI. Off-label use of azathioprine in dermatology: A systematic review. Arch. Dermatol. . M. E. Schram, Department of Dermatology, Academic Medical Center, University of Amsterdam, 1100 DE Amsterdam, Netherlands; 2011;147:474–88.

857. Shackleton B, Bundy C, Griffiths C. Can stress reduction techniques improve the clinical severity of psoriasis? A systematic review. Br. J. Dermatol. . B. Shackleton, University of Manchester, Manchester, United Kingdom; 2011;165:e20–1.

858. Shapiro S, Heremans A, Mays DA, Martin AL, Hernandez-Medina M, Lanes S. Use of topical tretinoin and the development of noncutaneous adverse events: Evidence from a systematic review of the literature. J. Am. Acad. Dermatol. . S. Lanes, United BioSource Corp, Lexington, MA 02420, United States; 2011;65:1194–201.

859. Singal A, Khanna D. Onychomycosis: Diagnosis and management. Indian J. Dermatol. Venereol. Leprol. . A. Singal, B-14, Law Apartments, Karkardooma, Delhi-110 092, India; 2011;77:659–72.

860. Sivakumaran S, Agakov F, Theodoratou E, Prendergast JG, Zgaga L, Manolio T, et al. Abundant pleiotropy in human complex diseases and traits. Am. J. Hum. Genet. . H. Campbell, Centre for Population Health Sciences, University of Edinburgh, Edinburgh EH8 9AG, United Kingdom; 2011;89:607–18.

861. Smolen JS, Emery P. Infliximab: 12 years of experience. Arthritis Res. Ther. . J.S. Smolen, Division of Rheumatology, Department of Medicine III, Medical University of Vienna, Vienna, Austria; 2011;13.

862. Szepietowski J, Kaczor M, Pawlik D, Wojcik R, Reich A. Systematic review and mixed treatment comparison of biologic therapies in psoriasis. J. Invest. Dermatol. . J. Szepietowski, Department of Dermatology, Venereology and Allergology, Wroclaw Medical University, Wroclaw, Poland; 2011;131:S38.

863. Tan J. Informed shared decision making in psoriasis management. Australas. J. Dermatol. . J. Tan, Department of Medicine, University of Western Ontario, Canada; 2011;52:11.

864. Tan JY, Li S, Yang K, Ma B, Chen W, Zha C, et al. Ustekinumab, a human interleukin-12/23 monoclonal antibody, in patients with psoriasis: A meta-analysis. J. Dermatolog. Treat. . J.Y. Tan, Institute of Immunology, School of Basic Medical Science, Lanzhou University, Lanzhou 730000, GanSu, China; 2011;22:323–36.

865. Tejasvi T, Stuart PE, Chandran V, Voorhees JJ, Gladman DD, Rahman P, et al. TNFAIP3 polymorphisms and age at onset are associated with response to tumour necrosis factor blockade in psoriasis. Br. J. Dermatol. . T. Tejasvi, Department of Dermatology, University of Michigan, Ann Arbor, United States; 2011;165:e9–10.

866. Tzellos T, Kyrgidis A, Toulis K. Biologic therapies for chronic plaque psoriasis and cardiovascular events. JAMA - J. Am. Med. Assoc. . T. Tzellos, Department of Pharmacology and Clinical Pharmacology, Aristotle University of Thessaloniki, Thessaloniki, Greece; 2011;306:2095.

867. Van De Kerkhof P, De Peuter R, Ryttov J, Jansen JP. Mixed treatment comparison of a two-compound formulation (TCF) product containing calcipotriol and betamethasone dipropionate with other topical treatments in psoriasis vulgaris. Curr. Med. Res. Opin. . J. Ryttov, LEO Pharma, Princes Risborough, HP27 9RR, United Kingdom; 2011;27:225–38.

868. Weiss ST, Silverman EK. Pro: Genome-wide association studies (GWAS) in asthma. Am. J. Respir. Crit. Care Med. . S.T. Weiss, Channing Laboratory, Brigham and Women’s Hospital, Boston, MA, United States; 2011;184:631–3.

869. Wu D, Wu Y, Liu JL, Wang B, Zhang XD. Association between HLA-Cw*0602 polymorphism and psoriasis risk: A meta-analysis. Genet. Mol. Res. . X.D. Zhang, Department of Dermatology, Beihua Affiliated Hospital, Jilin, China; 2011;10:3109–20.

870. Wu Y, Wang B, Liu JL, Gao XH, Chen HD, Li YH. Association of -619C/T polymorphism in CDSN gene and psoriasis risk: A meta-analysis. Genet. Mol. Res. . Y.H. Li, Department of Dermatology, The First Affiliated Hospital, China Medical University, Shenyang, China; 2011;10:3632–40.

871. Yakasai IA, Thomson AJ, Fitzsimons C. Specific dermatoses of pregnancy: A review. West Afr. J. Med. . I. A. Yakasai, Department of Obstetrics Gynaecology, Royal Alexandra Hospital Paisley, PA2 9PN, United Kingdom; 2011;30:239–44.

872. Zhu KJ, He SM, Sun LD, Hu D, Cheng H, Zhang Z, et al. Smoking and psoriasis: A meta-analysis of case-control studies. J. Dermatol. Sci. . S. Yang, Anhui Medical University, Hefei, Anhui, China; 2011;63:126–8.

**2010**

873. Alandete JC. Cost-effectiveness of biologics in psoriasis in two la countries-comparison with the European experience. Value Heal. . J.C. Alandete, Janssen-Cilag, Bogota, Colombia; 2010;13:A467–8.

874. Bailey EE, Alikhan A, Armstrong AW. Combination treatments for psoriasis: A systematic review. J. Invest. Dermatol. . E.E. Bailey, Harvard School of Public Health, Boston, United States; 2010;130:S52.

875. Bailey J, Whitehair B. Topical treatments for chronic plaque psoriasis. Am. Fam. Physician . J. Bailey, David Grant Medical Center, Travis Air Force Base, Vacaville, CA, United States; 2010;81:596–7.

876. Beani J-C, Jeanmougin M. Narrow-band UVB therapy in psoriasis vulgaris: Good practice guideline and recommendations of the French Society of Photodermatology. Ann. Dermatol. Venereol. . J.-C. Beani, Clinique universitaire de dermato-vénéréologie, photobiologie et allergologie, Pôle pluridisciplinaire de médecine A.-Michallon, CHU de Grenoble, 38043 Grenoble cedex, France; 2010;137:21–31.

877. Bronsard V, Paul C, Prey S, Puzenat E, Gourraud P-A, Aractingi S, et al. What are the best outcome measures for assessing quality of life in plaque type psoriasis? A systematic review of the literature. J. Eur. Acad. Dermatology Venereol. . J.-P. Ortonne, Dermatology Department, Nice University, L’Archet II Hospital, Nice, France; 2010;24:17–22.

878. Cantini F, Niccoli L, Nannini C, Kaloudi O, Bertoni M, Cassarà E. Psoriatic arthritis: A systematic review. Int. J. Rheum. Dis. . F. Cantini, Department of Internal Medicine, Rheumatology Unit, Hospital Misericordia e Dolce, Prato, Italy; 2010;13:300–17.

879. Collamer AN, Battafarano DF. Psoriatic Skin Lesions Induced by Tumor Necrosis Factor Antagonist Therapy: Clinical Features and Possible Immunopathogenesis. Semin. Arthritis Rheum. . A.N. Collamer, Rheumatology Service, Brooke Army Medical Center, Fort Sam Houston, TX 78234, United States; 2010;40:233–40.

880. de Jager MEA, de Jong EMGJ, van de Kerkhof PCM, Seyger MMB. Efficacy and safety of treatments for childhood psoriasis: A systematic literature review. J. Am. Acad. Dermatol. . M.E.A. de Jager, Department of Dermatology, Radboud University Nijmegen Medical Center, Nijmegen, Netherlands; 2010;62:1013–30.

881. Díaz-Lagares C, Belenguer R, Ramos-Casals M. Systematic review on the use of adalimumab in autoinmune. Efficacy and safety in 54 patients. Reumatol. Clin. . M. Ramos-Casals, Laboratorio de Enfermedades Autoinmunes Josep Font, Servicio de Enfermedades Autoinmunes, IDIBAPS, Barcelona, Spain; 2010;6:121–7.

882. Dommasch ED, Abuabara K, Nguyen J, Troxel AB, Gelfand JM. The safety of tumor necrosis factor antagonists in psoriasis: A systematic review and meta-analysis of randomized controlled trials. J. Invest. Dermatol. . E.D. Dommasch, Dermatology, University of Pennsylvania, Philadelphia, United States; 2010;130:S59.

883. Espinoza-Chavez VE. Meta-analysis: Psoriasis and BMI. J. Eur. Acad. Dermatology Venereol. . V.E. Espinoza-Chavez, Facultad de Medicina, UNAM, Mexico City, Mexico; 2010;24:54.

884. Feldman S, Yentzer B. Topical clobetasol propionate in the treatment of psoriasis. J. Am. Acad. Dermatol. . S. Feldman, WFU School of Medicine, Winston Salem, United States; 2010;62:AB140.

885. Finckh A. Comparative effectiveness of rheumatoid arthritis therapies. Curr. Rheumatol. Rep. . A. Finckh, Division of Rheumatology, Department of Internal Medicine, University Hospital of Geneva, 1211 Geneva, Switzerland; 2010;12:348–54.

886. Garcia-Bustinduy MC, Gonzalez-Hernandez S, Garcia CR, De Paz NM, Ferrer PC, Rodriguez MS, et al. Cutaneous reactions in biological treatments. J. Eur. Acad. Dermatology Venereol. . M.C. Garcia-Bustinduy, Dermatology, Hospital Universitario De Canarias, Santa Cruz de Tenerife, Spain; 2010;24:27–8.

887. Garg AD, Nowis D, Golab J, Agostinis P. Photodynamic therapy: Illuminating the road from cell death towards anti-tumour immunity. Apoptosis . J. Golab, Department of Immunology, Center of Biostructure Research, Medical University of Warsaw, Warsaw 02-097, Poland; 2010;15:1050–71.

888. Gaujoux-Viala C, Giampietro C, Gaujoux T, Ea H-K, Orcel P, Liote F. Scleritis: A new paradoxical effect of etanercept? A series of 3 cases and a systematic literature review of etanercept-associated inflammatory eye disease. Arthritis Rheum. . C. Gaujoux-Viala, Paris 6 - Pierre et Marie Curie University, Rheumatology, Pitié-Salpêtrière Hospital, Paris, France; 2010;62:413.

889. Herfarth HH, Osterman MT, Isaacs KL, Lewis JD, Sands BE. Efficacy of methotrexate in ulcerative colitis: Failure or promise. Inflamm. Bowel Dis. . H. H. Herfarth, Division of Gastroenterology and Hepatology, Department of Medicine, University of North Carolina, Chapel Hill, NC 27599, United States; 2010;16:1421–30.

890. Ioannidis J, Karassa F. The need to consider the wider agenda in systematic reviews and meta-analyses. BMJ . J. Ioannidis, Department of Hygiene and Epidemiology, University of Ioannina School of Medicine, Ioannina, Greece; 2010;341:762–5.

891. Jensen P, Skov L, Zachariae C. Systemic combination treatment for psoriasis: A review. Acta Derm. Venereol. . P. Jensen, Department of Dermato-Allergology, Copenhagen University Hospital Gentofte, DK-2900 Hellerup, Denmark; 2010;90:341–9.

892. Kirino Y, Ombrello MJ, Gul A, Wang K, Meguro A, Yang B, et al. Homozygous 3.2kb deletion in LEPREL1 (P3H2) intron 1 reduces cytokine production and protects from multiple inflammatory diseases. Arthritis Rheum. . Y. Kirino, Department of Ocular Inflammation and Immunology, Hokkaido University, Graduate School of Medicine, Japan; 2010;62:2217.

893. Kwok T, Loo WJ, Guenther L. Psoriasis and multiple sclerosis: Is there a link? J. Cutan. Med. Surg. . W. J. Loo, St Joseph’s Hospital, London, ON N6A 4L6, Canada; 2010;14:151–5.

894. Langham S, Langham J, Goertz HP, Ratcliffe M. Large-scale, prospective, observational studies in patients with psoriasis and psoriatic arthritis: A systematic and critical review. Value Heal. . S. Langham, PHMR Consulting, London, United Kingdom; 2010;13:A217.

895. Lázaro P, Blasco AJ, Ferrándiz C, García A, Liso J. Efficiency (cost/efficacy) of biologic agents in the treatment of moderate to severe psoriasis. Value Heal. . P. Lázaro, Advanced Techniques in Health Services Research (TAISS), Madrid, Spain; 2010;13:A147.

896. Malhi GS, Adams D, Berk M. The pharmacological treatment of bipolar disorder in primary care. Med. J. Aust. . G. S. Malhi, CADE Clinic, Department of Psychiatry, Royal North Shore Hospital, Sydney, NSW, Australia; 2010;193:S24–30.

897. Mars TS, Abbey H. Mindfulness meditation practise as a healthcare intervention: A systematic review. Int. J. Osteopath. Med. . T.S. Mars, Research Centre, The British School of Osteopathy, London, SE1 1JE, United Kingdom; 2010;13:56–66.

898. Menon K, Van Voorhees AS, Bebo Jr. BF, Gladman DD, Hsu S, Kalb RE, et al. Psoriasis in patients with HIV infection: From the Medical Board of the National Psoriasis Foundation. J. Am. Acad. Dermatol. . B.E. Strober, Department of Dermatology, New York University, New York, NY, United States; 2010;62:291–9.

899. Mrowietz U, Guérin A, Mulani P, Gupta S. Comorbidity prevalence in psoriasis patients: A meta-analysis. J. Am. Acad. Dermatol. . U. Mrowietz, University Medical Center Schleswig-Holstein, Campus Kiel, Germany; 2010;62:AB123.

900. Naci H, Green J, Prasad M, Fleurence R. Biological agents for the treatment of nail psoriasis: A systematic review of the literature. Value Heal. . H. Naci, United BioSource Corporation, London, United Kingdom; 2010;13:A460.

901. Nybaek H, Jemec GBE. Skin problems in stoma patients. J. Eur. Acad. Dermatology Venereol. . H. Nybaek, Department of Dermatology, University of Copenhagen, Roskilde Hospital, Denmark; 2010;24:249–57.

902. Ormerod AD, Campalani E, Goodfield MJD. British Association of Dermatologists guidelines on the efficacy and use of acitretin in dermatology. Br. J. Dermatol. . A. D. Ormerod, Department of Dermatology, University of Aberdeen, Foresterhill, Aberdeen AB9 2ZB, United Kingdom; 2010;162:952–63.

903. Papp K. Clinical development of onercept, a tumor necrosis factor binding protein, in psoriasis. Curr. Med. Res. Opin. . K. Papp, Probity Medical Research Inc., Waterloo, ON N2J 1C4, Canada; 2010;26:2287–300.

904. Paul C, Gourraud P-A, Bronsard V, Prey S, Puzenat E, Aractingi S, et al. Evidence-based recommendations to assess psoriasis severity: Systematic literature review and expert opinion of a panel of dermatologists. J. Eur. Acad. Dermatology Venereol. . C. Paul, Dermatology Department, Paul Sabatier University, Toulouse, France; 2010;24:2–9.

905. Paul C, Ortonne J-P. Introduction: Psoriasis evaluation in clinical practice: Systematic review and expert opinion. J. Eur. Acad. Dermatology Venereol. . C. Paul, Department of Dermatology, Paul Sabatier University, Larrey Hospital, Toulouse, France; 2010;24:1.

906. Pedraz J, Daudén E. Psoriatic arthritis and etanercept. Actas Dermosifiliogr. . J. Pedraz, Servicio de Dermatología, Hospital Clínico San Carlos, Madrid, Spain; 2010;101:26–34.

907. Pietrzik K, Bailey L, Shane B. Folic acid and l-5-methyltetrahydrofolate: Comparison of clinical pharmacokinetics and pharmacodynamics. Clin. Pharmacokinet. . K. Pietrzik, Institute of Nutrition and Food Sciences, University of Bonn, D-53115 Bonn, Germany; 2010;49:535–48.

908. Piruzian E, Bruskin S, Ishkin A, Abdeev R, Moshkovskii S, Melnik S, et al. Integrated network analysis of transcriptomic and proteomic data in psoriasis. BMC Syst. Biol. . E. Piruzian, Vavilov Institute of General Genetics, Russian Academy of Sciences, Gubkina St, 3 GSP-1, 119991 Moscow, Russia.; 2010;4:41.

909. Powers J, Martin R. Incidence of serious infectious events with methotrexate treatment: Metaanalysis of randomized controlled trials. J. Am. Acad. Dermatol. . J. Powers, Grand Rapids Medical Education and Research Center, Grand Rapids, United States; 2010;62:AB4.

910. Prey S, Paul C, Bronsard V, Puzenat E, Gourraud P-A, Aractingi S, et al. Cardiovascular risk factors in patients with plaque psoriasis: A systematic review of epidemiological studies. J. Eur. Acad. Dermatology Venereol. . C. Paul, Dermatology Department, Paul Sabatier University, Larrey Hospital, Toulouse, France; 2010;24:23–30.

911. Prey S, Paul C, Bronsard V, Puzenat E, Gourraud P-A, Aractingi S, et al. Assessment of risk of psoriatic arthritis in patients with plaque psoriasis: A systematic review of the literature. J. Eur. Acad. Dermatology Venereol. . C. Paul, Dermatology Department, Paul Sabatier University, Larrey Hospital, Toulouse, France; 2010;24:31–5.

912. Price S, James C, Deighton C. Methotrexate use and alcohol. Clin. Exp. Rheumatol. . C. Deighton, Department of Rheumatology, Royal Derby Hospital, Derby DE22 3NE, United Kingdom; 2010;28:S114–6. Available from:

913. Puzenat E, Bronsard V, Prey S, Gourraud P-A, Aractingi S, Bagot M, et al. What are the best outcome measures for assessing plaque psoriasis severity? A systematic review of the literature. J. Eur. Acad. Dermatology Venereol. . F. Aubin, Franche Comté University, Dermatology Department, Besançon, France; 2010;24:10–6.

914. Raap U, Ständer S. Pruritus and skin: New facts on pruritus induction. Allergo J. . S. Ständer, Kompetenzzentrum Pruritus, Klinik und Poliklinik für Hautkrankheiten, Universitätsklinikum Münster, 4814g Münster, Germany; 2010;19:58–65.

915. Ramos PS, Gaffney PM, Criswell LA, Comeau ME, Williams AH, Graham RR, et al. Meta-analysis of autoimmune variants shared between systemic lupus erythematosus (SLE) and 16 other diseases identifies novel SLE loci. Arthritis Rheum. . P.S. Ramos, UCLA, School of Medicine, Los Angeles, United States; 2010;62:500.

916. Rioux JD. International inflammatory bowel disease genetics consortium identifies >50 genetic risk factors for ulcerative colitis. Gastroenterology . J.D. Rioux; 2010;139:e19.

917. Rodríguez Lozano C. Safety of biological therapies: New data from BIOBADASER. Reumatol. Clin. . C. Rodríguez Lozano, Servicio de Reumatología, Hospital Universitario de Gran Canaria Dr. Negrín, Las Palmas de Gran Canaria, Spain; 2010;6:S1–6.

918. Schöfer H, Simonsen L. Fusidic acid in dermatology: An updated review. Eur. J. Dermatology . H. Schöfer, Dept. of Dermatovenereology, University Hospital der J. W. Goethe-University, 60590 Frankfurt/Main, Germany; 2010;20:6–15.

919. Schram ME, Spuls PI, Bos JD. Off-label use of efalizumab in dermatology. Expert Rev. Dermatol. . M. E. Schram, Department of Dermatology, Academic Medical Center, University of Amsterdam, 1100 DE Amsterdam, Netherlands; 2010;5:535–47.

920. Shale MJ, Seow CH, Coffin CS, Kaplan GG, Panaccione R, Ghosh S. Review article: Chronic viral infection in the anti-tumour necrosis factor therapy era in inflammatory bowel disease. Aliment. Pharmacol. Ther. . S. Ghosh, Division of Gastroenterology, TRW Centre, University of Calgary, Calgary, AB T2N 4Z6, Canada; 2010;31:20–34.

921. Signorovitch JE, Wu EQ, Yu AP, Gerrits CM, Kantor E, Bao Y, et al. Comparative effectiveness without head-to-head trials: A method for matching-adjusted indirect comparisons applied to psoriasis treatment with adalimumab or etanercept. Pharmacoeconomics . J. E. Signorovitch, Analysis Group, Inc., Boston, MA 02199, United States; 2010;28:935–45.

922. Simonart T, Heenen M, Lejeune O. Epidermal kinetic alterations required to generate the psoriatic phenotype: A reappraisal. Cell Prolif. . T. Simonart, Department of Dermatology, Hôpital Universitaire Erasme, B-1070 Brussels, Belgium; 2010;43:321–5.

923. Spuls PI, Lecluse LLA, Poulsen M-LNF, Bos JD, Stern RS, Nijsten T. How good are clinical severity and outcome measures for psoriasis: Quantitative evaluation in a systematic review. J. Invest. Dermatol. . P. I. Spuls, Department of Dermatology, A0-227, Academic Medical Center, 1100 DD Amsterdam, Netherlands; 2010;130:933–43.

924. Storage SS, Agrawal H, Furst DE. Description of the efficacy and safety of three new biologics in the treatment of rheumatoid arthritis. Korean J. Intern. Med. . D. E. Furst, Division of Rheumatology, UCLA David Geffen School of Medicine, Los Angeles, CA 90095-1670, United States; 2010;25:1–17.

925. Stuart PE, Nair RP, ELinghaus E, Ding J, Tejasvi T, GudjonSon JE, et al. Genome-wide asociation analysis identifies three psoriasis susceptibility loci. Nat. Genet. . J. T. Elder, Department of Dermatology, University of Michigan Medical School, Ann Arbor, MI, United States; 2010;42:1000–4.

926. Sun Y, Wu Y, Chen L-H, Liu Y-B, Gao X-H. Efficacy and safety of adalimumab for plaque psoriasis: A systematic review. Chinese J. Evidence-Based Med. . Y. Sun, Department of Dermatology, First Hospital of China Medical University, Ministry of Health, Shenyang 110001, China; 2010;10:1085–95.

927. Tatar M, Sarioz F. Cost efficacy of ustekinumab in treatment of moderate to severe plaque psoriasis in Turkey. Value Heal. . M. Tatar, Hacettepe University, Ankara, Turkey; 2010;13:A146–7.

928. Vyas R, Juruwan R, Rademaker M, Weilert F, Yung A. Use of transient elastography for non-invasive monitoring of methotrexate induced liver fibrosis. Australas. J. Dermatol. . R. Vyas, Department of Dermatology, Waikato Hospital, Hamilton, New Zealand; 2010;51:A46–7.

929. Wiwanitkit V. LCE3C_LCE3B-del genotype and psoriasis: A summative meta-analysis. Acta Dermatovenerologica Croat. . V. Wiwanitkit, Wiwanitkit House, Bangkhae, Bangkok 10160, Thailand; 2010;18:130.

930. Yang S, Liu J, Zhang C, Zhang X. Meta-GWAS of psoriasis reveals genetic heterogeneity between Chinese and European populations and identifies two new susceptibility loci. J. Dermatol. . S. Yang, Key Laboratory of Dermatology, Anhui Medical University, Ministry of Education, China; 2010;37:102.

931. Poster Abstracts - American Academy of Dermatology 68th Annual Meeting. J. Am. Acad. Dermatol. . 2010;62.

**2009**

932. Albrecht J, Werth VP, Bigby M. The role of case reports in evidence-based practice, with suggestions for improving their reporting. J. Am. Acad. Dermatol. . J. Albrecht, Department of Medicine, Division of Dermatology, John H. Stroeger Jr Hospital of Cook County, Chicago, IL, United States; 2009;60:412–8.

933. Andronis L, Barton P, Bryan S. Sensitivity analysis in economic evaluation: An audit of NICE current practice and a review of its use and value in decision-making. Health Technol. Assess. (Rockv). . S. Bryan, Centre for Clinical Epidemiology and Evaluation, University of British Columbia, Vancouver, BC, Canada; 2009;13:ix – 84.

934. Atzeni F, Sarzi-Puttini P. Anti-cytokine antibodies for rheumatic diseases. Curr. Opin. Investig. Drugs . F. Atzeni, L Sacco University Hospital, Rheumatology Unit, 20127, Milan, Italy; 2009;10:1204–11.

935. Bansback N, Sizto S, Sun H, Feldman S, Willian MK, Anis A. Efficacy of systemic treatments for moderate to severe plaque psoriasis: Systematic review and meta-analysis. Dermatology . A. Anis, School of Population and Public Health, Faculty of Medicine UBC, James Mather Building, Vancouver, BC V6T 1Z3, Canada; 2009;219:209–18.

936. Blasco AJ, Lázaro P, Ferrándiz C, García-Díez A, Liso J. Efficiency of biologic agents in the treatment of moderate to severe psoriasis. Actas Dermosifiliogr. . A. J. Blasco, Técnicas Avanzadas de Investigación en Servicios de Salud (TAISS), 28034 Madrid, Spain; 2009;100:792–803.

937. Braun J, Baraliakos X. Treatment of ankylosing spondylitis and other spondyloarthritides. Curr. Opin. Rheumatol. . J. Braun, Rheumazentrum Ruhrgebiet, 44652 Herne, Germany; 2009;21:324–34.

938. Bray A, Barnova I, Przemioslo R, Kennedy CTC. Could transient elastography reduce the need for Liver biopsy? Br. J. Dermatol. . A. Bray, University Hospitals Bristol NHS Foundation Trust, Bristol, United Kingdom; 2009;161:10–1.

939. Brown BC, Warren RB, Grindlay DJC, Griffiths CEM. What’s new in psoriasis? Analysis of the clinical significance of systematic reviews on psoriasis published in 2007 and 2008. Clin. Exp. Dermatol. . C. E. M. Griffiths, Dermatological Sciences, Salford Royal Hospital, University of Manchester, Manchester, M6 8HD, United Kingdom; 2009;34:664–7.

940. Cantini F, Nannini C, Niccoli L. Bioboosters in the treatment of rheumatic diseases: A comprehensive review of currently available biologics in patients with rheumatoid arthritis, ankylosing spondylitis and psoriatic arthritis. Open Access Rheumatol. Res. Rev. . F. Cantini, Second Division of Medicine, Rheumatology Unit, Hospital of Prato, 159100, Prato, Italy; 2009;1:163–78.

941. Casanova JM, Sanmartín V, Soria X, Ferran M, Pujol RM, Ribera M. Topical treatment of psoriasis. Piel . J. M. Casanova, Servicio de Dermatología, Hospital Universitari Arnau de Vilanova, Lleida, Spain; 2009;24:556–67.

942. Chiang NYZ, Panting K, Parslew RAG. Clinical applicability of T-SPOT. TB screening prior to initiation of antitumour necrosis factor-α therapy. Br. J. Dermatol. . K. Panting, Royal Liverpool and Broadgreen University Hospitals, Liverpool, Merseyside, United Kingdom; 2009;161:43.

943. De Peuter R, Beriot-Mathiot A, Eijgelshoven I, Stam W. Efficacy of psoriasis treatments: Systematic literature review and Bayesian metaanalysis. J. Am. Acad. Dermatol. . R. De Peuter, Mapi Values Netherlands, Houten, Netherlands; 2009;60:AB177.

944. Diener HC, Hartung HP, Bien CG, Hacke W, Ringelstein EB, Ludolph A, et al. Clinical trials in neurology in Germany 2008. Aktuelle Neurol. . H. C. Diener, Universitätsklinik Essen, 45147 Essen; 2009;36:8–18.

945. Dommasch E, Gelfand JM. Is there truly a risk of lymphoma from biologic therapies? Dermatol. Ther. . J. M. Gelfand, University of Pennsylvania, Department of Dermatology, 2 Maloney Building, Philadelphia, PA 19104, United States; 2009;22:418–30.

946. Falagas ME, Zarkadoulia E, Rafailidis PI. The therapeutic effect of balneotherapy: Evaluation of the evidence from randomised controlled trials. Int. J. Clin. Pract. . M. E. Falagas, Alfa Institute of Biomedical Sciences (AIBS), 151 23 Marousi, Greece; 2009;63:1068–84.

947. Feily A, Namazi MR. Aloe vera in dermatology: A brief review. G. Ital. di Dermatologia e Venereol. . A. Feily, Resident of Dermatology, Jondishapur University of Medical Sciences, Ahvaz, Iran; 2009;144:85–91.

948. Feldman SR, Yentzer BA. Topical clobetasol propionate in the treatment of psoriasis: A review of newer formulations. Am. J. Clin. Dermatol. . S. R. Feldman, Wake Forest University, School of Medicine, Medical Center Blvd, Winston-Salem, NC 27157-1071, United States; 2009;10:397–406.

949. Forabosco P, Bouzigon E, Ng MY, Hermanowski J, Fisher SA, Criswell LA, et al. Meta-analysis of genome-wide linkage studies across autoimmune diseases. Eur. J. Hum. Genet. . C.M. Lewis, King’s College London School of Medicine, Department of Medical and Molecular Genetics, London, United Kingdom; 2009;17:236–43.

950. Frankel AJ, Van Voorhees AS, Hsu S, Korman NJ, Lebwohl MG, Bebo Jr. BF, et al. Treatment of psoriasis in patients with hepatitis C: From the Medical Board of the National Psoriasis Foundation. J. Am. Acad. Dermatol. . A.B. Gottlieb, Department of Dermatology, Tufts Medical Center, Boston, MA, United States; 2009;61:1044–55.

951. Gladman DD. Psoriatic arthritis. Dermatol. Ther. . D. D. Gladman, FRCPC, Centre for Prognosis Studies in the Rheumatic Diseases, TorontoWestern Hospital, Toronto, ON M5T 2S8; 2009;22:40–55.

952. Godse K V. Chronic urticaria and treatment options. Indian J. Dermatol. . K. V. Godse, Shree Skin Centre, Nerul, Navi Mumbai - 400 706, India; 2009;54:310–2.

953. Haslund P, Lee RA, Jemec GBE. Treatment of hidradenitis suppurativa with tumour necrosis factor-α inhibitors. Acta Derm. Venereol. . P. Haslund, Department of Dermatology, Roskilde Hospital, University of Copenhagen, DK-4000 Roskilde, Denmark; 2009;89:595–600.

954. Hawkins NS, Huntley A, Eaton J. Meta-analysis of biologic therapies for the treatment of moderate to severe psoriasis. Value Heal. . N.S. Hawkins, Oxford Outcomes (UK), Oxford, United Kingdom; 2009;12:A74–5.

955. Karaderi T, Harvey D, Farrar C, Appleton LH, Stone MA, Sturrock RD, et al. Association between the interleukin 23 receptor and ankylosing spondylitis is confirmed by a new UK case-control study and meta-analysis of published series. Rheumatology . J.J. Pointon, Oxford University, Institute of Musculoskeletal Science, Botnar Research Centre, Oxford OX3 7LD, United Kingdom; 2009;48:386–9.

956. Kircik LH, Weinberg JM. Critical reviews of clinical data: Focus on T cell agents for the treatment of psoriasis. J. Drugs Dermatology . L.H. Kircik, Derm Research, PLLC, Louisville, KY 40217, United States; 2009;8:6–15.

957. Ko JM, Gottlieb AB, Kerbleski JF. Induction and exacerbation of psoriasis with TNF-blockade therapy: A review and analysis of 127 cases. J. Dermatolog. Treat. . J. F. Kerbleski, Department of Dermatology, Tufts Medical Center, Box 114, Boston, MA 02111, United States; 2009;20:100–8.

958. Levy-Roy A, Porcher R, De Fonclare A-L, Morel P, Dupuy A. Efficacité des anti-TNF-α dans le psoriasis: Revue systématique et représentation graphique. Ann. Dermatol. Venereol. . A. Dupuy, Université Paris-7 Denis-Diderot, Paris, France; 2009;136:315–22.

959. Lima XT, Seidler EM, Lima HC, Kimball AB. Long-term safety of biologics in dermatology. Dermatol. Ther. . A. B. Kimball, Boston, MA 02114; 2009;22:2–21.

960. Ma C, Panaccione R, Heitman SJ, Devlin SM, Ghosh S, Kaplan GG. Systematic review: The short-term and long-term efficacy of adalimumab following discontinuation of infliximab. Aliment. Pharmacol. Ther. . G. G. Kaplan, Teaching Research and Wellness Center, Calgary, AB T2N 4N1, Canada; 2009;30:977–86.

961. Maringer B, Zietemann V, Ratzinger G, Siebert U. Effectiveness of omega-3-fatty acids in psoriasis: A systematic review. Aktuel. Ernahrungsmed. . B. Maringer, Department of Public Health, Medical Decision Making and Health Technology Assessment, UMIT-University of Health Sciences, Medical Informatics and Technology, Hall i.T., Austria; 2009;34:195–200.

962. Naldi L, Rzany B. Psoriasis (chronic plaque). BMJ Clin. Evid. . 2009;2009.

963. Olivieri I, D’Angelo S, Palazzi C, Padula A. Treatment strategies for early psoriatic arthritis. Expert Opin. Pharmacother. . I. Olivieri, San Carlo Hospital of Potenza and Madonna Delle Grazie Hospital of Matera, Rheumatology Department, 85100, Potenza, Italy; 2009;10:271–82.

964. Poulin Y, Langley R, Teixeira HD, Martel M-J, Cheung S. Biologics in the treatment of psoriasis: Clinical and economic overview. J. Cutan. Med. Surg. . R.G. Langley, Queen Elizabeth II Health Sciences Centre, Division of Dermatology, Dalhousie University, Halifax, NS B3H 2Y9, Canada; 2009;13:S49–57.

965. Prey S, Paul C. Effect of folic or folinic acid supplementation on methotrexate-associated safety and efficacy in inflammatory disease: A systematic review. Br. J. Dermatol. . S. Prey, Department of Dermatology, Paul Sabatier University, Purpan Hospital, 31 059 Toulouse Cedex 9, France; 2009;160:622–8.

966. Qureshi SU, Pyne JM, Magruder KM, Schulz PE, Kunik ME. The link between post-traumatic stress disorder and physical comorbidities: A systematic review. Psychiatr. Q. . M. E. Kunik, Houston Center for Quality of Care and Utilization Studies, Health Services Research and Development Service, Michael E. DeBakey Veterans Affairs Medical Center (152), Houston, TX 77030, United States; 2009;80:87–97.

967. Ritchlin CT, Kavanaugh A, Gladman DD, Mease PJ, Helliwell P, Boehncke W-H, et al. Treatment recommendations for psoriatic arthritis. Ann. Rheum. Dis. . C. T. Ritchlin, Clinical Immunology Research Center, University of Rochester Medical Center, Rochester, NY 14642, United States; 2009;68:1387–94.

968. Schmajuk G, Krishnan E. Real or perceived conflicts of interest in comparative effectiveness research. Arthritis Rheum. . G. Schmajuk, Stanford University, San Francisco, United States; 2009;60:1015.

969. Schuh A. Evidence of the efficacy of climatotherapy and thalassotherapy - A review. Schweizerische Zeitschrift fur GanzheitsMedizin . A. Schuh, Institut für Gesundheits- und Rehabilitationswissenschaften, Ludwig-Maximilians-Universität, DE-81377 München, Germany; 2009;21:96–104.

970. Sizto S, Bansback N, Feldman SR, Willian MK, Anis AH. Economic evaluation of systemic therapies for moderate to severe psoriasis. Br. J. Dermatol. . A. H. Anis, School of Population and Public Health, University of British Columbia, Vancouver, BC V6T 1Z3, Canada; 2009;160:1264–72.

971. Smith N, Weymann A, Tausk FA, Gelfand JM. Complementary and alternative medicine for psoriasis: A qualitative review of the clinical trial literature. J. Am. Acad. Dermatol. . N. Smith, Department of Dermatology, University of Rochester, Rochester, NY, United States; 2009;61:841–56.

972. Souza RC, Lima HC, Bottmann P, Monteiro R. Cost analysis of system therapy for psoriasis in Brazil. J. Am. Acad. Dermatol. . R.C. Souza, Faculdades Evangelica de Curitiba, Curitiba, Brazil; 2009;60:AB166.

973. Stahl E, Raychaudhuri S, Chen R, Coblyn J, Shadick N, Weinblatt ME, et al. Meta-analysis of six genome-wide association studies in >25,000 case-control samples identifies new rheumatoid arthritis risk loci. Arthritis Rheum. . E. Stahl, Brigham and Women’s Hospital, Boston, United States; 2009;60:1894.

974. Turner D, Picot J, Cooper K, Loveman E. Adalimumab for the treatment of psoriasis. Health Technol. Assess. . D. Turner, Southampton Health Technology Assessments Centre, Wessex Institute for Health Research and Development, University of Southampton, Southampton, UK.; 2009;13 Suppl 2:49–54.

975. Yu X, Wieczorek S, Franke A, Yin H, Pierer M, Sina C, et al. Association of UCP2 -866 G/A polymorphism with chronic inflammatory diseases. Genes Immun. . S.M. Ibrahim, Section of Immunogenetics, University of Rostock, Rostock, Germany; 2009;10:601–5.

976. Zhang Z, Schmitt J, Wozel G, Kirch W. Treatment of plaque psoriasis with biologics. A meta-analysis of randomized controlled trials. Med. Klin. . Z. Zhang, Institut für Klinische Pharmakologie, Medizinische Fakultät Carl Gustav Carus, Technische Universität Dresden, 01307 Dresden, Germany; 2009;104:125–36.

977. Zivkovich AH, Feldman SR. Are ointments better than other vehicles for corticosteroid treatment of psoriasis? J. Drugs Dermatology . S.R. Feldman, Department of Dermatology, Wake Forest University School of Medicine, Winston-Salem, NC 27157-1071, United States; 2009;8:570–2.

978. Abstracts Presented at the 12th Joint Meeting of the International Society of Dermatopathology. Am. J. Dermatopathol. . 2009;31.

**2008**

979. Alamanos Y, Voulgari P V, Drosos AA. Incidence and prevalence of psoriatic arthritis: A systematic review. J. Rheumatol. . A. A. Drosos, Department of Internal Medicine, Medical School, University of Ioannina, 45110 Ioannina, Greece; 2008;35:1354–8.

980. Brimhall AK, King LN, Licciardone JC, Jacobe H, Menter A. Safety and efficacy of alefacept, efalizumab, etanercept and infliximab in treating moderate to severe plaque psoriasis: A meta-analysis of randomized controlled trials. Br. J. Dermatol. . A. Menter, Department of Dermatology, University of Texas Southwestern Medical Center, Dallas, TX, United States; 2008;159:274–85.

981. Buell C, Koo J. Long-term safety of mycophenolate mofetil and cyclosporine: a review. J. Drugs Dermatol. . C. Buell, University of California-Los Angeles, Los Angeles, CA 90025, USA.; 2008;7:741–8.

982. Davis Jr. JC, Mease PJ. Insights Into the Pathology and Treatment of Spondyloarthritis: From the Bench to the Clinic. Semin. Arthritis Rheum. . P.J. Mease, Seattle Rheumatology Associates, Rheumatology Clinical Research, Swedish Medical Center, Seattle, WA, United States; 2008;38:83–100.

983. Falagas ME, Vouloumanou EK, Matthaiou DK, Kapaskelis AM, Karageorgopoulos DE. Effectiveness and safety of short-course vs long-course antibiotic therapy for group A β-hemolytic streptococcal tonsillopharyngitis: A meta-analysis of randomized trials. Mayo Clin. Proc. . M. E. Falagas, Alfa Institute of Biomedical Sciences, 151 23 Marousi, Athens, Greece; 2008;83:880–9.

984. Haider AS, Lowes MA, Suárez-Fariñas M, Zaba LC, Cardinale I, Khatcherian A, et al. Identification of cellular pathways of “type 1,” Th17 T cells, and TNF- and inducible nitric oxide synthase-producing dendritic cells in autoimmune inflammation through pharmacogenomic study of cyclosporine A in psoriasis. J. Immunol 2008;180:1913–20.

985. Li Y, Chang M, Schrodi SJ, Callis-Duffin KP, Matsunami N, Civello D, et al. The 5q31 variants associated with psoriasis and Crohn’s disease are distinct. Hum. Mol. Genet. . Y. Li, Celera, Alameda, CA 94502, United States; 2008;17:2978–85.

986. Morton CA, McKenna KE, Rhodes LE. Guidelines for topical photodynamic therapy: Update. Br. J. Dermatol. . C. A. Morton, Department of Dermatology, Stirling Royal Infirmary, Stirling FK2 8AU, United Kingdom; 2008;159:1245–66.

987. Ortonne J-P. What’s new in dermatological treatments? Ann. Dermatol. Venereol.2008;135:S360–70.

988. Reich K, Sinclair R, Roberts G, Griffiths CEM, Tabberer M, Barker J. Comparative effects of biological therapies on the severity of skin symptoms and health-related quality of life in patients with plaque-type psoriasis: A meta-analysis. Curr. Med. Res. Opin 2008;24:1237–54.

989. Rich P, Griffiths CEM, Reich K, Nestle FO, Scher RK, Li S, et al. Baseline nail disease in patients with moderate to severe psoriasis and response to treatment with infliximab during 1 year. J. Am. Acad. Dermatol. . P. Rich, Oregon Health Sciences University, Portland, OR, United States; 2008;58:224–31.

990. Schmitt J, Zhang Z, Wozel G, Meurer M, Kirch W. Efficacy and tolerability of biologic and nonbiologic systemic treatments for moderate-to-severe psoriasis: Meta-analysis of randomized controlled trials. Br. J. Dermatol. . J. Schmitt, Department of Dermatology, Medical Faculty Carl Gustav Carus, Technische Universität Dresden, D-01307 Dresden, Germany; 2008;159:513–26.

991. Stewart LA, Rydzewska LHM, Keogh GF, Knight RSG. Systematic review of therapeutic interventions in human prion disease. Neurology . L. A. Stewart, Centre for Reviews and Dissemination, University of York, York YO10 5DD, United Kingdom; 2008;70:1272–81.

992. Thiers BH. What’s new in dermatologic therapy. Dermatol. Ther. . B. H. Thiers, Department of Dermatology, Medical University of SC, Charleston, SC 29425, United States; 2008;21:142–9.

993. Boron. J. Diet. Suppl. . 2008;5:62–94.

**2007**

994. Bessell A, Moss TP. Evaluating the effectiveness of psychosocial interventions for individuals with visible differences: A systematic review of the empirical literature. Body Image . A. Bessell, Centre for Appearance Research, Faculty of Applied Sciences, University of West of England, Bristol, BS16 1QY, United Kingdom; 2007;4:227–38.

995. Canter PH, Wider B, Ernst E. The antioxidant vitamins A, C, E and selenium in the treatment of arthritis: A systematic review of randomized clinical trials. Rheumatology . P.H. Canter, Department of Complementary Medicine, Peninsula Medical School, Universities of Exeter and Plymouth, Exeter EX2 4NT, United Kingdom; 2007;46:1223–33.

996. Chan KHN. Optimising the management of psoriasis with biologics. Hong Kong J. Dermatology Venereol. . 2007;15:88.

997. D’Haens G. Risk and benefits of biologic therapy for inflammatory bowel diseases. Gut . G. D’Haens, Imelda GI Clinical Research Centre, 2820 Bonheiden, Belgium; 2007;56:725–32.

998. De Argila D, Rodríguez-Nevado I, Chaves A. Cost-effectiveness analysis comparing methotrexate with puva therapy for moderate-severe psoriasis in the sanitary area of Badajoz. Actas Dermosifiliogr. . D. De Argila, Unidad de Dermatología, Hospital Infanta Cristina, 06080 Badajoz; 2007;98:35–41.

999. Diamond GA, Bax L, Kaul S. Uncertain effects of rosiglitazone on the risk for myocardial infarction and cardiovascular death. Ann. Intern. Med. . S. Kaul, Cedars-Sinai Medical Center, Los Angeles, CA 90048, United States; 2007;147:578–81.

1000. Eggebeen AT. Gout: An update. Am. Fam. Physician . A.T. Eggebeen, University of Pittsburgh, Arthritis Institute, S700 Biomedical Science Tower, Pittsburgh, PA 15261, United States; 2007;76:801–12.

1001. Furst DE, Breedveld FC, Kalden JR, Smolen JS, Burmester GR, Sieper J, et al. Updated consensus statement on biological agents for the treatment of rheumatic diseases, 2007. Ann. Rheum. Dis. . D.E. Furst, David Geffen School of Medicine, UCLA, Los Angeles, CA 90025, United States; 2007;66:iii2–22.

1002. Garduno J, Bhosle MJ, Balkrishnan R, Feldman SR. Measures used in specifying psoriasis lesion(s), global disease and quality of life: A systematic review. J. Dermatolog. Treat. . S.R. Feldman, Wake Forest University School of Medicine, Department of Dermatology, Winston-Salem, NC 27157-1071, United States; 2007;18:223–42.

1003. Khan S, Justice E, Jobanputra P. Adverse reactions and safety of newer disease-modifying antirheumatic drugs (DMARDs) for rheumatoid arthritis. Adverse Drug React. Bull. . P. Jobanputra, Department of Rheumatology, Selly Oak Hospital, University Hospital NHS Trust, Birmingham B29 6JD, United Kingdom; 2007;927–30.

1004. Lee YH, Rho YH, Choi SJ, Ji JD, Song GG, Nath SK, et al. The PTPN22 C1858T functional polymorphism and autoimmune diseases - A meta-analysis. Rheumatology . Y.H. Lee, Division of Rheumatology, Department of Internal Medicine, Korea University Medical Center, Seoul 136-705, South Korea; 2007;46:49–56.

1005. Leon A, Nguyen A, Letsinger J, Koo J. An attempt to formulate an evidence-based strategy in the management of moderate-to-severe psoriasis: A review of the efficacy and safety of biologics and prebiologic options. Expert Opin. Pharmacother. . J. Koo, University of California, Department of Dermatology, San Francisco, CA, United States; 2007;8:617–32.

1006. Li C, Wang G, Gao Y, Liu L, Gao T. TNF-α gene promoter -238G>A and -308G>A polymorphisms alter risk of psoriasis vulgaris: A meta-analysis. J. Invest. Dermatol. . T. Gao, Xian, Shaanxi, 710032, China; 2007;127:1886–92.

1007. Lichtenstein GR. General safety concerns associated with biologic therapies. Gastroenterol. Hepatol. . G.R. Lichtenstein, Department of Medicine, University of Pennsylvania, School of Medicine, Philadelphia, PA, United States; 2007;3:4–6.

1008. Manriquez JJ, Villouta MF, Williams HC. Evidence-based dermatology: Number needed to treat and its relation to other risk measures. J. Am. Acad. Dermatol. . J.J. Manriquez, Units of Dermatology,; 2007;56:664–71.

1009. Mariette X. Anticytokines (anti-TNF) and infection risk. Rev. du Prat. . X. Mariette, Service de Rhumatologie, Hôpital Bicêtre, Groupe Recherche Axée Sur la Tolérance des Biothérapies (RATIO), 94275 Le Kremlin-Bicêtre; 2007;57:1681–2.

1010. Nast A, Kopp IB, Augustin M, Banditt K-B, Boehncke W-H, Follmann M, et al. Evidence-based (S3) guidelines for the treatment of psoriasis vulgaris. JDDG - J. Ger. Soc. Dermatology . A. Nast, Division of Evidence Based Medicine (dEBM), Klinik für Dermatologie, Venerologie und Allergologie, Charité - Universitätsmedizin Berlin, Germany; 2007;5:1–119.

1011. Philipp S, Morcinietz CS, Wallace L, Sterry W. Biologics for the treatment of psoriasis: A systematic review. G. Ital. di Dermatologia e Venereol. . S. Philipp, Interdisciplinary Group of Molecular Immunopathology, Department of Dermatology and Allergy, Universitätsmedizin Berlin, 10117 Berlin, Germany; 2007;142:567–91.

1012. Reich A, Szepietowski J. Etanercept significantly improves quality of life of patients with psoriasis: Current literature review. Dermatologia Klin. . A. Reich, Katedra I Klinika Dermatologii, Wenerologii I Alergologii AM, 50-368 Wrocław; 2007;9:189–91.

1013. Ruxton CHS, Reed SC, Simpson JA, Millington KJ. The health benefits of omega-3 polyunsaturated fatty acids: A review of the evidence. J. Hum. Nutr. Diet. . C. Ruxton, Accredited Sports Dietitian and Registered Public Health Nutritionist, Nutrition Communications, Cupar KY15 4EA, United Kingdom; 2007;20:275–85.

1014. Steptoe A, Hamer M, Chida Y. The effects of acute psychological stress on circulating inflammatory factors in humans: A review and meta-analysis. Brain. Behav. Immun. . A. Steptoe, Psychobiology Group, Department of Epidemiology and Public Health, University College London, London, WC1 6BT, United Kingdom; 2007;21:901–12.

1015. Thompson Coon J, Pittler MH, Ernst E. Trifolium pratense isoflavones in the treatment of menopausal hot flushes: A systematic review and meta-analysis. Phytomedicine . M.H. Pittler, Complementary Medicine, Peninsula Medical School, Universities of Exeter and Plymouth, Exeter, EX2 4NT, United Kingdom; 2007;14:153–9.

1016. Tilg H, Feichtenschlager T, Knoflach P, Petritsch W, Schöfl R, Vogelsang H, et al. Use of infliximab in ulcerative colitis. Z. Gastroenterol. . H. Tilg, Abteilung für Innere Medizin, Krankenhaus Hall i. T., Akademisches Lehrkrankenhaus der Universität Innsbruck, 6060 Hall i. T.; 2007;45:907–11.

1017. Ulbricht C, Armstrong J, Basch E, Basch S, Bent S, Dacey C, et al. An evidence-based systematic review of aloe vera by the natural standard research collaboration. J. Herb. Pharmacother. . C. Ulbricht, Massachusetts General Hospital, Boston, MA, United States; 2007;7:279–323.

1018. Vena GA, Cassano N. Anti-tumor necrosis factor therapies for psoriasis. Expert Rev. Dermatol. . G.A. Vena, University of Bari, Departamenti di Policlinico, Department of 2nd Dermatology Clinic, 70124 Bari, Italy; 2007;2:335–49.

1019. Xu L-M, Ru X-Y, Wu T-X. Meta-analysis of the relationship between IFN-γ and psoriasis. Chinese J. Evidence-Based Med. . T.-X. Wu, Evidence-Based Medicine and Clinical Epidemiology Centre, West China Hospital, Sichuan University, Chengdu 610041, China; 2007;7:516–22.

1020. Yan K-L, Huang W, Zhang X-J, Yang S, Chen Y-M, Xiao F-L, et al. Follow-up analysis of PSORS9 in 151 Chinese families confirmed the linkage to 4q31-32 and refined the evidence to the families of early-onset psoriasis. J. Invest. Dermatol. . X.-J. Zhang, Institute of Dermatology, Anhui Medical University, Hefei, Anhui 230032, China; 2007;127:312–8.

**2006**

1021. Adams AK, Warshaw EM. Allergic contact dermatitis from mercapto compounds. Dermatitis . E.M. Warshaw, Dermatology Department 111K, Veterans Affairs Medical Center, Minneapolis, MN 55417, United States; 2006;17:56–70.

1022. Aubin F, Laurent R. Human papillomavirus-associated cutaneous lesions. Rev. du Prat. . Service de Dermatologie, EA 3181, Université de Franche-Comté, 25030 Besançon; 2006;56:1905–11.

1023. Aziz R, Lorberg B, Tampi RR. Treatments for late-life bipolar disorder. Am. J. Geriatr. Pharmacother. . R.R. Tampi, Department of Psychiatry, Yale University School of Medicine, New Haven, CT, United States; 2006;4:347–64.

1024. Bayés M, Rabasseda X, Prous JR. Gateways to clinical trials: November 2006. Methods Find. Exp. Clin. Pharmacol. . M. Bayés, Prous Science, S.A., 08080 Barcelona, Spain; 2006;28:657–78.

1025. Bayés M, Rabasseda X, Prous JR. Gateways to Clinical Trials. Methods Find. Exp. Clin. Pharmacol. . M. Bayés, Prous Science, S.A., 08080 Barcelona, Spain; 2006;28:31–63.

1026. Boehncke W-H, Prinz J, Gottlieb AB. Biologic therapies for psoriasis. A systematic review. J. Rheumatol. . W.-H. Boehncke, Department of Dermatology, Johann Wolfgang Goethe University, D-60594 Frankfurt am Main, Germany; 2006;33:1447–51.

1027. Carey W, Glazer S, Gottlieb AB, Lebwohl M, Leonardi C, Menter A, et al. Relapse, rebound, and psoriasis adverse events: An advisory group report. J. Am. Acad. Dermatol. . W. Carey, Royal Victoria Hospital, Department of Dermatology, Montreal, Que. H3A 1A1, Canada; 2006;54:S171–81.

1028. Cassell S, Kavanaugh AF. Therapies for psoriatic nail disease. A systematic review. J. Rheumatol. . A.F. Kavanaugh, Division of Rheumatology, Allergy, and Immunology, University of California at San Diego, San Diego, CA 92093-0943, United States; 2006;33:1452–6.

1029. Chen S-L, Yan J, Liu Y, Xu X-L, Sun J-F, Wu T-X. Efalizumab for psoriasis: A systematic review. Chinese J. Evidence-Based Med. . J.-F. Sun, Institute of Dermatology, Chinese Academy of Medical Sciences, Peking Union Medical College, Nanjing 210042, China; 2006;6:267–72.

1030. Furst DE, Breedveld FC, Kalden JR, Smolen JS, Burmester GR, Emery P, et al. Updated consensus statement on biological agents for the treatment of rheumatic diseases, 2006. Ann. Rheum. Dis. . D.E. Furst, David Geffen School of Medicine, UCLA, Los Angeles, CA 90025, United States; 2006;65:iii2–15.

1031. Haase I. Response to Clark and Kupper [1]. J. Clin. Invest. . I. Haase, Department of Dermatology, Center for Molecular Medicine, University of Cologne (CMMC), 50924 Cologne, Germany; 2006;116:3088.

1032. Hankey GJ. Is plasma homocysteine a modifiable risk factor for stroke? Nat. Clin. Pract. Neurol. . G. Hankey, Department of Neurology, Royal Perth Hospital, Perth, WA 6001, Australia; 2006;2:26–33.

1033. Hawkins T. Appearance-related side effects of HIV-1 treatment. AIDS Patient Care STDS . T. Hawkins, Southwest CARE Center, Santa Fe, NM 87505, United States; 2006;20:6–18.

1034. Helliwell PS. Therapies for dactylitis in psoriatic arthritis. A systematic review. J. Rheumatol. . P.S. Helliwell, Academic Unit of Musculoskeletal Medicine, University of Leeds, Leeds LS2 9NZ, United Kingdom; 2006;33:1439–41.

1035. Herman SM, Shin MH, Holbrook A, Rosenthal D. The role of antimalarials in the exacerbation of psoriasis: A systematic review. Am. J. Clin. Dermatol. . S.M. Herman, Health Sciences Centre, McMaster University, Hamilton, Ont. L8N 3Z5, Canada; 2006;7:249–57.

1036. Kavanaugh AF, Ritchlin CT, Boehncke W-H, Cassell S, Gladman DD, Gottlieb AB, et al. Systematic review of treatments for psoriatic arthritis: An evidence based approach and basis for treatment guidelines. J. Rheumatol. . C.T. Ritchlin, Clinical Immunology Research Unit, University of Rochester Medical Center, Box 695, Rochester, NY 14642, United States; 2006;33:1417–21.

1037. Kemény L, Brodszky V, Kárpáti K, Gulácsi L. The role of biological drugs in the treatment of psoriasis, results from 9 randomized placebo-controlled trials. Orv. Hetil. . L. Kemény, Szeged 6720, Hungary; 2006;147:981–90.

1038. Kerns MJJ, Graves JE, Smith DI, Heffernan MP. Off-Label Uses of Biologic Agents in Dermatology: A 2006 Update. Semin. Cutan. Med. Surg. . M.P. Heffernan, Wright State University, Dayton, OH, United States; 2006;25:226–40.

1039. Lim L, Suhler EB, Smith JR. Biologic therapies for inflammatory eye disease. Clin. Exp. Ophthalmol. . J.R. Smith, Casey Eye Institute, Oregon Health and Science University, Portland, OR 97239, United States; 2006;34:365–74.

1040. Marsland a M, Chalmers RJG, Hollis S, Leonardi-Bee J, Griffiths CEM. Interventions for chronic palmoplantar pustulosis. Cochrane Database Syst. Rev. 2006;CD001433.

1041. Menter A, Leonardi CL, Sterry W, Bos JD, Papp KA. Long-term management of plaque psoriasis with continuous efalizumab therapy. J. Am. Acad. Dermatol. . A. Menter, Texas Dermatology Research Institute, Dallas, TX 75230, United States; 2006;54:S182–8.

1042. Nash P. Therapies for axial disease in psoriatic arthritis. A systematic review. J. Rheumatol. . P. Nash, Department of Medicine, University of Queensland, Cotton Tree, QLD, Australia; 2006;33:1431–4.

1043. Ritchlin C, Tausk F. A medical conundrum: Onset of psoriasis in patients receiving anti-tumour necrosis factor agents. Ann. Rheum. Dis. . C. Ritchlin, Clinical Immunology Research Center, Allergy, Immunology and Rheumatology Division, University of Rochester School of Medicine and Dentistry, Rochester, NY, United States; 2006;65:1541–4.

1044. Ruderman E. Adalimumab in psoriatic arthritis: A viewpoint by Eric Ruderman. Drugs . E. Ruderman, Division of Rheumatology, Northwestern University, Feinberg School of Medicine, Chicago, IL, United States; 2006;66:1497.

1045. Shear NH. Fulfilling an unmet need in psoriasis: Do biologicals hold the key to improved tolerability? Drug Saf. . N.H. Shear, Sunnybrook and Women’s College, Health Sciences Centre, M1-700, Toronto, Ont. M4N 3M5, Canada; 2006;29:49–66.

1046. Shigaki CL, Glass B, Schopp LH. Mindfulness-based stress reduction in medical settings. J. Clin. Psychol. Med. Settings . C.L. Shigaki, Department of Health Psychology, University of Missouri, Columbia, MO 65212, United States; 2006;13:209–16.

1047. Soriano ER, McHugh NJ. Therapies for peripheral joint disease in psoriatic arthritis. A systematic review. J. Rheumatol. . E.R. Soriano, Hospital Italiano de Buenos Aires, Capital Federal, Argentina; 2006;33:1422–30.

1048. Staunton DE, Lupher ML, Liddington R, Gallatin WM. Targeting Integrin Structure and Function in Disease . Adv. Immunol. D.E. Staunton, ICOS Corporation, Bothell, WA, United States; 2006. p. 111–57.

1049. Stern RS. Lymphoma risk in psoriasis: Results of the PUVA follow-up study. Arch. Dermatol. . R.S. Stern, Department of Dermatology, Beth Israel Deaconess Medical Center, Boston, MA 02215, United States; 2006;142:1132–5.

1050. Strober BE, Siu K, Menon K. Conventional systemic agents for psoriasis. A systematic review. J. Rheumatol. . B.E. Strober, New York University School of Medicine, TCH-158, New York, NY 10016, United States; 2006;33:1442–6.

1051. Tilg H, Kaser A, Moschen AR. How to modulate inflammatory cytokines in liver diseases. Liver Int. . H. Tilg, Department of Medicine, Division of Gastroenterology and Hepatology, Innsbruck Medical University, 6020 Innsbruck, Austria; 2006;26:1029–39.

1052. Wolkenstein P. Living with psoriasis. J. Eur. Acad. Dermatology Venereol. . P. Wolkenstein, Service de dermatologie, Hôpital Henri-Mondor, F-94010 Créteil, Cedex, France; 2006;20:28–32.

1053. Wollina U, Hansel G, Koch A, Abdel-Naser MB. Topical pimecrolimus for skin disease other than atopic dermatitis. Expert Opin. Pharmacother. . U. Wollina, Department of Dermatology, Academic Teaching Hospital Dresden-Friedrichstadt, Dresden, Germany; 2006;7:1967–75.

1054. Woolacott NF, Khadjesari ZCS, Bruce IN, Riemsma RP. Etanercept and infliximab for the treatment of psoriatic arthritis: A systematic review. Clin. Exp. Rheumatol. . N.F. Woolacott, Centre for Reviews and Dissemination, University of York, York YO10 5DD, United Kingdom; 2006;24:587–93.

1055. Woolacott N, Vergel YB, Hawkins N, Kainth A, Khadjesari Z, Misso K, et al. Etanercept and infliximab for the treatment of psoriatic arthritis: A systematic review and economic evaluation. Health Technol Assess 2006;10:iii – 78.

1056. Woolacott N, Hawkins N, Mason A, Kainth A, Khadjesari Z, Vergel YB, et al. Etanercept and efalizumab for the treatment of psoriasis: a systematic review. Health Technol. Assess. 2006.

1057. Young AH, Newham JI. Lithium in maintenance therapy for bipolar disorder. J. Psychopharmacol 2006;20:17–22.

1058. A betamethasone-calcipotriene combination for psoriasis. Med. Lett. Drugs Ther. . 2006;48:55–6.

**2005**

1059. Arnaud C, Veillard NR, Mach F. Cholesterol-independent effects of statins in inflammation, immunomodulation and atherosclerosis. Curr. Drug Targets - Cardiovasc. Haematol. Disord. . F. Mach, Division of Cardiology, Foundation for Medical Research, 1211 Geneva, Switzerland; 2005;5:127–34.

1060. Braddock M. Anti-arthritis therapies 2005. 23-24 February 2005, London, UK. IDrugs . M. Braddock, AstraZeneca R and D, Disease Science Section, Discovery Biosciences Division, Leicestershire LE11 5RH, United Kingdom; 2005;8:288–9.

1061. Brown LR. Commercial challenges of protein drug delivery. Expert Opin. Drug Deliv. . L.R. Brown, Epic Therapeutics, Inc., Baxter Healthcare Corporation, Norwood, MA 02062, United States; 2005;2:29–42.

1062. Buka RL, Cunningham BB. Connective tissue disease in children. Pediatr. Ann. . R.L. Buka, Childrens Hosp./Hlth. Ctr. San Diego, University of California San Diego, San Diego, CA 92123, United States; 2005;34:225–38.

1063. Cassano N, De Meo M, Scoppio BM, Loviglio MC, Del Vecchio S, Vena GA. Does oxidative stress play a role in the pathogenesis of urticarias? Eur. J. Inflamm. . G.A. Vena, Dept. Int. Med. Immunol./Infect. Dis, 2nd Unit of Dermatology, University of Bari, 70124 Bari, Italy; 2005;3:5–10.

1064. Cianci R, Cammarota G, Raducci F, Pandolfi F. The impact of biological agents interfering with receptor/ligand binding in the immune system. Eur. Rev. Med. Pharmacol. Sci. . R. Cianci, Institute of Internal Medicine, Catholic University, Rome, Italy; 2005;9:305–14.

1065. Dagenais NJ, Jamali F. Protective effects of angiotensin II interruption: Evidence for antiinflammatory actions. Pharmacotherapy . F. Jamali, 3118 Dentistry/Pharmacy Building, University of Alberta, Edmonton, Alta. T6G 2N8, Canada; 2005;25:1213–29.

1066. Davidson A, Diamond B, Wofsy D, Daikh D. Block and tackle: CTLA4Ig takes on lupus. Lupus . A. Davidson, New York, NY 10032, United States; 2005;14:197–203.

1067. Dominguez C, Tamayo N, Zhang D. p38 inhibitors: Beyond pyridinylimidazoles. Expert Opin. Ther. Pat. . C. Dominguez, Amgen Inc., Thousands Oaks, CA 91320, United States; 2005;15:801–16.

1068. Eklund JW, Kuzel TM. Denileukin diftitox: A concise clinical review. Expert Rev. Anticancer Ther. . T.M. Kuzel, R.H. Lurie Compreh. Cancer Center, Northwestern University, Chicago, IL 60611, United States; 2005;5:33–8.

1069. Fischer U, Schulze-Osthoff K. Pharmacological modulation of caspase activation. Curr. Med. Chem. Anti-inflamm. Anti-Allergy Agents . U. Fischer, Institute of Molecular Medicine, University of Düsseldorf, D-40225 Düsseldorf, Germany; 2005;4:407–19.

1070. Fisher VS. Clinical monograph for drug formulary review: systemic agents for psoriasis/psoriatic arthritis. J. Manag. Care Pharm. . V.S. Fisher, WellPoint Pharmacy Management, 5415 West Old Fort Dr., Spokane, WA 99208, USA.; 2005;11:33–55.

1071. Follmann M, Sterry W, Rzany B. Development of the evidence-based guidelines for psoriasis - A project of the German dermatological society (DDG). JDDG - J. Ger. Soc. Dermatology . B. Rzany, Klinik für Dermatologie, Charité - Universitätsmedizin Berlin, Campus Charité Mitte, D-10117 Berlin; 2005;3:678–89.

1072. Gordon KB, Bonish BK, Patel T, Leonardit CL, Nickoloff BJ. The tumour necrosis factor-α inhibitor adalimumab rapidly reverses the decrease in epidermal Langerhans cell density in psoriatic plaques. Br. J. Dermatol. . B.J. Nickoloff, Skin Cancer Research Program, Cardinal Bernardin Cancer Center, Oncology Institute, Maywood, IL 601 S3, United States; 2005;153:945–53.

1073. Gottlieb AB. Psoriasis: Emerging therapeutic strategies. Nat. Rev. Drug Discov. . A.B. Gottlieb, Clinical Research Center, UMDNJ-Robert Wood Johnson Med. Sch., New Brunswick, NJ 08901-0019, United States; 2005;4:19–34.

1074. Green C, Colquitt JL, Kirby J, Davidson P. Topical corticosteroids for atopic eczema: Clinical and cost effectiveness of once-daily vs. more frequent use. Br. J. Dermatol. . C. Green, Southampton Hlth. Technol. A. C., Wessex Inst. for Hlth. Res./Devmt., University of Southampton, Southampton SO16 7PX, United Kingdom; 2005;152:130–41.

1075. Hankin CS, Feldman SR, Szczotka A, Stinger RC, Fish L, Hankin DL. A cost comparison of treatments of moderate to severe psoriasis. Drug Benefit Trends . C.S. Hankin, BioMedEcon LLC, San Jose, CA, United States; 2005;17:200–14.

1076. Harries MJ, Chalmers RJG, Griffiths CEM. Fumaric acid esters for severe psoriasis: A retrospective review of 58 cases. Br. J. Dermatol. . M.J. Harries, Dermatology Centre, University of Manchester, Hope Hospital, Salford, Manchester, United Kingdom; 2005;153:549–51.

1077. Hrastinger A, Dietz B, Bauer R, Sagraves R, Mahady G. Is there clinical evidence supporting the use of botanical dietary supplements in children? J. Pediatr. . G. Mahady, UIC/NIH Ctr. Bot. Dietary S., College of Pharmacy, University of Illinois at Chicago, Chicago, IL 60612, United States; 2005;146:311–7.

1078. Huntley AL, Coon JT, Ernst E. The safety of herbal medicinal products derived from Echinacea species: A systematic review. Drug Saf. . A.L. Huntley, Peninsula Medical School, Complementary Medicine, Universities of Exeter and Plymouth, Exeter, EX2 4NT, United Kingdom; 2005;28:387–400.

1079. Jais PH. How frequent is altered gene expression among susceptibility genes to human complex disorders? Genet. Med. . P.H. Jais, CHIASMA, 75004 Paris, France; 2005;7:83–96.

1080. Kostovic K, Pasic A. New treatment modalities for vitiligo: Focus on topical immunomodulators. Drugs . K. Kostovic, Dept. of Dermatology and Venerology, Zagreb University Hospital Center, Zagreb, HR-10000, Croatia; 2005;65:447–59.

1081. Labro M-T. Biotechnology - Fourth Croatian International Scientific Conference. Biotechnology and immunomodulatory drugs. 20-23 February 2005, Zagreb, Croatia. IDrugs . M.-T. Labro, Faculte Xavier Bichat, Service d’Hematologie et d'Immunologie Biologiques, CHU Xavier Bichat, 75018 Paris, France; 2005;8:290–3.

1082. Lee CS, Koo J. A review of acitretin, a systemic retinoid for the treatment of psoriasis. Expert Opin. Pharmacother. . C.S. Lee, University of California, Davis Medical Center, Department of Dermatology, Sacramento, CA 95817, United States; 2005;6:1725–34.

1083. Lodén M. The clinical benefit of moisturizers. J. Eur. Acad. Dermatology Venereol. . M. Lodén, ACO HUD AB, Box 622, SE-194 26 Upplands Väsby, Sweden; 2005;19:672–88.

1084. Malhotra S, Bansal D, Shafiq N, Pandhi P, Kumar B. Potential therapeutic role of peroxisome proliferator activated receptor-γ agonists in psoriasis. Expert Opin. Pharmacother. . S. Malhotra, PGIMER, Department of Pharmacology, Chandigarh 160012, India; 2005;6:1455–61.

1085. Mariwalla K, Rohrer TE. Use of lasers and light-based therapies for treatment of acne vulgaris. Lasers Surg. Med. . T.E. Rohrer, SkinCare Physicians of Chestnut Hill, Chestnut Hill, MA 02467, United States; 2005;37:333–42.

1086. Mealy NE, Bayés M. Infliximab. Drugs Future . N.E. Mealy, Prous Science, 08080 Barcelona, Spain; 2005;30:845–6.

1087. Mease P, Goffe BS. Diagnosis and treatment of psoriatic arthritis. J. Am. Acad. Dermatol. . Seattle Rheumatology Associates, Swedish Hospital Medical Center, Division of Clinical Research,; 2005;52:1–19.

1088. Nasermoaddeli A, Kagamimori S. Balneotherapy in medicine: A review. Environ. Health Prev. Med. . A. Nasermoaddeli, Department of Welfare Promotion and Epidemiology, Faculty of Medicine, Toyama Medical and Pharmaceutical University, Toyama 930-0194, Japan; 2005;10:171–9.

1089. Parchamazad P, Ghazvini P, Honeywell M, Treadwell P. Abatacept (CTLA4-Ig, Orencia): An investigational biological compound for the treatment of rheumatoid arthritis. P T . P. Parchamazad, Center of Excellence at Florida, A and M University’s College of Pharmacy, Tallahassee, FL, United States; 2005;30:633–8+643+669.

1090. Ponticelli C. Cyclosporine: From renal transplantation to autoimmune diseases . Ann. N. Y. Acad. Sci. C. Ponticelli, 20131 Milan, Italy; 2005. p. 551–8.

1091. Ranieri G, Ria R, Roccaro AM, Vacca A, Ribatti D. Development of vasculature targeting strategies for the treatment of chronic inflammatory diseases. Curr. Drug Targets Inflamm. Allergy . D. Ribatti, Dept. of Human Anatomy and Histology, Policlinico, I-70124 Bari, Italy; 2005;4:13–22.

1092. Sains K. Clinical nutrition - Using nutraceuticals in critical care. Hosp. Pharm. . K. Sains, Essex Rivers Healthcare NHS Trust, Dept. of Health and Human Sciences, University of Essex, Colchester, United Kingdom; 2005;12:14–6.

1093. Schön MP, Ludwig RJ. Lymphocyte trafficking to inflamed skin - Molecular mechanisms and implications for therapeutic target molecules. Expert Opin. Ther. Targets . M.P. Schön, Bayerische Julius-Maximilians University, DFG Research Center for Experimental Biomedicine, Department of Dermatology and Venerology, Würzburg, Germany; 2005;9:225–43.

1094. Strieter RM, Belperio JA, Burdick MD, Keane MP. CXC chemokines in angiogenesis relevant to chronic fibroproliferation. Curr. Drug Targets Inflamm. Allergy . R.M. Strieter, Div. of Pulmonary Critical Care Med., Department of Medicine, UCLA School of Medicine, Los Angeles, CA 90024-1922, United States; 2005;4:23–6.

1095. Ulbricht C, Basch E, Barrette E-P, Bent S, Boon H, Hammerness PG, et al. Shark cartilage: An evidence-based systematic review for the natural standard research collaboration. J. Cancer Integr. Med. . C. Ulbricht, Natural Standard, Cambridge, MA 02142, United States; 2005;3:99–111.

1096. Van De Kerkhof PCM, Wasel N, Kragballe K, Cambazard F, Murray S. A two-compound product containing calcipotriol and betamethasone dipropionate provides rapid, effective treatment of psoriasis vulgaris regardless of baseline disease severity. Dermatology . P.C.M. Van De Kerkhof, University Hospital Nijmegen, NL-6500 HB Nijmegen, Netherlands; 2005;210:294–9.

1097. Wijdenes J. The new wave in mAbs therapeutics: Putting high expectations in perspective. EBR - Eur. Biopharm. Rev. . Diaclone SAS, France; 2005;50–5.

1098. Zhou X, Schmidtke P, Zepp F, Meyer CU. Boosting interleukin-10 production: Therapeutic effects and mechanisms. Curr. Drug Targets Immune, Endocr. Metab. Disord. . X. Zhou, Department of Paediatric Immunology and Infectious Diseases, University Hospital Mainz, D-55131 Mainz, Germany; 2005;5:465–75.

1099. Herbal medicine. Focus Altern. Complement. Ther. . 2005;10:222–41.

**2004**

1100. Bayés M, Rabasseda X, Prous JR. Gateways to Clinical Trials. Methods Find. Exp. Clin. Pharmacol. . M. Bayés, Prous Science, S.A., 08080 Barcelona, Spain; 2004;26:723–53.

1101. Cohen MD. Raising expectations for arthritis treatment: Biologic response modifiers are making remission possible. Postgrad. Med. . M.D. Cohen, Division of Rheumatology, Mayo Clinic, Jacksonville, FL 32224, United States; 2004;116:41–50.

1102. Dawson JK, Clewes AR, Hendry J. Pulmonary effects of low-dose methotrexate therapy. Clin. Pulm. Med. . J.K. Dawson, Department of Rheumatology, St. Helens and Knowsley NHS Trust, St. Helens, Merseyside, WA9 3DA, United Kingdom; 2004;11:307–17.

1103. Fenton C, Plosker GL. Calcipotriol/betamethasone dipropionate: A review of its use in the treatment of psoriasis vulgaris. Am. J. Clin. Dermatol. . C. Fenton, Adis International Inc., Yardley, PA 19067, United States; 2004;5:463–78.

1104. Gambichler T, Boms S, Freitag M. Contact dermatitis and other skin conditions in instrumental musicians. BMC Dermatol. . T. Gambichler, Dermatology Out-Patient Clinic, Oldchurch Hospital, London RM7 0BE, United Kingdom; 2004;4.

1105. Gupta AK, Skinner AR. A review of the use of infliximab to manage cutaneous dermatoses. J. Cutan. Med. Surg. . A.K. Gupta, London, Ont. N6K 1L6, Canada; 2004;8:77–89.

1106. Ho VC. The use of ciclosporin in psoriasis: A clinical review. Br. J. Dermatology, Suppl. . V.C. Ho, Division of Dermatology, University of British Columbia, Vancouver, BC, Canada; 2004;150:1–10.

1107. Ibbotson SH, Bilsland D, Cox NH, Dawe RS, Diffey B, Edwards C, et al. An update and guidance on narrowband ultraviolet B phototherapy: A British Photodermatology Group Workshop Report. Br. J. Dermatol. . S.H. Ibbotson, Photobiology Unit, Ninewells Hosp. and Medical School, Dundee DD1 9SY, United Kingdom; 2004;151:283–97.

1108. Mease PJ. Targeting therapy in psoriatic arthritis. Drug Discov. Today Ther. Strateg. . P.J. Mease, Seattle Rheumatology Associates, Seattle, WA 98104, United States; 2004;1:389–96.

1109. Menter A, Kosinski M, Bresnahan BW, Papp KA, Ware Jr. JE. Impact of efalizumab on psoriasis-specific patient-reported outcomes. Results from three randomized, placebo-controlled clinical trials of moderate to severe plaque psoriasis. J. Drugs Dermatol. . A. Menter, Baylor Medical Center, Dallas, Texas, USA.; 2004;3:27–38.

1110. Mitchell PB. Australian and New Zealand clinical practice guidelines for the treatment of bipolar disorder. Aust. N. Z. J. Psychiatry . P.B. Mitchell, School of Psychiatry, University of New South Wales, Prince of Wales Hospital, Randwick, NSW 2031, Australia; 2004;38:280–305.

1111. Ohmori K, Hasegawa K, Tamura T, Miyake K, Matsubara M, Masaki S, et al. Properties of olopatadine hydrochloride, a new antiallergic/antihistaminic drug. Arzneimittel-Forschung/Drug Res. . K. Ohmori, Pharmaceutical Research Institute, Kyowa Hakko Kogyo Co., Ltd., Suntogun, Shizuoka 411-8731, Japan; 2004;54:809–29.

1112. Russo PAJ, Ilchef R, Cooper A. Psychiatric morbidity in psoriasis: A review. Australas. J. Dermatol. . R. Ilchef, Consultation-Liaison Psychiatry, Royal North Shore Hospital, St Leonards, NSW 2065, Australia; 2004;45:155–60.

1113. Sagoo GS, Tazi-Ahnini R, Barker JWN, Elder JT, Nair RP, Samuelsson L, et al. Meta-analysis of genome-wide studies of psoriasis susceptibility reveals linkage to chromosomes 6p21 and 4q28-q31 in Caucasian and Chinese Hans population. J. Invest. Dermatol. . G.S. Sagoo, Division of Genomic Medicine, University of Sheffield, Royal Hallamshire Hospital, Sheffield S10 2RX, United Kingdom; 2004;122:1401–5.

1114. Sculpher MJ, Pang FS, Manca A, Drummond MF, Golder S, Urdahl H, et al. Generalisability in economic evaluation studies in healthcare: A review and case studies. Health Technol. Assess. (Rockv). . M.J. Sculpher, Centre for Health Economics, University of York, York, United Kingdom; 2004;8:iii – 117.

1115. Shiina T, Inoko H, Kulski JK. An update of the HLA genomic region, locus information and disease associations: 2004. Tissue Antigens . H. Inoko, Dept. of Basic Med. Sci./Molec. Med., Division of Molecular Life Science, Tokai University School of Medicine, Isehara 259-1143, Japan; 2004;64:631–49.

1116. Spuls PI, Tuut MK, Van Everdingen JJE, De Rie MA. The practice guideline “photo(chemo)therapy and systemic therapy in severe chronic plaque-psoriasis.” Ned. Tijdschr. Geneeskd. . J.J.E. Van Everdingen, Kwaliteitsinst. Gezondheidszorg CBO, Medisch-Specialistische Kwaliteit, 3502 LB Utrecht, Netherlands; 2004;148:2121–5.

1117. Stebbins WG, Lebwohl MG. Biologics in combination with nonbiologics: Efficacy and safety. Dermatol. Ther. . M.G. Lebwohl, Department of Dermatology, Mount Sinai School of Medicine, Box 1048, New York, NY 10029, United States; 2004;17:432–40.

1118. Whysner J, Reddy M V, Ross PM, Mohan M, Lax EA. Genotoxicity of benzene and its metabolites. Mutat. Res. - Rev. Mutat. Res. . J. Whysner, Division of Pathology and Toxicology, American Heath Foundation, Valhalla, NY 10595, United States; 2004;566:99–130.

**2003**

1119. Tamibarotene: AM 80, Retinobenzoic acid, Tamibaro. Drugs R D . 2004;5:359–62. Available from: http://www.embase.com/search/results?subaction=viewrecord&from=export&id=L396185001120. Bruner CR, Feldman SR, Ventrapragada M, Fleischer Jr. AB. A systematic review of adverse effects associated with topical treatments for psoriasis. Dermatol. Online J. . C.R. Bruner, Department of Dermatology, Wake Forest Univ. School of Medicine, Winston-Salem, NC, United States; 2003;9:31–4.

1121. Dawe RS. A quantitative review of studies comparing the efficacy of narrow-band and broad-band ultraviolet B for psoriasis [11]. Br. J. Dermatol. . R.S. Dawe, Department of Dermatology, University of Dundee, Ninewells Hosp. and Medical School, Dundee DD1 9SY, United Kingdom; 2003;149:669–72.

1122. Goustas P, Cork MJ, Higson D. EumovateTM (clobetasone butyrate 0.05%) cream: A review of clinical efficacy and safety. J. Dermatolog. Treat. . P. Goustas, GlaxoSmithKline, Brentford, Middlesex TW8 9GS, United Kingdom; 2003;14:71–85.

1123. Gupta AK, Ryder JE. Photodynamic Therapy and Topical Aminolevulinic Acid: An Overview. Am. J. Clin. Dermatol. . A.K. Gupta, London, Ont. N6K 1L6, Canada; 2003;4:699–708.

1124. Pearson MM, Rogers PD, Cleary JD, Chapman SW, Da Camara C, Perreault MM. Voriconazole: A new triazole antifungal agent. Ann. Pharmacother. . M.M. Pearson, Department of Pharmacy Practice, School of Pharmacy, University of Mississippi, Jackson, MS 39216-4505, United States; 2003;37:420–32.

1125. Ruderman EM. Evaluation and management of psoriatic arthritis: The role of biologic therapy. J. Am. Acad. Dermatol. . E.M. Ruderman, NW. Univ. Feinberg Sch. of Medicine, Division of Rheumatology, Chicago, IL 60611, United States; 2003;49:S125–32.

1126. Yaqoob P. Lipids and the immune response: From molecular mechanisms to clinical applications. Curr. Opin. Clin. Nutr. Metab. Care . P. Yaqoob, Hugh Sinclair Unit of Human Nutrition, School of Food Biosciences, University of Reading, Reading RG6 6AP, United Kingdom; 2003;6:133–50.

1127. Drug and Non-Drug Treatment Strategies to Assist Smoking Cessation. Therapie . 2003;58:479–97.

**2002**

1128. Brockbank J, Gladman D. Diagnosis and management of psoriatic arthritis. Drugs . D. Gladman, Ctr. Prognosis Studs. Rheum. Dis., Toronto Western Hospital, ECW 5-034B, Toronto, Ont. M5T 2S8, Canada; 2002;62:2447–57.

1129. Goetz CG, Koller WC, Poewe W, Rascol O, Sampaio C, Brin MF, et al. MAO-B inhibitors for the treatment of Parkinson’s disease. Mov. Disord. . A.J. Lees, London N20 8WE, United Kingdom; 2002;17:S38–44.

1130. Heydendael VMR, Spuls PI, Ten Berge IJM, Opmeer BC, Bos JD, De Rie MA. Cyclosporin trough levels: Is monitoring necessary during short-term treatment in psoriasis? A systematic review and clinical data on trough levels. Br. J. Dermatol. . M.A. De Rie, Department of Dermatology, Academic Medical Center, University of Amsterdam, 1100 DD Amsterdam, Netherlands; 2002;147:122–9.

1131. Krueger GG. Clinical features of psoriatic arthritis. Am. J. Manag. Care . 2002;8:S160–70.

1132. Levin C, Maibach H. Exploration of “alternative” and “natural” drugs in dermatology. Arch. Dermatol. . H. Maibach, Department of Dermatology, UCSF Medical Center, San Francisco, CA 94143, United States; 2002;138:207–11.

1133. Mason J, Mason AR, Cork MJ. Topical preparations for the treatment of psoriasis: A systematic review. Br. J. Dermatol. . J. Mason, Centre for Health Services Research, University of Newcastle Upon Tyne, Newcastle upon Tyne NE2 4AA, United Kingdom; 2002;146:351–64. A

1134. Sorbera LA, Leeson PA, Revel L, Bayés M. Siplizumab: Antipsoriatic treatment of transplant rejection. Drugs Future . L.A. Sorbera, Prous Science, 08080 Barcelona, Spain; 2002;27:558–62.

**2001**

1135. Chalmers RJG, O’Sullivan T, Owen CM, Griffiths CEM. A systematic review of treatments for guttate psoriasis. Br. J. Dermatol. . C.M. Owen, Dermatology Centre, University of Manchester, Hope Hospital, Salford, Manchester M6 8HD, United Kingdom; 2001;145:891–4.

1136. Darvay A. Phototherapy for psoriasis. C. Bull. Dermatology . A. Darvay, Kinghorn Dermatology Unit, Royal United Hospital Bath, NHS Trust, Bath BA1 3NG, United Kingdom; 2001;3:9–14.

1137. Faerber L, Braeutigam M, Weidinger G, Mrowietz U, Christophers E, Schulze HJ, et al. Cyclosporine in severe psoriasis: Results of a meta-analysis in 579 patients. Am. J. Clin. Dermatol. . M. Braeutigam, Novartis Pharma GmbH, Clinical Research Department, 90429 Nuremberg, Germany; 2001;2:41–7.

1138. Kuwano M, Fukushi J-I, Okamoto M, Nishie A, Goto H, Ishibashi T, et al. Angiogenesis factors. Intern. Med. . M. Kuwano, Department of Medical Biochemistry, Graduate School of Medical Science, Kyushu University, Higashi-ku, Fukuoka 812-8582, Japan; 2001;40:565–72.

1139. Owen CM, Chalmers RJG, O’Sullivan T, Griffiths CEM. A systematic review of antistreptococcal interventions for guttate and chronic plaque psoriasis. Br. J. Dermatol. . C.M. Owen, Dermatology Centre, University of Manchester, Hope Hospital, Salford, Manchester M6 8HD, United Kingdom; 2001;145:886–90.

1140. Paleolog E. Anti-TNFα in the treatment of inflammatory diseases. Cent. J. Immunol. . E. Paleolog, Endothelial Cell Biology Group, K. Inst. Rheumatol. Div. Fac. Med., Imperial. College of Sci. Technol., Hammersmith, London W6 8LH, United Kingdom; 2001;26:140–8.

1141. Rodewald EJ, Housman TS, Mellen BG, Feldman SR. The efficacy of 308nm laser treatment of psoriasis compared to historical controls. Dermatol. Online J. . E.J. Rodewald, Department of Dermatology,Wake Forest University School of Medicine, Winston-Salem, North Carolina, USA.; 2001;7:4.

1142. Vander Zanden JA, Valuck RJ, Bunch CL, Perlman JI, Anderson C, Wortman GI. Systemic adverse effects of ophthalmic β-blockers. Ann. Pharmacother. . R.J. Valuck, Department of Pharmacy Practice, School of Pharmacy, UCHSC, Denver, CO 80262-0238, United States; 2001;35:1633–7.

**2000**

1143. Alarcón GS. Methotrexate use in rheumatoid arthritis. A clinician’s perspective. Immunopharmacology . G.S. AlarconAlarcon, Div. Clinical Immunology/Rheumatol., University of Alabama at Birmingham, Birmingham, AL 35294, United States; 2000;47:259–71.

1144. Ashcroft DM, Li Wan Po A, Williams HC, Griffiths CEM. Systematic review of comparative efficacy and tolerability of calcipotriol in treating chronic plaque psoriasis. Br. Med. J. . A. Li Wan Po, Centre Evidence-Based Pharmacother., School of Life Health Sciences, Aston University, Birmingham B4 7ET, United Kingdom; 2000;320:963–7.

1145. Ashcroft DM, Po ALW, Williams HC, Griffiths CEM. Combination regimens of topical calcipotriene in chronic plaque psoriasis: Systematic review of efficacy and tolerability. Arch. Dermatol. . D.M. Ashcroft, Department of Medicines Management, Keele University, Keele, Staffordshire ST5 5BG, United Kingdom; 2000;136:1536–43.

1146. Gambichler T, Kreuter JA, Altmeyer P, Hoffmann K. Meta-analysis of the efficacy of Balneophototherapy. Aktuelle Derm. . T. Gambichler, Klin. für Dermatol./Allergol., Universität Bochum, 44791 Bochum, Germany; 2000;26:402–6.

1147. Griffiths CEM, Clark CM, Chalmers RJG, Li Wan Po A, Williams HC. A systematic review of treatments for severe psoriasis. Health Technol. Assess. (Rockv). . C.E.M. Griffiths, Hope Hospital, University of Manchester, School of Medicine, Salford, United Kingdom; 2000;4:i+iii – v+1–115.

1148. Guenther L. Tazarotene combination treatments in psoriasis. J. Am. Acad. Dermatol. . L. Guenther, London, Ont. N6A 3H7, Canada; 2000;43:S36–42.

1149. Lebwohl M. Strategies to optimize efficacy, duration of remission, and safety in the treatment of plaque psoriasis by using tazarotene in combination with a corticosteroid. J. Am. Acad. Dermatol. . M. Lebwohl, Department of Dermatology, Mount Sinai Hospital, New York, NY 10029, United States; 2000;43:S43–6.

1150. Menter A. Pharmacokinetics and safety of tazarotene. J. Am. Acad. Dermatol. . A. Menter, Division of Dermatology, Baylor University Medical Center, Dallas, TX 75246, United States; 2000;43:S31–5.

1151. Calcipotriol (Daivonex®) - New administration form. Geneesmiddelenbulletin . 2000;34:99–100.

**1999**

1152. Asadullah K, Sabat R, Wiese A, Döcke W-D, Volk H-D, Sterry W. Interleukin-10 in cutaneous disorders: Implications for its pathophysiological importance and therapeutic use. Arch. Dermatol. Res. . K. Asadullah, Department Experimental Dermatology, Schering AG, D-13342 Berlin, Germany; 1999;291:628–36.

1153. Chuang T-Y, Brashear R, Lewis C. Porphyria cutanea tarda and hepatitis C virus: A case-control study and meta-analysis of the literature. J. Am. Acad. Dermatol. . T.-Y. Chuang, Department of Dermatology, Indiana University Medical Center, Indianapolis, IN 46202-5267, United States; 1999;41:31–6.

1154. Hannuksela-Svahn A, Pukkala E, Koulu L, Jansen CT, Karvonen J. Cancer incidence among Finnish psoriasis patients treated with 8- methoxypsoralen bath PUVA. J. Am. Acad. Dermatol. . A. Hannuksela-Svahn, Department of Dermatology, University of Oulu, FIN-90220 Oulu, Finland; 1999;40:694–6.

1155. Mallon E, Newson R, Bunker CB. HLA-Cw6 and the genetic predisposition to psoriasis: A meta-analysis of published serologic studies [2]. J. Invest. Dermatol. . E. Mallon, Department of Dermatology, Chelsea and Westminster Hospital, Imperial College School of Medicine, London, United Kingdom; 1999;113:693–5.

1156. Pasker-De Jong PCM, Wielink G, Van Der Valk PGM, Van Der Wilt G-J. Treatment with UV-B for psoriasis and nonmelanoma skin cancer: A systematic review of the literature. Arch. Dermatol. . P.C.M. Pasker-de Jong, Department of Epidemiology 152, University of Nijmegen, 6500 HB Nijmegen, Netherlands; 1999;135:834–40.

1157. Rice K, Spencer J. Inhibitors of human mast cell serine proteases and potential therapeutic applications. Expert Opin. Ther. Pat. . K. Rice, Dept. of Medicinal Chemisty, Axys Pharmaceuticals, Inc., South San Francisco, CA 94080, United States; 1999;9:1537–55.

1158. Shanahan W.R. J. ISIS 2302, an antisense inhibitor of intercellular adhesion molecule 1. Expert Opin. Investig. Drugs . W.R. Shanahan Jr., Isis Pharmaceuticals Inc., Carlsbad, CA 92008-7208, United States; 1999;8:1417–29.

1159. Vogler BK, Ernst E. Aloe vera: A systematic review of its clinical effectiveness. Br. J. Gen. Pract. . E. Ernst, Department of Complementary Medicine, Sch. Postgrad. Med. Health Sciences, University of Exeter, Exeter EX2 4NT, United Kingdom; 1999;49:823–8.

1160. Fish oil supplements. Geneesmiddelenbulletin . 1999;33:37–42.

**1998**

1161. Koo J, Arain S. Traditional chinese medicine for the treatment of dermatologic disorders. Arch. Dermatol. . J. Koo, Psoriasis Treatment Center, San Francisco, CA 94118, United States; 1998;134:1388–93.

1162. Marchetti A, LaPensee K, An P. A pharmacoeconomic analysis of topical therapies for patients with mild- to-moderate stable plaque psoriasis: A US study. Clin. Ther. . A. Marchetti, Health Economics Research, Secaucus, NJ 07094, United States; 1998;20:851–69.

1163. McNeely W, Goa KL. 5-methoxypsoralen. A review of its effects in psoriasis and vitiligo. Drugs . W. McNeely, Adis International Limited, Mairangi Bay, Auckland 10, New Zealand; 1998;56:667–90.

1164. Spuls PI, Bossuyt PMM, Van Everdingen JJE, Witkamp L, Bos JD. The development of practice guidelines for the treatment of severe plaque form psoriasis. Arch. Dermatol. . P.I. Spuls, Department of Dermatology, Academic Medical Center, University of Amsterdam, 1100 DE Amsterdam, Netherlands; 1998;134:1591–6.

1165. Stern RS, Lunder EJ. Risk of squamous cell carcinoma and methoxsalen (psoralen) and UV-A radiation (PUVA): A meta-analysis. Arch. Dermatol. . R.S. Stern, Department of Dermatology, Beth Israel Deaconess Medical Center, Harvard Medical School, Boston, MA 02215, United States; 1998;134:1582–5.

1166. Vercauteren SB, Bosmans J-L, Elseviers MM, Verpooten GA, De Broe ME. A meta-analysis and morphological review of cyclosporine-induced nephrotoxicity in auto-immune diseases. Kidney Int. . M.E. De Broe, University of Antwerp, Dept. of Nephrology-Hypertension, p/a University Hospital Antwerp, B-2650 Edegem/Antwetpen, Belgium; 1998;54:536–45.

1167. Williams HC, Po ALW, Murrel D, Naldi L, Diepgen T, Spuls PI, et al. A systematic review of five systemic treatments for severe psoriasis [15] (multiple letters). Br. J. Dermatol. . A.L.W. Po, Department of Pharmaceutical Science, University of Nottingham, Nottingham NG7 2RD, United Kingdom; 1998;139:757.

1168. Antiseptic/emollient combinations. Drug Ther. Bull. . 1998;36:84–6.

**1997**

1169. Mahé A, Bobin P, Coulibaly S, Tounkara A. Skin diseases revealing human immunodeficiency virus infection in Mali. Ann. Dermatol. Venereol. . A. Mahe, Unite de Dermatologie, Centre Hospitalier Universitaire, 97159 Pointe-a-Pitre Cedex, Guadeloupe; 1997;124:144–50.

1170. Mahrle G. Dithranol. Clin. Dermatol. . G. Mahrle, Department of Dermatology, University of Cologne, D-50924 Cologne, Germany; 1997;15:723–37.

1171. Oh PI, Gupta AK, Einarson TR, Maerov P, Shear NH. Calcipotriol in the treatment of psoriasis of limited severity: Pharmacoeconomic evaluation. J. Cutan. Med. Surg. . P.I. Oh, Sunnybrook Health Science Centre, Toronto, Ont. M4N 3M5, Canada; 1997;2:7–15.

1172. Orfanos CE, Zouboulis CC, Almond-Roesler B, Geilen CC. Current use and future potential role of retinoids in dermatology. Drugs . C.E. Orfanos, Department of Dermatology, Univ. Medical Ctr Benjamin Franklin, The Free University of Berlin, 12200 Berlin, Germany; 1997;53:358–88.

1173. Peters DC, Balfour JA. Tacalcitol. Drugs . D.C. Peters, Adis International Limited, Mairangi Bay, Auckland 10, New Zealand; 1997;54:265–72.

1174. Rosenthal D, Guenther L, Kelly J. Current thoughts on the use of methotrexate in patients with psoriasis. J. Cutan. Med. Surg. . D. Rosenthal, Department of Medicine, Division of Dermatology, McMaster University, Hamilton, Ont. L8N 3Z5, Canada; 1997;2:41–6.

1175. Spuls PI, Witkamp L, Bossuyt PMM, Bos JD. A systematic review of five systemic treatments for severe psoriasis. Br. J. Dermatol. . P.M.M. Bossuyt, Clinical Epidemiology/Biostatistics, Academisch Medisch Centrum, University of Amsterdam, 1105 AZ Amsterdam, Netherlands; 1997;137:943–9.

1176. Methotrexate in rheumatoid arthritis and psoriasis. Prescrire Int. . 1997;6:96–100.

**1996**

1177. Bryson HM, Wagstaff AJ. Liarozole. Drugs and Aging . H.M. Bryson, Adis International Limited, Auckland, New Zealand; 1996;9:478–85.

1178. Lipsky JJ. Mycophenolate mofetil. Lancet . J.J. Lipsky, Department of Medicine, Clinical Pharmacology Unit, Mayo Clinic, Rochester, MN 55905, United States; 1996;348:1357–9.

1179. Marks R. Early clinical development of tazarotene. Br. J. Dermatology, Suppl. . R. Marks, Department of Dermatology, University of Wales, College of Medicine, Cardiff, United Kingdom; 1996;135:26–31.

**1995**

1180. Chu AC, Munn S. Fluticasone propionate in the treatment of inflammatory dermatoses. Br. J. Clin. Pract. . A.C. Chu, Unit of Dermatology, Royal Postgraduate Medical School, Hammersmith Hospital, London W12 0NN, United Kingdom; 1995;49:131–3.

1181. Endres S, De Caterina R, Schmidt EB, Kristensen SD. n-3 Polyunsaturated fatty acids: Update 1995. Eur. J. Clin. Invest. . S. Endres, Medizinische Klinik, Klinikum Innenstadt, Ludwig-Maximilians-Universitat, D-80336 Munchen, Germany; 1995;25:629–38.

1182. Kruger K. Cyclosporin A in the treatment of rheumatoid arthritis and other rheumatic diseases. Z. Rheumatol. . K. Kruger, Rheuma-Einheit, Ludwig-Maximilians-Universitat, Klinik Innenstadt, 80336 Munchen, Germany; 1995;54:89–95.

1183. Zachariae H, Steen Olsen T. Efficacy of cyclosporin A (CyA) in psoriasis: An overview of dose/response, indications, contraindications and side-effects. Clin. Nephrol. . H. Zachariae, Department of Dermatology, Marselisborg Hospital, DK-8000 Aarhus C, Denmark; 1995;43:154–8.

1184. Once-a-day topical corticosteroids. Drug Ther. Bull. . 1995;33:21–2.

**1994**

1185. Arikian SR, Einarson TR, Sander HM, Menter A. The annual cost of psoriasis [6]. J. Am. Acad. Dermatol. . S.R. Arikian, Faculty of Pharmacy, University of Toronto, Toronto, Ont., Canada; 1994;30:1047–8.

1186. Einarson TR, Arikian SR, Shear N. Oral treatments for severe psoriasis: Government payor analysis for Canada. J. Dermatolog. Treat. . T.R. Einarson, Faculty of Pharmacy, Toronto, Ont M5S 2S2, Canada; 1994;5:S23–7.

1187. Geijer RMM, Meulenberg FAEM, Ek JW. The effectivity of topical drug therapy in psoriasis. Huisarts Wet. . R.M.M. Geijer, Nederlands Huisartsen Genootschap, 3502 GE Utrecht, Netherlands; 1994;37:243–7+275.

1188. Zhang WY, Po ALW. The effectiveness of topically applied capsaicin. A meta-analysis. Eur. J. Clin. Pharmacol. . A.L.W. Po, School of Pharmacy, Queen’s University of Belfast, Belfast BT9 7BL, United Kingdom; 1994;46:517–22.

**1991**

1189. Whiting-O’Keefe QE, Fye KH, Sack KD. Methotrexate and histologic hepatic abnormalities: A meta-analysis. Am. J. Med. . Q.E. Whiting-O’Keefe, Medaware, Inc., Redwood City, CA 94062, United States; 1991;90:711–6.

**1975**

1190. Mali JWH, Kuiper JP, Van De Staak WJBM, Groels AP. Treatment of psoriasis (Dutch). Ned. Tijdschr. Geneeskd 1975;119:89–93.

**Table C.** PROSPERO register file.

**Table D.** AMSTAR checklist. The tool contains 11 questions (Q1-Q11) with regard to the quality of the review. Every question should be assigned a score 0 or 1. The sum of all scores is the overall methodological quality score of the systematic review.

| Item | Question | Responses | Code values |
| --- | --- | --- | --- |
| Q1 | Was an "a priori" design provided? | Yes[[1]](#footnote-2)/no/Can't answer/NA | 1/0/0/0 |
| Q2 | Was there duplicate study selection and data extraction? | Yes[[2]](#footnote-3)/no/Can't answer/NA | 1/0/0/0 |
| Q3 | Was a comprehensive literature search performed? | Yes[[3]](#footnote-4)/no/Can't answer/NA | 1/0/0/0 |
| Q4 | Was the status of publication (i.e. grey literature) used as an inclusion criterion? | Yes[[4]](#footnote-5)/no/Can't answer/NA | 1/0/0/0 |
| Q5 | Was a list of studies (included and excluded) provided? | Yes/no/Can't answer/NA | 1/0/0/0 |
| Q6 | Were the characteristics of the included studies provided? | Yes[[5]](#footnote-6)/no/Can't answer/NA | 1/0/0/0 |
| Q7 | Was the scientific quality of the included studies assessed and documented? | Yes[[6]](#footnote-7)/no/Can't answer/NA | 1/0/0/0 |
| Q8 | Was the scientific quality of the included studies used appropriately in formulating conclusions? | Yes[[7]](#footnote-8)/no/Can't answer/NA | 1/0/0/0 |
| Q9 | Were the methods used to combine the findings of studies appropriate? | Yes[[8]](#footnote-9)/no/Can't answer/NA | 1/0/0/0 |
| Q10 | Was the likelihood of publication bias assessed? | Yes[[9]](#footnote-10)/no/Can't answer/NA | 1/0/0/0 |
| Q11 | Was the conflict of interest included? | Yes[[10]](#footnote-11)/no/Can't answer/NA | 1/0/0/0 |

**2. Supplementary Figures.**

**Figure A.** Percentage of explained variances by dimension of multifactorial analysis (MFA).

**
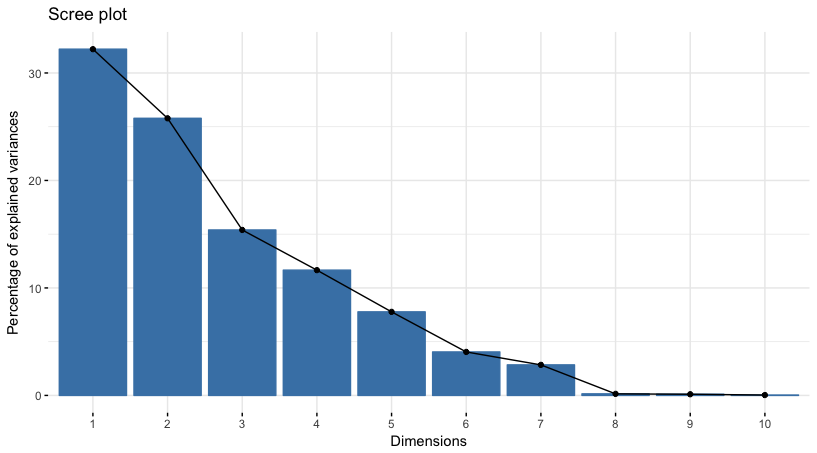
**

**Figure B.** PC1-PC2 projections of variable groups. Goup composition: 'amstar': AMSTAR levels; 'funding': source of funding; 'conflict of interest': number of authors with conflict of interest; 'social': Twitter, Facebook, and Google+ mention counts; 'readers': Mendeley and SCOPUS readers; cites: citation counts from Google Scholar.

**
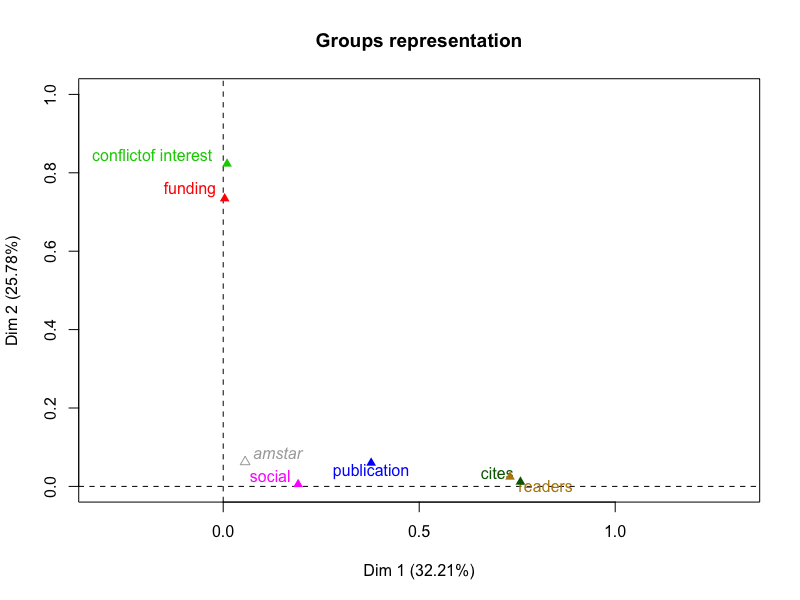
**

1. The research question and inclusion criteria were established before conducting the review. [↑](#footnote-ref-2)
2. At least two people working independently extracted the data and the method was reported for reaching consensus if disagreements arose. [↑](#footnote-ref-3)
3. At least two electronic sources were searched; details of the databases, years searched and search strategy were provided; the search was supplemented by searching of reference lists of included studies, and specialised registers, and by contacting experts. [↑](#footnote-ref-4)
4. The authors stated that they excluded studies from the review based on publication status. No−authors searched for reports irrespective of publication type. They did not exclude reports based on publication from the systematic review. [↑](#footnote-ref-5)
5. Data on participants, interventions and outcomes were provided, and the range of relevant characteristics reported. [↑](#footnote-ref-6)
6. Predetermined methods of assessing quality were reported. [↑](#footnote-ref-7)
7. The quality (and limitations) of included studies was used in the analysis, conclusions and recommendations of the review [↑](#footnote-ref-8)
8. If results were pooled statistically, heterogeneity was assessed and used to inform the decision of statistical model to be used. If heterogeneity was present, the appropriateness of combining studies was considered by review authors [↑](#footnote-ref-9)
9. Publication bias was explicitly considered and assessed. [↑](#footnote-ref-10)
10. Sources of support were clearly acknowledged. [↑](#footnote-ref-11)
